# LAPTM5 Plays a Key Role in the Diagnosis and Prognosis of Testicular Germ Cell Tumors

**DOI:** 10.1155/2021/8816456

**Published:** 2021-01-13

**Authors:** Xiunan Li, Yu Su, Jiayao Zhang, Ye Zhu, Yingkun Xu, Guangzhen Wu

**Affiliations:** ^1^Department of Urology, The First Affiliated Hospital of Dalian Medical University, Dalian, Liaoning 116011, China; ^2^Department of Endocrinology, Shandong Provincial Hospital, Cheeloo College of Medicine, Shandong University, Jinan, Shandong 250021, China; ^3^Department of Hepatobiliary Surgery and Center of Organ Transplantation, Shandong Provincial Hospital, Cheeloo College of Medicine, Shandong University, Jinan, Shandong 250021, China; ^4^Department of Orthopedic Surgery, The First Affiliated Hospital of Dalian Medical University, Dalian, Liaoning 116011, China; ^5^Department of Urology, Shandong Provincial Hospital, Cheeloo College of Medicine, Shandong University, Jinan, Shandong 250021, China

## Abstract

**Objective:**

Testicular germ cell tumors (TGCT) are a serious malignant tumor with low early diagnosis rates and high mortality.

**Methods:**

To investigate novel biomarkers to predict the diagnosis and prognosis of this cancer, bioinformatics analysis was used as an accurate, efficient, and economical method.

**Results:**

Our study detected 39 upregulated and 589 downregulated differentially expressed genes (DEGs) using the GEO and TCGA databases. To identify the function of DEGs, GO functional analysis, three pathway analysis (KEGG, REACTOME, and PANTHER), and protein-protein interaction network were performed using the KOBAS website, as well as the String database. After a series of analyses in GEPIA and TIMER, including differential expression, we found one candidate gene related to the prognosis and diagnosis of TGCT. LAPTM5 was also associated with CD8+ T cell and PDCD1 expression, which suggests that it may affect immune infiltration.

**Conclusions:**

LAPTM5 was identified as a hub gene, which could be used as a potential biomarker for TGCT diagnosis and prognosis.

## 1. Introduction

Testicular germ cell tumors (TGCT) are clinically rare malignant endocrine tumors [1], with an incidence of 0.002‰–0.005% in the worldwide. This cancer has various clinical manifestations and is prone to invasion and metastasis [2]. Due to the low rate of early diagnosis and high mortality, the survival period is generally no more than three years [3]. Complete surgical resection is regarded as the only potentially curative treatment for TGCT [4, 5]. Therefore, finding novel biomarkers for effective screening in the earlier stages of TGCT can be a powerful tool to improve the long-term survival.

Many studies have shown that prognosis is related to the levels of multiple molecules. Overexpression of KIT can be detected in most patients with TGCT [6]. The upregulation of cellular tumor antigen (FGFR3) [7] and proliferation marker protein (BCL10), [8, 9] are thought to be involved in the development of multiple tumors, including TGCT. Additionally, Wilkie et al. also verified that the expression of FGFR3 and HRAS was increased in TGCT compared to normal tissues [10]. However, it is still necessary to discover more representative candidate biomarkers for high-risk TGCT assessment and inadequate prognosis predictions.

Currently, the discovery of crucial tumor biomarkers using bioinformatics analysis has become a reliable and profitable method [11–13], providing a variety of basic information about gene functions, regulatory pathways, cellular network, and prognostic results of diverse diseases, which play an important role in practical research. In our study, we first downloaded raw data from GEO and TCGA databases to obtain differentially expressed genes (DEGs) in TGCT. We performed gene ontology, pathway enrichment analysis, and a protein-protein interaction (PPI) network. GEPIA was adopted to observe differential expression and prognostic characteristics of these genes. In addition, Tumor Immune Estimation Resource (TIMER) was used to determine the distribution in pan cancers, pathway enrichment, features in pathological parameters, and their relationship with other genes. We attempted to identify precise hub genes that may serve as novel biomarkers for TGCT.

## 2. Materials and Methods

### 2.1. Data Selection

The GEO database (http://www.ncbi.nlm.nih.gov/geo/) was used for identifying the data sets of gene expression between TGCT tissues and normal testis tissues [14]. Complete information regarding the relevant data sets was then evaluated. Finally, in line with the Affymetrix Human Genome (GPL570) platform, one data set, GSE3218, was chosen for subsequent analysis, which contained 101 TGCT samples and 6 normal samples. Moreover, TGCT datasets in TCGA were also used in the analysis, which contained 137 TGCT samples and 165 normal samples.

### 2.2. Differential Expression Analysis

The R language was used to analyze GEO data and draw volcano plots and heatmaps [15]. |Log_2_FC|>1, adjusted *P* value < 0.05 were considered the cutoff criterion to identify the differentially expressed genes (DEGs). To ensure more accurate results, we also analyzed DEGs in GSE3218 to verify TCGA data [16]. An online tool, Bioinformatics & Evolutionary Genomics Bioinformatics & Evolutionary Genomics (http://bioinformatics.psb.ugent.be/webtools/Venn/) (21079755), was used to draw the Venn diagram for upregulated and downregulated DEGs.

### 2.3. Gene Ontology and Pathway Enrichment Analysis

The upregulated and downregulated DEGs were integrated into the KOBAS website [17]; we performed Gene Ontology (GO) functional annotation enrichment analysis [18] and KEGG [19], REACTOME [20], and PANTHER [21] pathway enrichment analysis. The adjusted *P* value of <0.05 was considered statistically significant.

### 2.4. PPI Network and Identification of Hub Genes

The PPI network between target genes was built using the String database (http://stringdb.org/) [22]. String is an online database for searching known proteins and predicting protein interactions. This interaction includes not only the direct interaction between proteins but also the correlation of the indirect functions of proteins. First, we entered the target genes into the database and set the confidence score as ≥0.7. Then, the unlinked proteins were removed, and the remaining protein interaction data and panoramic images were obtained.

### 2.5. Gene Expression Analysis and Survival Analysis

GEPIA (http://gepia.cancerpku.cn/detail.php) is a newly developed interactive web server for analyzing the RNA sequencing expression data of 9736 tumors and 8587 normal samples in TCGA and GTEx projects [23]. Based on the analysis in GEPIA database, all genes associated with survival of TGCT were found. Furthermore, we identified genes related to DEGs and survival-related genes as TGCT risked genes.

### 2.6. Mutation Analysis in Risked Genes

The TGCT (TCGA, Provisional) dataset was selected, comprising data from 154 pathology reports. These genes were further analyzed using cbioportal (http://www.cbioportal.org/index.do) [24]. Genomic analysis was covered with mutations and coexpression analysis. Coexpression and networking were calculated based on the online instructions provided for the cbioportal. The adjusted *P* value < 0.05 was considered statistically significant.

### 2.7. Immune Infiltration Analysis

TIMER (https://cistrome.shinyapps.io/timer/) was used to analyze immune infiltration among pan-cancer [25]. In this study, we used this online website to analyze the association between hub genes and immune infiltration.

## 3. Results

### 3.1. DEGs in TGCT

**Figure 1:**
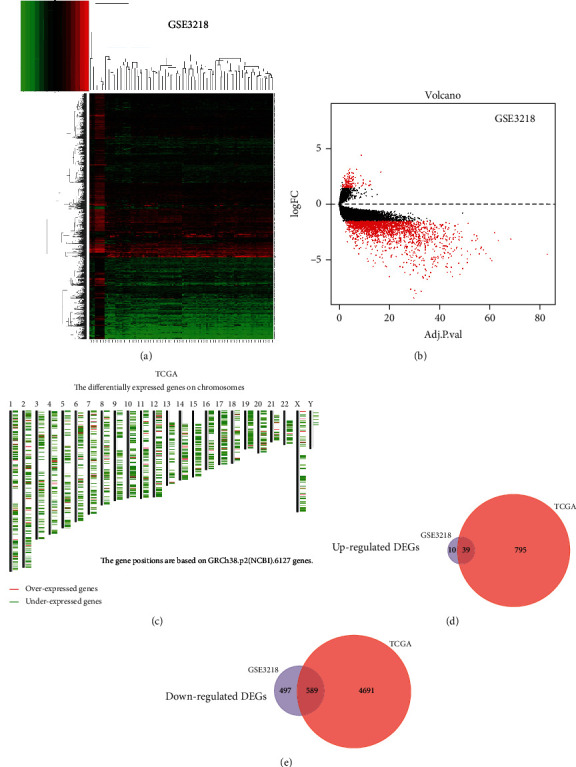
Identification of differentially expressed genes in TGCT. (a) Heatmap of GSE3218. (b) Volcano plot of GSE3218. (c) The location of differentially expressed genes of TCGA on chromosomes. Red represents overexpressed genes, and blue represents underexpressed genes. (d, e) Venn diagram of DEGs among GSE3218 and TCGA data.

Using the limma package, DEGs in GSE3218 were identified (Figure 1(a)). As shown in Figure 1(b), 49 overexpressed genes and 1086 low-expressed genes were considered as DEGs. Moreover, DEGs in TCGA datasets were also identified. The distribution of these DEGs, identified using TCGA, on different chromosomes is shown in Figure 1(c). Finally, DEGs in TGCT were identified by taking the intersection between the GEO datasets and TCGA data. A total of 628 DEGs consisting of 39 upregulated genes and 589 downregulated genes were obtained in our study (Figures 1(d) and 1(e)). Among them, the detailed information of these genes involved in the above two Venn diagrams are organized in Supplementary materials Table S1-S2.

### 3.2. Functional Enrichment of DEGs

**Figure 2:**
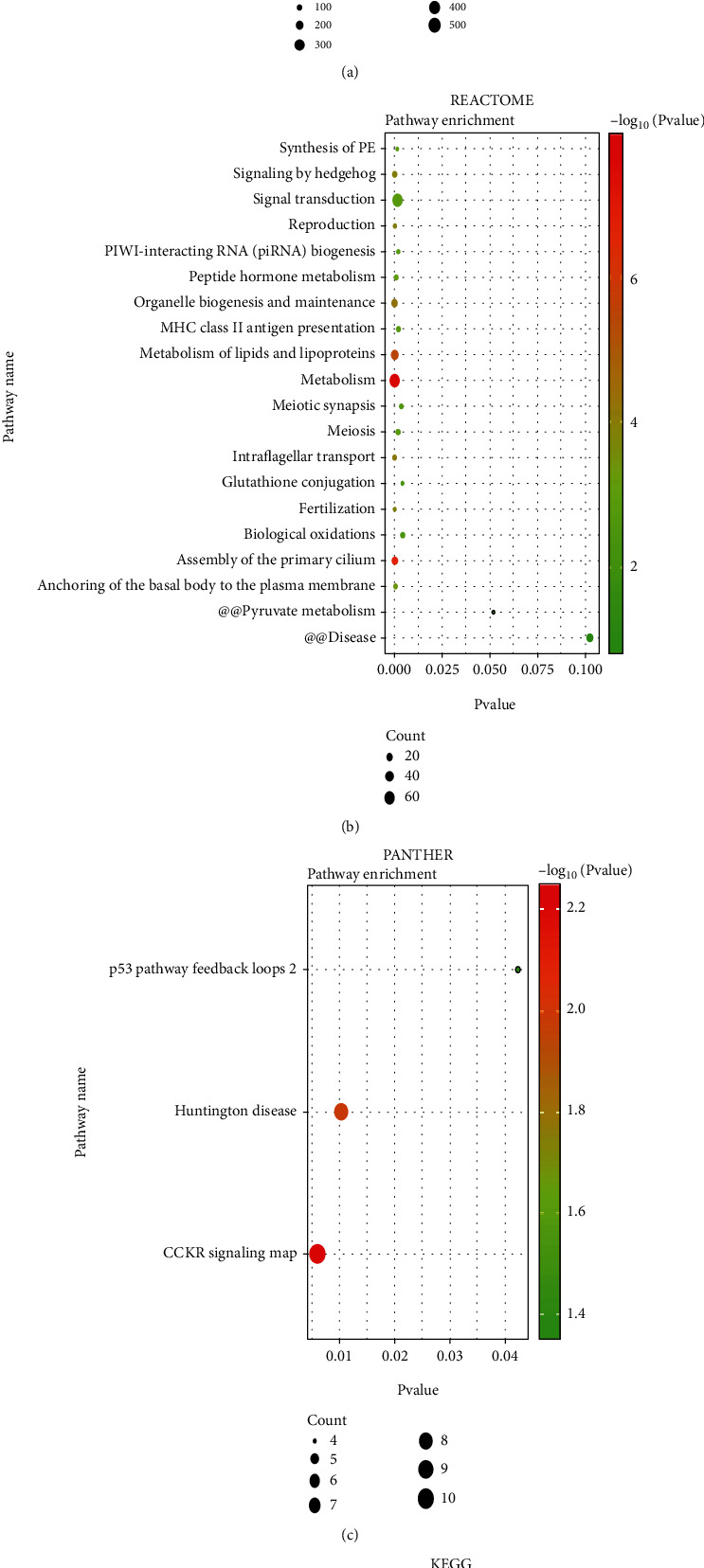
Function analysis and pathway analysis of DEGs. (a–d) Bubble plots of GO, REACTOME, PANTHER, and KEGG analysis.

GO functional enrichment analysis was performed on the identified DEGs, demonstrating that most genes are related to the regulation of cellular processes, metabolic processes, and biological regulation (Figure 2(a)). The detailed statistical information of GO analysis is shown in Supplementary materials Table S3. In addition, using three pathway databases (KEGG, REACTOME, and PANTHER) revealed that TGCT-related DEGs were mainly concentrated in metabolic pathways, salivary secretion, and the TP53 pathway (Figures 2(b)–2(d)). The detailed statistical information of the above three pathway analysis is shown in Supplementary materials Table S4-S6.

### 3.3. Identification of TGCT-Associated Risk Genes

**Figure 3:**
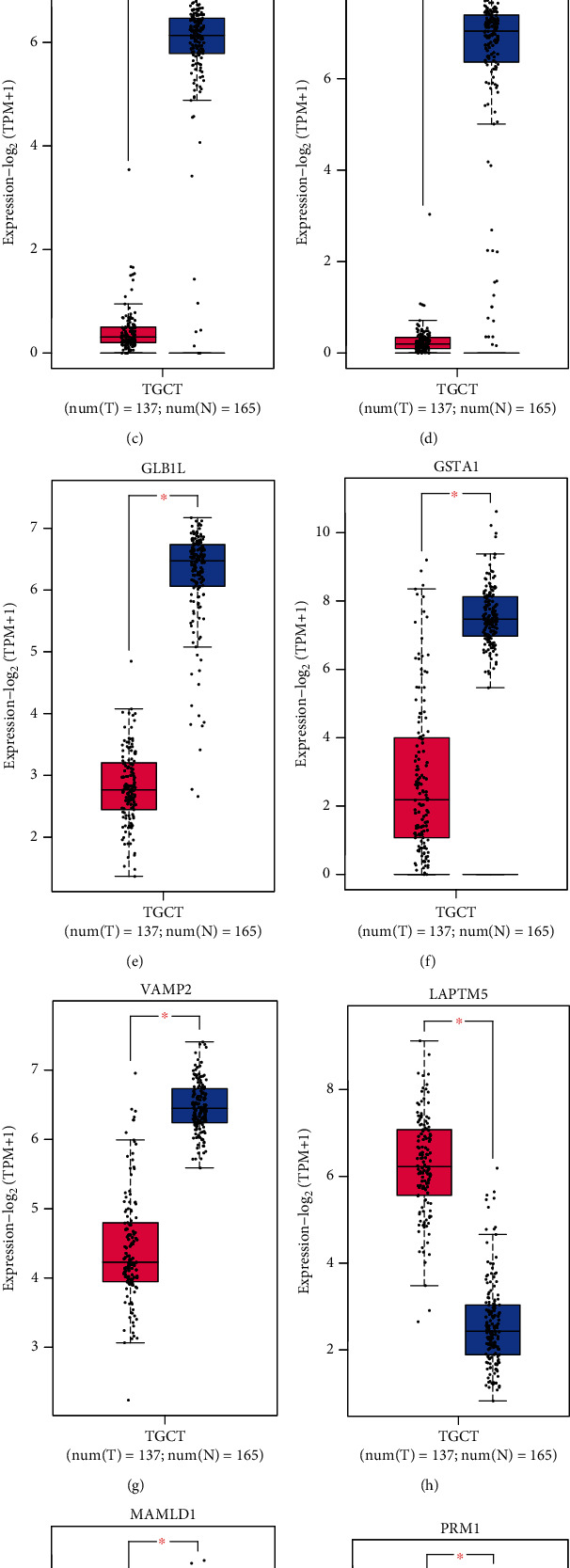
Cancer-related risk genes in TGCT. (A) Venn diagram of DEGs and survival-related genes in TGCT. (b–j) Gene expression in cancerous and normal samples. Red represents TGCT samples, and blue represents normal samples. ^∗^*P* < 0.05.

**Figure 4:**
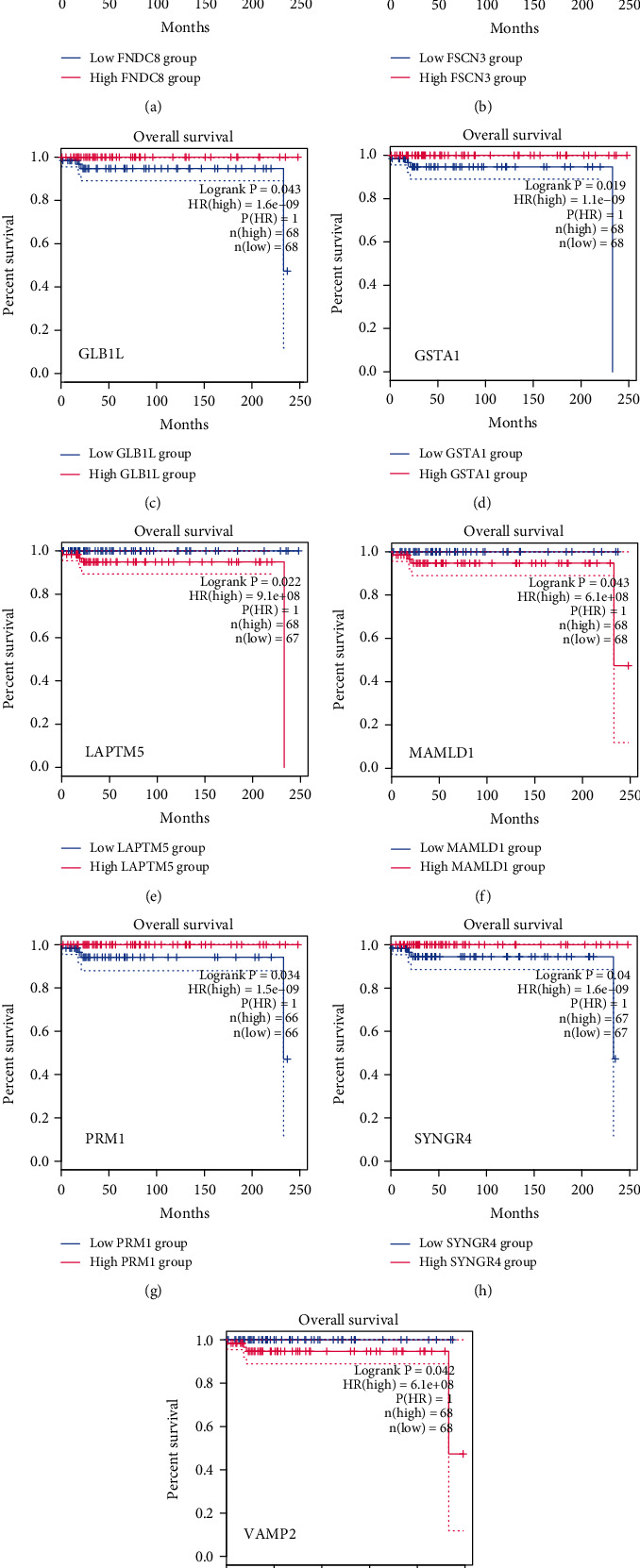
Survival plots for the nine risk-related genes. (a–i) FNDC8, FSCN3, GLB1L, GSTA1, LAPTM5, MAMLD1, PRM1, SYNGR4, and VAMP2, respectively. Red represents the high gene expression group, and blue represents the low gene expression group. *P* values are shown.

**Figure 5:**
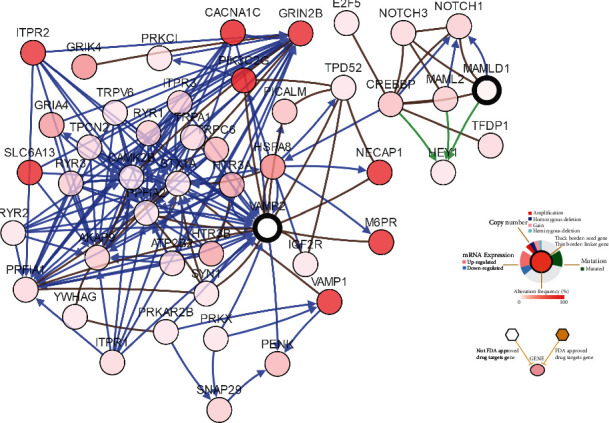
Protein-Protein Interaction (PPI) network. (a) PPI network diagram between nine risk-related genes and their related genes.

Based on the analysis of TCGA data, we identified genes related to prognosis. To better determine the TGCT-associated risk genes, we crossed the DEGs with those related to prognosis. As a result, nine genes were identified (Figure 3(a)), which are SYNGR4, FNDC8, FSCN3, GLB1L, GSTA1, LAPTM5, VAMP2, MAMLD1, and PRM1. The detailed information about this Venn diagram can be obtained through Supplementary materials Table S7. Till now, we have not distinguished whether these DEGs are highly or underexpressed. Therefore, we subsequently used the GEPIA website based on TCGA database to explore their expression levels (Figures 3(b)–3(j)) and their correlation with overall survival (Figures 4(a)–4(i)). Above all, we found genes associated with high expression (poor prognosis), such as LAPTM5. Similarly, we also found genes with low expression (good prognosis), such as MAMLD1 and VAMP2. Moreover, coexpression analysis was used for the TGCT-associated risk genes. As shown in Figure 5(a), most of the genes were correlated with VAMP2.

### 3.4. Hub Gene Identification

**Figure 6:**
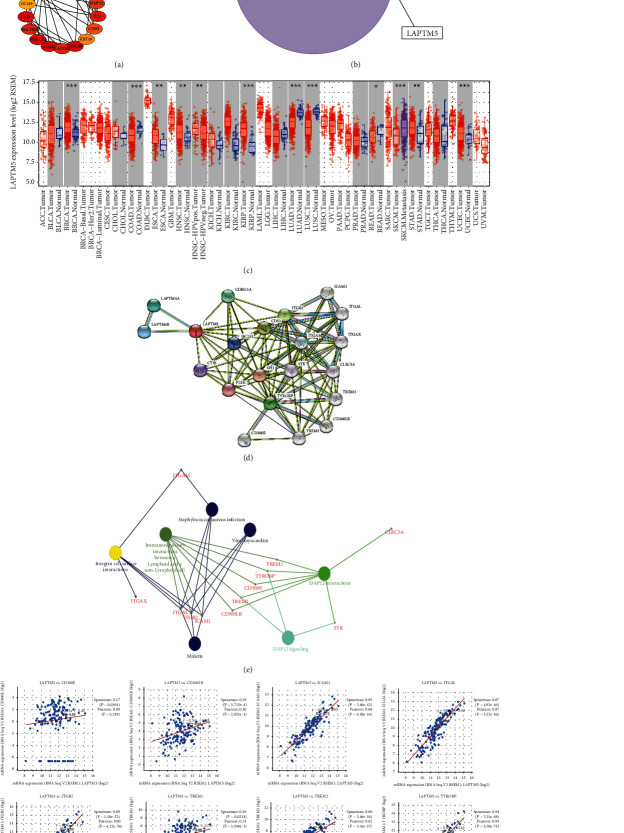
Schematics of the hub genes. (a) Top 40 hub genes by degree. (b) Venn diagram for the hub gene intersection. (c) The pan-cancer expression for LAPTM5. ^∗^*P* < 0.05, ^∗∗^*P* < 0.01, ^∗∗∗^*P* < 0.001. (d) Protein-protein interaction network for LAPTM5 and its related gene. (e) Pathway analysis for LAPTM5 and its related gene. (f) The relationship between the co-expression of LAPTM5 and its related genes. *P* values are shown.

To further identify the associated hub gene, we searched for the top 40 hub genes using the cytohubba plug-in in the Cytoscape software (Figure 6(a)) and then overlapped the overall survival-related genes intersections (Figure 6(b)) to find LAPTM5 as the TGCT-related hub gene. The detailed information related to this Venn diagram is shown in Supplementary materials Table S8. Combined with the previous findings that LAPTM5 is abnormally highly expressed in TGCT, and the prognosis of the LAPTM5 high expression group is significantly worse than that of the low expression group. Therefore, we determined that LAPTM5 is a hub gene in TGCT. In addition, we found that LAPTM5 is highly expressed in some cancer types, such as ESCA, KIRP, and KIRC. In contrast, LAPTM5 has a lower expression in other cancer types, such as LUAD, HNSC, and LUSC. This suggests that the LAPTM5 gene plays different biological roles in different cancer types (Figure 6(c)). Then, using LAPTM5 and its most related genes in the string dataset (Figure 6(d)), pathway analysis was used to identify the LAPTM5 pathway function (Figure 6(e)). The results indicate that there may be a correlation between these genes and immunoregulatory interactions between a Lymphoid and a non-Lymphoid cell. The relationship between the coexpression of LAPTM5 and its related genes is shown in Figure 6(f).

### 3.5. Analysis of the Correlation between LAPTM5 and Immunity

**Figure 7:**
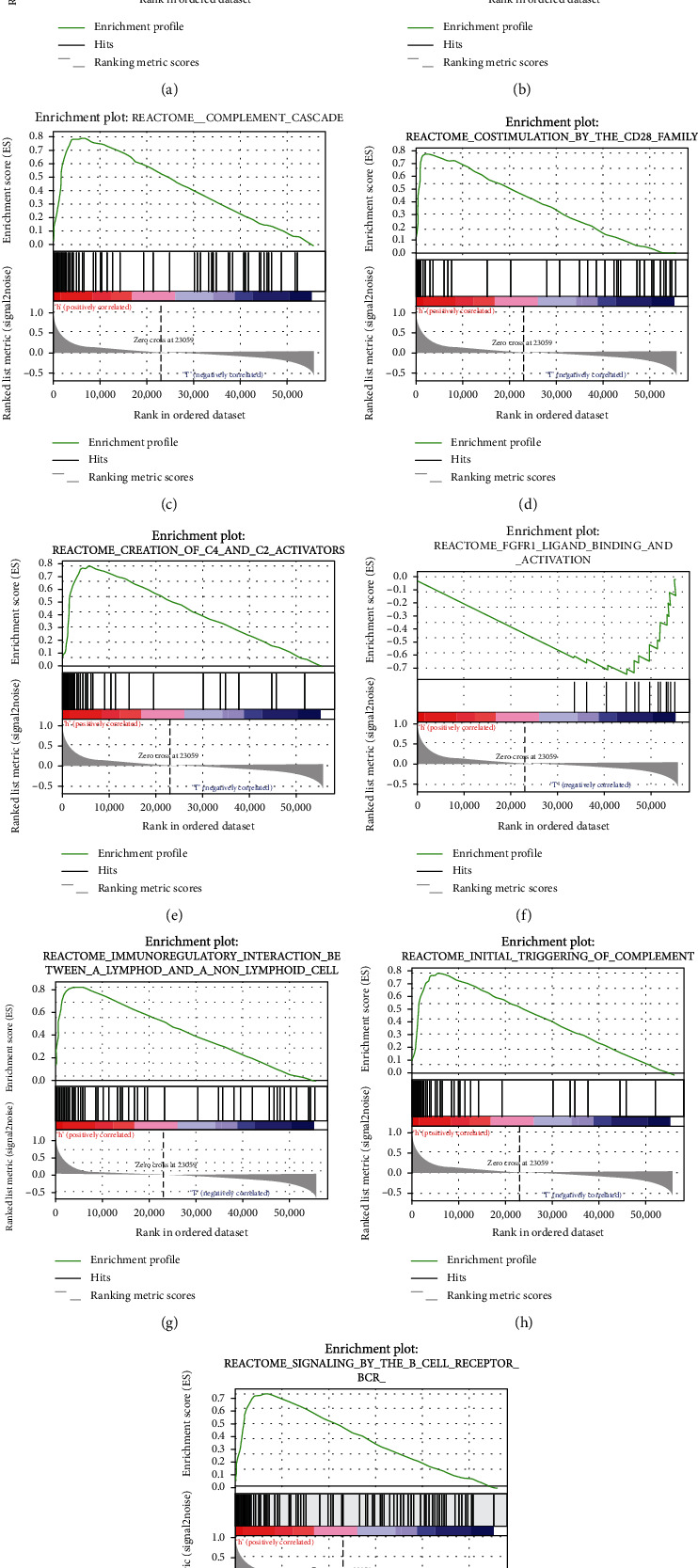
GSEA. (a) Adherens junctions interactions. (b) Antigen activates B cell receptor BCR leading to generation of second messengers. (c) Complement cascade. (d) Costimulation by the CD28 family. (e) Creation of C4 and C2 activators. (f) FGFR1 ligand binding and activation. (g) Immunoregulatory interactions between a Lymphoid and a non-Lymphoid cell. (h) Initial triggering of complement. (i) Signaling by the B cell receptor BCR.

**Figure 8:**
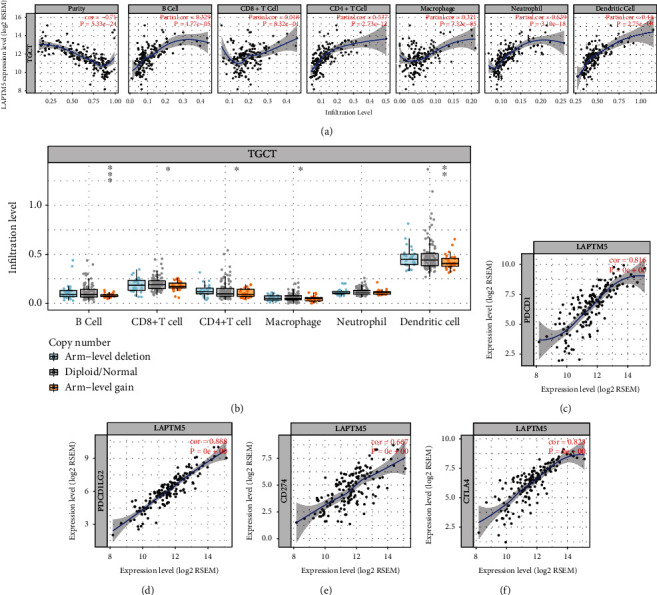
Relationship between LAPTM5 and immune responses. (a, b) Relationship between LAPTM5 and immune infiltration. ^∗^*P* < 0.05, ^∗∗^*P* < 0.01, ^∗∗∗^*P* < 0.001. (c–f) Relationship between LAPTM5 and immune checkpoints. *P* values are shown.

In order to explore the correlation between LAPTM5 and immunity, we performed GSEA of the LAPTM5 gene associated with TGCT. The analysis results show that LAPTM5 can activate antigen-activated B cell receptor (BCR), leading to the generation of second messengers (Figure 7(b)), complement cascade (Figure 7(c)), costimulation by the CD28 family (Figure 7(d)), creation of C4 and C2 activators (Figure 7(e)), immunoregulatory interactions between a Lymphoid and a non-Lymphoid cell (Figure 7(g)), initial complement triggering (Figure 7(h)), and signaling using the BCR biological pathway (Figure 7(i)). Secondly, LAPTM5 inhibited adherens junction interactions (Figure 7(a)), FGFR1 ligand binding, and activation of biological pathways (Figure 7(f)). Furthermore, the correlation between LAPTM5 and immune infiltration was analyzed using the TIMER website. As shown in Figures 8(a) and 8(b), LAPTM5 was associated with purity and CD8+ T cells. Moreover, the immune checkpoint genes were related to LAPTM5 expression. As shown in Figures 8(c)–8(f), PDCD1 showed coexpression with LAPTM5.

## 4. Discussion

In the past 20 years, molecular biology studies on TGCT have made great progress [26–29], but the main pathogenesis of this cancer is still unclear. As a highly malignant tumor, there is an urgent need to find effective diagnostic and prognostic markers for identifying early-stage patients, developing valid treatments, and improving the ineffective prognosis of TGCT.

In our research, GSE3218 (6 normal testis tissues and 101 TGCT tissues) was selected from the GEO database. After the results obtained from using the R language were correlated with data from TCGA, 39 upregulated genes and 589 downregulated genes were identified in our study. These genes may play a critical role in the development of TGCT. We also carried out GO functional analysis and pathway analysis (KEGG, REACTOME, and PANTHER) using the KOBAS website to learn the gene function and regulatory processes of these candidates. In the functional analysis, the TP53 pathway was associated with DEGs, which is consistent with the results of previous research [30]. The TP53 pathway plays an important role in TGCT. Previous studies have shown that TP53 pathway is abnormally expressed in TGCT [31]. In addition, microRNA-214 can regulate the progress of TGCT by regulating the activity of the TP53 pathway [32].

Moreover, we identified the DEGs and survival-associated genes. Furthermore, nine genes (FNDC8, SYNGR4, FSCN3, GLB1L, GSTA1, LAPTM5, MAMLD1, VAMP2, and PRM1) were identified as both DEGs and survival-related genes. Glutathione S-transferase alpha 1 (GSTA1) encodes a member of the enzyme family whose function is to add glutathione to therapeutic drug carcinogens and oxidative stress products. This action is an essential step in the detoxification of these compounds. This family of enzymes plays a special role in protecting cells from reactive oxygen species and peroxidation products. The polymorphism of this gene affects the individual's ability to metabolize different drugs. This gene is located in a cluster of similar genes and pseudogenes on chromosome 6. Alternative splicing results in multiple transcript variants. Many studies have shown the relationship between GSTA1 and cancer, including breast cancer, hepatocellular carcinoma [33], and colorectal cancer [34]. Mastermind-like domain containing 1 (MAMLD1) encodes a mastermind-like domain-containing protein. This protein may function as a transcriptional coactivator. Mutations in this gene cause X-linked hypospadias type 2 and alternate splicing results in multiple transcript variants. Using mass spectrometry, Mohammed et al. found that some phosphorylated proteins, including MAMLD1, affect tumorigenesis [35]. Protamine 1 (PRM1) was found to affect colon cancer proliferation, invasion, migration, diagnosis, and prognosis [36]. Other genes, similar to galactosidase beta 1 like (GLB1L), fibronectin type iii domain containing 8 (FNDC8), synaptogyrin 4 (SYNGR4), MAMLD1, and fascin actin-bundling protein 3 (FSCN3), which is a novel paralog of action binding protein fascin, especially expressed in slender sperm heads, have not been reported to be associated with tumors. These genes should be investigated in-depth in TGCT in the future.

We further identified hub genes affecting TGCT through core protein network and prognosis analysis. As a result of this analysis, we found that LAPTM5 encodes a transmembrane receptor that is associated with lysosomes. The encoded protein, also known as E3 protein, may play a role in hematopoiesis. Previous studies have shown that LAPTM5 is a protein preferentially expressed in immune cells and that it interacts with the Nedd4 family of ubiquitin ligases. LAPTM5 is an active regulator of the proinflammatory signaling pathways in macrophages [37]. In addition, we performed GSEA in TGCT for LAPTM5, the results of which showed that there is a correlation between LAPTM5 and multiple important immune pathways. In bladder cancer cells, Chen et al. reported that low expression of LAPTM5 suppresses cell cycle changes [38]. LAPTM5 can also be a biomarker in lung cancer, which shows a correlation between methylation and genes [39]. In TGCT, we found abnormal high expression of LAPTM5 in cancer samples. Moreover, LAPTM5 can also affect tumor prognosis. However, at present, laboratory and clinical studies of LAPTM5 in TGCT have not been carried out, and its potential mechanism needs to be confirmed by further studies.

## 5. Conclusions

In summary, based on the results of a series of bioinformatic analyses, our study concluded that LAPTM5 is particularly important for the high-risk assessment and inadequate prognosis of TGCT, suggesting that LAPTM5 could be a very useful biomarker for determining the accurate diagnosis and prognosis of this disease. However, more efforts should be invested in clinical experiments to learn the biological functions and pathological importance of these genes in TGCT. In addition, we believe that our research can provide valuable data for future scientific research.

## Data Availability

The data used to support the findings of this study are available from the corresponding author upon request.

## Abbreviations

LAPTM5: Lysosomal protein transmembrane 5
GEO: Gene expression omnibus
TCGA: The Cancer Genome Atlas
GO: Gene Ontology
KEGG: Kyoto Encyclopedia of Genes and Genomes
TGCT: Testicular germ cell tumors
PDCD1: Programmed cell death 1
BCL10: BCL10 immune signaling adaptor
FGFR3: Fibroblast growth factor receptor 3
PPI: Protein-protein interaction
DEGs: Differentially expressed genes
FNDC8: Fibronectin type III domain containing 8
SYNGR4: Synaptogyrin 4
FSCN3: Fascin actin-bundling protein 3
GLB1L: Galactosidase beta 1 like
GSTA1: Glutathione S-transferase alpha 1
VAMP2: Vesicle associated membrane protein 2
MAMLD1: Mastermind like domain containing 1
PRM1: Protamine 1
ESCA: Esophageal carcinoma
KIRP: Kidney renal papillary cell carcinoma
KIRC: Kidney renal clear cell carcinoma
LUAD: Lung adenocarcinoma
HNSC: Head and neck squamous cell carcinoma
LUSC: Lung squamous cell carcinoma.

## Supplementary Materials

Supplementary materials Table S1: the detailed information on Venn diagrams related to upregulated DEGs. Table S2: the detailed information on Venn diagrams related to downregulated DEGs. Table S3: the detailed statistical information related to GO analysis. Table S4: the detailed statistical information related to REACTOME analysis. Table S5: the detailed statistical information related to PANTHER analysis. Table S6: the detailed statistical information related to KEGG analysis. Table S7: the detailed information about the Venn diagram between OS-related genes and DEGs. Table S8: the detailed information about the Venn diagram between OS-related genes and Top 40 hub genes.
