# Supplementary material for: LAPTM5 Plays a Key Role in the Diagnosis and Prognosis of Testicular Germ Cell Tumors
Source: Int J Genomics. 2021 Jan 13;2021:8816456. doi: 10.1155/2021/8816456 (PMC7817270; doi:10.1155/2021/8816456)
Supplement: Supplementary materials — Table S1: the detailed information on Venn diagrams related to upregulated DEGs. Table S2: the detailed information on Venn diagrams related to downregulated DEGs. Table S3: the detailed statistical information related to GO analysis. Table S4: the detailed statistical information related to REACTOME analysis. Table S5: the detailed statistical information related to PANTHER analysis. Table S6: the detailed statistical information related to KEGG analysis. Table S7: the detailed information about the Venn diagram between OS-related genes and DEGs. Table S8: the detailed information about the Venn diagram between OS-related genes and Top 40 hub genes. [file 8816456.f1.pdf]

Table S1

GSE3218 UP-DEGs

	logFC	AveExpr	t	P.Value	adj.P.Val	B
PTPN6	2.899634	8.841773	10.47077	3.95E-18	3.36E-17	30.33059
GSTO1	2.069213	10.22676	8.481514	1.28E-13	6.78E-13	19.99726
TNFRSF1B	2.191372	7.875343	7.222055	7.57E-11	2.99E-10	13.66913
LYZ	4.40737	9.575078	6.851431	4.68E-10	1.70E-09	11.86936
GIMAP4	2.575582	7.936951	6.718353	8.94E-10	3.14E-09	11.23207
DSP	3.127381	9.840683	5.875985	4.73E-08	1.38E-07	7.328812
ID3	2.40759	8.385845	5.611722	1.56E-07	4.29E-07	6.158184
CLEC2B	2.28262	7.201044	5.214743	8.94E-07	2.25E-06	4.456451
CXCR4	2.505302	9.401293	5.203745	9.37E-07	2.35E-06	4.41034
EFEMP1	2.791673	8.056005	5.146078	1.20E-06	2.98E-06	4.169539
COL4A1	2.136587	9.869108	5.045385	1.84E-06	4.46E-06	3.752992
CRIP1	2.209437	8.418037	5.023176	2.02E-06	4.88E-06	3.661799
LAPTM5	2.447935	9.885282	4.887061	3.58E-06	8.36E-06	3.108414
MMP9	2.853058	10.05481	4.831425	4.50E-06	1.04E-05	2.885001
BASP1	2.331306	10.07127	4.749771	6.30E-06	1.43E-05	2.560109
NFE2L3	2.04723	7.297341	4.700537	7.70E-06	1.73E-05	2.365961
CORO1A	2.321112	7.994298	4.606747	1.12E-05	2.47E-05	1.999825
CCL19	3.163703	8.5001	4.36491	2.92E-05	6.11E-05	1.078946
HLA-DRB1	2.070638	11.55822	4.355366	3.03E-05	6.32E-05	1.043309
TYROBP	2.343191	8.678314	4.326146	3.40E-05	7.03E-05	0.93454
C1QA	2.573849	9.696292	4.321535	3.46E-05	7.15E-05	0.917425
ISG15	2.448932	9.701545	4.30024	3.75E-05	7.73E-05	0.838542
CD9	2.420087	10.95204	4.29315	3.86E-05	7.94E-05	0.812341
KRT8	2.801219	9.362724	4.255454	4.46E-05	9.10E-05	0.673542
COL3A1	2.865768	10.90275	4.243282	4.67E-05	9.50E-05	0.628913
CDH1	2.05525	7.086546	4.193025	5.66E-05	0.000114	0.445603
C1QB	2.423139	8.815061	4.17265	6.11E-05	0.000122	0.371735
CD74	2.277603	11.57639	4.055186	9.48E-05	0.000185	-0.04902
TGFBI	2.118771	9.716294	4.012553	0.000111	0.000215	-0.19955
ARHGDIB	2.017181	9.605089	3.998844	0.000117	0.000225	-0.2477
LUM	3.036576	9.076593	3.965671	0.000132	0.000252	-0.36372
HLA-DRA	2.676211	10.37743	3.938811	0.000145	0.000277	-0.45713
FZD5	2.072476	7.736783	3.908055	0.000163	0.000308	-0.56351
CD24	2.484677	8.700762	3.846955	0.000203	0.00038	-0.77297
IER3	2.002148	9.72681	3.779697	0.000258	0.000476	-1.00064
ANXA1	2.350583	9.24707	3.647495	0.000409	0.000736	-1.43912
IGJ	2.756591	7.464785	3.645847	0.000412	0.00074	-1.44451
COL5A2	2.204503	8.77171	3.550809	0.00057	0.001006	-1.75208
CCL2	2.099359	8.749958	3.550341	0.000571	0.001007	-1.75358
GBP1	2.085364	7.676152	3.533883	0.000604	0.001062	-1.80619
LY96	2.071412	7.296311	3.519403	0.000634	0.001112	-1.85231
VCAN	2.105023	8.667108	3.398051	0.000951	0.001624	-2.23291
RAB25	2.048519	7.313901	3.301617	0.001304	0.002185	-2.52767
MMP12	2.197765	7.59177	3.157813	0.002061	0.003358	-2.95427
ADAMDEC1	2.122861	7.383283	3.137978	0.002193	0.003558	-3.01188
SPP1	2.304872	9.292198	2.972414	0.003644	0.005747	-3.48085

LIN28A	2.565314	8.567552	2.913406	0.004346	0.006784	-3.64278
POU5F1P3	2.305926	8.077748	2.774057	0.006524	0.009933	-4.01413
KRT19	2.407296	8.019048	2.468472	0.015137	0.021824	-4.77262

		TCGA UP-DEGs				Venn gene
A4GALT	ENSG0000C	45.15	8.44	2.289	8.68E-25	PTPN6
ABHD12B	ENSG0000C	18.191	0.86	3.367	4.95E-31	GSTO1
AC004057.	ENSG0000C	971.115	224.846	2.106	5.55E-24	TNFRSF1B
AC005255.	ENSG0000C	7.65	0.81	2.257	5.00E-39	LYZ
AC005339.	ENSG0000C	6.49	0	2.905	2.48E-43	GIMAP4
AC005682.	ENSG0000C	14.68	1.02	2.957	8.74E-52	DSP
AC005943.	ENSG0000C	7.27	0	3.048	7.34E-18	CXCR4
AC005943.	ENSG0000C	19.271	0	4.341	1.80E-12	EFEMP1
AC008753.	ENSG0000C	44.86	0.26	5.186	1.92E-61	COL4A1
AC009065.	ENSG0000C	11.37	0	3.629	2.81E-73	CRIP1
AC010677.	ENSG0000C	24	1.67	3.227	6.40E-11	LAPTM5
AC011558.	ENSG0000C	68.46	9.99	2.66	6.00E-42	MMP9
AC021066.	ENSG0000C	9.5	0	3.392	4.24E-08	BASP1
AC092580.	ENSG0000C	5.96	0	2.799	5.36E-39	NFE2L3
AC104534.	ENSG0000C	17.49	2.44	2.426	1.39E-10	CORO1A
AC133644.	ENSG0000C	3.77	0.07	2.156	6.01E-50	CCL19
ACP5	ENSG0000C	67.06	3.9	3.796	6.07E-61	HLA-DRB1
ACTB	ENSG0000C	3840.556	832.38	2.205	9.87E-64	TYROBP
ACTG2	ENSG0000C	32.36	5.7	2.316	3.05E-13	C1QA
ADAMDEC1	ENSG0000C	12.14	0.22	3.429	2.56E-39	ISG15
ADD2	ENSG0000C	25.5	5.18	2.1	3.56E-20	CD9
ADGRE5	ENSG0000C	26.241	4.5	2.308	1.14E-41	KRT8
AEN	ENSG0000C	80.979	14.46	2.407	1.97E-39	CDH1
AIF1	ENSG0000C	55.491	3.41	3.679	3.68E-53	C1QB
AK4	ENSG0000C	88.101	9.58	3.074	2.63E-38	CD74
AK4P1	ENSG0000C	14.87	1.18	2.864	3.07E-29	TGFBI
AL162151.	ENSG0000C	1067.928	88.781	3.574	6.66E-07	ARHGDIB
ALPL	ENSG0000C	262.086	7.74	4.912	5.07E-59	LUM
ALPP	ENSG0000C	4.16	0.15	2.166	8.46E-22	HLA-DRA
ALPPL2	ENSG0000C	57.579	0.35	5.439	1.37E-42	FZD5
AMICA1	ENSG0000C	9.72	1.41	2.153	1.18E-30	CD24
ANP32A	ENSG0000C	162.005	24.58	2.672	7.21E-68	CCL2
ANP32E	ENSG0000C	45.72	6.73	2.596	1.39E-70	GBP1
ANXA3	ENSG0000C	21.66	1.84	2.996	3.45E-56	RAB25
AOAH	ENSG0000C	9.6	0.96	2.435	1.03E-41	MMP12
AP003391.	ENSG0000C	13.57	0.07	3.767	1.95E-04	ADAMDEC1
APEX1	ENSG0000C	226.128	33.7	2.711	4.49E-85	SPP1
APLNR	ENSG0000C	6.42	0.76	2.076	6.93E-24	LIN28A
APOC1	ENSG0000C	655.157	21.22	4.884	4.45E-64	KRT19
APOC1P1	ENSG0000C	10.52	0.4	3.041	7.98E-43	
APOE	ENSG0000C	1146.403	155.664	2.873	1.51E-46	
ARHGDIB	ENSG0000C	126.517	14.9	3.004	2.18E-50	
ARL4C	ENSG0000C	9.99	0.92	2.517	3.00E-60	
ARPC1B	ENSG0000C	256.87	34.53	2.86	3.82E-57	
ARRB1	ENSG0000C	17.289	3.27	2.099	7.16E-33	
ARSE	ENSG0000C	5	0.37	2.131	6.80E-17	
ATP2A3	ENSG0000C	22.77	4.55	2.099	7.00E-34	

AURKB	ENSG0000C	49.309	1.9	4.117	2.19E-90
B2M	ENSG0000C	3093.447	450.879	2.776	3.95E-34
B3GNT7	ENSG0000C	8.51	1.23	2.092	9.03E-40
BASP1	ENSG0000C	25.76	1.55	3.391	1.75E-72
BATF	ENSG0000C	9.3	0.54	2.742	1.68E-50
BATF3	ENSG0000C	10.71	1.72	2.106	2.59E-43
BAX	ENSG0000C	235.796	38.019	2.601	4.98E-62
BCL2A1	ENSG0000C	7.65	0.37	2.658	1.16E-35
BFSP2	ENSG0000C	4.72	0.19	2.265	4.86E-34
BICD1	ENSG0000C	57.249	5.07	3.262	5.63E-59
BMF	ENSG0000C	21	2.22	2.772	1.91E-33
BNIP3P17	ENSG0000C	17.93	0.41	3.747	6.23E-45
C12orf57	ENSG0000C	468.075	108.623	2.097	1.38E-31
C19orf33	ENSG0000C	14.19	2.09	2.297	4.33E-26
C1QA	ENSG0000C	226.724	7.66	4.717	7.02E-50
C1QB	ENSG0000C	226.347	10.52	4.303	1.95E-41
C1QC	ENSG0000C	168.639	7.22	4.367	1.79E-47
C2	ENSG0000C	25.91	3.64	2.536	1.13E-34
C6orf132	ENSG0000C	8.98	0.89	2.401	4.11E-33
CAPG	ENSG0000C	213.426	35.589	2.551	2.77E-48
CAPN13	ENSG0000C	6.28	0.7	2.098	1.23E-20
CARD16	ENSG0000C	15.68	2.13	2.414	1.66E-41
CASC9	ENSG0000C	4.35	0.17	2.193	4.44E-51
CASP1	ENSG0000C	13.32	1.89	2.309	1.17E-36
CBR3	ENSG0000C	20.381	3.7	2.186	1.92E-29
CCL18	ENSG0000C	10.83	1.52	2.231	1.47E-23
CCL19	ENSG0000C	60.078	0.42	5.427	2.24E-45
CCL2	ENSG0000C	46.52	4.1	3.22	4.37E-26
CCL26	ENSG0000C	14.45	1.05	2.914	8.57E-19
CCL3	ENSG0000C	20.81	0.72	3.665	4.46E-46
CCL4	ENSG0000C	19.569	2.97	2.373	3.77E-24
CCL4L2	ENSG0000C	8.94	0.27	2.968	3.98E-39
CCL5	ENSG0000C	55.73	0.77	5.002	7.77E-62
CCND2	ENSG0000C	75.992	4.92	3.701	8.83E-61
CCR1	ENSG0000C	6.07	0.24	2.511	1.87E-49
CCR5	ENSG0000C	5.88	0.09	2.658	1.10E-45
CCT6A	ENSG0000C	399.983	92.027	2.108	2.12E-60
CD14	ENSG0000C	41.14	7.48	2.313	3.03E-19
CD2	ENSG0000C	18.4	1.33	3.058	1.40E-36
CD24	ENSG0000C	36.319	4.54	2.752	2.25E-13
CD247	ENSG0000C	7.23	0.76	2.225	7.66E-36
CD27	ENSG0000C	14.78	0.3	3.602	2.46E-53
CD300A	ENSG0000C	9.55	1.13	2.308	1.37E-49
CD38	ENSG0000C	12.21	0.62	3.028	4.20E-32
CD3D	ENSG0000C	23.309	0.72	3.821	1.84E-51
CD3E	ENSG0000C	23	0.79	3.745	2.82E-48
CD3EAP	ENSG0000C	13.13	2.32	2.09	4.43E-40
CD4	ENSG0000C	27.57	3.2	2.766	4.19E-39
CD44	ENSG0000C	32.751	6.1	2.249	4.90E-33

CD48	ENSG0000C	24.97	2.48	2.9	3.02E-41
CD52	ENSG0000C	105.018	1.32	5.514	1.23E-80
CD53	ENSG0000C	49.921	2.95	3.688	2.77E-53
CD68	ENSG0000C	87.997	0.92	5.535	5.27E-60
CD7	ENSG0000C	14.91	0.87	3.089	5.32E-55
CD74	ENSG0000C	1649.255	105.141	3.959	1.44E-49
CD79A	ENSG0000C	17.47	0.48	3.642	3.50E-42
CD8A	ENSG0000C	8.92	0.47	2.755	2.51E-36
CD8B	ENSG0000C	5.17	0.21	2.35	1.24E-44
CD9	ENSG0000C	483.709	26.85	4.121	7.85E-59
CDA	ENSG0000C	11.51	1.66	2.234	5.85E-34
CDCA7	ENSG0000C	40.051	6.03	2.546	2.54E-48
CDH1	ENSG0000C	21.48	0.58	3.831	9.14E-39
CDH3	ENSG0000C	57.42	3.35	3.747	1.33E-81
CDK4	ENSG0000C	184.412	33.73	2.416	7.93E-67
CDT1	ENSG0000C	48.701	5.95	2.838	2.71E-53
CDX1	ENSG0000C	24.07	3.07	2.623	6.03E-16
CEACAM6	ENSG0000C	4.44	0.16	2.229	2.28E-24
CEBPA	ENSG0000C	11.78	1.25	2.506	1.46E-46
CENPA	ENSG0000C	51.211	3.29	3.605	3.55E-70
CGB8	ENSG0000C	4.14	0	2.362	3.26E-18
CH507-513	ENSG0000C	6.06	0.38	2.355	1.13E-26
CH507-528	ENSG0000C	6.06	0.38	2.355	1.13E-26
CH507-9B2	ENSG0000C	3.9	0.04	2.236	1.41E-14
CHEK2	ENSG0000C	49.121	10.64	2.106	2.62E-35
CHI3L1	ENSG0000C	21.95	0.7	3.755	3.87E-21
CHIT1	ENSG0000C	5.16	0.22	2.336	3.43E-17
CHST2	ENSG0000C	23.55	2.3	2.895	2.11E-43
CKMT1B	ENSG0000C	7.12	0.8	2.173	2.46E-30
CKS1BP3	ENSG0000C	20.74	0.88	3.532	8.90E-49
CLDN6	ENSG0000C	159.885	0.83	6.458	########
CLDN7	ENSG0000C	30.84	3.15	2.94	2.17E-44
CLEC4D	ENSG0000C	10.14	0.1	3.34	2.52E-36
CNN2	ENSG0000C	78.732	15.401	2.281	3.53E-55
COL4A1	ENSG0000C	50.98	10.6	2.164	2.63E-33
CORO1A	ENSG0000C	72.238	3.37	4.067	6.14E-63
COX7B	ENSG0000C	129.167	22.751	2.454	6.63E-57
CPXM1	ENSG0000C	31.48	3.66	2.801	2.39E-51
CRABP1	ENSG0000C	23.74	0.78	3.797	6.40E-38
CRB3	ENSG0000C	6.87	0.53	2.363	2.63E-21
CRIP1	ENSG0000C	332.059	17.93	4.137	3.15E-69
CRYGEP	ENSG0000C	11.39	0.12	3.468	1.76E-37
CSF2RA	ENSG0000C	6.18	0.73	2.053	1.68E-38
CST7	ENSG0000C	12.89	0.58	3.136	1.85E-42
CTB-151G2	ENSG0000C	9.74	0.53	2.811	6.35E-27
CTB-151G2	ENSG0000C	12.98	1.57	2.443	1.37E-16
CTB-63M22	ENSG0000C	103.349	12.59	2.941	1.18E-43
CTC-260E6	ENSG0000C	23.489	1.13	3.523	5.29E-30
CTC-260E6	ENSG0000C	35.409	0.77	4.362	3.11E-55

CTC-260E6	ENSG0000C	13.45	0.87	2.95	7.05E-32
CTD-2196E	ENSG0000C	6.55	0.59	2.247	5.78E-05
CTD-2354A	ENSG0000C	6.93	0.45	2.451	6.61E-23
CTD-2396E	ENSG0000C	6.39	0.67	2.146	1.26E-19
CTD-2510F	ENSG0000C	6.36	0.3	2.501	1.64E-53
CTD-2540E	ENSG0000C	19	1.2	3.184	1.12E-58
CTHRC1	ENSG0000C	13.71	2.3	2.156	1.32E-32
CTSB	ENSG0000C	367.881	49.401	2.872	1.66E-52
CTSC	ENSG0000C	130.788	14.11	3.125	2.05E-55
CTSS	ENSG0000C	62.808	2.23	4.304	5.06E-47
CTSZ	ENSG0000C	145.199	32.549	2.124	2.25E-31
CXCL10	ENSG0000C	31.811	0.23	4.737	1.00E-62
CXCL11	ENSG0000C	6.04	0.11	2.665	1.41E-38
CXCL13	ENSG0000C	8.07	0.79	2.341	2.01E-18
CXCL9	ENSG0000C	50.86	1.96	4.131	8.06E-37
CXCR2P1	ENSG0000C	4.49	0.07	2.359	1.54E-35
CXCR3	ENSG0000C	6.44	0.24	2.585	5.69E-39
CXCR4	ENSG0000C	48.62	4.07	3.291	1.74E-56
CXCR6	ENSG0000C	5.42	0.18	2.444	1.68E-34
CYBA	ENSG0000C	153.393	9.89	3.826	3.92E-77
CYBB	ENSG0000C	11.89	0.6	3.01	5.66E-43
CYP2S1	ENSG0000C	73.059	1	5.211	2.16E-74
DACT2	ENSG0000C	8.33	1.03	2.2	6.44E-25
DEFB1	ENSG0000C	5.19	0.38	2.165	9.51E-22
DENND1C	ENSG0000C	11.29	1.91	2.078	5.31E-69
DERA	ENSG0000C	58.871	8.65	2.633	4.79E-68
DLG3	ENSG0000C	45.87	2.56	3.719	4.02E-82
DMD	ENSG0000C	32.17	6.01	2.242	3.59E-40
DNMT3L	ENSG0000C	16.72	0.27	3.802	3.60E-22
DPPA3	ENSG0000C	168.709	0.57	6.756	5.45E-50
DPPA3P2	ENSG0000C	11.7	0.18	3.428	1.26E-28
DPPA4	ENSG0000C	104.112	4.68	4.21	1.14E-33
DPPA5	ENSG0000C	377.438	1.5	7.242	5.87E-36
DSC2	ENSG0000C	5.7	0.45	2.208	5.54E-53
DSCC1	ENSG0000C	14.23	2.4	2.163	5.66E-53
DSG2	ENSG0000C	15.38	1.59	2.661	1.42E-52
DSP	ENSG0000C	35.631	1.81	3.704	4.56E-77
DSTNP2	ENSG0000C	43.251	5.19	2.838	1.16E-51
EEF1A1	ENSG0000C	9013.051	1767.996	2.349	2.86E-75
EEF1A1P12	ENSG0000C	4.15	0.17	2.138	3.46E-63
EEF1A1P5	ENSG0000C	200.546	24.17	3.001	6.99E-38
EEF1A1P6	ENSG0000C	21.96	3.16	2.464	2.68E-51
EFEMP1	ENSG0000C	46.339	3.14	3.515	9.92E-31
EFNA4	ENSG0000C	20.29	3.17	2.352	3.00E-76
EIF3K	ENSG0000C	552.909	97.673	2.489	7.76E-70
EIF4EBP1	ENSG0000C	83.227	14.21	2.469	9.81E-77
ELF3	ENSG0000C	24.319	0.97	3.684	5.47E-39
ELOVL6	ENSG0000C	23.07	0.83	3.717	1.24E-65
EMG1	ENSG0000C	102.7	13.72	2.817	1.10E-81

ENO2	ENSG0000C	94.549	14.17	2.655	1.30E-43
TMEM176A	ENSG0000C	31.2	5.81	2.241	1.19E-17
RECQL	ENSG0000C	26.629	5.57	2.072	7.79E-36
NDUFAB1	ENSG0000C	354.366	83.141	2.078	2.14E-32
ITGAL	ENSG0000C	12.63	1.51	2.441	9.57E-35
PLEKHG6	ENSG0000C	11.83	1.32	2.467	1.26E-18
MGST1	ENSG0000C	71.461	13.18	2.353	1.43E-13
IL32	ENSG0000C	182.265	16.21	3.413	1.00E-41
NME2	ENSG0000C	34.441	4.06	2.808	6.82E-33
TSPAN9	ENSG0000C	54.059	6.68	2.842	9.41E-54
TYROBP	ENSG0000C	137.615	9.4	3.736	2.77E-54
GPRC5A	ENSG0000C	40.14	1.2	4.225	1.65E-35
NLRP2	ENSG0000C	147.155	23.18	2.615	6.59E-23
TYMP	ENSG0000C	52.691	5.89	2.962	3.72E-35
SLAMF7	ENSG0000C	19.311	0.09	4.22	1.49E-49
SH2D2A	ENSG0000C	7.32	0.64	2.343	2.56E-64
TNFRSF1B	ENSG0000C	18.1	2.79	2.333	1.12E-38
NSMAF	ENSG0000C	61.239	11.31	2.338	1.37E-36
LCP2	ENSG0000C	15.67	3.02	2.052	4.08E-27
NFE2L3	ENSG0000C	85.247	3.59	4.232	6.10E-72
PRSS8	ENSG0000C	16.52	0.84	3.251	2.92E-35
NNAT	ENSG0000C	27.959	6.13	2.022	2.40E-15
TRAF3IP2	ENSG0000C	19.73	4.04	2.04	8.94E-45
GYG2	ENSG0000C	10.38	1.28	2.319	8.97E-55
PRDM1	ENSG0000C	15.95	1.09	3.02	4.71E-42
SLC2A3	ENSG0000C	74.63	13.81	2.352	3.66E-22
MRPS35	ENSG0000C	80.392	17.259	2.156	1.76E-41
RPL18	ENSG0000C	2780.058	628.905	2.142	3.66E-65
ERBB3	ENSG0000C	21.17	2.84	2.529	2.44E-44
NGEF	ENSG0000C	30.759	5.22	2.352	6.61E-18
SPI1	ENSG0000C	27.029	4.16	2.442	7.96E-36
PRR11	ENSG0000C	13.43	1.23	2.694	2.29E-62
OSBPL3	ENSG0000C	18.4	3.17	2.218	2.91E-26
RPL31	ENSG0000C	4174.535	1016.855	2.036	2.73E-67
HSD17B10	ENSG0000C	149.872	29.709	2.297	1.59E-63
ST6GAL1	ENSG0000C	45.379	2.04	3.931	9.22E-68
TPD52	ENSG0000C	47.221	7.12	2.57	2.25E-52
RARB	ENSG0000C	6.58	0.49	2.347	1.50E-38
NFKB2	ENSG0000C	69.748	16.22	2.039	2.07E-31
FBLN1	ENSG0000C	155.158	26.17	2.523	2.71E-38
PTPRC	ENSG0000C	15.43	1.64	2.638	4.36E-30
PLOD1	ENSG0000C	112.407	15.37	2.792	6.85E-72
GSTP1	ENSG0000C	634.729	62.439	3.325	2.51E-69
FCN1	ENSG0000C	8.34	0.24	2.913	2.69E-46
SNX10	ENSG0000C	22.99	2.02	2.99	1.90E-43
FTL	ENSG0000C	3055.514	441.386	2.789	2.71E-49
TFAP2C	ENSG0000C	63.99	0.62	5.326	1.52E-58
RPL6	ENSG0000C	2031.316	299.31	2.759	7.15E-96
RBBP9	ENSG0000C	16.16	3	2.101	4.93E-44

OAS1	ENSG0000C	23.41	4.01	2.285	2.40E-29
RPLP0	ENSG0000C	3900.652	583.504	2.739	2.23E-90
FXYD5	ENSG0000C	115.551	11.36	3.237	5.34E-63
LYZ	ENSG0000C	176.264	2.43	5.692	4.30E-53
TFAP4	ENSG0000C	17.04	2.67	2.297	6.40E-62
MSH2	ENSG0000C	75.719	17.21	2.075	1.08E-36
TREM2	ENSG0000C	7	0.27	2.655	3.35E-38
SUSD2	ENSG0000C	77.918	5.69	3.56	5.61E-25
PRODH	ENSG0000C	90.183	4.85	3.962	9.01E-48
LGALS2	ENSG0000C	17.98	0.16	4.032	1.22E-53
XBP1	ENSG0000C	149.862	21.529	2.743	7.83E-73
MCM5	ENSG0000C	118.742	28.111	2.04	1.72E-45
IL2RB	ENSG0000C	7.67	0.76	2.3	8.87E-36
GZMH	ENSG0000C	6.49	0.25	2.583	8.10E-40
GZMB	ENSG0000C	20.569	0.27	4.086	1.03E-46
PYGL	ENSG0000C	44.569	7.29	2.459	5.15E-38
SPTLC2	ENSG0000C	24.68	4.63	2.189	1.67E-54
MTHFD1	ENSG0000C	67.451	10.41	2.585	4.37E-70
TCL1A	ENSG0000C	77.799	10.62	2.762	8.18E-06
MMP9	ENSG0000C	92.25	1.11	5.466	1.27E-67
MYBL2	ENSG0000C	359.387	20.629	4.058	3.75E-52
SALL4	ENSG0000C	43.901	6.65	2.553	4.49E-38
TRIB3	ENSG0000C	12	1.88	2.174	6.87E-57
HCK	ENSG0000C	12.75	1.78	2.306	2.47E-36
RENBP	ENSG0000C	15	2.07	2.382	5.02E-34
PIM2	ENSG0000C	235.583	11.17	4.281	2.59E-45
PQBP1	ENSG0000C	154.964	30.391	2.313	2.44E-67
PGK1	ENSG0000C	363.295	62.292	2.525	1.06E-38
FGD1	ENSG0000C	22.68	3.27	2.471	1.27E-54
PIN4	ENSG0000C	43.901	7.8	2.351	5.63E-72
RBM3	ENSG0000C	386.787	88.07	2.122	2.14E-42
QPRT	ENSG0000C	57.759	3.32	3.766	1.32E-76
PYCARD	ENSG0000C	36.031	2.96	3.225	1.76E-63
LAPTM4B	ENSG0000C	254.866	47.33	2.404	1.20E-36
ESRP1	ENSG0000C	67.681	1.4	4.839	1.66E-95
MAN2B1	ENSG0000C	103.028	17.21	2.514	6.38E-70
MAP4K1	ENSG0000C	39.17	3.61	3.123	7.14E-45
TNNT1	ENSG0000C	85.318	15.79	2.362	2.34E-33
RPS16	ENSG0000C	2964.554	626.121	2.241	3.18E-73
SHD	ENSG0000C	14.95	0.68	3.247	7.79E-38
LIM2	ENSG0000C	15.88	0.19	3.826	2.77E-35
RPS19	ENSG0000C	5665.548	847.458	2.74	2.28E-93
NKG7	ENSG0000C	36.829	0.91	4.308	1.20E-53
RPL18A	ENSG0000C	2592.448	471.985	2.455	2.80E-68
ETHE1	ENSG0000C	39.94	6.5	2.449	3.44E-55
TSPAN13	ENSG0000C	36.171	5.97	2.415	7.89E-56
TMEM176B	ENSG0000C	56.712	12.04	2.146	8.83E-18
PDLIM1	ENSG0000C	99.532	19.99	2.26	6.80E-55
RPL28	ENSG0000C	3450.524	783.722	2.137	1.13E-79

ZMIZ1	ENSG0000C	37.909	6.45	2.385	6.90E-38
RPL19	ENSG0000C	3488.762	823.713	2.081	9.31E-92
PFN1	ENSG0000C	987.611	138.668	2.823	8.33E-86
UNC93B1	ENSG0000C	17.91	2.65	2.373	4.59E-53
MS4A6A	ENSG0000C	35.05	5.15	2.551	1.12E-24
FOLR1	ENSG0000C	6.09	0.37	2.372	1.09E-26
IL10RA	ENSG0000C	9.93	1.28	2.261	1.28E-33
SLC15A3	ENSG0000C	46.898	10.42	2.068	3.93E-33
MDK	ENSG0000C	425.612	25.141	4.029	5.27E-58
KRT18	ENSG0000C	91.829	10	3.077	2.90E-12
PPM1H	ENSG0000C	15.47	1.39	2.785	3.54E-52
WNT5B	ENSG0000C	17.8	2.65	2.365	2.03E-24
MAGOHB	ENSG0000C	82.002	11.73	2.705	3.39E-79
OAS2	ENSG0000C	13	1.58	2.44	1.41E-39
FZD10	ENSG0000C	3.9	0.22	2.006	4.03E-40
GAPDH	ENSG0000C	6497.188	988.981	2.715	7.32E-70
PTPN6	ENSG0000C	59.672	9.35	2.551	2.56E-70
NANOG	ENSG0000C	223.572	0.35	7.378	7.67E-54
GOLT1B	ENSG0000C	40.901	9.35	2.017	5.72E-39
LDHB	ENSG0000C	1302.167	144.246	3.165	5.24E-61
MDFI	ENSG0000C	78.912	6.57	3.4	2.73E-59
GZMK	ENSG0000C	9.36	0.3	2.994	7.84E-38
IRX4	ENSG0000C	6.89	0.85	2.093	7.71E-20
RRP9	ENSG0000C	63.131	14.45	2.053	5.08E-50
TFCP2L1	ENSG0000C	18.56	1.38	3.039	7.00E-33
FN1	ENSG0000C	185.708	38.419	2.244	7.25E-19
GNLY	ENSG0000C	20.21	0.73	3.616	3.11E-44
HSPE1	ENSG0000C	414.491	66.04	2.632	3.02E-67
PLEK	ENSG0000C	15.67	1.37	2.814	5.34E-37
RPL22	ENSG0000C	1011.232	169.882	2.566	1.34E-79
SRM	ENSG0000C	250.713	52.261	2.241	4.37E-60
OLFML3	ENSG0000C	26.33	5.32	2.112	9.82E-20
MYCL	ENSG0000C	18.74	1.57	2.941	1.14E-50
PLA2G2D	ENSG0000C	10.08	0.06	3.386	5.91E-26
GBP1	ENSG0000C	22.68	4.8	2.03	2.86E-23
SLC2A1	ENSG0000C	66.132	9.93	2.619	9.66E-31
PRDX1	ENSG0000C	904.263	163.233	2.463	2.82E-57
MAN1C1	ENSG0000C	56.102	6.04	3.02	4.02E-37
SPP1	ENSG0000C	37.569	5.55	2.558	1.80E-11
SET	ENSG0000C	391.725	79.621	2.284	1.02E-49
FKBP1B	ENSG0000C	17.33	3.24	2.112	1.16E-57
EPCAM	ENSG0000C	137.911	21.17	2.647	8.87E-40
TGFBI	ENSG0000C	39.719	8.01	2.176	2.82E-27
PLBD1	ENSG0000C	139.729	12.34	3.399	6.64E-51
KCNJ8	ENSG0000C	8.3	1.1	2.147	6.50E-31
TNFSF10	ENSG0000C	16.09	2.36	2.347	5.34E-30
SASH3	ENSG0000C	10.92	0.54	2.952	1.52E-47
GLIPR2	ENSG0000C	45.61	3.95	3.235	2.61E-50
PLAU	ENSG0000C	22.52	0.98	3.57	7.91E-62

SLC25A16	ENSG0000C	33.13	3.61	2.888	9.45E-38
FAM124B	ENSG0000C	5.11	0.18	2.372	1.57E-30
RPS10	ENSG0000C	3233.094	744.949	2.116	1.46E-71
SOX4	ENSG0000C	51.41	7.78	2.578	3.11E-71
MT1G	ENSG0000C	227.149	3.43	5.687	7.55E-37
RPL23	ENSG0000C	2208.727	422.204	2.384	4.76E-70
RTN2	ENSG0000C	19.83	3.29	2.28	8.30E-62
MMP24	ENSG0000C	13.47	0.45	3.319	7.23E-45
HCST	ENSG0000C	45.141	3.89	3.238	1.07E-48
IFI6	ENSG0000C	298.999	70.629	2.066	4.78E-15
PTPN12	ENSG0000C	116.872	23.76	2.251	1.70E-43
SRD5A3	ENSG0000C	24.2	4.5	2.196	3.19E-42
YWHAH	ENSG0000C	92.097	21.75	2.033	7.70E-49
RAC2	ENSG0000C	43.971	2.31	3.764	2.17E-56
PODXL	ENSG0000C	33.569	4.14	2.75	3.93E-30
SOX15	ENSG0000C	85.989	3.45	4.289	2.21E-44
FAM64A	ENSG0000C	11.87	0.6	3.008	3.08E-53
FGF13	ENSG0000C	15.71	2.7	2.175	3.94E-38
HDHD1	ENSG0000C	25.351	4.25	2.327	1.61E-65
STARD8	ENSG0000C	7.11	1.02	2.005	1.39E-35
GDPD2	ENSG0000C	6.9	0.07	2.884	7.04E-53
ZSCAN10	ENSG0000C	10.89	0.31	3.182	8.78E-32
RPL36	ENSG0000C	2127.14	401.316	2.403	8.63E-80
LSP1	ENSG0000C	54.879	11.99	2.105	9.86E-26
NPAS1	ENSG0000C	7.79	1.1	2.065	2.15E-31
LILRB2	ENSG0000C	9.29	0.64	2.649	2.40E-46
PYY	ENSG0000C	17.96	1.45	2.952	2.14E-27
IDO1	ENSG0000C	9.75	0.43	2.91	1.33E-41
RPL27	ENSG0000C	3182.4	548.367	2.535	9.66E-80
LIN28A	ENSG0000C	75.081	6.53	3.337	2.80E-27
JCHAIN	ENSG0000C	74.962	0.71	5.473	7.30E-55
RAB25	ENSG0000C	10.7	0.97	2.57	8.72E-36
TPT1	ENSG0000C	4412.864	931.169	2.243	1.73E-64
RARRES3	ENSG0000C	36.671	5.28	2.585	3.64E-24
GIMAP4	ENSG0000C	21.69	2.57	2.668	1.72E-37
VRTN	ENSG0000C	17.62	2.18	2.55	3.26E-36
LOXL2	ENSG0000C	46.211	3.3	3.457	1.46E-64
FKBP11	ENSG0000C	71.5	16.54	2.047	4.92E-57
MYCN	ENSG0000C	51.94	3.28	3.629	2.34E-58
RPS15A	ENSG0000C	2789.517	632.27	2.14	3.06E-75
LRP4	ENSG0000C	34.35	3.16	3.087	5.42E-60
HNRNPA1	ENSG0000C	1883.629	335.762	2.484	2.94E-80
ITM2C	ENSG0000C	334.021	27.521	3.554	8.21E-65
GPNMB	ENSG0000C	73.602	14.26	2.289	5.73E-30
MYO1G	ENSG0000C	7.14	0.88	2.114	2.94E-42
GYPC	ENSG0000C	50.011	6.96	2.68	5.02E-44
TXN	ENSG0000C	303.341	32.17	3.198	1.85E-67
SIT1	ENSG0000C	5.46	0.11	2.541	6.19E-52
PIM1	ENSG0000C	91.938	13.66	2.664	5.29E-57

TUBB2B	ENSG0000C	40.059	1.47	4.055	1.42E-60
HMGA1	ENSG0000C	491.312	107.061	2.188	7.61E-24
TCF19	ENSG0000C	14.58	2.63	2.102	2.79E-63
MRPL15	ENSG0000C	126.158	14.32	3.053	3.56E-75
SDCBP	ENSG0000C	148.621	32.561	2.156	4.08E-44
RPLP1	ENSG0000C	4014.764	609.549	2.717	1.69E-86
RPL7P9	ENSG0000C	85.65	9.72	3.015	1.92E-61
RPS24	ENSG0000C	5482.43	1042.187	2.394	3.21E-84
TET1	ENSG0000C	9.99	0.2	3.195	4.35E-70
YARS2	ENSG0000C	49.531	11.27	2.042	7.90E-65
FAM60A	ENSG0000C	114.61	20.599	2.42	3.49E-34
RPL14P1	ENSG0000C	19.929	2.99	2.391	7.81E-61
LUM	ENSG0000C	67.859	14.74	2.129	6.51E-12
NPFF	ENSG0000C	5.01	0.01	2.573	4.85E-27
ITGB7	ENSG0000C	5.9	0.71	2.013	7.97E-33
GALNT6	ENSG0000C	18.92	0.76	3.501	5.88E-77
RAB15	ENSG0000C	109.348	2.74	4.883	1.89E-75
ZNF710	ENSG0000C	15.46	2.49	2.238	6.94E-74
ITGAX	ENSG0000C	11.06	0.96	2.621	1.38E-36
IGSF6	ENSG0000C	8.54	0.32	2.853	2.62E-40
RPS2	ENSG0000C	4142.826	594.362	2.799	8.30E-96
PMAIP1	ENSG0000C	17.8	1.76	2.768	1.47E-46
GRB7	ENSG0000C	20.5	1.95	2.866	1.02E-37
FBN3	ENSG0000C	11.44	1.12	2.553	1.57E-35
RPL13A	ENSG0000C	4152.889	820.75	2.338	3.70E-69
SH3BGRL3	ENSG0000C	130.843	29.881	2.094	1.88E-52
RPL11	ENSG0000C	3470.192	610.225	2.506	7.50E-91
RPS8	ENSG0000C	2840.442	468.173	2.598	1.63E-77
XCL2	ENSG0000C	3.16	0	2.057	1.79E-40
RPS27A	ENSG0000C	2258.579	561.794	2.005	7.94E-49
HSPD1	ENSG0000C	393.575	73.991	2.396	1.26E-54
RP11-977G	ENSG0000C	7.61	0.7	2.34	6.86E-17
PLAC8	ENSG0000C	6.21	0.63	2.145	2.65E-33
SFRP2	ENSG0000C	47.811	1.14	4.512	1.91E-54
RPL37	ENSG0000C	2954.503	548.177	2.428	6.19E-91
SKP2	ENSG0000C	14.55	2.88	2.003	9.89E-57
GZMA	ENSG0000C	20.12	0.45	3.864	2.29E-48
TNFAIP8	ENSG0000C	14.51	2.67	2.079	3.60E-57
PLA2G7	ENSG0000C	10.56	0.2	3.268	6.25E-48
MSN	ENSG0000C	101.482	19.211	2.342	2.53E-49
IL2RG	ENSG0000C	38.109	1.51	3.962	8.68E-53
RPL10	ENSG0000C	3693.312	511.431	2.85	1.14E-55
PRDM14	ENSG0000C	21.17	0.45	3.934	1.10E-28
RPL7	ENSG0000C	2834.345	614.342	2.204	1.35E-53
STOM	ENSG0000C	166.571	28.46	2.508	5.68E-38
MKI67	ENSG0000C	23.271	3.79	2.341	4.02E-56
INA	ENSG0000C	18.8	3.13	2.261	6.24E-22
GSTO1	ENSG0000C	101.567	18.5	2.395	2.28E-47
RGS10	ENSG0000C	62.921	3.98	3.682	2.94E-76

SLC43A1	ENSG0000C	48.431	4.9	3.067	1.04E-18
SERPINH1	ENSG0000C	250.557	33.739	2.856	4.81E-55
RPS3	ENSG0000C	3259.42	684.828	2.249	1.39E-66
VENTX	ENSG0000C	33.639	1.3	3.913	1.31E-39
LPCAT1	ENSG0000C	65.78	11.42	2.427	1.07E-70
THY1	ENSG0000C	107.373	10.3	3.262	3.92E-56
PITPNC1	ENSG0000C	26.15	3.95	2.455	8.21E-45
GBP5	ENSG0000C	19.49	2.27	2.648	3.95E-25
MAGED4	ENSG0000C	19.191	0.81	3.48	4.35E-40
PDIA4	ENSG0000C	150.351	30.659	2.257	8.68E-52
RMND1	ENSG0000C	53.341	11.4	2.132	3.59E-45
SLA	ENSG0000C	7.42	1.02	2.059	9.86E-27
PCDH1	ENSG0000C	14.56	2.03	2.36	7.75E-41
RPL30	ENSG0000C	3746.427	843.298	2.15	1.05E-66
NODAL	ENSG0000C	12.21	2.26	2.019	3.61E-17
UTP14A	ENSG0000C	18.621	2.53	2.475	3.25E-65
MX1	ENSG0000C	85.698	8.87	3.135	3.62E-35
LDLRAP1	ENSG0000C	43.779	7.9	2.331	2.09E-48
KRTCAP3	ENSG0000C	29.14	6.25	2.056	4.94E-48
FAM46B	ENSG0000C	103.917	8.02	3.54	2.96E-27
NCF1	ENSG0000C	10.86	1.39	2.311	2.64E-27
SLAMF8	ENSG0000C	14.05	0.91	2.978	2.66E-37
FCER1G	ENSG0000C	89.411	6.54	3.584	6.33E-44
LAD1	ENSG0000C	12.93	0.45	3.264	4.60E-65
IGF2BP1	ENSG0000C	36.921	4.92	2.679	1.16E-37
GIP	ENSG0000C	40.439	0.44	4.847	2.61E-28
PTMS	ENSG0000C	568.138	130.598	2.113	1.56E-40
ITGB2	ENSG0000C	53.701	2.32	4.042	1.68E-50
VAV2	ENSG0000C	27.689	6.13	2.009	6.72E-42
RUSC1	ENSG0000C	41.18	8.98	2.079	9.78E-38
LY6E	ENSG0000C	413.229	65.199	2.646	4.27E-52
FOXH1	ENSG0000C	32.5	1.35	3.833	4.85E-43
RPL8	ENSG0000C	5915.973	712.822	3.051	1.35E-90
ZNF385A	ENSG0000C	25.871	4.05	2.412	1.51E-47
FAM171A2	ENSG0000C	8.49	0.76	2.431	2.83E-43
RPL26	ENSG0000C	3394.538	716.736	2.242	3.63E-69
POLR3K	ENSG0000C	19.43	3.25	2.265	7.03E-67
PDPN	ENSG0000C	146.361	4.14	4.841	2.88E-49
LAPTM5	ENSG0000C	73.659	4.39	3.792	9.41E-52
GBP4	ENSG0000C	13.94	1.17	2.783	5.67E-45
VANGL2	ENSG0000C	16.11	2.62	2.241	1.97E-57
PQLC3	ENSG0000C	85.23	8.99	3.11	1.61E-36
TNFAIP8L2	ENSG0000C	6.31	0.56	2.228	5.74E-41
S100A11	ENSG0000C	530.496	118.102	2.158	3.00E-21
FZD5	ENSG0000C	16.52	0.52	3.527	6.33E-51
IFI16	ENSG0000C	105.229	21.75	2.223	3.83E-24
SNHG16	ENSG0000C	101.04	19.56	2.311	2.85E-70
RPL9	ENSG0000C	1639.34	405.623	2.012	9.36E-60
TKT	ENSG0000C	232.743	53.02	2.113	6.59E-44

F2RL1	ENSG0000C	19.83	0.43	3.865	2.67E-77
SCGB3A2	ENSG0000C	31.13	0.13	4.83	7.89E-53
RPS14	ENSG0000C	1964.708	452.539	2.116	2.34E-69
SOX17	ENSG0000C	26.23	4.03	2.437	7.14E-18
FBP1	ENSG0000C	20.47	2.25	2.724	4.49E-22
SLC7A3	ENSG0000C	35.661	1.03	4.175	3.11E-49
SPTSSA	ENSG0000C	34.009	5.66	2.394	1.54E-55
FOLR2	ENSG0000C	14.91	2.85	2.047	1.54E-18
OTX2	ENSG0000C	7.83	0.54	2.519	2.46E-29
SALL2	ENSG0000C	14.34	1.77	2.469	1.07E-55
GYLTL1B	ENSG0000C	45.55	3.91	3.245	2.79E-57
SPINT1	ENSG0000C	84.752	2.69	4.539	1.75E-91
RPL27A	ENSG0000C	3085.31	524.899	2.553	2.02E-87
SNX20	ENSG0000C	4.65	0.26	2.165	2.06E-40
TUBA1C	ENSG0000C	831.515	39.659	4.356	1.14E-57
NLRP7	ENSG0000C	105.411	4.96	4.158	9.19E-26
TK1	ENSG0000C	37.369	4.15	2.897	1.51E-68
RPSA	ENSG0000C	3353.146	476.753	2.812	1.82E-73
GBX2	ENSG0000C	6.89	0.81	2.124	1.77E-20
SEMA4C	ENSG0000C	51.002	6.8	2.737	4.66E-43
PLA2G4F	ENSG0000C	9.02	0.16	3.111	2.39E-35
LGALS9	ENSG0000C	57.739	8.59	2.615	5.69E-41
SLC25A6	ENSG0000C	574.315	82.481	2.785	2.60E-57
RAB3B	ENSG0000C	6.92	0.38	2.521	4.00E-58
RNASE6	ENSG0000C	14.39	1.61	2.56	2.01E-37
MT1E	ENSG0000C	114.578	12.68	3.079	7.74E-24
GPRC5C	ENSG0000C	60.48	7.29	2.891	2.78E-55
KRT8	ENSG0000C	158.495	12.5	3.562	4.43E-24
MZB1	ENSG0000C	31.051	0.56	4.361	1.67E-45
SMAGP	ENSG0000C	74.899	10.68	2.7	5.14E-46
ZNF296	ENSG0000C	22.42	3	2.55	7.62E-33
KRT19	ENSG0000C	25.98	4.46	2.305	1.12E-09
RGS19	ENSG0000C	14.7	2.72	2.077	5.75E-78
TMEM51	ENSG0000C	23.669	3.26	2.534	6.66E-58
UTF1	ENSG0000C	51.349	7.76	2.579	1.46E-11
RRM2	ENSG0000C	47.359	5.87	2.815	2.61E-46
RPS21	ENSG0000C	3199.653	683.784	2.225	2.99E-64
RPS7	ENSG0000C	2555.34	552.296	2.208	2.49E-94
POP7	ENSG0000C	78.221	16.731	2.16	6.71E-70
MIR4435-2	ENSG0000C	22.65	3.05	2.546	3.46E-52
GLRX	ENSG0000C	26.06	3.4	2.621	1.55E-50
UQCRH	ENSG0000C	588.173	100.391	2.539	1.03E-59
JUP	ENSG0000C	127.964	16.29	2.899	2.33E-56
NRROS	ENSG0000C	11.93	1.87	2.172	7.09E-39
PHLDA3	ENSG0000C	117.01	17.78	2.652	1.60E-47
RPL4	ENSG0000C	3918.538	665.271	2.557	2.40E-93
KIAA1551	ENSG0000C	166.444	23.131	2.795	1.31E-58
UBE2C	ENSG0000C	146.118	22.33	2.657	7.89E-45
SFN	ENSG0000C	7.42	0.7	2.308	1.54E-30

ETV4	ENSG0000C	142.586	1.22	6.015	5.80E-98
GPX2	ENSG0000C	76.119	3.55	4.083	1.09E-43
PRR15	ENSG0000C	7.71	0.71	2.349	9.62E-37
NANOGP1	ENSG0000C	48.64	0.71	4.859	2.77E-42
VSIG10	ENSG0000C	52.8	10.37	2.242	2.99E-36
RPRM	ENSG0000C	22.92	1.51	3.252	3.84E-59
RPLP2	ENSG0000C	2293.602	563.588	2.023	7.21E-73
GLDC	ENSG0000C	65.33	4.29	3.648	3.19E-36
MSC	ENSG0000C	25.47	3.84	2.451	1.22E-23
RRS1	ENSG0000C	42.78	6.7	2.507	1.12E-74
TRIML2	ENSG0000C	53.578	5.89	2.986	1.45E-20
RCC2	ENSG0000C	208.354	28.111	2.846	1.36E-71
ZFP42	ENSG0000C	42.961	2.54	3.634	2.86E-34
GIMAP7	ENSG0000C	13.92	2.45	2.113	3.95E-28
HLA-DQB1	ENSG0000C	71.958	8.57	2.93	4.47E-26
1-Sep	ENSG0000C	10.64	1.85	2.03	6.06E-29
HCLS1	ENSG0000C	29.441	5.16	2.305	3.97E-29
PRF1	ENSG0000C	8.35	0.22	2.938	1.26E-43
RAP2B	ENSG0000C	14.77	2.51	2.168	2.24E-65
SNRPE	ENSG0000C	324.19	79.269	2.018	2.54E-63
IDH2	ENSG0000C	46.879	7.66	2.467	2.57E-55
NOP10	ENSG0000C	202.741	48.22	2.049	2.10E-70
SHMT2	ENSG0000C	95.49	12.07	2.884	5.07E-65
RPS17	ENSG0000C	3548.269	447.485	2.984	3.85E-90
LCK	ENSG0000C	12.1	0.75	2.904	7.47E-54
UPP1	ENSG0000C	175.959	13.64	3.595	9.92E-39
FAM162B	ENSG0000C	28.541	3.11	2.845	6.43E-29
KIRREL	ENSG0000C	14.41	2.61	2.094	6.00E-25
ST6GALNAC	ENSG0000C	5.13	0.41	2.12	9.07E-44
KCNK12	ENSG0000C	5.75	0.61	2.068	3.77E-35
GDF3	ENSG0000C	99.023	0.22	6.357	3.07E-45
SOCS3	ENSG0000C	16.95	1.98	2.591	7.78E-24
ZNF93	ENSG0000C	17.94	3.69	2.014	1.89E-41
MIXL1	ENSG0000C	4.87	0.45	2.017	1.42E-38
PDE6G	ENSG0000C	17.421	2.45	2.417	1.57E-22
P4HB	ENSG0000C	526.539	129.894	2.011	6.61E-61
NLRP9	ENSG0000C	6.96	0.2	2.73	9.42E-17
EVI2B	ENSG0000C	10.21	1.43	2.206	5.92E-30
IFITM1	ENSG0000C	481.635	64.039	2.892	2.70E-29
SAPCD2	ENSG0000C	5.98	0.65	2.081	4.58E-41
TOR3A	ENSG0000C	42.65	4.85	2.899	6.00E-82
RPS23	ENSG0000C	3761.78	838.402	2.164	2.29E-57
HPDL	ENSG0000C	5.93	0.44	2.267	1.18E-30
LILRB4	ENSG0000C	22.89	0.66	3.847	1.92E-44
TNFRSF4	ENSG0000C	7.37	0.6	2.387	4.77E-54
ERCC6L	ENSG0000C	11.72	0.75	2.862	1.11E-56
FPR3	ENSG0000C	7.75	0.83	2.257	1.59E-31
PTMA	ENSG0000C	1425.546	328.351	2.115	3.42E-82
NANOS3	ENSG0000C	198.555	8.06	4.461	8.11E-09

ISG15	ENSG0000C	158.385	9.67	3.901	8.10E-50
TMSB4XP8	ENSG0000C	11.51	0	3.645	3.47E-20
SPRY4	ENSG0000C	20.019	2.42	2.62	5.48E-29
TCEA1	ENSG0000C	115.896	25.23	2.156	3.51E-40
HIST1H1C	ENSG0000C	160.618	30.3	2.368	7.91E-17
SELL	ENSG0000C	7.44	0.76	2.262	1.30E-30
ZDHHC9	ENSG0000C	25.12	4.24	2.317	1.08E-47
RPL14	ENSG0000C	1687.532	262.504	2.68	6.16E-88
KLRG2	ENSG0000C	44.851	0.21	5.244	2.09E-48
LITAF	ENSG0000C	74.227	7.46	3.153	6.60E-52
S100A14	ENSG0000C	8.13	0.83	2.319	8.15E-17
HLA-DRB1	ENSG0000C	210.08	22.65	3.158	1.33E-39
S100A4	ENSG0000C	92.021	21.159	2.07	1.42E-24
TUBB	ENSG0000C	1027.127	103.271	3.302	1.30E-88
EPHB4	ENSG0000C	109.31	18.09	2.531	3.28E-57
HSH2D	ENSG0000C	6.25	0.5	2.273	2.30E-51
HLA-DQA1	ENSG0000C	69.391	1.75	4.678	1.29E-49
SERPINA1	ENSG0000C	43.299	2.26	3.764	5.27E-38
SPN	ENSG0000C	6.12	0.15	2.63	1.37E-47
MPEG1	ENSG0000C	13.6	0.62	3.172	2.83E-51
TEAD4	ENSG0000C	108.337	5.18	4.145	4.64E-74
RPL12	ENSG0000C	2545.793	456.793	2.476	2.73E-70
RPS4X	ENSG0000C	1906.353	189.768	3.322	2.15E-66
MT1F	ENSG0000C	37.681	3.31	3.166	5.20E-35
HLA-DRB5	ENSG0000C	39.62	2.46	3.553	1.19E-15
WDHD1	ENSG0000C	44.78	7.02	2.513	1.87E-43
PPIAP22	ENSG0000C	231.6	21.52	3.369	7.03E-78
GLMP	ENSG0000C	68.998	13.35	2.286	1.81E-50
RPL10A	ENSG0000C	1611.288	334.021	2.267	1.46E-48
PNP	ENSG0000C	55.718	10.38	2.317	9.20E-46
RPL39	ENSG0000C	3704.594	457.395	3.015	8.08E-65
FCGR3A	ENSG0000C	24.69	1.64	3.283	6.51E-45
KHDC3L	ENSG0000C	239.568	0.39	7.435	5.57E-44
HLA-DOA	ENSG0000C	7.97	0.64	2.451	9.96E-48
HNRNPCP2	ENSG0000C	16.999	2.69	2.286	2.95E-60
HLA-DMA	ENSG0000C	56.712	10.54	2.322	3.40E-33
PSMB8	ENSG0000C	43.371	7.88	2.321	2.88E-27
HLA-DRA	ENSG0000C	508.885	26.53	4.211	1.02E-52
LST1	ENSG0000C	26.82	3.15	2.745	2.93E-44
HLA-C	ENSG0000C	409.096	84.641	2.26	1.76E-37
POU5F1	ENSG0000C	1381.959	1.15	9.329	8.08E-67
HLA-E	ENSG0000C	196.896	45.86	2.078	1.52E-25
GNB2L1	ENSG0000C	2101.057	471.494	2.153	1.02E-74
MT1H	ENSG0000C	64.811	0.21	5.765	4.61E-36
TMSB4X	ENSG0000C	3739.422	475.894	2.971	2.63E-60
RP11-706C	ENSG0000C	32.88	7.42	2.009	2.27E-73
HLA-H	ENSG0000C	19.73	3.74	2.129	2.57E-18
TRIM71	ENSG0000C	25.72	4.53	2.273	2.79E-28
SNORA70	ENSG0000C	25.44	4.95	2.152	5.00E-08

SNORD15B	ENSG0000C	3.2	0	2.07	1.35E-07
IGKC	ENSG0000C	5973.661	9.24	9.189	2.92E-60
IGKV4-1	ENSG0000C	176.386	0.14	7.282	6.69E-50
IGLV4-69	ENSG0000C	19.66	0	4.369	2.32E-26
IGLV8-61	ENSG0000C	5.23	0	2.639	2.26E-18
IGLV6-57	ENSG0000C	29.08	1.09	3.847	4.95E-27
IGLV1-51	ENSG0000C	64.511	1.81	4.543	1.26E-30
IGLV1-47	ENSG0000C	88.15	0.22	6.191	5.78E-38
IGLV7-46	ENSG0000C	4.24	0	2.39	1.08E-21
IGLV1-44	ENSG0000C	59.379	0.17	5.689	1.06E-35
IGLV7-43	ENSG0000C	4.45	0	2.446	1.10E-21
IGLV1-40	ENSG0000C	71.699	0.35	5.751	1.21E-38
IGLV3-25	ENSG0000C	92.231	0	6.543	2.33E-41
IGLV2-23	ENSG0000C	62.691	0.13	5.817	3.81E-43
IGLV3-21	ENSG0000C	55.18	1.96	4.246	5.76E-25
IGLV3-19	ENSG0000C	72.188	0	6.194	1.67E-39
IGLV2-14	ENSG0000C	138.389	0.29	6.756	2.11E-45
IGLV2-11	ENSG0000C	29.97	0.21	4.678	2.84E-30
IGLV3-10	ENSG0000C	22.73	0	4.569	9.88E-34
IGLV3-1	ENSG0000C	110.407	0	6.8	1.99E-44
IGLC1	ENSG0000C	674.137	1.54	8.054	2.93E-56
IGLC2	ENSG0000C	1718.458	2.17	9.083	1.09E-59
IGLC3	ENSG0000C	1056.15	1.24	8.882	2.34E-57
IGLC7	ENSG0000C	5.44	0.41	2.191	2.79E-17
TRBV25-1	ENSG0000C	31.99	1.15	3.94	2.48E-45
TRBC2	ENSG0000C	60.761	11.01	2.362	4.25E-21
TRDC	ENSG0000C	6.1	0.08	2.717	1.56E-38
IGHA2	ENSG0000C	14.65	0.93	3.019	1.12E-26
IGHG4	ENSG0000C	67.841	1.64	4.705	3.04E-35
IGHG2	ENSG0000C	371.057	1.95	6.979	1.38E-52
IGHA1	ENSG0000C	186.275	3.17	5.489	2.89E-41
IGHG1	ENSG0000C	4046.895	6.87	9.007	2.26E-58
IGHG3	ENSG0000C	585.124	0.71	8.421	6.58E-59
IGHM	ENSG0000C	418.91	1.42	7.439	2.49E-52
IGHV1-2	ENSG0000C	31.321	0	5.014	4.95E-34
IGHV2-5	ENSG0000C	11.74	0.19	3.42	6.01E-22
IGHV3-7	ENSG0000C	6.43	0	2.893	8.75E-17
IGHV3-11	ENSG0000C	15.88	0	4.077	2.53E-31
IGHV3-15	ENSG0000C	38.711	0	5.311	1.59E-35
IGHV1-18	ENSG0000C	35.641	0.2	4.932	5.19E-31
IGHV3-21	ENSG0000C	44.019	0	5.492	1.85E-38
IGHV3-23	ENSG0000C	88.199	0.2	6.216	2.99E-43
IGHV3-33	ENSG0000C	74.392	0.19	5.985	8.13E-39
IGHV4-34	ENSG0000C	23.99	0.21	4.368	1.45E-32
IGHV4-39	ENSG0000C	36.75	0.19	4.987	9.56E-30
IGHV1-46	ENSG0000C	8.56	0.09	3.133	7.52E-22
IGHV3-48	ENSG0000C	4.31	0	2.409	1.22E-19
IGHV3-49	ENSG0000C	11.33	0.93	2.675	1.56E-14
IGHV5-51	ENSG0000C	39.45	0.15	5.136	1.07E-36

IGHV4-61	ENSG0000C	11.4	0	3.632	6.60E-21
IGHV1-69	ENSG0000C	7.62	0	3.108	9.83E-20
IGHV3-73	ENSG0000C	5.17	0	2.625	3.88E-20
SNORA45B	ENSG0000C	3.03	0	2.011	7.95E-05
RP11-641D	ENSG0000C	607.145	47.722	3.642	2.23E-97
TCL1B	ENSG0000C	36.59	3.55	3.046	1.85E-14
RPS7P11	ENSG0000C	8.58	0.78	2.428	6.29E-71
PTPRCAP	ENSG0000C	20.649	1.32	3.222	2.33E-44
RPL18AP3	ENSG0000C	72.378	15.19	2.18	5.66E-47
RPLP0P6	ENSG0000C	46	3.29	3.454	1.01E-62
LBH	ENSG0000C	57.15	12.51	2.106	6.16E-38
RPS29	ENSG0000C	6099.604	1441.544	2.08	8.32E-53
UBD	ENSG0000C	42.019	0.45	4.891	7.62E-51
PPM1N	ENSG0000C	34.999	2.23	3.478	1.53E-39
ZNF90	ENSG0000C	31.899	2.63	3.18	8.91E-46
FOXI3	ENSG0000C	10.38	0.35	3.076	2.02E-41
RPL7P1	ENSG0000C	29.39	5.06	2.326	1.38E-42
PHB2	ENSG0000C	590.952	67.111	3.12	1.04E-81
LINC00189	ENSG0000C	6.6	0.28	2.57	9.62E-51
IFI30	ENSG0000C	228.095	5.66	5.104	2.56E-77
RPS2P55	ENSG0000C	7.84	0.3	2.766	########
RP11-40C6	ENSG0000C	1135.175	0	10.15	########
RP11-572F	ENSG0000C	1106.969	234.996	2.231	1.01E-22
LINC00152	ENSG0000C	28.47	2.2	3.203	8.65E-59
HLA-DPB1	ENSG0000C	177.195	14.63	3.511	4.73E-52
UBE2SP2	ENSG0000C	30.13	2.37	3.208	1.24E-57
IGHV4-59	ENSG0000C	36.671	0	5.235	9.18E-39
HLA-DPB2	ENSG0000C	3.72	0.05	2.168	9.57E-33
HNRNPA1P4	ENSG0000C	31.8	6.97	2.041	6.47E-66
RP11-510€	ENSG0000C	76.468	7.8	3.138	6.73E-72
IGHV3-74	ENSG0000C	13.12	0.23	3.521	2.38E-23
GCSHP5	ENSG0000C	40.419	5.91	2.584	1.02E-53
RPL35P5	ENSG0000C	14.09	1.34	2.689	3.05E-75
RP4-706A1	ENSG0000C	43.09	9.13	2.122	1.04E-62
LINC00937	ENSG0000C	15.2	2.2	2.34	2.28E-33
RPS28P7	ENSG0000C	1271.045	116.419	3.437	5.41E-93
LTB	ENSG0000C	24.19	0.83	3.783	8.92E-49
HMGN1P37	ENSG0000C	8.48	0	3.245	8.18E-74
HLA-DRB6	ENSG0000C	8.91	0.47	2.753	4.33E-38
XIST	ENSG0000C	128.559	7.87	3.869	1.27E-18
TMSB4XP6	ENSG0000C	15.08	0	4.007	2.08E-07
TCEA1P2	ENSG0000C	9.73	1.43	2.143	4.55E-19
LINC0116C	ENSG0000C	32.401	3.93	2.76	2.17E-10
HLA-DPA1	ENSG0000C	165.455	15.43	3.341	2.01E-42
IGHV4-31	ENSG0000C	5.48	0	2.696	3.74E-19
RPS18	ENSG0000C	7370.26	1048.854	2.812	4.25E-88
SNRPFP1	ENSG0000C	4.24	0	2.39	9.56E-55
FTH1P7	ENSG0000C	19.74	2.33	2.639	8.51E-59
IGHV3-43	ENSG0000C	4.24	0.24	2.079	2.02E-15

RP11-132A	ENSG0000C	14.68	0.07	3.873	5.74E-32
RP11-132A	ENSG0000C	33.889	0.44	4.599	1.64E-60
HLA-DQB2	ENSG0000C	6.13	0.58	2.174	1.37E-22
ERVH48-1	ENSG0000C	29.229	0.41	4.422	2.86E-38
GAS5	ENSG0000C	547.455	119.27	2.189	6.84E-56
RPL23AP42	ENSG0000C	1726.697	274.735	2.647	1.67E-58
FABP5P7	ENSG0000C	103.852	20.019	2.319	1.28E-26
RPL39P3	ENSG0000C	724.83	67.841	3.398	5.27E-26
RPL6P27	ENSG0000C	19.69	3.49	2.204	1.93E-67
GAPDHP14	ENSG0000C	3.48	0.11	2.013	1.91E-42
RPL13AP5	ENSG0000C	122.947	22.45	2.402	3.21E-57
RP11-390F	ENSG0000C	5.19	0.18	2.391	3.98E-32
HLA-DQA2	ENSG0000C	7.44	0.45	2.541	4.00E-20
UBE2Q2P6	ENSG0000C	1854.737	417.171	2.15	2.96E-20
OR2I1P	ENSG0000C	3.02	0	2.007	1.31E-30
RP11-464D	ENSG0000C	4.68	0.39	2.031	1.83E-62
TXNDC5	ENSG0000C	83.719	17.4	2.203	7.95E-42
NME1	ENSG0000C	162.545	34.22	2.215	4.63E-69
IGKV1-6	ENSG0000C	7.04	0.14	2.818	1.15E-21
IGKV3-20	ENSG0000C	336.065	0.4	7.911	3.48E-54
IGKV1D-33	ENSG0000C	5.77	0	2.759	1.23E-26
PSMB9	ENSG0000C	47.94	6.09	2.787	1.50E-35
U47924.6	ENSG0000C	33.3	4.98	2.52	4.56E-33
IGKV1-17	ENSG0000C	28.68	0	4.891	9.31E-29
L1TD1	ENSG0000C	119.858	4.7	4.406	2.99E-43
IGKV1-8	ENSG0000C	19.08	0.12	4.164	1.14E-31
IGKV1-16	ENSG0000C	17.67	0	4.223	1.42E-29
TDGF1	ENSG0000C	9.38	0.81	2.52	1.47E-16
IGKV2-24	ENSG0000C	7.89	0	3.152	1.56E-24
RPL36A	ENSG0000C	3483.205	287.555	3.594	2.42E-91
IGKV3-11	ENSG0000C	364.101	0.67	7.772	1.85E-56
IGKV1-9	ENSG0000C	28.06	0	4.861	2.21E-36
IGKV1-33	ENSG0000C	133.768	0.14	6.885	1.67E-45
MTFP1	ENSG0000C	25.359	5.14	2.102	5.49E-49
IGKV2D-28	ENSG0000C	31.95	0	5.042	6.61E-32
HLA-DMB	ENSG0000C	44.111	3.73	3.254	5.09E-40
RP11-408F	ENSG0000C	11.32	1.43	2.342	2.67E-67
IGKV2-30	ENSG0000C	9.39	0	3.377	3.45E-28
IGKV2D-29	ENSG0000C	7.2	0	3.036	5.47E-25
RN7SL8P	ENSG0000C	4.43	0	2.441	2.35E-33
IGKV1-5	ENSG0000C	76.298	0.14	6.083	1.55E-45
NME1-NME2	ENSG0000C	711.045	101.581	2.795	3.30E-75
LEFTY1	ENSG0000C	8.44	1.16	2.128	7.95E-21
IGKV2-28	ENSG0000C	178.687	0	7.489	8.85E-46
RP11-466H	ENSG0000C	527.05	85.959	2.602	7.16E-51
IGKV3-15	ENSG0000C	104.516	0.24	6.411	1.63E-44
RP11-10G1	ENSG0000C	5.72	0	2.748	6.03E-92
IGKV1-27	ENSG0000C	18.87	0	4.313	2.82E-32
MTND4P12	ENSG0000C	37.46	0.53	4.652	6.76E-20

PCED1B-AS	ENSG0000C	14.48	1.08	2.896	3.03E-44
RP11-148E	ENSG0000C	42.411	0.28	5.084	6.00E-54
RP11-256F	ENSG0000C	5.76	0	2.757	6.63E-07
RP11-148E	ENSG0000C	5.58	0.08	2.607	3.63E-39
RP11-253E	ENSG0000C	11.22	0.69	2.854	7.19E-44
IGKV1D-39	ENSG0000C	244.416	0.32	7.539	4.14E-54
TRNP1	ENSG0000C	25.01	2.86	2.752	5.05E-66
RP11-798K	ENSG0000C	4.24	0	2.39	3.74E-27
LYN	ENSG0000C	11.57	1.78	2.177	6.41E-47
IGLL5	ENSG0000C	75.358	1.5	4.933	6.12E-41
MEX3A	ENSG0000C	11.67	0.84	2.784	6.16E-51
PIGY	ENSG0000C	14.52	2.3	2.234	2.24E-11
NANOGP8	ENSG0000C	54.56	0.99	4.803	1.07E-23
RP11-277E	ENSG0000C	4.54	0	2.47	1.40E-11
RP11-234E	ENSG0000C	8.89	0.13	3.13	1.50E-41
TRIL	ENSG0000C	5.99	0.65	2.083	1.57E-27
ZNF486	ENSG0000C	21.7	1.18	3.38	4.62E-46
RP11-771K	ENSG0000C	6.66	0.12	2.774	1.54E-36
RPL41P2	ENSG0000C	49.101	8.86	2.345	8.11E-37
SNRPEP2	ENSG0000C	13.13	2.24	2.125	9.39E-44
RP11-320N	ENSG0000C	7.83	0.12	2.979	4.53E-27
RP11-611C	ENSG0000C	9	1.23	2.165	5.41E-27
RP11-1143	ENSG0000C	15.33	0.54	3.407	5.39E-20
RP11-386G	ENSG0000C	433.262	78.498	2.45	1.48E-33
RP11-161H	ENSG0000C	508.462	24.06	4.346	2.21E-86
RP11-316M	ENSG0000C	10.79	0.31	3.17	1.68E-27
RP11-345J	ENSG0000C	11.5	0	3.644	6.17E-14
MMP12	ENSG0000C	7.32	0.06	2.972	6.54E-29
RP11-667K	ENSG0000C	11.2	0	3.609	1.06E-37
RP11-294J	ENSG0000C	4.76	0.07	2.428	2.03E-17
RP11-473I	ENSG0000C	3.21	0	2.074	2.36E-25
RP11-334E	ENSG0000C	40.109	3.13	3.315	6.76E-34
SNRPGP2	ENSG0000C	19.16	3.17	2.273	1.26E-47
ESRG	ENSG0000C	16.5	0.04	4.073	6.77E-36
RP3-461F1	ENSG0000C	70.992	5.92	3.379	1.66E-51
RP11-442H	ENSG0000C	4.66	0	2.501	2.69E-35
RP5-940J5	ENSG0000C	12.66	0	3.772	8.71E-16
IGHV3-30	ENSG0000C	139.342	0.18	6.894	8.29E-47
IGHV2-70	ENSG0000C	6.42	0	2.891	9.76E-22
RP11-457D	ENSG0000C	3.7	0	2.233	1.19E-29
HIST1H2BH	ENSG0000C	6.86	0.91	2.041	1.68E-31
RP11-843E	ENSG0000C	12.98	0.6	3.127	5.43E-48
TRAC	ENSG0000C	48.12	7.61	2.512	3.41E-30
RP11-452N	ENSG0000C	59.821	4.79	3.393	7.21E-64
IGLV2-8	ENSG0000C	16.151	0.11	3.95	1.49E-32
RPL41	ENSG0000C	5210.477	576.668	3.173	1.03E-68
RP11-63A1	ENSG0000C	3.14	0	2.05	1.10E-26
RP11-44N2	ENSG0000C	15.72	2.19	2.39	4.49E-15
FKSG62	ENSG0000C	7.42	0.61	2.387	7.52E-21

IGHV1-69-	ENSG0000C	28.479	0	4.882	9.00E-29
PCAT14	ENSG0000C	98.23	2.26	4.928	1.33E-46
RP3-467D1	ENSG0000C	16.14	0.49	3.524	6.77E-37

Table S2

GSE3218 DOWN-DEGs

	logFC	AveExpr	t	P.Value	adj.P.Val	B
SPACA1	-4.44548	4.903485	-63.5721	2.11E-87	2.63E-83	187.557
LINC00588	-3.1073	4.384861	-45.8443	1.28E-72	7.95E-69	154.8825
MSH4	-3.21302	4.770274	-40.8031	1.88E-67	7.80E-64	143.2616
RNFT1	-2.544	4.542406	-39.2985	8.44E-66	2.62E-62	139.5308
RIBC2	-5.04245	5.395351	-38.3125	1.09E-64	2.72E-61	137.0138
KPNA5	-4.81911	4.679376	-37.6775	5.88E-64	1.22E-60	135.3616
ZNF280B	-4.39581	5.549718	-36.159	3.61E-62	6.42E-59	131.3061
DNAI2	-4.06401	5.614811	-35.3681	3.28E-61	5.09E-58	129.1332
NACA2	-3.81643	4.383538	-34.6363	2.61E-60	3.61E-57	127.0843
TTC40	-3.92312	5.509752	-34.4236	4.81E-60	5.98E-57	126.4817
KANSL1L	-4.43527	4.717224	-33.896	2.22E-59	2.51E-56	124.9725
MORC1	-3.08911	4.193156	-33.3484	1.11E-58	1.15E-55	123.3848
GPR137	-2.82508	7.313656	-33.0217	2.92E-58	2.79E-55	122.4268
ADAM18	-3.98548	5.214746	-32.7405	6.76E-58	5.91E-55	121.5957
ADGB	-3.21987	4.410419	-32.7224	7.13E-58	5.91E-55	121.5418
ADCY10	-2.70537	5.055397	-32.1941	3.52E-57	2.73E-54	119.9633
MED7	-3.23389	6.461817	-31.5481	2.55E-56	1.87E-53	118.0027
CCDC70	-4.48016	5.407904	-31.0036	1.39E-55	9.59E-53	116.3239
SPO11	-4.17085	4.591645	-30.188	1.84E-54	1.14E-51	113.7633
RNF32	-4.87349	4.453373	-30.1465	2.10E-54	1.24E-51	113.6314
C11orf63	-5.19056	5.406154	-30.0906	2.51E-54	1.42E-51	113.4536
ADAM2	-5.74122	4.701372	-28.7236	2.19E-52	1.19E-49	109.0206
PAPOLB	-2.95192	4.772263	-28.5034	4.57E-52	2.11E-49	108.2906
LINC00837	-4.51853	4.676481	-28.4344	5.76E-52	2.56E-49	108.0612
BTG4	-3.96117	5.408784	-28.3957	6.56E-52	2.81E-49	107.9322
DPY19L1P1	-2.43667	4.866006	-28.2937	9.25E-52	3.83E-49	107.5913
LDHAL6B	-6.25625	4.851723	-27.906	3.44E-51	1.38E-48	106.288
SCIN	-2.60803	5.244489	-27.7927	5.06E-51	1.96E-48	105.9042
TMEM57	-2.89151	6.602747	-27.6647	7.83E-51	2.92E-48	105.4695
DNAJC28	-3.20172	4.838708	-27.6587	8.00E-51	2.92E-48	105.4488
GLB1L	-3.1458	7.424517	-27.6224	9.06E-51	3.22E-48	105.3254
CEP112	-5.26742	5.437052	-27.5766	1.06E-50	3.66E-48	105.1692
ATXN3L	-2.4586	4.871529	-27.2724	3.03E-50	9.90E-48	104.1267
RPGRIP1	-3.83986	6.451213	-27.0356	6.89E-50	2.20E-47	103.3092
SYNGR4	-3.93281	6.59106	-26.8908	1.14E-49	3.55E-47	102.8067
SMURF1	-2.96126	6.349629	-26.6074	3.09E-49	9.37E-47	101.8167
SPATA1	-2.27228	4.259151	-26.5355	3.98E-49	1.15E-46	101.5644
SOX30	-4.07442	4.737431	-26.3547	7.56E-49	2.09E-46	100.9271
TEX12	-2.61786	4.444907	-26.1877	1.37E-48	3.70E-46	100.336
CCDC88A	-2.97618	5.313718	-26.016	2.53E-48	6.69E-46	99.72518
CYLC1	-3.12021	5.611317	-26.0092	2.59E-48	6.71E-46	99.70089
DMC1	-2.72598	5.335899	-25.9801	2.88E-48	7.21E-46	99.59682
ELAVL2	-4.2131	4.947002	-25.9778	2.90E-48	7.21E-46	99.58894
UBQLN3	-6.46465	5.609914	-25.805	5.40E-48	1.32E-45	98.97014
GRK4	-4.62498	5.32801	-25.5896	1.18E-47	2.82E-45	98.1948
CCDC186	-3.37378	5.581683	-25.56	1.31E-47	3.08E-45	98.08806

CYLC2	-5.62813	5.317585	-25.496	1.65E-47	3.81E-45	97.85654
ERO1LB	-3.91543	5.22661	-25.4421	2.01E-47	4.55E-45	97.66114
GAPDHS	-6.05915	5.890437	-25.258	3.94E-47	8.76E-45	96.99191
AURKC	-4.42936	6.281318	-25.1705	5.44E-47	1.19E-44	96.67244
GPC5	-2.97527	4.681421	-25.1141	6.69E-47	1.43E-44	96.46616
CARF	-3.55479	4.496776	-25.0198	9.47E-47	1.96E-44	96.12052
LINC00342	-3.10688	5.233744	-25.0149	9.64E-47	1.96E-44	96.10247
SPATA31C:	-2.09378	4.479296	-24.8488	1.78E-46	3.57E-44	95.49101
C20orf195	-4.76045	6.210539	-24.7493	2.58E-46	5.08E-44	95.1237
C6orf10	-4.30046	5.337565	-24.6724	3.43E-46	6.66E-44	94.83892
IQCK	-2.23668	5.711925	-24.6096	4.33E-46	8.29E-44	94.60564
CAMK4	-2.90829	5.616801	-24.484	6.93E-46	1.31E-43	94.13811
ART3	-6.07695	5.093963	-24.457	7.67E-46	1.42E-43	94.03772
RWDD2A	-3.76499	5.750645	-24.437	8.26E-46	1.51E-43	93.96303
RFPL3S	-5.30029	4.993432	-24.4155	8.96E-46	1.61E-43	93.88245
AKAP4	-5.64016	5.561033	-24.3917	9.79E-46	1.74E-43	93.79369
CCHCR1	-3.17602	6.75992	-24.3496	1.15E-45	2.01E-43	93.63613
IRGC	-3.26893	5.467078	-24.3203	1.28E-45	2.21E-43	93.52657
TSSK2	-2.82359	6.680229	-24.1656	2.29E-45	3.91E-43	92.9455
GK2	-5.55196	6.164795	-24.1301	2.62E-45	4.41E-43	92.81179
NME8	-3.15835	5.284334	-24.095	3.00E-45	4.84E-43	92.6796
CBLN1	-3.69492	5.413333	-24.0947	3.00E-45	4.84E-43	92.67847
CCIN	-3.72546	6.72889	-24.0667	3.34E-45	5.32E-43	92.57295
SLC25A21	-2.03769	4.382415	-24.0411	3.68E-45	5.79E-43	92.47608
RTDR1	-3.13796	5.975804	-24.0127	4.09E-45	6.36E-43	92.36898
USP2	-2.14441	5.941551	-23.9978	4.33E-45	6.65E-43	92.31242
PPP1R2P9	-4.06273	5.948684	-23.9939	4.40E-45	6.67E-43	92.29794
CEP97	-2.00864	4.61567	-23.9837	4.57E-45	6.84E-43	92.25938
ADD3-AS1	-3.69161	5.907659	-23.9675	4.86E-45	7.19E-43	92.19802
PRKAA1	-2.60519	5.050583	-23.9516	5.16E-45	7.55E-43	92.13779
ALLC	-3.71499	6.432565	-23.8107	8.83E-45	1.28E-42	91.60299
C16orf95	-4.36665	6.589616	-23.7072	1.31E-44	1.87E-42	91.20901
TEX28	-2.4173	5.569671	-23.6704	1.51E-44	2.13E-42	91.06843
MYT1L	-2.70716	4.822134	-23.6594	1.58E-44	2.20E-42	91.02648
COIL	-3.96323	7.193157	-23.6558	1.60E-44	2.21E-42	91.01272
CT62	-4.26666	5.008402	-23.6125	1.89E-44	2.58E-42	90.8471
RFX2	-2.81468	6.635986	-23.566	2.25E-44	3.05E-42	90.66931
PEX7	-2.35802	6.041051	-23.5381	2.51E-44	3.36E-42	90.56219
MROH7	-3.71827	6.685117	-23.4768	3.18E-44	4.20E-42	90.32708
PRPS1L1	-4.64383	5.35119	-23.4734	3.22E-44	4.21E-42	90.31392
PMS2P8	-2.90072	6.05224	-23.4471	3.56E-44	4.61E-42	90.21288
TNP2	-2.18329	4.842645	-23.4059	4.18E-44	5.35E-42	90.0545
C9orf9	-4.84798	5.996348	-23.3693	4.81E-44	6.10E-42	89.91373
PSORS1C1	-3.955	5.372265	-23.3357	5.48E-44	6.88E-42	89.78421
GPR135	-3.07794	5.474999	-23.2752	6.93E-44	8.53E-42	89.5505
RPGRIP1L	-3.51063	4.646984	-23.153	1.11E-43	1.34E-41	89.07777
HSPA1L	-4.69626	6.602983	-23.0408	1.72E-43	2.06E-41	88.64181
TTLL7	-3.06323	5.502694	-22.8367	3.83E-43	4.45E-41	87.8458
PMFBP1	-3.03301	6.622311	-22.7401	5.60E-43	6.45E-41	87.46707

ZBTB32	-4.51284	5.473399	-22.7236	5.97E-43	6.81E-41	87.40247
LRRC36	-3.4144	7.070416	-22.6553	7.82E-43	8.76E-41	87.13396
FBXO24	-3.85728	5.451583	-22.6421	8.24E-43	9.14E-41	87.08216
NR2C2	-2.16226	5.471445	-22.4053	2.11E-42	2.32E-40	86.14658
POM121L9	-4.79384	5.919851	-22.1992	4.79E-42	5.22E-40	85.3273
AQP5	-2.83193	5.746522	-22.1547	5.72E-42	6.18E-40	85.14998
ZC2HC1C	-4.64194	6.03668	-22.149	5.85E-42	6.27E-40	85.12696
KDM4D	-5.21727	5.474984	-22.0874	7.49E-42	7.96E-40	84.88088
ZNRF4	-2.56907	7.632622	-22.0723	7.96E-42	8.39E-40	84.82036
ACRV1	-4.67373	6.332774	-22.0585	8.41E-42	8.79E-40	84.76517
ZSCAN5A	-2.93866	6.078625	-22.0248	9.63E-42	9.98E-40	84.63026
CTAGE1	-2.92427	4.833459	-22.0195	9.84E-42	1.01E-39	84.60906
NOL4	-2.85742	5.145032	-21.9227	1.45E-41	1.48E-39	84.22032
MMACHC	-2.10501	6.665404	-21.8971	1.61E-41	1.63E-39	84.1171
LRRC48	-2.97659	5.602891	-21.8612	1.86E-41	1.87E-39	83.97296
TSGA10	-5.54265	5.462958	-21.8112	2.28E-41	2.27E-39	83.77105
RDH8	-2.03304	5.765968	-21.7826	2.56E-41	2.52E-39	83.65594
CATSPERG	-3.73609	5.903981	-21.7719	2.67E-41	2.61E-39	83.61271
APH1B	-4.74756	7.579104	-21.6423	4.52E-41	4.39E-39	83.08842
GRAMD1C	-3.79388	4.910989	-21.6264	4.82E-41	4.64E-39	83.0239
SLCO3A1	-3.13397	6.652041	-21.533	7.05E-41	6.74E-39	82.64463
SNX19	-2.4474	7.198235	-21.5193	7.46E-41	7.08E-39	82.58863
MNS1	-5.48179	5.755329	-21.4808	8.72E-41	8.15E-39	82.432
SYCP2	-4.89675	4.654947	-21.4706	9.09E-41	8.44E-39	82.39053
VPRBP	-2.6738	6.201826	-21.4307	1.07E-40	9.86E-39	82.22776
LRIT1	-2.01514	5.268312	-21.4224	1.11E-40	1.01E-38	82.19398
CEP63	-2.86534	6.144112	-21.4191	1.12E-40	1.02E-38	82.18027
HIRIP3	-2.98443	7.372751	-21.3912	1.26E-40	1.13E-38	82.0666
PACRG	-3.55782	5.819784	-21.3701	1.37E-40	1.23E-38	81.98014
ZCCHC14	-2.44015	6.520705	-21.3278	1.63E-40	1.45E-38	81.8073
PPP3CC	-2.24829	5.599471	-21.2934	1.88E-40	1.65E-38	81.6663
IZUMO4	-5.06654	6.578573	-21.2921	1.89E-40	1.65E-38	81.6613
TEX15	-2.74872	5.669456	-21.2776	2.00E-40	1.74E-38	81.60183
TULP2	-3.58355	6.390667	-21.2712	2.06E-40	1.78E-38	81.57537
C16orf71	-4.15956	6.546048	-21.266	2.10E-40	1.79E-38	81.55429
EFCAB6	-3.01117	6.260419	-21.2642	2.12E-40	1.79E-38	81.54671
HCFC2	-4.71786	6.337392	-21.1709	3.11E-40	2.61E-38	81.16387
PGK2	-5.1248	5.852843	-21.1426	3.49E-40	2.92E-38	81.04731
HIF1AN	-2.26126	7.529895	-20.9762	6.95E-40	5.72E-38	80.36094
ADAM5	-2.1409	5.810096	-20.973	7.04E-40	5.76E-38	80.34773
WDR78	-3.99646	5.599738	-20.9524	7.67E-40	6.16E-38	80.26249
NGLY1	-2.60322	7.141346	-20.9517	7.69E-40	6.16E-38	80.25962
PRKACG	-3.08104	6.511578	-20.9505	7.73E-40	6.16E-38	80.25502
TDRD1	-2.31491	5.436178	-20.9476	7.82E-40	6.20E-38	80.24284
TRIM52	-2.49783	6.059618	-20.9287	8.46E-40	6.66E-38	80.16474
PDHA2	-5.34269	6.529259	-20.9034	9.40E-40	7.35E-38	80.05976
MTRF1	-2.68306	6.005864	-20.8822	1.03E-39	7.98E-38	79.97191
CLPB	-4.91359	6.721433	-20.8752	1.06E-39	8.16E-38	79.94302
ANKMY1	-2.4397	5.70584	-20.7904	1.50E-39	1.15E-37	79.5908

DDX25	-5.62386	6.005658	-20.7702	1.64E-39	1.25E-37	79.50671
ADAM30	-3.37912	6.24354	-20.7452	1.82E-39	1.38E-37	79.40267
CCNE2	-3.13497	5.408522	-20.6913	2.27E-39	1.71E-37	79.17815
ACTL7B	-4.75564	5.874317	-20.649	2.71E-39	2.03E-37	79.00166
TAF7L	-4.906	5.295409	-20.6066	3.24E-39	2.41E-37	78.82475
SSX3	-2.91095	6.464464	-20.6039	3.28E-39	2.42E-37	78.81339
PDE3B	-2.15636	5.878595	-20.5822	3.59E-39	2.64E-37	78.72259
CETN1	-3.27347	7.628004	-20.5513	4.09E-39	2.99E-37	78.59331
RAB27B	-2.05937	4.62696	-20.5248	4.57E-39	3.32E-37	78.48206
ZCWPW1	-2.94012	7.569064	-20.4995	5.08E-39	3.67E-37	78.3762
LRP2BP	-3.09561	5.689201	-20.4335	6.70E-39	4.79E-37	78.09923
SLC25A31	-6.10738	4.533061	-20.4179	7.16E-39	5.09E-37	78.0335
LECT2	-2.88807	5.154946	-20.379	8.43E-39	5.96E-37	77.87013
ARHGAP35	-2.19242	6.548975	-20.3346	1.02E-38	7.14E-37	77.68295
TCP11	-6.45698	6.802799	-20.1237	2.48E-38	1.70E-36	76.79183
AHI1	-2.32323	6.074999	-20.1196	2.53E-38	1.72E-36	76.77414
ACR	-3.09815	7.253251	-20.1055	2.68E-38	1.81E-36	76.71459
GK3P	-3.13301	5.413924	-20.0994	2.75E-38	1.85E-36	76.68836
OXCT2	-3.26288	7.173753	-20.0908	2.86E-38	1.91E-36	76.65221
STAM2	-2.42203	5.882331	-20.0672	3.16E-38	2.10E-36	76.5518
SAP30L	-2.97433	5.917729	-19.9841	4.50E-38	2.97E-36	76.19852
CAPN11	-2.93016	5.659523	-19.9549	5.09E-38	3.35E-36	76.07431
ZKSCAN4	-2.72765	6.884998	-19.8605	7.63E-38	4.99E-36	75.67124
TRAPPC12	-3.44376	8.009997	-19.8385	8.38E-38	5.46E-36	75.57729
CST8	-2.61667	6.676876	-19.8015	9.82E-38	6.36E-36	75.41907
CCDC7	-3.66144	6.851342	-19.7985	9.95E-38	6.41E-36	75.406
MCF2	-2.40565	5.018214	-19.7412	1.27E-37	8.15E-36	75.16079
CLIP4	-3.36565	6.112735	-19.6594	1.81E-37	1.15E-35	74.80961
DOC2A	-2.24336	6.186493	-19.6342	2.02E-37	1.27E-35	74.70107
HHIPL2	-3.1402	6.214085	-19.6303	2.05E-37	1.29E-35	74.68424
LOC81691	-6.70903	6.574109	-19.6094	2.24E-37	1.40E-35	74.59442
ST7L	-2.12182	6.614836	-19.5995	2.34E-37	1.45E-35	74.55197
CCDC15	-3.21546	6.30373	-19.5945	2.39E-37	1.48E-35	74.53044
GDF9	-2.01047	6.532865	-19.491	3.74E-37	2.29E-35	74.08399
SLC1A6	-3.39819	6.384219	-19.4447	4.57E-37	2.78E-35	73.88387
NAT6	-2.57657	7.373531	-19.4217	5.05E-37	3.05E-35	73.78446
PIWIL1	-5.94114	4.886973	-19.3892	5.81E-37	3.49E-35	73.64355
CDADC1	-2.02201	5.545865	-19.3482	6.94E-37	4.15E-35	73.46588
AP1AR	-2.11002	5.360355	-19.3215	7.80E-37	4.64E-35	73.34997
LYZL6	-3.27856	6.464771	-19.3147	8.03E-37	4.76E-35	73.32024
C17orf80	-3.12746	6.010081	-19.3095	8.21E-37	4.82E-35	73.29807
SSX1	-2.53784	5.435691	-19.3094	8.22E-37	4.82E-35	73.29753
SOCS7	-3.83476	6.681913	-19.2385	1.12E-36	6.53E-35	72.9894
SPATA6	-5.7972	6.095342	-19.2231	1.20E-36	6.95E-35	72.92235
KIF3A	-3.73063	5.855571	-19.2195	1.22E-36	7.03E-35	72.90665
AFF4	-3.89506	5.555483	-19.2159	1.23E-36	7.11E-35	72.89102
HSPA4L	-6.34665	5.290574	-19.1988	1.33E-36	7.61E-35	72.81629
HOOK1	-4.06726	5.907237	-19.1982	1.33E-36	7.61E-35	72.81388
ZNF821	-2.08499	7.075546	-19.1892	1.39E-36	7.88E-35	72.77447

SPATA7	-4.34355	5.339507	-19.161	1.57E-36	8.87E-35	72.65176
TRAF6	-2.13488	7.065783	-19.1026	2.03E-36	1.14E-34	72.39692
FNDC8	-3.53598	6.134446	-19.0955	2.09E-36	1.17E-34	72.36567
IMPACT	-3.01337	5.379108	-19.0878	2.16E-36	1.20E-34	72.33201
TBC1D12	-2.98836	6.463825	-19.0873	2.17E-36	1.20E-34	72.32993
DKKL1	-3.5153	6.853302	-19.0866	2.17E-36	1.20E-34	72.32699
ACE	-2.68181	5.822512	-19.0742	2.29E-36	1.26E-34	72.27274
PHTF1	-2.83412	6.517324	-19.0717	2.32E-36	1.27E-34	72.26171
MS4A5	-3.56406	6.652623	-19.0715	2.32E-36	1.27E-34	72.26061
FAM114A2	-2.06135	7.245951	-19.0645	2.39E-36	1.30E-34	72.23036
NR6A1	-2.13301	6.929058	-19.0151	2.97E-36	1.61E-34	72.01389
TSKS	-3.28321	6.019183	-18.9699	3.62E-36	1.93E-34	71.81594
KIAA0586	-2.02498	6.867789	-18.942	4.10E-36	2.18E-34	71.69356
TTC26	-2.43189	6.462369	-18.9237	4.44E-36	2.35E-34	71.61309
SYCP1	-5.01619	5.275333	-18.9015	4.89E-36	2.58E-34	71.51577
YPEL1	-5.43967	7.052433	-18.8915	5.12E-36	2.68E-34	71.47151
RGS7	-3.0892	4.90557	-18.8775	5.44E-36	2.84E-34	71.41009
MMP28	-2.75316	5.122302	-18.8335	6.60E-36	3.41E-34	71.21636
ROPN1B	-6.57893	6.19667	-18.7881	8.07E-36	4.14E-34	71.01643
HIST1H1T	-2.07979	6.347137	-18.7256	1.06E-35	5.40E-34	70.74064
CHD5	-2.64506	6.135964	-18.7153	1.11E-35	5.62E-34	70.69516
TEX40	-4.8607	7.415822	-18.7051	1.16E-35	5.86E-34	70.65025
LYRM2	-2.88423	5.245027	-18.6428	1.53E-35	7.67E-34	70.37463
IFT74	-2.92762	4.74336	-18.6424	1.54E-35	7.67E-34	70.3728
LDHC	-6.47991	6.818314	-18.6221	1.68E-35	8.36E-34	70.2828
TTC12	-2.50962	6.014209	-18.5794	2.03E-35	1.01E-33	70.09368
DNAH17	-3.27493	6.247792	-18.4887	3.04E-35	1.50E-33	69.69096
CTNNA3	-2.08267	6.369727	-18.4615	3.43E-35	1.69E-33	69.56973
ZBBX	-4.99773	4.967885	-18.448	3.65E-35	1.78E-33	69.50967
CRY1	-3.5378	6.236502	-18.424	4.06E-35	1.98E-33	69.40283
ZBTB48	-2.50502	6.265141	-18.3922	4.68E-35	2.27E-33	69.26098
TRIM66	-2.21436	6.983239	-18.3648	5.28E-35	2.56E-33	69.13909
SHCBP1L	-4.62607	6.331808	-18.3423	5.84E-35	2.82E-33	69.03846
BCAP29	-3.40054	5.445287	-18.3284	6.22E-35	2.98E-33	68.97636
CRISP2	-7.78247	5.312297	-18.2962	7.18E-35	3.42E-33	68.83251
FSCN3	-3.54228	7.865052	-18.1494	1.39E-34	6.53E-33	68.17459
UTRN	-3.6359	6.723664	-18.1323	1.50E-34	7.03E-33	68.09809
PIGV	-3.27327	6.992987	-18.1303	1.51E-34	7.07E-33	68.08873
BBS4	-2.37827	6.227007	-18.1093	1.66E-34	7.74E-33	67.99436
C1orf105	-3.71634	7.175392	-18.1047	1.70E-34	7.86E-33	67.9738
PRSS21	-4.71029	6.078814	-18.0712	1.97E-34	9.02E-33	67.82317
RORA	-3.232	5.492277	-18.0711	1.97E-34	9.02E-33	67.82254
RAD17	-3.04312	7.795615	-18.0697	1.99E-34	9.04E-33	67.81659
SPTBN2	-2.85942	6.273518	-18.0432	2.24E-34	1.02E-32	67.69722
SLC25A28	-2.26038	7.743847	-18.0043	2.67E-34	1.21E-32	67.52196
ACSBG2	-5.36397	6.622906	-17.9815	2.96E-34	1.33E-32	67.41901
ZMYND10	-4.10889	6.472051	-17.9783	3.00E-34	1.35E-32	67.40455
ACTL7A	-3.25778	7.635329	-17.9586	3.28E-34	1.47E-32	67.3155
IL13	-2.7539	6.468923	-17.9401	3.56E-34	1.59E-32	67.23232

IFT88	-4.78033	7.147302	-17.9202	3.90E-34	1.73E-32	67.14228
RAB11FIP5	-3.40293	7.738513	-17.9099	4.09E-34	1.80E-32	67.09561
SLC12A6	-3.42201	6.193337	-17.8865	4.54E-34	2.00E-32	66.98975
TCP10	-2.39299	5.84492	-17.8805	4.67E-34	2.04E-32	66.96255
HSF2BP	-3.05633	6.521347	-17.8706	4.88E-34	2.12E-32	66.91794
AGBL2	-3.71088	5.316701	-17.8378	5.66E-34	2.45E-32	66.76939
ZNF473	-3.19543	6.971105	-17.8316	5.82E-34	2.51E-32	66.74133
MAPRE3	-2.65798	6.104375	-17.7965	6.83E-34	2.93E-32	66.58205
DYRK3	-3.00204	7.483993	-17.7806	7.34E-34	3.14E-32	66.50985
MAK	-2.87575	5.243223	-17.77	7.70E-34	3.28E-32	66.46204
FER	-2.19431	6.550682	-17.7669	7.81E-34	3.32E-32	66.44772
AHCTF1	-3.53537	6.329533	-17.7487	8.49E-34	3.58E-32	66.36511
RSF1	-2.22909	5.271759	-17.7198	9.68E-34	4.04E-32	66.23393
CREM	-3.30236	6.57426	-17.7132	9.97E-34	4.15E-32	66.20395
ELL3	-3.6076	6.465172	-17.6726	1.20E-33	4.97E-32	66.0191
SFMBT1	-4.06183	7.325838	-17.6608	1.27E-33	5.23E-32	65.96554
TP73-AS1	-2.0538	6.310394	-17.6411	1.39E-33	5.68E-32	65.87564
ZPBP	-6.13428	6.424679	-17.5748	1.87E-33	7.64E-32	65.57373
USP32	-3.642	6.58684	-17.5692	1.92E-33	7.81E-32	65.548
SMG7-AS1	-2.06089	5.049594	-17.5516	2.08E-33	8.44E-32	65.46749
BBIP1	-3.31256	6.667482	-17.5213	2.39E-33	9.63E-32	65.32923
BRD8	-3.70348	7.28371	-17.5167	2.44E-33	9.80E-32	65.30814
CBL	-2.52274	6.401748	-17.4443	3.41E-33	1.36E-31	64.9767
SFT2D2	-2.4652	7.46571	-17.4374	3.52E-33	1.40E-31	64.94513
KIF2A	-3.14941	6.868574	-17.3863	4.44E-33	1.75E-31	64.71084
RBMXL2	-2.87591	5.611531	-17.3832	4.51E-33	1.77E-31	64.69685
ODF1	-5.55953	6.966183	-17.3584	5.05E-33	1.98E-31	64.58284
SOX5	-3.4092	6.141112	-17.3401	5.49E-33	2.14E-31	64.49893
CHGA	-4.67191	6.678828	-17.3292	5.78E-33	2.24E-31	64.4486
HYPM	-2.84641	6.459213	-17.3224	5.96E-33	2.31E-31	64.41769
DRICH1	-2.06917	6.508736	-17.3187	6.06E-33	2.34E-31	64.40042
ANKS1B	-2.08319	4.947075	-17.3097	6.32E-33	2.43E-31	64.35915
ABCB9	-2.37062	6.439843	-17.3046	6.47E-33	2.48E-31	64.33573
MAPKBP1	-2.68833	7.657991	-17.3028	6.52E-33	2.50E-31	64.32745
CHKA	-3.88811	7.490747	-17.288	6.98E-33	2.66E-31	64.2593
CCT6B	-5.53316	6.979407	-17.2743	7.44E-33	2.83E-31	64.19628
TEX30	-4.56658	7.215136	-17.2358	8.88E-33	3.36E-31	64.01913
PCSK2	-2.26273	5.020782	-17.2115	9.93E-33	3.74E-31	63.90714
ALDH1A2	-2.14024	6.2443	-17.211	9.96E-33	3.74E-31	63.90499
ANKRD7	-7.97415	5.179087	-17.1941	1.08E-32	4.03E-31	63.8267
CLTCL1	-2.84506	7.346788	-17.123	1.49E-32	5.58E-31	63.49876
SMPD2	-2.53185	7.381775	-17.1137	1.56E-32	5.81E-31	63.45549
CCNO	-2.63189	6.177703	-17.1077	1.60E-32	5.96E-31	63.42792
INTS6	-3.98381	6.359287	-17.0637	1.97E-32	7.26E-31	63.22455
SPANXB1	-2.43496	4.837606	-17.056	2.04E-32	7.50E-31	63.1886
TEX14	-5.42972	5.914249	-17.055	2.05E-32	7.51E-31	63.18407
MAGEC1	-3.57113	5.848646	-17.0514	2.08E-32	7.61E-31	63.16757
FAM188A	-2.39659	6.561369	-17.0316	2.28E-32	8.30E-31	63.07581
BAP1	-2.1625	8.763247	-17.0268	2.33E-32	8.46E-31	63.05352

FGFR1OP	-2.22917	6.791986	-17.0204	2.40E-32	8.69E-31	63.02376
CDKL3	-2.06379	5.912736	-17.0149	2.47E-32	8.89E-31	62.99838
SAMD4A	-2.22786	6.063249	-17.0045	2.59E-32	9.27E-31	62.95008
CASC1	-4.35012	4.944115	-16.9952	2.70E-32	9.66E-31	62.90693
NR3C2	-3.04408	5.119696	-16.9688	3.06E-32	1.08E-30	62.78432
CCNA1	-4.99205	6.690814	-16.9586	3.20E-32	1.13E-30	62.73734
PHF1	-2.19297	8.540085	-16.9551	3.26E-32	1.15E-30	62.72114
PRM2	-7.68646	6.45826	-16.9414	3.47E-32	1.22E-30	62.65721
TTBK2	-2.47386	4.773441	-16.9124	3.97E-32	1.38E-30	62.52266
ARHGAP28	-3.46191	5.833065	-16.8529	5.24E-32	1.81E-30	62.24561
MTL5	-4.23279	6.248373	-16.8466	5.39E-32	1.85E-30	62.21634
TSSK1B	-3.016	5.362889	-16.8056	6.53E-32	2.24E-30	62.02524
HRASLS	-5.3433	5.878191	-16.7812	7.32E-32	2.49E-30	61.91187
PRM1	-8.37738	6.395203	-16.7504	8.45E-32	2.87E-30	61.76795
SLC6A16	-5.07203	6.764617	-16.7341	9.12E-32	3.09E-30	61.69193
ODF2	-5.45865	6.85742	-16.7223	9.64E-32	3.26E-30	61.63667
STAG3	-4.33365	7.032465	-16.7129	1.01E-31	3.39E-30	61.59272
PPFIA1	-2.40023	7.042715	-16.71	1.02E-31	3.43E-30	61.57902
SPA17	-5.92999	6.301503	-16.7071	1.03E-31	3.47E-30	61.56585
C2orf42	-3.41933	7.488007	-16.695	1.09E-31	3.66E-30	61.50909
FKBP6	-2.8551	7.186265	-16.6898	1.12E-31	3.74E-30	61.48472
GSG1	-5.8506	5.465694	-16.6766	1.19E-31	3.97E-30	61.42296
SAYSD1	-2.73941	7.34008	-16.6646	1.26E-31	4.17E-30	61.36676
RNF19A	-2.69613	5.932377	-16.664	1.27E-31	4.17E-30	61.3642
MAP9	-2.21441	4.818482	-16.6322	1.47E-31	4.82E-30	61.21542
FRMPD1	-2.71415	5.819398	-16.6247	1.52E-31	4.98E-30	61.17995
GSTA3	-2.81342	6.453538	-16.6166	1.58E-31	5.16E-30	61.14234
SLC2A5	-3.12027	7.020215	-16.6124	1.61E-31	5.25E-30	61.12242
TEX13A	-2.30016	5.88654	-16.593	1.77E-31	5.73E-30	61.03139
SMCP	-4.52446	7.90725	-16.588	1.81E-31	5.85E-30	61.00816
C10orf88	-2.68133	5.871318	-16.5821	1.86E-31	6.00E-30	60.98041
LY6G5C	-2.06699	5.898081	-16.5376	2.29E-31	7.34E-30	60.77164
DLEU2	-2.36618	5.83982	-16.5246	2.43E-31	7.78E-30	60.71042
HPS5	-3.34033	6.095764	-16.5099	2.61E-31	8.29E-30	60.64145
KIAA0556	-2.22031	7.39799	-16.5029	2.70E-31	8.55E-30	60.60844
TTC30A	-2.76023	4.508603	-16.4912	2.85E-31	9.01E-30	60.55345
NUP62CL	-3.21881	5.094364	-16.4545	3.39E-31	1.07E-29	60.38096
C1QTNF9B	-2.40625	6.853172	-16.4431	3.57E-31	1.12E-29	60.32713
PHF7	-4.75428	6.627081	-16.4138	4.10E-31	1.28E-29	60.18944
RPL10L	-3.43346	6.969336	-16.3983	4.41E-31	1.36E-29	60.11645
LRP8	-3.74917	6.374441	-16.3915	4.56E-31	1.40E-29	60.08411
ZNHIT2	-2.21994	6.406333	-16.3832	4.74E-31	1.45E-29	60.04531
DAK	-2.12714	6.257958	-16.3776	4.86E-31	1.49E-29	60.01859
NDUFAF3	-3.48947	8.572338	-16.3661	5.14E-31	1.57E-29	59.96451
PCYT2	-4.10188	7.431033	-16.3653	5.15E-31	1.57E-29	59.96075
SPAG1	-2.76861	4.857447	-16.3465	5.63E-31	1.70E-29	59.87195
SPEF1	-2.59171	5.893128	-16.3219	6.33E-31	1.91E-29	59.75576
PHLPP2	-2.00205	7.293582	-16.3005	7.00E-31	2.10E-29	59.65475
CRAT	-3.0599	7.747921	-16.2798	7.72E-31	2.31E-29	59.55721

INHA	-5.00264	6.570891	-16.2423	9.22E-31	2.74E-29	59.37977
CUL3	-2.41075	6.477567	-16.2204	1.02E-30	3.03E-29	59.27592
RNF17	-2.5383	5.754407	-16.1871	1.20E-30	3.54E-29	59.11807
SUPT3H	-2.29209	6.603764	-16.1694	1.30E-30	3.83E-29	59.03452
TBCCD1	-2.81907	5.909432	-16.1632	1.34E-30	3.93E-29	59.0048
DUSP13	-2.8607	6.76595	-16.1386	1.51E-30	4.38E-29	58.88839
CLGN	-5.29002	4.912932	-16.1337	1.54E-30	4.46E-29	58.86475
OAZ3	-3.15665	5.649454	-16.1169	1.67E-30	4.80E-29	58.7852
TNP1	-7.57496	6.563554	-16.1152	1.68E-30	4.83E-29	58.77715
ATG9A	-2.43361	8.060869	-16.06	2.19E-30	6.20E-29	58.515
LIN7A	-2.68906	5.662861	-16.0597	2.19E-30	6.20E-29	58.51355
YBX2	-4.69256	8.102006	-16.0578	2.21E-30	6.24E-29	58.50453
SPAG9	-2.54869	6.455743	-16.044	2.36E-30	6.65E-29	58.43886
GPN3	-2.6681	7.252744	-16.0148	2.72E-30	7.57E-29	58.29977
DDX4	-6.29448	5.450864	-16.0127	2.74E-30	7.63E-29	58.28962
ULK2	-2.55788	6.661138	-16.0041	2.86E-30	7.93E-29	58.2489
SNAPC3	-2.10757	5.92063	-15.9903	3.05E-30	8.45E-29	58.18317
UIMC1	-3.10848	7.823505	-15.9802	3.20E-30	8.85E-29	58.13489
ZNF165	-4.57015	5.30425	-15.9686	3.39E-30	9.31E-29	58.07989
DNAI1	-3.35738	6.512497	-15.9671	3.41E-30	9.36E-29	58.0727
AK6	-2.29481	7.314851	-15.9612	3.51E-30	9.60E-29	58.04454
HMGN5	-3.11949	5.614694	-15.933	4.01E-30	1.09E-28	57.91006
CEP350	-2.46945	7.724332	-15.921	4.25E-30	1.15E-28	57.85284
VCX2	-4.26634	6.380686	-15.9073	4.54E-30	1.22E-28	57.78733
BAG1	-2.61597	8.137353	-15.8393	6.28E-30	1.68E-28	57.46228
IGLL1	-2.23291	5.825614	-15.8391	6.28E-30	1.68E-28	57.46158
TSNAXIP1	-2.39987	7.599606	-15.7968	7.69E-30	2.04E-28	57.25928
IQCE	-2.7493	6.143108	-15.7904	7.94E-30	2.10E-28	57.22826
DXO	-2.242	7.965519	-15.7864	8.09E-30	2.13E-28	57.2094
ADAM3A	-2.49354	4.501968	-15.7807	8.31E-30	2.19E-28	57.18198
FRS3	-2.4938	6.953835	-15.7789	8.38E-30	2.20E-28	57.17356
DNAH7	-2.49251	6.141841	-15.7544	9.43E-30	2.46E-28	57.05592
SPAG6	-4.93077	6.42241	-15.734	1.04E-29	2.69E-28	56.95836
PCM1	-2.45519	7.67165	-15.72	1.11E-29	2.87E-28	56.891
PLEKHA1	-3.421	6.97963	-15.7181	1.12E-29	2.90E-28	56.88189
PPP1R7	-2.35091	7.570857	-15.6938	1.26E-29	3.23E-28	56.76549
USP29	-2.0753	6.48798	-15.6568	1.51E-29	3.82E-28	56.58779
CDKL2	-2.04594	6.13561	-15.6556	1.52E-29	3.83E-28	56.58168
IPO13	-2.03095	8.220629	-15.6483	1.57E-29	3.96E-28	56.54658
VRK3	-2.33074	8.637323	-15.6149	1.84E-29	4.63E-28	56.38624
DZIP3	-2.91236	6.559429	-15.6129	1.86E-29	4.65E-28	56.37639
LIN37	-2.32415	5.106372	-15.5918	2.06E-29	5.11E-28	56.27519
SH3TC2	-2.39562	5.793933	-15.51	3.06E-29	7.54E-28	55.881
ANKRD36B	-4.50681	5.576938	-15.4772	3.58E-29	8.79E-28	55.72287
POMZP3	-3.75907	6.548461	-15.4547	3.99E-29	9.77E-28	55.61395
GIN1	-2.20906	6.248881	-15.4473	4.14E-29	1.01E-27	55.57862
DFNB31	-2.28422	6.718204	-15.4234	4.64E-29	1.13E-27	55.46294
RALGPS1	-2.035	6.031755	-15.3632	6.21E-29	1.50E-27	55.17174
PHKG2	-2.54389	7.951397	-15.3408	6.93E-29	1.67E-27	55.06315

NRIP3	-3.89519	7.289041	-15.3239	7.52E-29	1.81E-27	54.98146
CYB5R2	-4.42279	7.226263	-15.32	7.66E-29	1.84E-27	54.96266
BRDT	-5.20138	5.446948	-15.3072	8.15E-29	1.95E-27	54.90064
TOLLIP	-2.32815	7.775172	-15.3053	8.23E-29	1.96E-27	54.89123
OR7E14P	-2.87006	5.161762	-15.2545	1.05E-28	2.49E-27	54.64506
IFT140	-2.3288	5.252471	-15.2442	1.11E-28	2.61E-27	54.59497
RSPH6A	-2.35297	6.650737	-15.2391	1.13E-28	2.66E-27	54.57027
FAM169A	-3.79983	5.366105	-15.2347	1.16E-28	2.71E-27	54.5491
TBL2	-2.66798	7.477836	-15.1991	1.38E-28	3.20E-27	54.37583
EDC3	-2.4565	6.555173	-15.1926	1.42E-28	3.29E-27	54.34435
MYBL1	-4.11932	5.63578	-15.1921	1.43E-28	3.29E-27	54.3421
KIAA0895	-3.6268	4.602207	-15.1549	1.71E-28	3.93E-27	54.16104
H2AFJ	-2.39429	4.940761	-15.1196	2.03E-28	4.63E-27	53.98921
VAMP2	-2.31765	7.115401	-15.0429	2.95E-28	6.68E-27	53.61548
PIBF1	-2.73125	6.077351	-15.0405	2.98E-28	6.74E-27	53.60366
GID4	-3.74372	6.900302	-15.0184	3.32E-28	7.49E-27	53.49574
ELF2	-2.28881	7.372361	-14.9985	3.66E-28	8.22E-27	53.39897
ADORA1	-2.16192	6.61663	-14.9898	3.82E-28	8.56E-27	53.35651
MTMR6	-3.38604	6.41397	-14.9821	3.97E-28	8.85E-27	53.31893
B9D1	-2.07831	6.347709	-14.9741	4.13E-28	9.19E-27	53.2797
CCDC181	-4.2906	4.9927	-14.9725	4.16E-28	9.25E-27	53.27178
SPINK2	-6.07512	6.938706	-14.9711	4.19E-28	9.29E-27	53.26516
RUNDC3B	-2.82749	4.740195	-14.9113	5.61E-28	1.24E-26	52.97277
TLE4	-2.22793	5.931339	-14.9105	5.63E-28	1.24E-26	52.96869
CNNM1	-2.53486	6.642546	-14.8833	6.43E-28	1.41E-26	52.83561
C9orf116	-2.7285	6.359496	-14.8796	6.55E-28	1.43E-26	52.81726
CNTRL	-3.32685	7.183067	-14.8723	6.79E-28	1.48E-26	52.78156
MARK1	-2.62442	6.129805	-14.8701	6.87E-28	1.50E-26	52.77082
KIF18A	-2.97474	5.614303	-14.8691	6.90E-28	1.50E-26	52.76609
CTNNA2	-3.89387	5.700915	-14.8534	7.45E-28	1.61E-26	52.68927
GAPVD1	-2.26234	7.61799	-14.8516	7.52E-28	1.62E-26	52.68002
SLC30A4	-2.21261	4.812768	-14.85	7.58E-28	1.63E-26	52.67214
SRPK2	-2.30492	7.486576	-14.8482	7.64E-28	1.64E-26	52.66356
CDK20	-2.01565	7.454926	-14.825	8.56E-28	1.84E-26	52.54976
FAAH	-2.80665	6.852013	-14.8202	8.77E-28	1.88E-26	52.52609
PIP5K1C	-2.10596	8.573517	-14.8092	9.26E-28	1.98E-26	52.47224
GEMIN4	-2.39269	8.084962	-14.8066	9.37E-28	2.00E-26	52.45954
TP53TG5	-2.94721	6.920037	-14.8009	9.64E-28	2.05E-26	52.43179
OBFC1	-2.20575	7.588584	-14.7988	9.74E-28	2.07E-26	52.42111
BRAP	-2.41107	6.817238	-14.7968	9.84E-28	2.08E-26	52.41147
CCNH	-3.21127	8.379597	-14.7944	9.95E-28	2.10E-26	52.39963
NME5	-5.18716	6.736024	-14.7642	1.15E-27	2.44E-26	52.25156
ATF7IP2	-2.75505	7.783134	-14.7436	1.28E-27	2.69E-26	52.15014
SCAPER	-2.27696	6.434705	-14.7116	1.50E-27	3.12E-26	51.99296
STX18	-2.44155	8.238113	-14.6725	1.81E-27	3.74E-26	51.80098
CENPC	-2.04031	6.678157	-14.6407	2.12E-27	4.35E-26	51.64429
PSMC3IP	-2.16874	7.144019	-14.6151	2.40E-27	4.91E-26	51.51829
NDUFAF1	-2.55394	7.461832	-14.5764	2.91E-27	5.92E-26	51.32726
AR	-2.19704	5.697173	-14.5037	4.17E-27	8.44E-26	50.96878

CABYR	-5.6267	6.621019	-14.5032	4.18E-27	8.45E-26	50.966
RUFY3	-2.10476	5.937532	-14.4908	4.44E-27	8.94E-26	50.90465
ZDHHC14	-2.12996	7.59643	-14.4637	5.08E-27	1.02E-25	50.7708
AGFG1	-2.01246	6.724473	-14.4468	5.52E-27	1.10E-25	50.68707
YEATS4	-2.44318	5.633037	-14.4442	5.59E-27	1.11E-25	50.67469
CBLL1	-2.09806	4.7374	-14.4248	6.16E-27	1.22E-25	50.57847
DPEP3	-4.47074	6.069473	-14.4154	6.45E-27	1.28E-25	50.53206
GPS2	-2.44056	8.364721	-14.3807	7.66E-27	1.51E-25	50.36023
ZNF415	-2.58582	5.855167	-14.3718	8.01E-27	1.58E-25	50.31593
USPL1	-2.52131	7.805907	-14.3429	9.24E-27	1.82E-25	50.17304
PPME1	-2.12969	8.904824	-14.3033	1.12E-26	2.20E-25	49.97646
SLC30A3	-2.49338	8.75094	-14.2937	1.18E-26	2.30E-25	49.92866
NR0B1	-2.46325	5.433061	-14.2805	1.26E-26	2.45E-25	49.86349
UPF3A	-2.23616	7.418565	-14.245	1.50E-26	2.92E-25	49.68699
AKAP11	-2.47968	7.083172	-14.2439	1.51E-26	2.93E-25	49.68169
MPP6	-3.37108	5.960802	-14.1971	1.91E-26	3.66E-25	49.44883
FAM53C	-2.20111	8.383786	-14.1815	2.06E-26	3.94E-25	49.37124
PLSCR2	-3.01875	5.677229	-14.174	2.14E-26	4.08E-25	49.3338
CSNK2A2	-3.65623	8.076622	-14.1525	2.38E-26	4.52E-25	49.22713
TOPORS	-2.17812	6.014314	-14.1513	2.39E-26	4.54E-25	49.22115
KLHL11	-2.16582	7.067531	-14.1475	2.44E-26	4.62E-25	49.20218
DZIP1	-3.24812	6.383238	-14.1415	2.52E-26	4.75E-25	49.17221
PIK3R3	-3.12639	7.124495	-14.1335	2.62E-26	4.93E-25	49.13234
CUTC	-3.1978	7.052847	-14.0997	3.10E-26	5.79E-25	48.96406
SIK3	-2.1606	7.108875	-14.0641	3.70E-26	6.86E-25	48.78626
GAGE3	-5.55329	6.761195	-14.0495	3.98E-26	7.35E-25	48.71351
PAOX	-2.96454	6.866966	-14.044	4.09E-26	7.54E-25	48.68627
DIXDC1	-3.20703	7.069851	-14.0411	4.15E-26	7.64E-25	48.67191
KIF5B	-2.65986	8.322283	-14.0264	4.47E-26	8.19E-25	48.59841
RABIF	-2.0965	7.457081	-13.9934	5.27E-26	9.61E-25	48.43374
DHX57	-2.06569	6.14485	-13.9925	5.29E-26	9.65E-25	48.42882
CGRRF1	-2.8089	7.158008	-13.9883	5.40E-26	9.84E-25	48.40791
ZDHHC13	-2.59275	6.687047	-13.9864	5.46E-26	9.92E-25	48.39838
KCTD13	-3.3202	7.418382	-13.9719	5.86E-26	1.06E-24	48.32632
ATG2B	-2.30039	5.224871	-13.9672	6.00E-26	1.08E-24	48.3028
CCP110	-3.06591	7.527872	-13.9621	6.16E-26	1.11E-24	48.27712
RNF14	-2.99337	6.693867	-13.9351	7.05E-26	1.26E-24	48.14212
HAGH	-2.4821	7.853889	-13.925	7.42E-26	1.32E-24	48.09142
AP4M1	-2.14322	6.956269	-13.8327	1.18E-25	2.08E-24	47.62961
TTLL1	-2.3454	6.611541	-13.8322	1.18E-25	2.08E-24	47.62717
PGAM2	-3.46722	5.400421	-13.8254	1.22E-25	2.15E-24	47.59292
GRAMD1B	-2.28965	7.259998	-13.7635	1.67E-25	2.90E-24	47.2826
FHL5	-2.29632	5.193174	-13.7541	1.75E-25	3.04E-24	47.23495
TMCC1	-2.09236	6.496157	-13.7227	2.05E-25	3.54E-24	47.07738
LRRC49	-3.27588	6.140506	-13.6843	2.48E-25	4.27E-24	46.88463
IK	-2.15291	9.369106	-13.6696	2.67E-25	4.58E-24	46.8107
FUZ	-2.17736	6.842258	-13.668	2.69E-25	4.61E-24	46.80251
SETX	-2.33375	8.271714	-13.6399	3.10E-25	5.29E-24	46.66115
S1PR2	-2.48464	5.810728	-13.6226	3.39E-25	5.74E-24	46.5742

SHARPIN	-2.18751	7.946245	-13.6155	3.51E-25	5.95E-24	46.53839
METTL22	-2.50238	7.652408	-13.6009	3.78E-25	6.37E-24	46.46475
CCDC19	-2.2386	6.656634	-13.5952	3.89E-25	6.55E-24	46.43648
PROX1	-2.08644	5.424008	-13.5854	4.08E-25	6.84E-24	46.38697
CCT8L2	-2.1142	6.081772	-13.5683	4.45E-25	7.44E-24	46.30093
DYNC2H1	-2.655	6.933518	-13.5375	5.20E-25	8.62E-24	46.14564
COQ9	-2.13783	8.464972	-13.534	5.29E-25	8.75E-24	46.12798
DNALI1	-3.81483	6.67465	-13.5269	5.49E-25	9.05E-24	46.09184
ASB9	-3.53444	6.163623	-13.4803	6.94E-25	1.14E-23	45.85691
TRIM13	-3.40275	7.686263	-13.4801	6.95E-25	1.14E-23	45.85612
EIF4G3	-2.61193	8.174015	-13.4121	9.80E-25	1.59E-23	45.51265
FEM1C	-2.23314	6.865346	-13.3806	1.15E-24	1.86E-23	45.35324
NUP98	-2.1686	7.453997	-13.3684	1.22E-24	1.96E-23	45.29168
SURF2	-2.73777	6.801819	-13.358	1.29E-24	2.06E-23	45.23892
CDK8	-2.01013	6.214044	-13.3518	1.33E-24	2.13E-23	45.20744
OXR1	-3.32142	5.580095	-13.3467	1.36E-24	2.18E-23	45.18158
CDK19	-2.21718	7.410627	-13.3311	1.48E-24	2.34E-23	45.10277
FAM216A	-4.63852	6.641428	-13.3309	1.48E-24	2.34E-23	45.10174
PAAF1	-2.25828	8.082401	-13.3055	1.68E-24	2.66E-23	44.97333
OR7E156P	-3.40311	7.812759	-13.3047	1.69E-24	2.66E-23	44.96897
UBN1	-2.59502	8.092431	-13.3015	1.72E-24	2.70E-23	44.95288
SKAP2	-2.09504	5.951353	-13.2951	1.77E-24	2.78E-23	44.92027
PGR	-2.28157	4.94154	-13.278	1.93E-24	3.02E-23	44.83373
LOC44160:	-2.22065	7.102141	-13.2667	2.05E-24	3.18E-23	44.77668
CCDC176	-3.05503	7.90462	-13.2385	2.36E-24	3.66E-23	44.63358
MTO1	-2.70319	6.794142	-13.2235	2.55E-24	3.94E-23	44.55745
PGBD5	-2.70003	6.224551	-13.1954	2.94E-24	4.52E-23	44.41526
CTSF	-3.07027	7.726991	-13.1492	3.72E-24	5.69E-23	44.18047
PEX13	-2.45031	6.584956	-13.11	4.54E-24	6.84E-23	43.9813
MEPCE	-2.27728	9.182	-13.1008	4.75E-24	7.11E-23	43.93486
ARL4A	-3.84212	8.540869	-12.9984	8.01E-24	1.18E-22	43.41354
SPRR2C	-2.72679	5.624144	-12.9673	9.39E-24	1.38E-22	43.2552
TLR3	-2.05736	5.131031	-12.9585	9.82E-24	1.44E-22	43.2102
CCDC86	-2.30288	8.291971	-12.9471	1.04E-23	1.52E-22	43.15251
DENND5B	-2.0394	4.724337	-12.9467	1.04E-23	1.52E-22	43.15004
NEK4	-3.94072	6.634836	-12.9403	1.08E-23	1.57E-22	43.11787
PIWIL2	-2.07299	6.332327	-12.9254	1.16E-23	1.69E-22	43.04179
RANBP9	-3.30275	7.734765	-12.9098	1.26E-23	1.82E-22	42.9619
WDR60	-2.53209	6.910319	-12.8903	1.39E-23	2.01E-22	42.86271
PCID2	-2.30697	8.323737	-12.8552	1.66E-23	2.40E-22	42.68336
PDXK	-2.70455	8.604124	-12.8479	1.73E-23	2.49E-22	42.64652
DOCK3	-2.2321	5.461148	-12.8466	1.74E-23	2.50E-22	42.63959
ABCA5	-2.81581	5.757823	-12.8339	1.85E-23	2.65E-22	42.57497
TFDP3	-2.01126	6.417662	-12.8282	1.91E-23	2.73E-22	42.5456
ASB8	-2.03195	6.130533	-12.8257	1.93E-23	2.76E-22	42.53315
TBP	-2.24299	7.621944	-12.8238	1.95E-23	2.78E-22	42.52328
GDF10	-2.29802	6.33961	-12.8086	2.11E-23	2.99E-22	42.44549
COX20	-2.69185	6.710333	-12.7974	2.24E-23	3.16E-22	42.38812
ARFGAP3	-2.46563	7.705581	-12.7866	2.36E-23	3.33E-22	42.33315

PPEF1	-3.13003	5.773733	-12.766	2.63E-23	3.70E-22	42.22771
ANKRD55	-2.30038	6.049488	-12.7533	2.80E-23	3.93E-22	42.16295
STAG3L3	-2.91559	6.570129	-12.7406	2.99E-23	4.19E-22	42.09776
NPIPB15	-2.49974	7.628854	-12.7391	3.01E-23	4.21E-22	42.09036
13-Nov	-2.96713	6.532824	-12.708	3.53E-23	4.91E-22	41.93131
ETNK2	-2.62164	6.932527	-12.7008	3.67E-23	5.08E-22	41.89455
MARK4	-2.66006	8.027455	-12.6693	4.31E-23	5.94E-22	41.73321
AGBL5	-2.96728	8.03377	-12.6627	4.46E-23	6.13E-22	41.69917
SLC2A8	-2.29071	7.373873	-12.6624	4.46E-23	6.13E-22	41.69802
ELOVL2	-2.05933	5.653544	-12.6448	4.89E-23	6.68E-22	41.60752
TBPL1	-3.3523	8.29976	-12.5872	6.57E-23	8.88E-22	41.3126
TUBGCP5	-2.17248	4.753949	-12.5855	6.62E-23	8.95E-22	41.30392
SSX2IP	-2.48096	6.781884	-12.5797	6.82E-23	9.20E-22	41.27409
TEKT2	-2.36898	6.41064	-12.5791	6.84E-23	9.22E-22	41.2711
SKAP1	-2.57041	6.847066	-12.5774	6.90E-23	9.29E-22	41.26245
TMBIM4	-2.22458	8.08796	-12.5667	7.30E-23	9.77E-22	41.20734
OPLAH	-2.44262	6.546276	-12.562	7.47E-23	9.98E-22	41.18316
TCFL5	-4.5144	7.569513	-12.5399	8.37E-23	1.11E-21	41.06984
MKLN1	-2.01151	6.986061	-12.537	8.50E-23	1.13E-21	41.05481
CTAG2	-2.0897	5.89657	-12.5199	9.28E-23	1.23E-21	40.9674
KLHL12	-2.62105	7.132192	-12.5079	9.87E-23	1.30E-21	40.90573
AMZ2	-2.79889	9.060716	-12.5077	9.88E-23	1.30E-21	40.90457
ATP8A2	-2.42477	6.274294	-12.4934	1.06E-22	1.40E-21	40.83104
PRPH	-2.04804	8.256497	-12.4792	1.14E-22	1.50E-21	40.75834
ARHGAP19	-2.36644	6.774648	-12.4598	1.26E-22	1.66E-21	40.65854
RNF10	-2.20339	8.168638	-12.4556	1.29E-22	1.69E-21	40.6369
ITCH	-2.33747	7.299514	-12.4306	1.47E-22	1.91E-21	40.50877
CCSER2	-2.3001	7.745478	-12.3952	1.76E-22	2.28E-21	40.32664
PAK6	-3.34467	6.246423	-12.3892	1.82E-22	2.35E-21	40.29574
C12orf10	-2.52462	8.402792	-12.3885	1.82E-22	2.35E-21	40.29216
SLC35G2	-2.89577	6.988268	-12.3813	1.89E-22	2.44E-21	40.2554
CHODL	-4.59749	6.662978	-12.3712	1.99E-22	2.56E-21	40.20346
AKAP3	-4.71896	6.705648	-12.3666	2.04E-22	2.61E-21	40.1799
ZNF536	-2.11004	6.686155	-12.3651	2.06E-22	2.63E-21	40.17213
ADO	-2.72229	7.767231	-12.361	2.10E-22	2.68E-21	40.15069
ERI3	-2.01404	7.270218	-12.3552	2.16E-22	2.76E-21	40.12111
KHDRBS3	-3.86323	7.919214	-12.3535	2.18E-22	2.78E-21	40.11231
MYLK3	-2.94446	4.978365	-12.3533	2.19E-22	2.78E-21	40.11125
SMA4	-2.23784	5.871035	-12.3339	2.42E-22	3.05E-21	40.01138
CDKN2AIP	-2.38291	7.053067	-12.3226	2.56E-22	3.23E-21	39.95337
HGFAC	-2.31115	5.793434	-12.3008	2.87E-22	3.60E-21	39.84093
POLI	-3.5492	5.807773	-12.2928	2.99E-22	3.74E-21	39.79969
CRYBG3	-2.75857	8.054011	-12.2615	3.51E-22	4.38E-21	39.6387
GGNBP2	-2.21801	9.08485	-12.2399	3.92E-22	4.90E-21	39.52733
APOBEC3C	-2.36097	6.188143	-12.2204	4.34E-22	5.40E-21	39.42714
TPGS2	-2.36936	7.557351	-12.2041	4.72E-22	5.85E-21	39.34298
AP1G1	-2.4137	8.087507	-12.1997	4.83E-22	5.98E-21	39.32042
MRGBP	-3.25152	7.229256	-12.1816	5.30E-22	6.54E-21	39.22703
FAM46C	-3.54546	7.617991	-12.1765	5.44E-22	6.71E-21	39.20062

DEPDC1	-2.65912	6.065567	-12.1761	5.45E-22	6.71E-21	39.19874
MORC2	-2.26047	7.577988	-12.1649	5.78E-22	7.08E-21	39.14064
SNAP91	-3.16525	5.605191	-12.1337	6.79E-22	8.23E-21	38.97992
MBNL3	-3.96506	5.430772	-12.1255	7.08E-22	8.58E-21	38.93756
INPP4B	-2.38816	5.274204	-12.1043	7.90E-22	9.55E-21	38.82815
SLC4A1AP	-2.34683	7.666272	-12.0959	8.25E-22	9.95E-21	38.78484
APPBP2	-2.0915	6.360338	-12.0874	8.63E-22	1.04E-20	38.7406
C21orf91	-2.49419	7.025602	-12.0857	8.70E-22	1.05E-20	38.73206
IPO5	-2.57779	7.753367	-12.0795	8.99E-22	1.08E-20	38.7
GRB14	-3.58791	5.22481	-12.0742	9.24E-22	1.11E-20	38.67242
TPTE	-6.4765	6.113187	-12.0709	9.39E-22	1.12E-20	38.6555
MUM1	-2.68602	6.111931	-12.0692	9.48E-22	1.13E-20	38.64692
SPAG4	-2.5558	6.570452	-12.0501	1.05E-21	1.25E-20	38.54835
ELP5	-3.2857	7.928567	-12.0434	1.08E-21	1.29E-20	38.51373
TPP2	-2.20171	7.570691	-12.0379	1.11E-21	1.32E-20	38.48537
PPP1R9A	-2.39502	6.028965	-12.0298	1.16E-21	1.38E-20	38.44324
BRD1	-2.51602	8.235803	-12.0055	1.32E-21	1.56E-20	38.3178
IQCG	-3.87816	6.537882	-11.986	1.46E-21	1.72E-20	38.21707
RNF141	-2.52142	7.476306	-11.9856	1.46E-21	1.72E-20	38.21508
DCAF6	-3.04128	7.614657	-11.9695	1.59E-21	1.86E-20	38.13175
TBK1	-2.38214	8.043976	-11.9438	1.81E-21	2.12E-20	37.9985
TTC17	-2.46143	7.82732	-11.9281	1.97E-21	2.30E-20	37.91742
WDR19	-2.67736	7.02659	-11.8978	2.30E-21	2.67E-20	37.76045
MAP7	-2.53114	6.932597	-11.8864	2.44E-21	2.82E-20	37.70169
INPP1	-3.34469	7.974296	-11.8849	2.46E-21	2.84E-20	37.69417
GPX4	-2.54859	10.85576	-11.864	2.74E-21	3.15E-20	37.58573
TESC	-2.79754	6.765524	-11.8487	2.97E-21	3.39E-20	37.50661
DPY19L2P2	-2.74082	4.728539	-11.8397	3.11E-21	3.54E-20	37.45973
NAA35	-2.33493	6.480028	-11.8052	3.72E-21	4.20E-20	37.28144
PVRL3	-3.40779	5.526986	-11.7792	4.26E-21	4.78E-20	37.1466
POMT1	-2.20629	7.226705	-11.7613	4.67E-21	5.23E-20	37.05371
GOT1	-2.31352	7.745979	-11.7602	4.70E-21	5.25E-20	37.04818
PINK1	-2.65566	8.853122	-11.7368	5.31E-21	5.92E-20	36.92692
FAM178A	-2.35843	7.021474	-11.73	5.50E-21	6.12E-20	36.89142
RANGRF	-2.09781	8.363686	-11.7264	5.60E-21	6.23E-20	36.8726
STAT4	-2.59512	7.480874	-11.7163	5.91E-21	6.54E-20	36.82037
WT1	-3.53095	6.068619	-11.7043	6.29E-21	6.95E-20	36.75802
EHBP1	-2.18751	7.467853	-11.7013	6.38E-21	7.05E-20	36.74265
TOMM34	-2.12925	9.430301	-11.6839	6.99E-21	7.70E-20	36.65233
IPP	-2.23592	5.539766	-11.6804	7.12E-21	7.84E-20	36.63411
ZCCHC10	-2.09489	6.100868	-11.6619	7.84E-21	8.59E-20	36.53818
BNC1	-2.65424	5.755897	-11.6457	8.52E-21	9.30E-20	36.4544
KCNH2	-2.5403	7.622984	-11.6339	9.06E-21	9.87E-20	36.3929
CCDC53	-3.54073	8.185322	-11.6252	9.48E-21	1.03E-19	36.34801
NBR1	-2.1574	8.447522	-11.5428	1.46E-20	1.56E-19	35.91993
FBXL2	-2.42854	6.979617	-11.5166	1.67E-20	1.78E-19	35.78396
ACE2	-5.56901	5.709315	-11.4994	1.82E-20	1.94E-19	35.69447
LRRC23	-2.49715	7.407576	-11.4871	1.95E-20	2.06E-19	35.63064
CLEC16A	-2.04306	7.187924	-11.4572	2.27E-20	2.39E-19	35.47527

GUCA1A	-2.91915	6.527289	-11.4526	2.33E-20	2.45E-19	35.45133
CCNJL	-2.09338	6.721314	-11.437	2.53E-20	2.64E-19	35.37004
OXSM	-2.11001	7.472456	-11.4337	2.57E-20	2.68E-19	35.35287
NAA16	-2.37553	7.047833	-11.4336	2.57E-20	2.68E-19	35.35239
DHRS7B	-2.17375	7.350935	-11.4214	2.74E-20	2.86E-19	35.28906
CETN3	-4.12257	7.757154	-11.3899	3.23E-20	3.36E-19	35.12523
FBXW7	-2.03197	7.026739	-11.3866	3.28E-20	3.41E-19	35.10809
DR1	-2.02157	7.162363	-11.3705	3.57E-20	3.70E-19	35.02432
GLT8D1	-2.33682	7.593765	-11.3697	3.59E-20	3.71E-19	35.02014
IFT81	-2.79741	7.008297	-11.3685	3.61E-20	3.73E-19	35.01376
PDE1A	-3.22937	6.13915	-11.3575	3.82E-20	3.94E-19	34.95654
SCRG1	-5.52242	6.379317	-11.3511	3.95E-20	4.07E-19	34.92346
CMC2	-2.36581	8.716367	-11.3462	4.05E-20	4.17E-19	34.89771
ENOX1	-2.11973	5.312768	-11.346	4.06E-20	4.17E-19	34.89655
PDZD2	-4.15419	6.046142	-11.3375	4.24E-20	4.35E-19	34.85264
TRIM68	-2.14302	7.263476	-11.3296	4.42E-20	4.53E-19	34.81128
CALB2	-3.17209	6.504433	-11.3039	5.06E-20	5.16E-19	34.67746
OR7E37P	-2.07566	8.111595	-11.2818	5.67E-20	5.78E-19	34.56247
PKMYT1	-2.20687	8.476455	-11.2719	5.98E-20	6.06E-19	34.51087
WASF1	-3.80762	7.116781	-11.2564	6.48E-20	6.56E-19	34.43055
MLF1	-4.10484	7.730168	-11.2434	6.93E-20	7.01E-19	34.36245
PSMF1	-2.59182	7.896949	-11.2337	7.29E-20	7.35E-19	34.31186
KLHDC10	-2.55735	7.168686	-11.2199	7.84E-20	7.88E-19	34.24012
ERI2	-2.39505	6.34856	-11.1927	9.03E-20	9.06E-19	34.09848
LPIN1	-2.18443	7.411529	-11.1714	1.01E-19	1.01E-18	33.98751
GLUD2	-2.56116	6.413646	-11.1655	1.04E-19	1.04E-18	33.95663
KATNA1	-2.67407	7.632921	-11.1547	1.10E-19	1.09E-18	33.90037
AKTIP	-2.35125	8.327146	-11.1388	1.20E-19	1.18E-18	33.81751
TEX11	-2.08702	6.224687	-11.1332	1.23E-19	1.21E-18	33.78865
MBD2	-2.03065	6.842266	-11.1196	1.32E-19	1.30E-18	33.7178
CUL9	-2.00341	7.537639	-11.1191	1.33E-19	1.30E-18	33.71513
MAPK13	-2.6647	7.28666	-11.0667	1.74E-19	1.70E-18	33.44195
SCCPDH	-2.89751	7.8162	-11.0563	1.84E-19	1.79E-18	33.38787
MAMLD1	-2.9598	6.821176	-11.0527	1.88E-19	1.83E-18	33.36883
ANAPC10	-2.2611	6.363387	-11.0432	1.97E-19	1.92E-18	33.31943
EFHD2	-2.84827	8.123282	-11.0139	2.30E-19	2.23E-18	33.16668
HSD17B6	-2.59293	5.541565	-10.9941	2.55E-19	2.46E-18	33.06304
MEA1	-2.3344	10.05226	-10.9917	2.58E-19	2.48E-18	33.05075
SLC12A2	-2.23712	7.56787	-10.9773	2.78E-19	2.67E-18	32.9754
DERL2	-2.00682	8.434809	-10.9768	2.79E-19	2.67E-18	32.97289
CELSR2	-2.32426	7.487868	-10.9384	3.41E-19	3.24E-18	32.77275
CHIC2	-3.20156	8.014368	-10.9341	3.49E-19	3.31E-18	32.75012
RAB8B	-2.83383	7.301864	-10.9329	3.51E-19	3.33E-18	32.74389
CUL1	-2.1347	8.819086	-10.898	4.22E-19	3.96E-18	32.56176
PITRM1	-2.07299	8.370322	-10.8896	4.40E-19	4.13E-18	32.51812
RUVBL2	-2.49521	9.743672	-10.8893	4.41E-19	4.13E-18	32.51651
CEP76	-2.66089	6.303607	-10.8888	4.42E-19	4.14E-18	32.51357
TMEM8A	-2.69542	8.112835	-10.8801	4.63E-19	4.31E-18	32.46864
TBC1D8B	-2.3456	6.127877	-10.8778	4.69E-19	4.36E-18	32.45645

PRND	-5.16452	7.195613	-10.8774	4.69E-19	4.36E-18	32.45446
SECISBP2	-2.11635	7.577382	-10.8763	4.72E-19	4.38E-18	32.44844
SCML1	-3.68784	6.470735	-10.8616	5.10E-19	4.72E-18	32.37201
ME1	-2.10969	5.90673	-10.8605	5.13E-19	4.75E-18	32.36607
FOXG1	-2.72485	6.006477	-10.8565	5.24E-19	4.84E-18	32.34504
CDKN3	-4.16657	7.766756	-10.8515	5.38E-19	4.96E-18	32.31889
DDA1	-2.43356	7.413907	-10.8425	5.64E-19	5.19E-18	32.27206
FAF1	-2.13569	9.032568	-10.7992	7.07E-19	6.44E-18	32.04613
CRYZL1	-2.40522	6.461971	-10.7957	7.20E-19	6.55E-18	32.02773
EIF4ENIF1	-2.39623	7.949852	-10.7913	7.37E-19	6.70E-18	32.00489
EFHC1	-3.43146	6.831518	-10.7851	7.61E-19	6.92E-18	31.97234
KATNB1	-2.03036	7.195152	-10.7653	8.44E-19	7.66E-18	31.86908
TCEAL2	-4.80787	5.526902	-10.7434	9.47E-19	8.55E-18	31.75451
SOX3	-2.13685	5.471566	-10.7378	9.75E-19	8.79E-18	31.7253
HPCAL1	-2.51778	7.809094	-10.7315	1.01E-18	9.06E-18	31.69249
EXOC5	-3.81761	5.768183	-10.7212	1.06E-18	9.51E-18	31.6389
PAQR3	-2.49416	5.714011	-10.7191	1.08E-18	9.61E-18	31.6281
LIAS	-2.41428	6.352193	-10.6842	1.29E-18	1.14E-17	31.4456
RBKS	-2.08611	6.956408	-10.6689	1.40E-18	1.24E-17	31.36552
PCDH17	-2.74308	8.118123	-10.6603	1.46E-18	1.29E-17	31.32075
CITED1	-4.45785	7.413577	-10.6401	1.63E-18	1.43E-17	31.21542
UFSP2	-2.50678	7.429797	-10.6004	2.00E-18	1.75E-17	31.00777
FAM179B	-2.38192	6.738543	-10.5931	2.08E-18	1.82E-17	30.96963
TRIP12	-2.31394	10.01711	-10.591	2.10E-18	1.84E-17	30.9587
PAK3	-2.1617	7.0308	-10.5159	3.12E-18	2.68E-17	30.56615
DRG1	-2.05396	10.32891	-10.505	3.30E-18	2.83E-17	30.50951
UBE2M	-2.00908	8.193388	-10.4983	3.42E-18	2.93E-17	30.47468
NAALAD2	-2.67255	4.889647	-10.4708	3.95E-18	3.36E-17	30.33089
NXT2	-2.25109	7.005562	-10.4323	4.83E-18	4.05E-17	30.12978
ST6GALNA	-3.38491	7.645495	-10.4213	5.12E-18	4.28E-17	30.07195
CLCN3	-2.25451	7.233094	-10.4108	5.41E-18	4.51E-17	30.01713
PLXNC1	-2.22352	6.197943	-10.3918	5.98E-18	4.96E-17	29.91814
FAM13B	-2.77895	7.697355	-10.3874	6.12E-18	5.08E-17	29.89476
ORC3	-2.297	7.386033	-10.3782	6.42E-18	5.32E-17	29.847
FOXD1	-2.51	5.95833	-10.3642	6.91E-18	5.71E-17	29.77357
SPHK2	-2.02554	7.1982	-10.3386	7.90E-18	6.50E-17	29.63994
LRRC16A	-2.24163	6.935584	-10.3153	8.93E-18	7.31E-17	29.51803
MTCH2	-2.34605	9.883861	-10.3068	9.34E-18	7.63E-17	29.47351
HSPA2	-4.71122	8.160238	-10.3003	9.66E-18	7.88E-17	29.43955
PMS1	-3.33481	7.934786	-10.2992	9.71E-18	7.92E-17	29.43417
PSENEN	-2.3083	8.143112	-10.2765	1.09E-17	8.89E-17	29.31525
IDH3A	-2.16828	7.268546	-10.2472	1.28E-17	1.03E-16	29.16253
SNAPC1	-3.21169	6.291715	-10.2441	1.30E-17	1.05E-16	29.146
LGALS8	-2.01464	6.546898	-10.2438	1.30E-17	1.05E-16	29.14481
GOLGA8N	-3.35705	8.679355	-10.1963	1.67E-17	1.33E-16	28.89654
EXD2	-2.27367	7.641176	-10.1885	1.74E-17	1.38E-16	28.85574
PELI2	-2.05061	6.85588	-10.1859	1.76E-17	1.40E-16	28.84211
C9orf40	-2.09935	5.4291	-10.1802	1.81E-17	1.44E-16	28.81235
KIZ	-2.79738	8.290273	-10.166	1.95E-17	1.55E-16	28.7382

MAGEA1	-2.07643	5.000747	-10.1552	2.07E-17	1.64E-16	28.68161
NBPF1	-2.41656	10.3121	-10.1429	2.20E-17	1.74E-16	28.61727
OTUD3	-2.12731	7.116203	-10.1222	2.46E-17	1.93E-16	28.50915
PIGF	-2.34137	7.335108	-10.1198	2.49E-17	1.95E-16	28.49695
LANCL1	-2.0336	8.497319	-10.0788	3.08E-17	2.40E-16	28.28259
SAC3D1	-3.11043	6.980867	-10.0591	3.42E-17	2.65E-16	28.17951
TEX264	-2.01055	7.560424	-10.0565	3.47E-17	2.68E-16	28.16589
WDR62	-2.20099	7.57003	-10.0326	3.93E-17	3.03E-16	28.04118
SNX16	-2.06931	5.294422	-10.0182	4.24E-17	3.25E-16	27.96596
CYB5R4	-2.45926	6.492716	-10.0041	4.56E-17	3.49E-16	27.89245
ASTN2	-2.74395	6.031523	-9.99912	4.68E-17	3.58E-16	27.86635
S100PBP	-2.7282	7.698309	-9.99601	4.76E-17	3.63E-16	27.85009
BCL2L14	-2.85606	7.38284	-9.99322	4.83E-17	3.68E-16	27.83554
UCHL3	-2.677	8.23552	-9.97607	5.29E-17	4.01E-16	27.74599
CYP26B1	-3.02589	6.018412	-9.95835	5.80E-17	4.38E-16	27.65347
GMCL1	-3.34836	6.818201	-9.91574	7.25E-17	5.43E-16	27.43098
USP25	-2.42947	8.287736	-9.91212	7.39E-17	5.52E-16	27.4121
JADE3	-2.45083	6.882776	-9.90567	7.65E-17	5.70E-16	27.37839
MKKS	-2.32993	7.513158	-9.89689	8.00E-17	5.97E-16	27.33258
LRRC6	-3.206	6.926246	-9.88886	8.35E-17	6.21E-16	27.29067
CCDC92	-3.23406	8.8057	-9.88829	8.37E-17	6.23E-16	27.28771
KLHL7	-2.93581	6.680079	-9.88311	8.60E-17	6.39E-16	27.26062
PBK	-4.11425	6.750395	-9.87652	8.91E-17	6.61E-16	27.22623
WHSC1	-2.29249	7.355711	-9.87169	9.14E-17	6.77E-16	27.20101
RPP38	-2.0723	8.268762	-9.86797	9.31E-17	6.90E-16	27.1816
MAGEB1	-3.41118	5.234599	-9.84668	1.04E-16	7.67E-16	27.07048
HDGFRP3	-2.02215	6.978299	-9.83946	1.08E-16	7.94E-16	27.03283
UPF2	-2.13684	7.793073	-9.82707	1.15E-16	8.45E-16	26.96815
C3orf14	-2.51846	7.923441	-9.82455	1.17E-16	8.55E-16	26.95501
OARD1	-2.79728	8.453244	-9.80517	1.29E-16	9.41E-16	26.85388
ME3	-2.26854	6.905687	-9.76256	1.62E-16	1.17E-15	26.63158
MRPS15	-2.09986	7.608518	-9.7328	1.89E-16	1.36E-15	26.47637
SECISBP2L	-2.01694	6.842973	-9.72878	1.93E-16	1.38E-15	26.45539
ZNF200	-2.4583	7.417871	-9.71792	2.04E-16	1.46E-15	26.39879
ZBTB18	-2.26067	6.648438	-9.70073	2.24E-16	1.59E-15	26.30914
PSMG1	-3.39061	7.928058	-9.70054	2.24E-16	1.59E-15	26.30812
AGO3	-2.57651	6.735115	-9.69665	2.29E-16	1.62E-15	26.28787
HSPBAP1	-2.38528	7.015492	-9.68868	2.38E-16	1.69E-15	26.24632
TTYH1	-2.82803	6.245635	-9.67842	2.51E-16	1.78E-15	26.19282
FAM208B	-2.47252	7.346785	-9.67329	2.58E-16	1.83E-15	26.16606
SLC6A1	-2.03499	4.904654	-9.66913	2.64E-16	1.87E-15	26.14439
SERPINB6	-2.44639	8.992268	-9.66654	2.68E-16	1.89E-15	26.13087
NDRG4	-3.71552	7.222099	-9.66344	2.72E-16	1.92E-15	26.11469
PPP2R3C	-2.82478	7.294466	-9.64828	2.94E-16	2.07E-15	26.03567
ANKRD27	-2.43188	7.576918	-9.64132	3.05E-16	2.15E-15	25.9994
AZGP1	-2.54443	4.933709	-9.6344	3.17E-16	2.22E-15	25.96336
SLC4A8	-2.83905	5.404658	-9.62136	3.39E-16	2.37E-15	25.8954
ATAD2	-2.67543	6.363684	-9.60534	3.69E-16	2.57E-15	25.8119
PENK	-4.53889	6.859934	-9.60083	3.77E-16	2.63E-15	25.78841

TCEA2	-2.89798	7.423572	-9.59217	3.95E-16	2.74E-15	25.74327
IFT57	-3.2223	7.007882	-9.57133	4.40E-16	3.04E-15	25.6347
DUSP22	-2.09452	8.752755	-9.56154	4.64E-16	3.19E-15	25.5837
FDXR	-2.80379	7.132537	-9.56142	4.64E-16	3.19E-15	25.58311
CLK4	-2.39771	7.240475	-9.55282	4.85E-16	3.33E-15	25.53831
RBBP8	-2.59294	8.24833	-9.55045	4.91E-16	3.37E-15	25.52594
GAS2L1	-2.07383	7.469758	-9.53797	5.24E-16	3.59E-15	25.46093
GPR18	-3.81334	5.569092	-9.53076	5.45E-16	3.73E-15	25.4234
AGPAT5	-2.12427	7.92709	-9.51478	5.92E-16	4.04E-15	25.34021
BAZ2B	-2.6702	7.978023	-9.50029	6.39E-16	4.34E-15	25.26476
RNF19B	-2.48818	8.544434	-9.49215	6.66E-16	4.52E-15	25.22241
FAM118A	-2.27433	6.821484	-9.48893	6.78E-16	4.59E-15	25.2056
DHX30	-2.00697	8.317692	-9.48578	6.89E-16	4.66E-15	25.18923
INHBB	-2.72563	7.803408	-9.47584	7.26E-16	4.90E-15	25.13747
RNF139	-2.26868	8.580242	-9.47244	7.39E-16	4.98E-15	25.1198
MAGEA11	-2.18498	4.880828	-9.46074	7.85E-16	5.28E-15	25.05891
BAZ1A	-3.1364	6.888605	-9.45794	7.97E-16	5.35E-15	25.04434
GSTM3	-5.24211	8.315584	-9.45489	8.10E-16	5.43E-15	25.02843
CAMLG	-2.21396	9.151725	-9.41346	1.01E-15	6.70E-15	24.81289
DBF4	-3.72518	7.26733	-9.40929	1.03E-15	6.84E-15	24.79119
TEX2	-2.08875	8.688247	-9.40225	1.07E-15	7.09E-15	24.75457
C1orf112	-2.54982	5.754484	-9.38571	1.16E-15	7.73E-15	24.66857
CAND2	-3.24966	6.991453	-9.38216	1.18E-15	7.87E-15	24.6501
SEPT10	-2.41195	7.343271	-9.35799	1.34E-15	8.91E-15	24.52444
KIF2C	-2.83644	8.168897	-9.32766	1.57E-15	1.04E-14	24.36681
MPC2	-2.59202	9.624644	-9.32545	1.59E-15	1.05E-14	24.35528
GLRX2	-2.19444	7.877584	-9.30816	1.74E-15	1.14E-14	24.26545
FZD3	-2.10144	4.998201	-9.26209	2.22E-15	1.44E-14	24.02618
MYOM2	-3.46171	5.964066	-9.2436	2.44E-15	1.57E-14	23.93016
COPS3	-2.16914	9.095187	-9.22991	2.62E-15	1.69E-14	23.85906
CLPX	-2.29764	6.262806	-9.22876	2.64E-15	1.69E-14	23.85314
POT1	-2.03	7.463872	-9.2045	2.99E-15	1.92E-14	23.72721
SPAG16	-2.71902	6.39743	-9.19985	3.07E-15	1.96E-14	23.70307
GULP1	-2.29084	6.286383	-9.17748	3.45E-15	2.19E-14	23.58703
DMRT1	-5.04921	5.777263	-9.15728	3.83E-15	2.43E-14	23.48224
C11orf49	-2.08519	7.721854	-9.14412	4.10E-15	2.60E-14	23.41402
NCAPH	-2.26329	7.781091	-9.14164	4.16E-15	2.63E-14	23.40116
ZCCHC2	-2.60049	6.445348	-9.1406	4.18E-15	2.64E-14	23.39577
BCCIP	-2.29789	7.33011	-9.13286	4.35E-15	2.74E-14	23.35561
KLK11	-2.4522	7.061603	-9.11909	4.67E-15	2.94E-14	23.28423
17-Nov	-2.15436	7.08807	-9.11657	4.74E-15	2.97E-14	23.27117
ISYNA1	-3.20877	8.121563	-9.10764	4.96E-15	3.10E-14	23.22487
DYNC1LI1	-2.42043	7.324287	-9.10644	4.99E-15	3.12E-14	23.21868
ALDH9A1	-2.1442	9.401508	-9.105	5.03E-15	3.14E-14	23.2112
DAZL	-5.53438	5.62249	-9.04239	6.97E-15	4.29E-14	22.88688
GTF2A2	-2.60757	9.678802	-9.03557	7.22E-15	4.44E-14	22.85161
RPL39L	-2.86658	7.872875	-9.01789	7.92E-15	4.85E-14	22.76007
FAM98A	-2.62002	8.86514	-9.01525	8.03E-15	4.91E-14	22.74641
KDM5B	-2.02799	8.159107	-9.00209	8.60E-15	5.24E-14	22.6783

TRMT1L	-2.18367	5.807997	-8.99907	8.73E-15	5.31E-14	22.6627
PSMD6	-2.10222	10.25866	-8.99696	8.83E-15	5.36E-14	22.65177
TBC1D15	-2.06899	7.804095	-8.98652	9.32E-15	5.65E-14	22.59776
CYP17A1	-3.22905	6.976927	-8.98607	9.35E-15	5.66E-14	22.59541
SAR1B	-2.69938	6.901569	-8.97226	1.00E-14	6.06E-14	22.524
PPARGC1A	-2.23171	5.052053	-8.97084	1.01E-14	6.10E-14	22.51664
ZNF516	-2.30812	5.797574	-8.95658	1.09E-14	6.54E-14	22.44291
FBXO3	-2.63843	6.459514	-8.9465	1.15E-14	6.87E-14	22.39079
EPB41L3	-2.86228	6.077781	-8.92829	1.26E-14	7.52E-14	22.29665
SEC61A2	-2.04215	6.794329	-8.92825	1.26E-14	7.52E-14	22.29646
MTA1	-2.00587	7.344095	-8.91314	1.37E-14	8.09E-14	22.21837
TUBG1	-3.24385	8.718989	-8.90708	1.41E-14	8.33E-14	22.18708
TMX4	-2.02779	7.209899	-8.90134	1.45E-14	8.57E-14	22.15742
NUCB2	-2.83555	7.926185	-8.89886	1.47E-14	8.68E-14	22.14459
NXF3	-4.02311	6.579229	-8.88405	1.59E-14	9.34E-14	22.06812
ACAT1	-2.38809	9.228279	-8.86866	1.72E-14	1.01E-13	21.98863
ASRGL1	-3.16158	9.178003	-8.86453	1.76E-14	1.03E-13	21.9673
CROCCP2	-2.07689	7.563855	-8.85096	1.89E-14	1.10E-13	21.89728
GPATCH2	-2.41758	7.241305	-8.84274	1.97E-14	1.15E-13	21.85485
ALG8	-2.20098	8.757474	-8.84151	1.98E-14	1.15E-13	21.84848
DPP8	-2.55467	8.43996	-8.81728	2.25E-14	1.30E-13	21.72349
G6PD	-2.01721	8.406353	-8.81708	2.25E-14	1.30E-13	21.72244
TPK1	-2.1472	6.208611	-8.75095	3.17E-14	1.80E-13	21.38152
SPATA20	-2.52975	9.035418	-8.74309	3.30E-14	1.87E-13	21.34103
ZNF318	-2.27159	7.307121	-8.74086	3.34E-14	1.89E-13	21.32955
MICAL2	-2.00934	7.182394	-8.73603	3.42E-14	1.93E-13	21.30467
HSD17B3	-2.92794	6.849714	-8.73211	3.49E-14	1.97E-13	21.28448
PPM1G	-2.12047	9.18293	-8.7314	3.51E-14	1.97E-13	21.28082
SNAP29	-2.00105	8.451324	-8.7257	3.61E-14	2.03E-13	21.2515
DAAM2	-2.28518	7.244164	-8.71905	3.74E-14	2.10E-13	21.21727
GYG1	-2.75772	9.387592	-8.70521	4.02E-14	2.25E-13	21.14602
SH3GL2	-2.23994	4.946381	-8.70153	4.09E-14	2.29E-13	21.12703
KLK5	-2.09104	6.106325	-8.69184	4.30E-14	2.41E-13	21.0772
MYO6	-2.20202	6.803444	-8.68547	4.45E-14	2.48E-13	21.0444
IL13RA2	-4.39636	6.202259	-8.6687	4.85E-14	2.70E-13	20.95811
PLP1	-3.71324	6.515239	-8.65809	5.13E-14	2.84E-13	20.90357
DDX43	-3.7577	5.727629	-8.63387	5.81E-14	3.20E-13	20.77903
SAP130	-2.07947	8.526207	-8.62284	6.15E-14	3.38E-13	20.72237
P4HTM	-2.06699	7.837344	-8.56316	8.38E-14	4.56E-13	20.41591
DNAJB9	-2.06317	7.066345	-8.56099	8.47E-14	4.61E-13	20.40473
STX8	-2.18747	8.574107	-8.54016	9.43E-14	5.09E-13	20.29787
OPTN	-2.18369	7.7864	-8.5384	9.52E-14	5.13E-13	20.28885
DACH1	-2.20715	5.238746	-8.52723	1.01E-13	5.42E-13	20.23158
FAM193A	-2.18534	8.711276	-8.51622	1.07E-13	5.72E-13	20.17509
TUBB4B	-2.3158	11.18815	-8.50911	1.11E-13	5.92E-13	20.13869
IFT20	-2.0684	8.474468	-8.49248	1.21E-13	6.42E-13	20.05344
MFF	-2.82016	7.758411	-8.47172	1.34E-13	7.12E-13	19.9471
RPS6KC1	-2.17271	7.811904	-8.47009	1.35E-13	7.17E-13	19.93876
LZTFL1	-2.71591	7.217201	-8.44534	1.54E-13	8.10E-13	19.81204

LPPR4	-2.28011	5.622485	-8.42991	1.67E-13	8.76E-13	19.73306
CHPT1	-2.29895	8.449571	-8.42131	1.74E-13	9.14E-13	19.68907
CLDND1	-2.65287	7.841659	-8.38666	2.08E-13	1.08E-12	19.51192
LRRN3	-2.96795	7.103697	-8.38011	2.15E-13	1.12E-12	19.47843
APPL2	-2.21083	6.354928	-8.35731	2.42E-13	1.25E-12	19.36196
MAGEH1	-2.44923	8.005143	-8.34881	2.53E-13	1.30E-12	19.31858
NUP155	-2.94749	8.058575	-8.33377	2.73E-13	1.40E-12	19.24179
ORC2	-2.42639	7.190769	-8.33287	2.75E-13	1.41E-12	19.23718
SPDL1	-2.33877	7.174732	-8.33031	2.78E-13	1.42E-12	19.22411
SMC6	-2.30765	8.55665	-8.30937	3.10E-13	1.58E-12	19.11731
INPP5A	-2.04016	8.980676	-8.30105	3.23E-13	1.64E-12	19.07488
SPECC1L	-2.00441	8.500607	-8.24676	4.27E-13	2.14E-12	18.79819
MYL6B	-2.63512	9.424729	-8.23432	4.55E-13	2.27E-12	18.73485
UAP1	-2.68945	9.092888	-8.22147	4.86E-13	2.42E-12	18.66948
NCAPG2	-2.24636	7.584476	-8.19161	5.67E-13	2.80E-12	18.51756
PTBP2	-2.26269	7.762618	-8.17492	6.18E-13	3.04E-12	18.43272
PDP1	-2.22364	7.515419	-8.14541	7.18E-13	3.51E-12	18.28283
IPO4	-2.05885	7.85957	-8.13884	7.43E-13	3.63E-12	18.24948
PON3	-2.52524	7.299418	-8.1317	7.70E-13	3.76E-12	18.21325
TKTL1	-5.10202	6.972325	-8.12745	7.87E-13	3.84E-12	18.19165
TAF5	-2.43888	7.528827	-8.12702	7.89E-13	3.84E-12	18.18948
ZWILCH	-3.34558	7.48216	-8.10604	8.78E-13	4.27E-12	18.08305
FBXL5	-2.02932	8.187182	-8.10421	8.87E-13	4.30E-12	18.07375
KDM3A	-2.16869	8.980192	-8.09063	9.50E-13	4.60E-12	18.00488
CCDC90B	-2.07798	8.670911	-8.07934	1.01E-12	4.85E-12	17.94767
PRKCZ	-2.78246	8.351493	-8.06893	1.06E-12	5.09E-12	17.89494
C16orf45	-2.40727	7.044653	-8.06892	1.06E-12	5.09E-12	17.8949
CYB5R1	-2.33501	8.678874	-8.06028	1.11E-12	5.31E-12	17.85112
TRAPPC8	-2.47542	9.301498	-8.05104	1.16E-12	5.55E-12	17.8043
USP32P2	-3.01228	8.054174	-8.03027	1.29E-12	6.15E-12	17.6992
EPHA4	-2.13172	7.931846	-8.02665	1.32E-12	6.25E-12	17.68089
REEP1	-3.6226	8.303914	-8.01817	1.38E-12	6.51E-12	17.63795
DNAJB1	-2.37681	9.113874	-8.01517	1.40E-12	6.60E-12	17.62277
GATM	-3.43404	6.55153	-8.01165	1.42E-12	6.72E-12	17.605
VLDLR	-2.16187	5.961541	-8.00275	1.49E-12	7.01E-12	17.56001
SERPINA5	-4.86851	8.263411	-7.98743	1.61E-12	7.56E-12	17.48253
SYPL1	-2.25911	8.846296	-7.97424	1.72E-12	8.07E-12	17.4159
LIPT1	-2.08398	7.222402	-7.95818	1.87E-12	8.74E-12	17.33482
SLC47A1	-3.08673	7.251039	-7.93151	2.14E-12	9.95E-12	17.20021
LRIF1	-2.84871	6.926963	-7.91493	2.33E-12	1.08E-11	17.11663
BAG2	-2.65636	6.212356	-7.88346	2.73E-12	1.26E-11	16.95803
SLC27A6	-2.86141	5.284249	-7.87638	2.83E-12	1.30E-11	16.92241
DMTF1	-2.14593	8.485447	-7.84388	3.34E-12	1.52E-11	16.75884
KIFAP3	-2.57147	8.033704	-7.8241	3.69E-12	1.67E-11	16.6594
PPFIBP2	-2.70124	7.343505	-7.81676	3.83E-12	1.73E-11	16.62253
PLEKHO1	-2.1954	8.007821	-7.81003	3.97E-12	1.79E-11	16.58874
GSTA1	-2.44237	7.207584	-7.80352	4.10E-12	1.84E-11	16.55603
DDX3Y	-2.34331	7.434055	-7.80308	4.11E-12	1.85E-11	16.5538
BBX	-2.30523	8.822519	-7.79097	4.37E-12	1.96E-11	16.493

AGPS	-2.57493	7.259776	-7.77814	4.66E-12	2.08E-11	16.42862
HMMR	-2.15694	7.596656	-7.76809	4.90E-12	2.19E-11	16.37822
HSPA14	-2.37203	7.983952	-7.76631	4.95E-12	2.21E-11	16.36927
CENPU	-3.9127	8.226557	-7.75858	5.15E-12	2.29E-11	16.33053
RNF219	-2.05834	6.603569	-7.74726	5.45E-12	2.42E-11	16.27376
YIPF5	-2.07146	6.584684	-7.73789	5.71E-12	2.53E-11	16.22684
CAPRIN1	-2.2749	9.228806	-7.70974	6.59E-12	2.90E-11	16.08587
USP1	-2.3942	8.589212	-7.70942	6.60E-12	2.90E-11	16.08427
ARNT2	-2.3629	6.648159	-7.65789	8.56E-12	3.73E-11	15.8266
NEK2	-3.09326	7.104158	-7.65274	8.78E-12	3.82E-11	15.80086
VPS26A	-2.12621	9.585429	-7.6237	1.02E-11	4.40E-11	15.65592
SUV420H1	-2.01335	8.002622	-7.61063	1.09E-11	4.68E-11	15.59074
PTTG1	-2.38351	10.21653	-7.60576	1.11E-11	4.79E-11	15.56645
UBL3	-2.03673	7.62229	-7.58038	1.26E-11	5.41E-11	15.44002
ELAVL4	-2.00841	6.225927	-7.57663	1.29E-11	5.50E-11	15.42136
COPS4	-2.36502	8.626308	-7.5662	1.36E-11	5.79E-11	15.36942
ACSL4	-2.0381	5.740973	-7.55169	1.46E-11	6.21E-11	15.29721
PRKCDBP	-2.13885	7.257692	-7.54511	1.51E-11	6.40E-11	15.26449
EMC7	-2.04909	8.839924	-7.52812	1.64E-11	6.95E-11	15.18002
EMC2	-2.23571	7.799532	-7.52782	1.65E-11	6.96E-11	15.17853
MTCL1	-2.20488	7.507619	-7.52698	1.65E-11	6.98E-11	15.17436
WBSCR22	-2.29125	9.50891	-7.51661	1.74E-11	7.34E-11	15.12283
NDC80	-2.33869	6.180552	-7.5035	1.86E-11	7.81E-11	15.05774
DDX39A	-2.42822	9.340401	-7.47597	2.14E-11	8.93E-11	14.92119
PLXDC2	-2.12951	5.897319	-7.46761	2.23E-11	9.29E-11	14.87969
AASDHPPT	-2.00327	8.343483	-7.46665	2.24E-11	9.33E-11	14.87494
KTN1	-2.09874	9.307066	-7.45794	2.34E-11	9.72E-11	14.83181
JAM2	-2.64437	7.685143	-7.43932	2.57E-11	1.06E-10	14.73957
GPR56	-2.16247	7.784203	-7.39865	3.14E-11	1.29E-10	14.5384
SUCO	-2.11541	8.304891	-7.38817	3.31E-11	1.35E-10	14.4866
FDFT1	-2.0032	9.725815	-7.3805	3.44E-11	1.41E-10	14.44874
LOC10027:	-2.49925	7.420718	-7.3437	4.14E-11	1.67E-10	14.26717
SLBP	-2.50661	8.596062	-7.33897	4.24E-11	1.71E-10	14.24386
COMMD8	-2.19467	8.037542	-7.29872	5.18E-11	2.07E-10	14.04562
PRKRA	-2.18315	7.958351	-7.26501	6.12E-11	2.43E-10	13.8799
ACTL6A	-2.52885	7.089696	-7.24722	6.68E-11	2.65E-10	13.79255
OSR2	-3.38999	5.468096	-7.23986	6.93E-11	2.74E-10	13.75647
TIPIN	-2.42592	6.378712	-7.16066	1.03E-10	3.99E-10	13.3686
EIF1B	-2.29258	9.255576	-7.14494	1.11E-10	4.30E-10	13.2918
MAGEA4	-3.23023	5.142249	-7.10447	1.35E-10	5.20E-10	13.09439
TSPYL4	-2.38763	7.98285	-7.08512	1.49E-10	5.71E-10	13.00012
MTX2	-2.04407	8.29751	-7.0846	1.49E-10	5.72E-10	12.9976
KRT23	-3.50895	6.238397	-7.06732	1.63E-10	6.22E-10	12.91348
CTR9	-2.00341	8.32876	-7.06306	1.66E-10	6.35E-10	12.89274
18-Nov	-2.27572	7.673856	-7.05484	1.73E-10	6.60E-10	12.8528
ZBTB38	-2.08848	8.570724	-7.04583	1.81E-10	6.88E-10	12.80901
ASAP2	-2.98401	7.599704	-7.04162	1.85E-10	7.02E-10	12.78855
STK39	-2.5526	7.445985	-7.01759	2.08E-10	7.85E-10	12.67189
TUBA4A	-2.07796	7.910118	-7.00844	2.17E-10	8.19E-10	12.62751

NACC2	-2.09757	6.62777	-6.98301	2.46E-10	9.24E-10	12.50427
HPRT1	-2.34855	9.494672	-6.96477	2.69E-10	1.01E-09	12.41601
WDR47	-2.59241	7.286883	-6.93626	3.10E-10	1.15E-09	12.27817
TDRD7	-2.21576	7.829795	-6.87013	4.28E-10	1.56E-09	11.95931
TACC3	-2.13887	8.302321	-6.83048	5.19E-10	1.87E-09	11.76868
RRAGD	-2.01396	7.06936	-6.81757	5.52E-10	1.99E-09	11.70676
TMEM97	-2.1322	9.077304	-6.81179	5.68E-10	2.04E-09	11.67898
EZH2	-2.3915	6.838666	-6.81021	5.72E-10	2.06E-09	11.67143
FAM184A	-2.38164	7.739745	-6.80709	5.81E-10	2.09E-09	11.65646
WRB	-2.15444	8.539266	-6.76738	7.05E-10	2.51E-09	11.46628
ALDH1A1	-3.85654	7.003832	-6.72048	8.85E-10	3.11E-09	11.24221
HLTF	-2.56126	8.792803	-6.70448	9.56E-10	3.34E-09	11.16592
GMPR	-2.78716	7.58334	-6.6984	9.84E-10	3.44E-09	11.13695
FANCI	-2.04653	7.693094	-6.68968	1.03E-09	3.58E-09	11.09541
PNMAL1	-3.07742	7.670076	-6.67618	1.10E-09	3.80E-09	11.03119
DGAT1	-2.12843	7.281941	-6.60904	1.51E-09	5.19E-09	10.7124
TOPBP1	-2.22868	8.225667	-6.60856	1.52E-09	5.20E-09	10.71015
CEP70	-2.01066	7.300669	-6.56343	1.88E-09	6.38E-09	10.49668
SHCBP1	-2.01141	6.121678	-6.54919	2.02E-09	6.79E-09	10.42945
AMIGO2	-2.62041	7.104855	-6.47232	2.91E-09	9.65E-09	10.06759
TNFAIP6	-2.04876	6.404106	-6.46875	2.96E-09	9.81E-09	10.05083
ZFAND6	-2.01044	9.685287	-6.45304	3.19E-09	1.05E-08	9.977114
GSPT2	-2.1429	7.735853	-6.44991	3.24E-09	1.07E-08	9.962456
PTGDS	-3.93814	9.980781	-6.405	4.01E-09	1.31E-08	9.752264
MAP3K5	-2.04088	7.135865	-6.40479	4.02E-09	1.31E-08	9.751256
GFPT1	-2.05095	8.65783	-6.3354	5.58E-09	1.80E-08	9.427807
KIAA1598	-2.26937	7.057354	-6.32398	5.89E-09	1.89E-08	9.374726
INSL3	-4.42578	6.558794	-6.31018	6.29E-09	2.01E-08	9.310633
CDO1	-2.84059	6.633815	-6.27472	7.43E-09	2.36E-08	9.146257
PRPS2	-2.89243	7.600258	-6.26428	7.81E-09	2.47E-08	9.097929
SLC46A3	-2.09237	7.384887	-6.25791	8.04E-09	2.55E-08	9.068457
KNTC1	-2.01775	7.392082	-6.21054	1.00E-08	3.15E-08	8.849815
MANSC1	-2.14498	6.148295	-6.18559	1.13E-08	3.52E-08	8.734996
CNN1	-2.38906	7.699907	-6.1828	1.14E-08	3.57E-08	8.722148
NEFM	-3.18888	6.060853	-6.17155	1.21E-08	3.75E-08	8.670456
KLF11	-2.01567	7.951182	-6.15666	1.29E-08	4.00E-08	8.602121
SERPINI1	-2.19409	6.774631	-6.14825	1.34E-08	4.16E-08	8.563526
CPE	-2.83115	7.742035	-6.08894	1.77E-08	5.41E-08	8.2922
SMARCA1	-2.04022	7.197794	-6.0611	2.02E-08	6.13E-08	8.165291
PLK2	-2.92386	7.124713	-6.01996	2.44E-08	7.36E-08	7.978269
CLU	-3.6792	8.392444	-5.98929	2.81E-08	8.42E-08	7.839256
LOC72839:	-2.34093	7.992796	-5.98862	2.82E-08	8.44E-08	7.836217
NAP1L3	-2.78795	6.924249	-5.98037	2.93E-08	8.75E-08	7.798878
TUBB4A	-2.77533	7.63117	-5.97945	2.94E-08	8.78E-08	7.794717
PLA2G16	-2.43683	8.79257	-5.97056	3.06E-08	9.12E-08	7.754521
TIPARP	-2.34519	8.580721	-5.94695	3.42E-08	1.01E-07	7.647927
ID4	-2.01151	7.084823	-5.9073	4.10E-08	1.20E-07	7.469402
IMPA1	-2.16396	9.397146	-5.86775	4.91E-08	1.43E-07	7.291894
OIP5	-2.67838	7.592619	-5.84926	5.34E-08	1.55E-07	7.209155

PJA2	-2.04962	8.265318	-5.8395	5.59E-08	1.61E-07	7.16552
TSPYL5	-3.09596	8.445149	-5.82985	5.84E-08	1.68E-07	7.122386
DHRS11	-2.04565	6.955527	-5.81173	6.34E-08	1.82E-07	7.041569
SOX9	-3.72017	7.824546	-5.75956	8.04E-08	2.28E-07	6.809563
KIF5C	-2.48862	8.054207	-5.75796	8.09E-08	2.29E-07	6.80245
MAGEA12	-3.33174	5.650704	-5.7569	8.13E-08	2.31E-07	6.797746
RFWD3	-2.0142	6.28938	-5.75264	8.29E-08	2.35E-07	6.778876
GATA4	-2.55034	6.267544	-5.75024	8.38E-08	2.37E-07	6.768221
HOPX	-3.12422	7.68764	-5.71024	1.00E-07	2.82E-07	6.591224
ZBTB20	-2.07548	6.952077	-5.70154	1.04E-07	2.92E-07	6.552855
CCNB2	-2.3996	8.996931	-5.69427	1.08E-07	3.02E-07	6.520745
RASSF2	-2.17363	7.972304	-5.69123	1.09E-07	3.05E-07	6.507377
HMGCS2	-3.4625	7.37523	-5.66582	1.23E-07	3.40E-07	6.395496
TTK	-3.12199	7.650454	-5.65352	1.30E-07	3.59E-07	6.341404
AKAP12	-3.05388	9.52255	-5.64542	1.34E-07	3.72E-07	6.305852
PNMA1	-2.16532	9.212116	-5.62254	1.49E-07	4.10E-07	6.205545
CKM	-2.12539	5.959221	-5.61746	1.52E-07	4.18E-07	6.183308
STAR	-3.43244	6.97624	-5.5971	1.67E-07	4.56E-07	6.094274
ABCA8	-3.34075	6.586929	-5.58128	1.79E-07	4.87E-07	6.02522
SLC6A8	-2.1115	8.610079	-5.55607	2.00E-07	5.43E-07	5.915394
FHL1	-2.30185	7.314716	-5.5533	2.03E-07	5.49E-07	5.903328
SOBP	-2.56449	7.524569	-5.487	2.72E-07	7.27E-07	5.615877
PRKCB	-2.10393	6.719317	-5.40416	3.92E-07	1.03E-06	5.259512
MSMO1	-2.23483	7.788943	-5.39191	4.14E-07	1.08E-06	5.207081
SPOCK1	-2.64704	7.82393	-5.36121	4.73E-07	1.23E-06	5.075937
LIPG	-2.07789	6.106003	-5.35242	4.92E-07	1.28E-06	5.038511
MAGEA6	-3.92674	5.805993	-5.34962	4.98E-07	1.29E-06	5.026561
TSC22D1	-2.13656	10.07381	-5.32402	5.57E-07	1.44E-06	4.917714
CEP55	-2.36038	7.769067	-5.29576	6.30E-07	1.62E-06	4.797856
CADM1	-2.16643	8.30294	-5.2594	7.37E-07	1.88E-06	4.644279
SYNGR3	-2.02199	7.211632	-5.21794	8.82E-07	2.22E-06	4.469883
MOK	-2.60475	7.658229	-5.21017	9.12E-07	2.29E-06	4.437271
NPTX2	-3.00192	7.682805	-5.18681	1.01E-06	2.52E-06	4.339446
TOP2A	-2.52687	8.233182	-5.13744	1.25E-06	3.08E-06	4.133597
DONSON	-2.11241	7.169969	-5.12153	1.33E-06	3.28E-06	4.067517
PFKP	-2.00225	9.102797	-5.07612	1.62E-06	3.95E-06	3.879586
PRKAR2B	-2.39151	7.933922	-5.04172	1.87E-06	4.53E-06	3.737914
MAGEB2	-3.11128	6.67465	-4.99931	2.24E-06	5.37E-06	3.564103
NUDT11	-2.4089	8.944003	-4.96714	2.56E-06	6.09E-06	3.432814
CYP11A1	-2.579	6.463185	-4.96163	2.62E-06	6.23E-06	3.410383
SLC7A5	-2.0454	9.412779	-4.87702	3.73E-06	8.68E-06	3.06799
KCNV2	-2.37555	5.271977	-4.84155	4.32E-06	1.00E-05	2.925525
PLSCR4	-2.12207	7.93356	-4.8414	4.32E-06	1.00E-05	2.924931
NUPR1	-2.04787	7.690274	-4.82254	4.67E-06	1.08E-05	2.849472
TJP2	-2.41226	7.939721	-4.80978	4.93E-06	1.13E-05	2.798517
PRAME	-2.78973	7.839782	-4.80585	5.01E-06	1.15E-05	2.782846
CST3	-2.02111	10.56235	-4.75554	6.15E-06	1.40E-05	2.582957
HERC5	-2.92666	8.522217	-4.49515	1.75E-05	3.76E-05	1.570659
PRC1	-2.05255	8.549199	-4.43909	2.19E-05	4.64E-05	1.357785

PEG3	-2.81422	7.666541	-4.37964	2.76E-05	5.80E-05	1.134063
CKS2	-2.26252	9.644786	-4.36151	2.96E-05	6.18E-05	1.066245
DDAH1	-2.43731	9.085944	-4.3413	3.20E-05	6.66E-05	0.99088
PEG10	-2.07669	8.536305	-4.252	4.52E-05	9.21E-05	0.660858
PLAT	-2.21829	7.074586	-4.19488	5.62E-05	0.000113	0.452352
CPEB1	-2.27391	8.401875	-4.08797	8.39E-05	0.000165	0.067544
BUB1B	-2.09309	8.414691	-3.93537	0.000147	0.00028	-0.46907
SERPINF1	-2.24983	10.00226	-3.76462	0.000272	0.0005	-1.05124
APOA1	-3.34534	8.452587	-3.74318	0.000293	0.000537	-1.12297
CTSV	-2.2188	9.189896	-3.69979	0.000341	0.00062	-1.26711
BEX1	-2.44118	9.625111	-3.23478	0.001616	0.002671	-2.7279

		TCGA DOWN-DEGs				Venn gene
AACSP1	ENSG0000(	0.03	6.99	-2.956	2.87E-78	SPACA1
AADACL2-	ENSG0000(	0.3	5.48	-2.317	4.96E-54	LINC00588
AANAT	ENSG0000(	0.31	6.63	-2.542	3.71E-62	RNFT1
AARD	ENSG0000(	2.72	26.13	-2.867	1.01E-45	RIBC2
AATBC	ENSG0000(	0.56	8.28	-2.573	1.45E-36	KPNA5
ABAT	ENSG0000(	3.38	17.019	-2.041	1.61E-35	ZNF280B
ABCA10	ENSG0000(	0.53	14.5	-3.341	2.82E-97	DNAI2
ABCA17P	ENSG0000(	0.68	76.478	-5.527	2.57E-90	NACA2
ABCA5	ENSG0000(	3.57	25.451	-2.533	9.80E-69	MORC1
ABCA8	ENSG0000(	0.79	33.24	-4.258	1.03E-75	GPR137
ABCB11	ENSG0000(	0	3.55	-2.186	9.74E-52	ADAM18
ABCB6	ENSG0000(	13.47	60.989	-2.099	4.77E-54	ADGB
ABCB9	ENSG0000(	4.27	24.75	-2.289	4.96E-69	ADCY10
ABCC11	ENSG0000(	0.14	5.2	-2.443	4.42E-74	CCDC70
ABCC12	ENSG0000(	0.72	15.49	-3.261	3.41E-44	SPO11
ABCG4	ENSG0000(	0.47	9.34	-2.814	6.03E-58	RNF32
ABHD1	ENSG0000(	0.44	131.297	-6.522	4.92E-86	C11orf63
ABHD16B	ENSG0000(	0	19.86	-4.383	1.79E-52	ADAM2
ABHD2	ENSG0000(	8.46	71.258	-2.933	3.40E-45	PAPOLB
ABHD8	ENSG0000(	3.63	58.569	-3.685	3.12E-67	LINC00837
ABLIM1	ENSG0000(	6.22	33.18	-2.243	1.09E-44	BTG4
ABO	ENSG0000(	0.27	8.83	-2.952	1.18E-69	DPY19L1P
AC000036.	ENSG0000(	0.05	13.12	-3.749	4.22E-57	LDHAL6B
AC000099.	ENSG0000(	0	3.28	-2.098	1.35E-42	SCIN
AC001226.	ENSG0000(	0.09	3.41	-2.016	9.03E-95	GLB1L
AC002056.	ENSG0000(	0	3.39	-2.134	1.20E-41	CEP112
AC002064.	ENSG0000(	0	15.63	-4.056	3.19E-66	ATXN3L
AC002115.	ENSG0000(	0	25.47	-4.726	1.84E-70	RPGRIP1
AC002117.	ENSG0000(	0	4.49	-2.457	1.55E-57	SYNGR4
AC002310.	ENSG0000(	0.6	20.031	-3.716	1.86E-71	SPATA1
AC002465.	ENSG0000(	0	5.89	-2.784	1.20E-51	SOX30
AC002467.	ENSG0000(	5.34	99.988	-3.994	1.36E-64	TEX12
AC003075.	ENSG0000(	0.24	12.35	-3.428	1.95E-78	CCDC88A
AC003688.	ENSG0000(	0	8.43	-3.237	2.45E-08	CYLC1
AC003973.	ENSG0000(	0	3.54	-2.183	5.54E-52	UBQLN3
AC003991.	ENSG0000(	0.18	4.67	-2.265	1.76E-48	GRK4
AC004009.	ENSG0000(	0	18.67	-4.298	6.42E-66	CCDC186
AC004070.	ENSG0000(	0	13.36	-3.844	9.23E-56	CYLC2
AC004221.	ENSG0000(	0.05	6.19	-2.776	4.47E-82	ERO1LB
AC004381.	ENSG0000(	4.06	279.653	-5.794	2.68E-83	GAPDHS
AC004448.	ENSG0000(	0.27	14.05	-3.567	3.24E-63	AURKC
AC004471.	ENSG0000(	0.1	3.47	-2.023	6.38E-77	GPC5
AC004510.	ENSG0000(	0	7.82	-3.141	1.10E-67	CARF
AC004623.	ENSG0000(	0.88	7.33	-2.148	5.21E-78	LINC00342
AC004692.	ENSG0000(	0	4.37	-2.425	8.10E-50	SPATA31C
AC004862.	ENSG0000(	0	5.35	-2.667	8.68E-46	C20orf195
AC004869.	ENSG0000(	0	4.04	-2.333	1.79E-57	C6orf10

AC005008. ENSG0000(	0	3.36	-2.124	5.65E-49	IQCK
AC005009. ENSG0000(	0	3.57	-2.192	3.22E-53	CAMK4
AC005076. ENSG0000(	3.81	33.77	-2.854	2.63E-48	ART3
AC005077. ENSG0000(	0.19	13.65	-3.622	3.18E-89	RFPL3S
AC005077. ENSG0000(	0	3.38	-2.131	1.38E-47	AKAP4
AC005324. ENSG0000(	0	3.07	-2.025	1.70E-50	CCHCR1
AC005324. ENSG0000(	0	7.42	-3.074	8.60E-46	IRGC
AC005391. ENSG0000(	0	4.5	-2.459	8.53E-49	TSSK2
AC005481. ENSG0000(	0.05	3.41	-2.07	4.46E-100	GK2
AC005522. ENSG0000(	0	7.33	-3.058	4.22E-61	NME8
AC005523. ENSG0000(	0.14	6.68	-2.752	1.98E-64	CBLN1
AC005754. ENSG0000(	0.08	70.722	-6.053	5.10E-76	CCIN
AC005808. ENSG0000(	0.16	4.83	-2.329	1.55E-42	USP2
AC006014. ENSG0000(	0.56	75.18	-5.61	2.88E-89	PPP1R2P9
AC006019. ENSG0000(	0.17	64.52	-5.807	1.50E-52	ADD3-AS1
AC006116. ENSG0000(	0.15	5.02	-2.388	3.36E-73	PRKAA1
AC006116. ENSG0000(	0.41	6.14	-2.34	3.98E-48	ALLC
AC006153. ENSG0000(	0	17.71	-4.226	4.23E-62	C16orf95
AC006272. ENSG0000(	0.21	5.45	-2.414	2.99E-81	TEX28
AC006277. ENSG0000(	0.51	11.29	-3.025	2.09E-61	MYT1L
AC006538. ENSG0000(	1.22	8.07	-2.03	2.79E-50	COIL
AC006547. ENSG0000(	0.15	63.53	-5.81	2.82E-80	CT62
AC006942. ENSG0000(	0.65	47.251	-4.87	7.93E-90	RFX2
AC006946. ENSG0000(	0.1	16.999	-4.032	5.48E-62	MROH7
AC007000. ENSG0000(	0	10.52	-3.526	2.10E-13	PRPS1L1
AC007009. ENSG0000(	0.01	5.67	-2.723	8.12E-58	TNP2
AC007040. ENSG0000(	0	3.52	-2.176	2.57E-50	C9orf9
AC007131. ENSG0000(	0	9.7	-3.42	9.59E-60	PSORS1C1
AC007163. ENSG0000(	0.03	7.13	-2.981	1.42E-63	GPR135
AC007249. ENSG0000(	0.23	7.15	-2.728	2.50E-53	HSPA1L
AC007391. ENSG0000(	0	6	-2.807	2.63E-54	TTLL7
AC007392. ENSG0000(	0	4.65	-2.498	3.34E-44	PMFBP1
AC007392. ENSG0000(	0	3.53	-2.18	1.84E-42	ZBTB32
AC007403. ENSG0000(	0	3.03	-2.011	7.39E-35	LRRC36
AC007463. ENSG0000(	0	6.15	-2.838	9.45E-53	FBXO24
AC007557. ENSG0000(	0	596.673	-9.223	5.73E-74	POM121L9
AC007557. ENSG0000(	0	7.58	-3.101	1.60E-57	AQP5
AC007557. ENSG0000(	0	8.98	-3.319	1.20E-55	ZC2HC1C
AC007557. ENSG0000(	0	16.93	-4.164	5.44E-61	KDM4D
AC007563. ENSG0000(	0	4.16	-2.367	4.87E-57	ZNRF4
AC007566. ENSG0000(	0.85	11.55	-2.762	2.13E-84	ACRV1
AC007879. ENSG0000(	0.97	9.93	-2.472	3.57E-47	ZSCAN5A
AC007879. ENSG0000(	0.34	6.35	-2.456	1.38E-56	CTAGE1
AC007966. ENSG0000(	0.04	3.58	-2.139	2.49E-37	NOL4
AC008073. ENSG0000(	0	6.65	-2.935	1.05E-76	LRRC48
AC008079. ENSG0000(	0	23.811	-4.633	1.57E-70	TSGA10
AC008088. ENSG0000(	0.66	34.811	-4.431	1.57E-73	RDH8
AC008132. ENSG0000(	0.04	192.951	-7.543	5.93E-88	CATSPERC
AC008132. ENSG0000(	0.03	11.64	-3.617	5.44E-65	APH1B

AC008427. ENSG0000(	0	8.32	-3.22	7.38E-53	GRAMD1C
AC008694. ENSG0000(	0	35.74	-5.199	1.38E-76	SLCO3A1
AC009120. ENSG0000(	0.11	4.59	-2.332	1.16E-69	SNX19
AC009120. ENSG0000(	4.18	22.33	-2.171	4.29E-66	MNS1
AC009133. ENSG0000(	0	4.07	-2.342	8.71E-19	SYCP2
AC009158. ENSG0000(	0	7.1	-3.018	4.58E-58	VPRBP
AC009299. ENSG0000(	0.09	10.79	-3.435	9.45E-60	LRIT1
AC009303. ENSG0000(	0	3.59	-2.199	7.91E-46	HIRIP3
AC009365. ENSG0000(	0	6.73	-2.95	3.34E-58	PACRG
AC009411. ENSG0000(	0	9.43	-3.383	4.04E-57	ZCCHC14
AC009473. ENSG0000(	0.15	10.6	-3.334	3.76E-68	IZUMO4
AC009487. ENSG0000(	0	41.859	-5.422	2.00E-68	TEX15
AC009505. ENSG0000(	0	3.2	-2.07	5.56E-47	TULP2
AC009506. ENSG0000(	1.59	9.66	-2.041	5.96E-49	C16orf71
AC010127. ENSG0000(	0.05	11.04	-3.519	9.06E-35	EFCAB6
AC010168. ENSG0000(	2.92	282.968	-6.179	4.18E-41	HCFC2
AC010524. ENSG0000(	0.4	8.68	-2.79	5.54E-79	PGK2
AC010649. ENSG0000(	0.25	28.479	-4.56	2.88E-61	ADAM5
AC010894. ENSG0000(	1.24	41.22	-4.236	2.50E-82	WDR78
AC010980. ENSG0000(	0.13	293.069	-8.024	1.45E-86	PRKACG
AC011196. ENSG0000(	0	4.3	-2.406	9.84E-46	TDRD1
AC011525. ENSG0000(	0	8.55	-3.256	1.30E-70	PDHA2
AC011718. ENSG0000(	0	21.18	-4.471	1.34E-66	CLPB
AC011752. ENSG0000(	0	7.28	-3.05	2.16E-52	ANKMY1
AC012075. ENSG0000(	0	6.86	-2.975	3.78E-68	DDX25
AC012370. ENSG0000(	0	19.95	-4.389	3.43E-66	ADAM30
AC012456. ENSG0000(	0	3.27	-2.094	5.83E-61	ACTL7B
AC012462. ENSG0000(	0	5.29	-2.653	2.71E-47	TAF7L
AC012594. ENSG0000(	0	51.14	-5.704	6.18E-69	SSX3
AC013271. ENSG0000(	1.37	114.126	-5.602	7.72E-58	CETN1
AC013460. ENSG0000(	0.02	6.95	-2.962	1.04E-52	RAB27B
AC013470. ENSG0000(	0	6.45	-2.897	1.48E-55	ZCWPW1
AC013472. ENSG0000(	0	9.33	-3.369	1.25E-65	LRP2BP
AC015849. ENSG0000(	0.96	11.2	-2.638	7.53E-43	SLC25A31
AC015849. ENSG0000(	0.11	8.95	-3.164	3.56E-74	LECT2
AC015922. ENSG0000(	0.1	6.29	-2.728	1.27E-59	ARHGAP3!
AC015971. ENSG0000(	0.1	6.68	-2.804	2.63E-66	TCP11
AC016582. ENSG0000(	0.39	7.5	-2.612	4.59E-58	AHI1
AC016738. ENSG0000(	0	5.45	-2.689	3.22E-47	ACR
AC016738. ENSG0000(	0.03	3.55	-2.143	1.80E-80	OXCT2
AC016757. ENSG0000(	0.73	39.89	-4.563	1.27E-88	CAPN11
AC016907. ENSG0000(	0.02	4.57	-2.449	1.04E-71	TRAPPC12
AC016909. ENSG0000(	0	6.41	-2.889	2.08E-53	CST8
AC016912. ENSG0000(	0.51	16.89	-3.567	3.43E-51	CCDC7
AC018766. ENSG0000(	0.28	7.78	-2.778	2.83E-72	MCF2
AC019048. ENSG0000(	0	4.73	-2.518	3.23E-51	CLIP4
AC019055. ENSG0000(	0	25.621	-4.734	6.32E-65	DOC2A
AC019172. ENSG0000(	0.03	7.35	-3.019	1.55E-90	HHIPL2
AC020956. ENSG0000(	0	5.57	-2.716	1.29E-83	ST7L

AC022007. ENSG0000(	2.37	82.458	-4.63	2.94E-78	CCDC15
AC024560. ENSG0000(	6.05	27.319	-2.006	2.73E-46	GDF9
AC024619. ENSG0000(	0	19.329	-4.345	1.47E-64	SLC1A6
AC026471. ENSG0000(	0.15	48.05	-5.415	6.61E-70	NAT6
AC034198. ENSG0000(	0	7.5	-3.087	4.19E-53	PIWIL1
AC034220. ENSG0000(	0.91	6.76	-2.022	1.45E-59	CDADC1
AC034243. ENSG0000(	0.28	5.37	-2.315	9.63E-52	LYZL6
AC053503. ENSG0000(	0.22	4.09	-2.061	6.27E-67	SSX1
AC062017. ENSG0000(	0.47	52.211	-5.178	3.47E-90	SOCS7
AC064834. ENSG0000(	0	4.62	-2.491	2.57E-46	SPATA6
AC067945. ENSG0000(	0.14	7.88	-2.962	1.38E-59	HSPA4L
AC068042. ENSG0000(	0	12.3	-3.733	3.07E-61	HOOK1
AC068538. ENSG0000(	0	7.95	-3.162	9.13E-59	ZNF821
AC069213. ENSG0000(	0.15	3.81	-2.064	2.52E-23	SPATA7
AC069363. ENSG0000(	0.29	4.83	-2.176	2.54E-39	FNDC8
AC073043. ENSG0000(	0.45	56.928	-5.32	2.86E-71	DKKL1
AC073130. ENSG0000(	0.01	9.79	-3.417	7.08E-59	ACE
AC073342. ENSG0000(	0	3.51	-2.173	9.51E-70	MS4A5
AC073464. ENSG0000(	0	33.19	-5.095	1.47E-77	TSKS
AC073628. ENSG0000(	0	9.64	-3.411	1.12E-54	SYCP1
AC073636. ENSG0000(	0	4.78	-2.531	1.64E-44	YPEL1
AC074389. ENSG0000(	0	11.72	-3.669	1.99E-77	MMP28
AC074391. ENSG0000(	0.12	4	-2.158	2.31E-46	ROPN1B
AC079305. ENSG0000(	0.08	3.68	-2.115	2.77E-58	CHD5
AC079354. ENSG0000(	0.01	7.79	-3.122	1.24E-58	TEX40
AC079354. ENSG0000(	0.17	9.37	-3.148	6.71E-55	LDHC
AC079776. ENSG0000(	0.12	14.9	-3.827	6.44E-64	TTC12
AC079779. ENSG0000(	0	16.959	-4.167	1.49E-53	DNAH17
AC079807. ENSG0000(	0.7	7.16	-2.263	2.53E-90	CTNNA3
AC079922. ENSG0000(	0.67	5.8	-2.026	7.82E-58	ZBBX
AC083884. ENSG0000(	0.17	7.01	-2.775	6.64E-96	TRIM66
AC084082. ENSG0000(	0	6.55	-2.916	1.58E-67	SHCBP1L
AC091633. ENSG0000(	0.1	6.65	-2.798	2.50E-52	CRISP2
AC092155. ENSG0000(	0	3.72	-2.239	4.94E-37	FSCN3
AC092566. ENSG0000(	0	5.61	-2.725	1.64E-38	UTRN
AC093642. ENSG0000(	0	10.95	-3.579	1.60E-53	PIGV
AC093716. ENSG0000(	0.16	4.67	-2.289	1.43E-32	C1orf105
AC093822. ENSG0000(	0	7.77	-3.133	1.51E-59	PRSS21
AC093901. ENSG0000(	0.36	24.289	-4.217	3.14E-60	SPTBN2
AC096570. ENSG0000(	0	5.43	-2.685	1.55E-50	SLC25A28
AC096649. ENSG0000(	0.15	6.65	-2.734	3.14E-63	ACSBG2
AC096669. ENSG0000(	0	7.58	-3.101	1.24E-56	ZMYND10
AC097374. ENSG0000(	0.01	3.08	-2.014	1.47E-63	ACTL7A
AC097467. ENSG0000(	0	17.56	-4.214	7.56E-68	IL13
AC097495. ENSG0000(	0	13.35	-3.843	2.60E-63	IFT88
AC098614. ENSG0000(	1.1	16.42	-3.052	2.87E-77	RAB11FIP5
AC099552. ENSG0000(	0	5.55	-2.711	2.83E-53	TCP10
AC102948. ENSG0000(	0	4.68	-2.506	3.55E-50	HSF2BP
AC102953. ENSG0000(	0	9.44	-3.384	5.07E-65	AGBL2

AC104113.	ENSG0000(	0	3.86	-2.281	3.77E-40	ZNF473
AC104451.	ENSG0000(	0	3.57	-2.192	1.41E-05	MAPRE3
AC104653.	ENSG0000(	0.3	8.4	-2.854	1.36E-57	DYRK3
AC104654.	ENSG0000(	0	9.11	-3.338	6.32E-50	MAK
AC104655.	ENSG0000(	0.3	5.28	-2.272	7.13E-56	FER
AC104777.	ENSG0000(	0	22.33	-4.544	1.42E-64	CREM
AC105339.	ENSG0000(	0.03	15.17	-3.973	1.53E-74	ELL3
AC108004.	ENSG0000(	0	6.31	-2.87	5.05E-47	SFMBT1
AC108025.	ENSG0000(	0.02	3.11	-2.011	2.58E-45	ZPBP
AC108713.	ENSG0000(	0	6.66	-2.937	4.09E-28	USP32
AC113607.	ENSG0000(	0	12.61	-3.767	3.24E-65	SMG7-AS1
AC113617.	ENSG0000(	0	6.73	-2.95	1.68E-68	BBIP1
AC114271.	ENSG0000(	1.42	13.81	-2.613	4.59E-54	RBMXL2
AC114273.	ENSG0000(	0	4.84	-2.546	1.89E-56	ODF1
AC114783.	ENSG0000(	0	126.071	-6.989	8.20E-73	SOX5
AC114812.	ENSG0000(	0.26	92.308	-6.211	4.85E-71	CHGA
AC114814.	ENSG0000(	0	3.34	-2.118	3.72E-46	HYPM
AC116035.	ENSG0000(	0.07	4.14	-2.264	8.82E-20	DRICH1
AC116614.	ENSG0000(	0.25	17.07	-3.854	2.01E-60	ANKS1B
AC125634.	ENSG0000(	0	3.92	-2.299	1.84E-56	ABCB9
AC129778.	ENSG0000(	0.02	5.83	-2.743	3.81E-53	MAPKBP1
AC132217.	ENSG0000(	7.19	57.559	-2.838	1.12E-09	CHKA
AC135048.	ENSG0000(	1.4	18.12	-2.994	9.21E-65	CCT6B
AC138035.	ENSG0000(	3.29	68.588	-4.02	4.16E-54	TEX30
AC138647.	ENSG0000(	0	10.32	-3.501	5.71E-61	PCSK2
AC140725.	ENSG0000(	0.17	8.33	-2.995	4.46E-71	ALDH1A2
AC144838.	ENSG0000(	0.5	7.69	-2.534	6.04E-30	ANKRD7
AC145123.	ENSG0000(	0	8.65	-3.271	1.69E-66	CLTCL1
AC145343.	ENSG0000(	0.38	6.35	-2.413	3.35E-65	SMPD2
AC159540.	ENSG0000(	9.29	184.106	-4.169	1.61E-63	CCNO
AC234582.	ENSG0000(	0.09	7.36	-2.939	1.18E-40	INTS6
ACACB	ENSG0000(	3.39	20.949	-2.322	1.70E-58	SPANXB1
ACADL	ENSG0000(	0.22	4.7	-2.224	3.79E-48	TEX14
ACAP1	ENSG0000(	11.9	126.386	-3.304	2.60E-44	MAGEC1
ACAT1	ENSG0000(	19.401	110.614	-2.452	1.35E-77	BAP1
ACCS	ENSG0000(	3.83	33.469	-2.835	1.51E-54	FGFR1OP
ACE	ENSG0000(	27.07	161.634	-2.535	1.37E-18	CDKL3
ACE2	ENSG0000(	0.35	34.671	-4.724	7.42E-79	SAMD4A
ACOX2	ENSG0000(	3.87	27.059	-2.526	1.68E-27	CASC1
ACPT	ENSG0000(	0.03	4.94	-2.528	4.93E-53	NR3C2
ACR	ENSG0000(	0.17	121.759	-6.713	1.53E-81	CCNA1
ACRBP	ENSG0000(	4.49	401.289	-6.195	1.34E-73	PHF1
ACRC	ENSG0000(	1.28	12.47	-2.563	3.53E-54	PRM2
ACRV1	ENSG0000(	0.24	155.19	-6.977	4.25E-74	TTBK2
ACSBG1	ENSG0000(	0.41	7.41	-2.576	9.89E-56	ARHGAP28
ACSBG2	ENSG0000(	0.35	235.094	-7.45	5.03E-76	MTL5
ACSL1	ENSG0000(	9.18	55.039	-2.461	1.52E-46	TSSK1B
ACSM1	ENSG0000(	0.12	6.76	-2.793	4.40E-98	HRASLS
ACSM3	ENSG0000(	2.63	31.6	-3.167	1.01E-60	PRM1

ACSS1	ENSG0000(	4.99	51.388	-3.129	9.50E-65	SLC6A16
ACTA2	ENSG0000(	76.351	365.872	-2.246	2.97E-15	ODF2
ACTA2-AS	ENSG0000(	0.34	11.3	-3.198	7.59E-48	STAG3
ACTG1P17	ENSG0000(	0.6	10.41	-2.834	5.26E-82	SPA17
ACTL10	ENSG0000(	0.35	77.831	-5.868	7.54E-69	C2orf42
ACTL7A	ENSG0000(	0	295.291	-8.211	3.85E-76	FKBP6
ACTL7B	ENSG0000(	0	157.465	-7.308	1.83E-79	GSG1
ACTL8	ENSG0000(	0.29	9.68	-3.05	1.74E-70	SAYSD1
ACTL9	ENSG0000(	0	148.92	-7.228	3.60E-72	FRMPD1
ACTN2	ENSG0000(	1.63	15.56	-2.655	2.00E-56	GSTA3
ACTN3	ENSG0000(	0.38	4.88	-2.091	9.15E-42	SLC2A5
ACTRT1	ENSG0000(	0.02	4.5	-2.431	2.47E-49	TEX13A
ACTRT2	ENSG0000(	0	207.777	-7.706	1.53E-74	SMCP
ACTRT3	ENSG0000(	0.79	61.32	-5.122	2.56E-68	C10orf88
ACYP1	ENSG0000(	7.67	35.099	-2.058	1.44E-66	LY6G5C
ADAD1	ENSG0000(	0.28	97.099	-6.26	1.47E-78	TTC30A
ADAD2	ENSG0000(	1.04	143.081	-6.142	2.93E-90	NUP62CL
ADAM11	ENSG0000(	0.84	10.29	-2.617	8.62E-73	C1QTNF9B
ADAM18	ENSG0000(	0.23	15.85	-3.776	6.65E-59	PHF7
ADAM2	ENSG0000(	0	30.659	-4.985	6.98E-75	RPL10L
ADAM20	ENSG0000(	0.03	5.48	-2.653	4.50E-58	LRP8
ADAM21	ENSG0000(	0.1	10.16	-3.343	1.55E-59	ZNHIT2
ADAM29	ENSG0000(	0.02	45.972	-5.525	2.57E-72	NDUFAF3
ADAM30	ENSG0000(	0	21.681	-4.503	3.08E-69	PCYT2
ADAM32	ENSG0000(	1.13	47.751	-4.517	2.36E-62	SPAG1
ADAM33	ENSG0000(	3.21	48.1	-3.544	2.49E-55	SPEF1
ADAM3A	ENSG0000(	0	18.02	-4.249	1.07E-38	CRAT
ADAM5	ENSG0000(	0	24.87	-4.693	1.23E-61	INHA
ADAM6	ENSG0000(	0	16.7	-4.146	1.54E-63	CUL3
ADAMTS1:	ENSG0000(	2.35	22.47	-2.809	1.07E-68	RNF17
ADAMTS1	ENSG0000(	0.99	9.15	-2.351	1.49E-51	DUSP13
ADAMTS7I	ENSG0000(	0.06	5.01	-2.503	3.12E-57	CLGN
ADARB2	ENSG0000(	0.17	8.01	-2.945	3.35E-90	OAZ3
ADCY10	ENSG0000(	0.24	23.28	-4.291	1.69E-70	TNP1
ADCY10P1	ENSG0000(	0.69	16.16	-3.344	7.02E-81	LIN7A
ADCY2	ENSG0000(	0.27	6.52	-2.566	8.91E-48	YBX2
ADCY5	ENSG0000(	1.29	13.23	-2.636	6.67E-32	DDX4
ADD3-AS1	ENSG0000(	0.58	92.078	-5.88	8.49E-86	ULK2
ADGB	ENSG0000(	0.23	30.181	-4.664	8.46E-64	ZNF165
ADGRG1	ENSG0000(	5	60.85	-3.366	1.08E-42	DNAI1
ADGRL3-A	ENSG0000(	0	4.78	-2.531	3.45E-40	HMGN5
ADHFE1	ENSG0000(	1.96	21.769	-2.943	2.33E-71	CEP350
ADIG	ENSG0000(	0	75.892	-6.265	2.93E-71	VCX2
ADIRF	ENSG0000(	3.87	27.49	-2.548	3.11E-18	BAG1
ADIRF-AS1	ENSG0000(	0.71	54.89	-5.031	6.50E-103	IGLL1
ADNP-AS1	ENSG0000(	0.88	29.73	-4.031	1.70E-83	TSNAXIP1
ADORA1	ENSG0000(	1.62	27.81	-3.459	3.60E-66	IQCE
ADORA2A-	ENSG0000(	0.21	20.22	-4.132	3.05E-83	DXO
ADORA3	ENSG0000(	1.81	66.721	-4.591	8.67E-63	ADAM3A

ADPGK-AS	ENSG0000(	0.15	4.28	-2.199	6.76E-63	DNAH7
ADRA2A	ENSG0000(	0.76	6.5	-2.091	2.24E-38	SPAG6
ADSSL1	ENSG0000(	3.87	83.62	-4.119	9.88E-79	USP29
AF038458.!	ENSG0000(	0.07	4.16	-2.27	1.63E-52	CDKL2
AF067845.:	ENSG0000(	0	4.5	-2.459	9.75E-71	VRK3
AF131216.:	ENSG0000(	0.59	45.129	-4.859	2.91E-66	SH3TC2
AF146191.4	ENSG0000(	0.18	6.7	-2.706	1.67E-74	DFNB31
AF186192.(	ENSG0000(	0.39	7.15	-2.552	2.57E-60	RALGPS1
AFF3	ENSG0000(	0.86	6.88	-2.083	1.66E-38	PHKG2
AGAP10P	ENSG0000(	0.38	12.09	-3.246	1.68E-78	NRIP3
AGAP11	ENSG0000(	0.18	5.65	-2.495	2.91E-90	CYB5R2
AGAP5	ENSG0000(	3.84	19.95	-2.114	1.99E-60	BRDT
AGAP7P	ENSG0000(	0.72	9.47	-2.606	1.80E-48	TOLLIP
AGAP9	ENSG0000(	4.57	33.321	-2.623	3.39E-52	IFT140
AGBL1-AS:	ENSG0000(	0	12.75	-3.781	8.53E-59	RSPH6A
AGBL2	ENSG0000(	0.66	53.241	-5.03	1.43E-80	TBL2
AGBL3	ENSG0000(	1.26	11.65	-2.485	1.73E-70	KIAA0895
AGBL5	ENSG0000(	19.24	289.014	-3.841	1.47E-59	H2AFJ
AGPAT3	ENSG0000(	9.19	46.62	-2.224	9.35E-32	VAMP2
AGPAT9	ENSG0000(	0.55	9.13	-2.708	6.41E-52	GID4
AGT	ENSG0000(	0.27	5.08	-2.259	1.52E-06	ADORA1
AHI1	ENSG0000(	6.16	45.339	-2.694	1.84E-67	B9D1
AHRR	ENSG0000(	0.33	63.619	-5.602	1.99E-85	CCDC181
AHSA2	ENSG0000(	11.42	89.647	-2.868	3.35E-64	SPINK2
AHSP	ENSG0000(	0.37	5.04	-2.14	3.13E-37	TLE4
AJ011932.1	ENSG0000(	0.5	9.56	-2.816	1.74E-47	CNNM1
AK1	ENSG0000(	20.41	179.731	-3.077	3.82E-64	C9orf116
AK7	ENSG0000(	1	7.9	-2.154	1.70E-44	CNTRL
AK8	ENSG0000(	1.26	33.321	-3.925	7.87E-62	CTNNA2
AK9	ENSG0000(	0.78	20.21	-3.575	4.30E-89	SLC30A4
AKAP14	ENSG0000(	0	100.837	-6.67	1.73E-67	SRPK2
AKAP3	ENSG0000(	4.77	103.113	-4.173	4.08E-38	CDK20
AKAP4	ENSG0000(	0.01	257.28	-7.998	2.07E-77	FAAH
AKAP6	ENSG0000(	0.42	6.63	-2.426	2.03E-60	PIP5K1C
AKNAD1	ENSG0000(	0.24	11.35	-3.316	6.74E-55	TP53TG5
AKR1C1	ENSG0000(	1.2	19.569	-3.225	7.52E-37	BRAP
AKR1C2	ENSG0000(	0.63	12.99	-3.101	1.55E-31	CCNH
AKR1D1	ENSG0000(	0.01	4.31	-2.394	1.09E-34	NME5
AKR1E2	ENSG0000(	1.41	48.751	-4.368	4.52E-88	ATF7IP2
AL031601.!	ENSG0000(	0	3.26	-2.091	5.30E-54	CABYR
AL050303.:	ENSG0000(	0	5.5	-2.7	6.21E-55	RUFY3
AL121578.:	ENSG0000(	0	5.15	-2.621	1.05E-48	ZDHHC14
AL121656.!	ENSG0000(	0	5.47	-2.694	1.07E-49	DPEP3
AL157871.2	ENSG0000(	0	4.57	-2.478	1.49E-31	ZNF415
AL157902.:	ENSG0000(	0	11.06	-3.592	2.35E-66	SLC30A3
AL353997.:	ENSG0000(	0.07	25.871	-4.65	2.39E-81	NR0B1
AL442127.:	ENSG0000(	0	17.14	-4.181	4.80E-10	FAM53C
AL450226.2	ENSG0000(	0	21.381	-4.484	2.06E-62	PLSCR2
AL589743.:	ENSG0000(	0.62	5.61	-2.029	2.24E-33	CSNK2A2

AL591893.1	ENSG0000(	0.03	4.74	-2.478	3.36E-58	KLHL11
ALB	ENSG0000(	0.3	4.79	-2.155	4.95E-14	DZIP1
ALDH1A1	ENSG0000(	14.25	132.293	-3.128	5.78E-44	PIK3R3
ALDH1A2	ENSG0000(	0.65	42.7	-4.727	4.62E-60	SIK3
ALDH1L1	ENSG0000(	0.53	22.95	-3.968	8.00E-78	PAOX
ALKBH3-A!	ENSG0000(	0.21	14.37	-3.667	9.50E-66	KCTD13
ALKBH7	ENSG0000(	90.722	745.156	-3.024	1.47E-47	HAGH
ALLC	ENSG0000(	0.05	30.61	-4.912	2.10E-90	TTLL1
ALOX12-A!	ENSG0000(	2.14	12.92	-2.148	1.55E-83	PGAM2
ALOX12P2	ENSG0000(	1.05	11.14	-2.566	4.14E-44	GRAMD1B
ALS2CR11	ENSG0000(	2.2	29.84	-3.269	8.14E-45	FHL5
ALS2CR12	ENSG0000(	0.19	18.63	-4.044	3.81E-65	LRRC49
AMH	ENSG0000(	3.47	24.55	-2.515	3.70E-38	FUZ
AMHR2	ENSG0000(	1.17	17.289	-3.075	1.96E-47	SHARPIN
AMN1	ENSG0000(	8	47.261	-2.423	6.11E-49	PROX1
AMOTL1	ENSG0000(	1.51	13.39	-2.519	3.46E-23	CCT8L2
AMY1C	ENSG0000(	0	6.84	-2.971	1.13E-38	DYNC2H1
AMZ2P1	ENSG0000(	6.02	49.931	-2.859	3.34E-89	DNALI1
ANG	ENSG0000(	2.52	13.99	-2.09	1.37E-25	ASB9
ANGEL1	ENSG0000(	14.21	66.911	-2.159	1.04E-36	TRIM13
ANKAR	ENSG0000(	0.79	11.04	-2.75	6.36E-89	EIF4G3
ANKEF1	ENSG0000(	2.27	25.38	-3.012	6.46E-50	NUP98
ANKFN1	ENSG0000(	0.05	4.06	-2.269	6.38E-56	OXR1
ANKMY1	ENSG0000(	3.91	95.882	-4.302	1.31E-81	CDK19
ANKRD12	ENSG0000(	4.15	27.04	-2.445	4.53E-61	FAM216A
ANKRD13B	ENSG0000(	2.65	36.719	-3.369	7.98E-73	OR7E156P
ANKRD18A	ENSG0000(	0.47	21.23	-3.919	4.71E-76	SKAP2
ANKRD18B	ENSG0000(	0.55	8.84	-2.666	8.84E-62	PGR
ANKRD18C	ENSG0000(	0.15	3.7	-2.031	5.05E-61	CCDC176
ANKRD18D	ENSG0000(	0.06	3.75	-2.164	2.94E-66	CTSF
ANKRD19P	ENSG0000(	2.08	85.912	-4.819	2.99E-38	ARL4A
ANKRD20A	ENSG0000(	0.3	10.13	-3.098	7.77E-85	SPRR2C
ANKRD20A	ENSG0000(	0.02	3.72	-2.21	2.81E-68	PIWIL2
ANKRD20A	ENSG0000(	0.09	18.77	-4.181	1.42E-81	WDR60
ANKRD20A	ENSG0000(	0.08	4.32	-2.3	3.19E-72	PDXK
ANKRD20A	ENSG0000(	0.16	3.79	-2.046	1.51E-58	DOCK3
ANKRD20A	ENSG0000(	0.76	33.369	-4.287	2.21E-64	ABCA5
ANKRD24	ENSG0000(	1.87	17.66	-2.701	3.73E-70	TFDP3
ANKRD26P	ENSG0000(	2.69	99.408	-4.766	3.05E-43	GDF10
ANKRD30B	ENSG0000(	0.01	18.11	-4.242	1.87E-68	PPEF1
ANKRD30B	ENSG0000(	0.05	21.561	-4.425	1.91E-88	STAG3L3
ANKRD30B	ENSG0000(	0	9.27	-3.36	3.42E-72	NPIPB15
ANKRD31	ENSG0000(	0.12	6.02	-2.648	3.79E-86	ETNK2
ANKRD36B	ENSG0000(	2.07	23.98	-3.024	1.66E-54	MARK4
ANKRD36C	ENSG0000(	1.03	17.03	-3.151	9.41E-103	AGBL5
ANKRD42	ENSG0000(	2.9	47.399	-3.633	2.24E-89	SLC2A8
ANKRD45	ENSG0000(	0.72	14.79	-3.198	5.96E-66	ELOVL2
ANKRD50	ENSG0000(	1.62	11.27	-2.227	7.61E-41	TBPL1
ANKRD53	ENSG0000(	0.59	40.369	-4.701	5.74E-70	SSX2IP

ANKRD60	ENSG0000(	0	4.04	-2.333	2.00E-52	TEKT2
ANKRD61	ENSG0000(	0.19	6.77	-2.707	1.35E-56	SKAP1
ANKRD65	ENSG0000(	1.51	9.29	-2.035	6.27E-31	TMBIM4
ANKRD7	ENSG0000(	1.54	663.107	-8.03	8.53E-83	OPLAH
ANKRD9	ENSG0000(	5.31	49.559	-3.002	7.73E-59	TCFL5
ANKS1B	ENSG0000(	1.03	19.849	-3.36	1.34E-53	CTAG2
ANO1	ENSG0000(	2.48	17.8	-2.434	1.46E-30	ATP8A2
ANO1-AS2	ENSG0000(	0	7.39	-3.069	2.40E-63	PRPH
ANTXRL	ENSG0000(	0.01	26	-4.741	7.76E-115	CCSER2
ANXA13	ENSG0000(	0.1	4.87	-2.416	1.48E-24	PAK6
AP000281.:	ENSG0000(	0	4.01	-2.325	7.76E-44	SLC35G2
AP000289.(	ENSG0000(	0	4.21	-2.381	1.22E-48	CHODL
AP000345.:	ENSG0000(	0.08	12.84	-3.68	9.22E-70	AKAP3
AP000432.:	ENSG0000(	0.23	4.04	-2.035	1.33E-52	KHDRBS3
AP000442.:	ENSG0000(	0.64	10.5	-2.81	1.37E-104	MYLK3
AP001048.4	ENSG0000(	0	9.55	-3.399	3.20E-57	HGFAC
AP001062.8	ENSG0000(	0.68	6.57	-2.172	5.79E-53	POLI
AP001425.:	ENSG0000(	0.03	3.82	-2.226	7.08E-54	CRYBG3
AP001468.!	ENSG0000(	0	5.23	-2.639	1.87E-47	TPGS2
AP001476.:	ENSG0000(	0.07	4.05	-2.239	5.93E-73	MRGBP
AP001625.4	ENSG0000(	0	11.25	-3.615	6.88E-65	FAM46C
AP001630.!	ENSG0000(	0	5	-2.585	8.78E-61	SNAP91
AP003025.:	ENSG0000(	0	13.54	-3.862	1.78E-63	MBNL3
AP003774.!	ENSG0000(	0	7.05	-3.009	8.60E-58	INPP4B
AP004782.:	ENSG0000(	0.05	3.25	-2.017	9.76E-76	APPBP2
AP005530.:	ENSG0000(	0.06	13.18	-3.742	3.92E-62	IPO5
AP006216.!	ENSG0000(	0	4.29	-2.403	1.71E-51	GRB14
APBB3	ENSG0000(	8.62	94	-3.304	2.62E-115	TPTE
APH1B	ENSG0000(	7.69	295.947	-5.095	1.31E-65	SPAG4
APITD1-C(	ENSG0000(	1.76	22	-3.059	8.88E-67	ELP5
APLF	ENSG0000(	0.94	7.04	-2.051	7.25E-70	TPP2
APOA1	ENSG0000(	8.32	459.556	-5.627	2.30E-24	IQCG
APOBEC4	ENSG0000(	0	8.47	-3.243	6.46E-50	RNF141
APOD	ENSG0000(	18.42	91.74	-2.256	4.64E-15	MAP7
APOM	ENSG0000(	2.02	20.73	-2.847	2.86E-18	INPP1
APOPT1	ENSG0000(	34	148.384	-2.094	3.70E-39	GPX4
APPBP2	ENSG0000(	5.5	33.261	-2.398	1.83E-53	TESC
AQP11	ENSG0000(	1.5	11.42	-2.313	4.78E-77	DPY19L2P:
AQP5	ENSG0000(	2.72	293.496	-6.307	5.22E-66	PVRL3
AQP7	ENSG0000(	0.19	48.999	-5.393	2.04E-111	POMT1
AREG	ENSG0000(	0.16	11.86	-3.471	1.28E-74	STAT4
ARHGAP20	ENSG0000(	0.2	6.8	-2.7	4.42E-95	WT1
ARHGAP22	ENSG0000(	0	8.14	-3.192	2.54E-61	TOMM34
ARHGAP26	ENSG0000(	3.61	23.381	-2.403	3.41E-61	KCNH2
ARHGAP26	ENSG0000(	0	10.05	-3.466	1.00E-51	CCDC53
ARHGAP26	ENSG0000(	0	5.5	-2.7	2.89E-52	FBXL2
ARHGAP28	ENSG0000(	6.48	52.991	-2.852	6.35E-27	ACE2
ARHGAP35	ENSG0000(	8.92	134	-3.766	2.12E-63	CLEC16A
ARHGAP39	ENSG0000(	5.36	28.391	-2.208	3.03E-51	GUCA1A

ARHGAP9	ENSG0000	13.91	66.911	-2.187	1.22E-29	CCNJL
ARHGEF33	ENSG0000	1.48	13.58	-2.556	2.13E-51	CETN3
ARHGEF37	ENSG0000	0.25	5.02	-2.268	8.78E-59	IFT81
ARHGEF7-/	ENSG0000	0	4.27	-2.398	4.80E-56	PDE1A
ARHGEF7-I	ENSG0000	0	3.13	-2.046	6.46E-62	SCRG1
ARL13A	ENSG0000	0.06	13.99	-3.822	2.34E-55	ENOX1
ARL2BPP3	ENSG0000	0	13.25	-3.833	5.01E-64	PDZD2
ARL4A	ENSG0000	18.77	246.611	-3.647	3.17E-56	CALB2
ARL6	ENSG0000	1.95	11.28	-2.058	4.37E-63	WASF1
ARL9	ENSG0000	0.34	10	-3.037	6.82E-61	MLF1
ARMC12	ENSG0000	0.43	86.318	-5.932	2.69E-90	KLHDC10
ARMC2	ENSG0000	2.79	27.14	-2.892	1.25E-72	LPIN1
ARMC3	ENSG0000	0.33	33.819	-4.71	8.13E-62	GLUD2
ARMC4	ENSG0000	0.25	30.73	-4.666	2.23E-67	TEX11
ARMS2	ENSG0000	0.03	3.47	-2.118	4.63E-47	CUL9
ARNT2	ENSG0000	0.62	12.21	-3.028	9.23E-42	MAPK13
ARNTL	ENSG0000	3.25	19.73	-2.286	8.80E-93	SCCPDH
ARRDC1-A	ENSG0000	6.03	30.399	-2.159	1.01E-82	MAMLD1
ARRDC3-A	ENSG0000	0.58	9.11	-2.678	5.75E-88	SLC12A2
ARRDC5	ENSG0000	0.14	21.43	-4.298	3.31E-63	PRND
ARSA	ENSG0000	13.05	108.608	-2.964	1.09E-57	SCML1
ART3	ENSG0000	0.29	95.299	-6.222	9.05E-99	FOXG1
ART5	ENSG0000	4.98	31.65	-2.449	4.22E-30	CDKN3
ASAP2	ENSG0000	5.53	32.271	-2.349	4.11E-26	EFHC1
ASB17	ENSG0000	0	41.521	-5.41	7.97E-67	TCEAL2
ASB5	ENSG0000	0	7.63	-3.109	2.12E-41	PAQR3
ASB9	ENSG0000	1.39	32.28	-3.8	7.89E-65	CITED1
ASGR1	ENSG0000	2.04	38.109	-3.685	2.38E-35	NAALAD2
ASIC3	ENSG0000	11.05	54.78	-2.211	1.02E-30	ST6GALNA
ASXL3	ENSG0000	0.09	8.13	-3.066	2.41E-58	FOXD1
ASZ1	ENSG0000	0.09	13.22	-3.706	1.88E-94	SPHK2
ATAD1	ENSG0000	13.28	92.937	-2.718	2.40E-53	LRRC16A
ATE1-AS1	ENSG0000	0.28	4.94	-2.214	2.06E-50	HSPA2
ATF7IP2	ENSG0000	7.82	92.289	-3.403	4.89E-86	PMS1
ATG4D	ENSG0000	27.93	125.07	-2.124	1.73E-49	GOLGA8N
ATG9B	ENSG0000	8.23	44.47	-2.3	2.73E-15	MAGEA1
ATL1	ENSG0000	0.92	7.57	-2.158	1.19E-38	WDR62
ATP10A	ENSG0000	1.62	9.67	-2.026	2.86E-57	CYB5R4
ATP1A2	ENSG0000	0.28	7.47	-2.726	1.71E-17	BCL2L14
ATP1A3	ENSG0000	5.12	53.641	-3.158	1.04E-35	CYP26B1
ATP1A4	ENSG0000	0.44	27.77	-4.32	2.65E-61	USP25
ATP5G1P1	ENSG0000	0	7.43	-3.076	1.43E-71	LRRC6
ATP6AP1L	ENSG0000	2.66	16.74	-2.277	1.76E-64	CCDC92
ATP6V1E2	ENSG0000	4.27	37.83	-2.881	9.43E-49	ME3
ATP8A2	ENSG0000	0.87	10.33	-2.599	8.45E-53	TTYH1
ATP8B3	ENSG0000	1.41	51.189	-4.437	2.08E-69	SLC6A1
ATP8B5P	ENSG0000	0.2	13.99	-3.643	1.46E-77	NDRG4
ATRNL1	ENSG0000	3.36	17.81	-2.109	1.21E-36	AZGP1
ATXN3L	ENSG0000	0	4.34	-2.417	2.89E-56	PENK

AURKC	ENSG0000(	1.9	73.848	-4.69	2.43E-77	TCEA2
AXDND1	ENSG0000(	0.56	41.04	-4.752	3.76E-76	IFT57
AY269186.:	ENSG0000(	0	3.87	-2.284	9.33E-53	CLK4
AY269186.:	ENSG0000(	0	3.94	-2.305	1.47E-46	GPR18
AZGP1	ENSG0000(	0.14	43.822	-5.297	1.60E-73	BAZ2B
AZIN1-AS1	ENSG0000(	1.64	57.059	-4.459	4.40E-75	INHBB
AZIN2	ENSG0000(	2.7	73.17	-4.325	1.41E-84	RNF139
B3GALT4	ENSG0000(	3.21	36.38	-3.15	1.36E-60	MAGEA11
B4GALT1-/	ENSG0000(	0.71	22.59	-3.786	2.13E-67	BAZ1A
B4GALT4-/	ENSG0000(	0	5.49	-2.698	1.64E-47	GSTM3
B9D1	ENSG0000(	11.95	94.398	-2.881	5.35E-47	CAND2
BAALC-AS:	ENSG0000(	1.16	10.88	-2.459	1.08E-46	MPC2
BAG1	ENSG0000(	27.92	178.923	-2.637	5.21E-61	GLRX2
BAG5	ENSG0000(	16.61	137.091	-2.971	2.01E-41	MYOM2
BAHCC1	ENSG0000(	1.6	11.14	-2.223	6.68E-28	SPAG16
BAIAP3	ENSG0000(	4.6	43.11	-2.978	1.55E-43	GULP1
BANF2	ENSG0000(	0	140.808	-7.148	4.97E-71	DMRT1
BAP1	ENSG0000(	41.06	196.596	-2.232	7.01E-66	ZCCHC2
BAZ1A	ENSG0000(	7.31	40.61	-2.324	2.42E-61	KLK11
BAZ2B	ENSG0000(	3.53	19.66	-2.189	2.72E-40	ISYNA1
BBIP1	ENSG0000(	11.09	123.22	-3.361	3.04E-77	DAZL
BBS1	ENSG0000(	5.79	28.729	-2.13	5.02E-46	RPL39L
BBX	ENSG0000(	8.78	55.741	-2.536	5.07E-58	CYP17A1
BCL2L14	ENSG0000(	1.58	44.409	-4.138	2.81E-41	SAR1B
BCL2L2-PA	ENSG0000(	0.74	13.72	-3.081	2.14E-55	PPARGC1A
BCL6	ENSG0000(	3.38	26.67	-2.659	1.94E-44	ZNF516
BCO2	ENSG0000(	0.63	11.49	-2.938	5.02E-88	EPB41L3
BCRP2	ENSG0000(	1.51	26.21	-3.438	1.44E-76	SEC61A2
BCRP3	ENSG0000(	7.21	33.889	-2.087	1.89E-13	TMX4
BEAN1	ENSG0000(	0.61	8.86	-2.615	8.83E-63	NXF3
BEND2	ENSG0000(	0.01	3.6	-2.187	2.65E-89	ACAT1
BEND7	ENSG0000(	4.44	35.36	-2.741	1.59E-63	SPATA20
BEST1	ENSG0000(	0.9	12.44	-2.822	6.22E-87	MICAL2
BEST3	ENSG0000(	0.04	3.82	-2.212	1.64E-57	HSD17B3
BEX1	ENSG0000(	14	112.813	-2.924	2.06E-24	DAAM2
BEX5	ENSG0000(	2.44	15.65	-2.275	1.73E-29	SH3GL2
BHMG1	ENSG0000(	0.36	9.45	-2.942	6.66E-64	KLK5
BIRC8	ENSG0000(	0	9.78	-3.43	5.39E-65	IL13RA2
BMP7-AS1	ENSG0000(	0	3.72	-2.239	4.42E-43	P4HTM
BMPR1B-A	ENSG0000(	0	12.94	-3.801	2.24E-63	STX8
BNIPL	ENSG0000(	0.38	7.07	-2.548	7.49E-41	OPTN
BOD1L2	ENSG0000(	0.06	341.631	-8.336	8.01E-84	DACH1
BOLL	ENSG0000(	0.07	78.188	-6.21	2.74E-87	LZTFL1
BPI	ENSG0000(	0.46	32.981	-4.541	1.42E-71	LRRN3
BPIFA3	ENSG0000(	0	133.564	-7.072	1.42E-73	TKTL1
BPIFB1	ENSG0000(	0.03	6.63	-2.889	1.61E-28	PRKCZ
BRAP	ENSG0000(	19.54	85.603	-2.076	1.38E-37	C16orf45
BRDT	ENSG0000(	9.78	82.229	-2.949	1.84E-28	REEP1
BRICD5	ENSG0000(	5.4	36.859	-2.565	7.73E-78	GATM

BRINP2	ENSG0000(	0.15	3.66	-2.019	2.63E-40	SERPINA5
BRINP3	ENSG0000(	0.04	5.5	-2.644	1.03E-37	SLC27A6
BRSK2	ENSG0000(	1.62	21.739	-3.118	6.16E-60	PPFIBP2
BRWD1-AS	ENSG0000(	0.03	3.19	-2.024	2.06E-54	GSTA1
BRWD1-AS	ENSG0000(	0.61	50.741	-5.006	4.53E-77	BBX
BSCL2	ENSG0000(	36.911	359.213	-3.248	2.99E-59	CENPU
BSN-AS2	ENSG0000(	0.16	6.09	-2.612	3.80E-65	ARNT2
BSNDP2	ENSG0000(	0	3.04	-2.014	1.17E-33	ELAVL4
BTBD16	ENSG0000(	0.02	10.33	-3.474	5.58E-60	PRKCDBP
BTBD6	ENSG0000(	20.45	101.75	-2.26	1.42E-56	JAM2
BTBD9-AS	ENSG0000(	0	6.57	-2.92	1.12E-64	OSR2
BTG4	ENSG0000(	0.09	23.821	-4.509	2.83E-82	MAGEA4
BTNL8	ENSG0000(	0.12	7.92	-2.994	1.57E-67	KRT23
BTNL9	ENSG0000(	2.51	13.27	-2.023	2.47E-36	ZBTB38
BVES	ENSG0000(	1.43	9.13	-2.06	3.89E-44	ASAP2
BVES-AS1	ENSG0000(	0.27	12.79	-3.441	3.99E-70	TUBA4A
BX255923.:	ENSG0000(	0.22	101.983	-6.399	1.84E-68	ALDH1A1
BZW1P2	ENSG0000(	10.21	43.919	-2.002	1.32E-20	CEP70
C10orf107	ENSG0000(	0.07	9.51	-3.296	2.93E-57	TNFAIP6
C10orf120	ENSG0000(	0	37.021	-5.249	2.24E-66	PTGDS
C10orf53	ENSG0000(	0	50.32	-5.681	1.48E-74	INSL3
C10orf55	ENSG0000(	0.15	15.93	-3.88	2.07E-55	CNN1
C10orf62	ENSG0000(	0	358.367	-8.489	1.27E-77	NEFM
C10orf67	ENSG0000(	0.16	9.4	-3.164	4.61E-72	CPE
C10orf82	ENSG0000(	2.5	124.862	-5.168	1.16E-58	SMARCA1
C10orf88	ENSG0000(	4.45	25.621	-2.288	1.24E-58	PLK2
C10orf90	ENSG0000(	0.04	9.2	-3.294	1.20E-54	CLU
C11orf16	ENSG0000(	0.14	4.02	-2.139	2.41E-41	PLA2G16
C11orf42	ENSG0000(	0.04	32.159	-4.995	6.37E-63	TIPARP
C11orf53	ENSG0000(	0.02	6.38	-2.855	9.82E-58	TSPYL5
C11orf63	ENSG0000(	0.79	25.72	-3.9	9.64E-79	SOX9
C11orf65	ENSG0000(	0.91	55.811	-4.895	1.71E-71	KIF5C
C11orf70	ENSG0000(	1.1	44.12	-4.425	1.73E-82	MAGEA12
C11orf71	ENSG0000(	3.4	148.219	-5.084	5.49E-85	HOPX
C11orf85	ENSG0000(	0.57	33.18	-4.444	3.21E-76	ZBTB20
C11orf88	ENSG0000(	0.05	35.49	-5.119	3.91E-60	HMGCS2
C11orf91	ENSG0000(	0.05	13.05	-3.742	1.21E-79	CKM
C11orf94	ENSG0000(	0.1	74.258	-6.096	7.80E-75	STAR
C11orf96	ENSG0000(	7.29	77.219	-3.238	2.45E-48	ABCA8
C11orf97	ENSG0000(	0	25.05	-4.703	3.12E-72	FHL1
C12orf42	ENSG0000(	0.16	36.559	-5.017	1.10E-66	SPOCK1
C12orf50	ENSG0000(	0.03	61.571	-5.925	3.57E-75	LIPG
C12orf54	ENSG0000(	0.11	101.398	-6.527	3.46E-69	MAGEA6
C12orf60	ENSG0000(	3.35	19.311	-2.223	4.35E-33	CADM1
C12orf71	ENSG0000(	0.18	4.03	-2.092	2.09E-41	MOK
C12orf80	ENSG0000(	0	8.81	-3.294	2.70E-59	PFKP
C14orf39	ENSG0000(	0.64	6.94	-2.275	1.20E-51	CYP11A1
C14orf79	ENSG0000(	5.66	44.01	-2.757	5.68E-63	CST3
C15orf26	ENSG0000(	0.25	6.58	-2.6	3.41E-58	DDAH1

C15orf39	ENSG00000	21.491	96.268	-2.113	4.30E-46	SERPINF1
C15orf43	ENSG00000	0.08	18.45	-4.171	1.55E-66	APOA1
C15orf48	ENSG00000	10.24	129.948	-3.542	6.17E-33	BEX1
C15orf52	ENSG00000	0.71	13.18	-3.052	7.17E-64	
C15orf59-/	ENSG00000	0.64	15.06	-3.292	1.43E-40	
C15orf61	ENSG00000	3.63	28.34	-2.664	1.70E-44	
C15orf65	ENSG00000	2.33	12.85	-2.056	2.79E-26	
C16orf45	ENSG00000	11.25	68.869	-2.512	2.49E-48	
C16orf46	ENSG00000	0.99	12.49	-2.761	6.53E-62	
C16orf71	ENSG00000	0.69	97.099	-5.859	2.38E-81	
C16orf78	ENSG00000	0	55.299	-5.815	8.78E-59	
C16orf82	ENSG00000	0.01	356.954	-8.469	1.71E-76	
C16orf86	ENSG00000	1.55	64.61	-4.685	1.44E-100	
C16orf90	ENSG00000	0.04	62.318	-5.928	6.20E-81	
C16orf92	ENSG00000	0	26.75	-4.794	3.66E-66	
C16orf93	ENSG00000	3.51	66.879	-3.912	1.43E-78	
C16orf95	ENSG00000	2.57	170	-5.582	4.85E-77	
C16orf96	ENSG00000	0.31	13.83	-3.501	3.53E-66	
C16orf97	ENSG00000	0	11.7	-3.667	2.83E-60	
C17orf104	ENSG00000	0.87	40.229	-4.463	1.84E-76	
C17orf105	ENSG00000	0.03	61.252	-5.917	7.70E-71	
C17orf112	ENSG00000	0	11.59	-3.654	4.47E-59	
C17orf47	ENSG00000	0.02	7.3	-3.025	2.60E-59	
C17orf50	ENSG00000	0.08	137.414	-7.002	2.95E-77	
C17orf64	ENSG00000	0.27	169.236	-7.067	3.13E-84	
C17orf74	ENSG00000	0.19	167.369	-7.145	3.13E-69	
C17orf97	ENSG00000	2.58	103.658	-4.87	1.34E-65	
C17orf98	ENSG00000	0.06	24.62	-4.595	4.13E-66	
C18orf63	ENSG00000	0	4.67	-2.503	5.81E-79	
C19orf18	ENSG00000	1.06	15.02	-2.959	4.21E-58	
C19orf38	ENSG00000	2.54	14.71	-2.15	4.52E-50	
C19orf44	ENSG00000	4.5	23.18	-2.136	3.24E-61	
C19orf45	ENSG00000	0.65	37.01	-4.526	2.15E-60	
C19orf47	ENSG00000	12.4	54.198	-2.042	5.03E-53	
C19orf57	ENSG00000	3.18	28.259	-2.807	1.46E-62	
C19orf67	ENSG00000	0.27	9.55	-3.054	5.25E-55	
C19orf71	ENSG00000	0.97	68.251	-5.136	9.73E-77	
C19orf81	ENSG00000	1.76	10.61	-2.073	4.50E-39	
C19orf84	ENSG00000	3.74	29.449	-2.683	6.18E-42	
C1GALT1C:	ENSG00000	0.6	7.35	-2.384	2.66E-69	
C1orf100	ENSG00000	0.15	72.66	-6.001	7.20E-66	
C1orf101	ENSG00000	0.4	19.231	-3.853	6.44E-77	
C1orf105	ENSG00000	0.27	34.63	-4.81	3.19E-64	
C1orf111	ENSG00000	0	72.998	-6.209	4.08E-70	
C1orf141	ENSG00000	0.02	11.07	-3.565	1.08E-99	
C1orf146	ENSG00000	0.2	5.36	-2.406	1.41E-59	
C1orf158	ENSG00000	0.01	61.349	-5.948	7.92E-69	
C1orf159	ENSG00000	8.34	59.128	-2.687	2.21E-76	
C1orf167	ENSG00000	0.55	52.921	-5.121	6.77E-81	

C1orf168	ENSG0000(	0.04	3.21	-2.017	2.32E-21
C1orf177	ENSG0000(	0.03	5.68	-2.697	8.64E-54
C1orf185	ENSG0000(	0	28.699	-4.892	4.18E-62
C1orf189	ENSG0000(	0	26.661	-4.79	9.88E-64
C1orf194	ENSG0000(	0.2	393.575	-8.361	5.49E-72
C1orf228	ENSG0000(	3.42	36.249	-3.075	1.86E-42
C1orf234	ENSG0000(	0.1	101.581	-6.543	8.12E-72
C1orf56	ENSG0000(	5.06	134.708	-4.485	1.23E-74
C1orf61	ENSG0000(	0.86	48.311	-4.729	7.92E-49
C1orf87	ENSG0000(	0.01	20.15	-4.388	1.05E-62
C1orf94	ENSG0000(	0.11	24.19	-4.504	5.28E-71
C1QTNF3	ENSG0000(	0.75	33.861	-4.316	1.77E-53
C1QTNF3-	ENSG0000(	0.06	3.71	-2.152	2.71E-85
C1QTNF9B	ENSG0000(	0.39	6.03	-2.338	2.32E-51
C1QTNF9B	ENSG0000(	1.57	44.191	-4.136	3.98E-61
C20orf141	ENSG0000(	0.08	173.946	-7.34	1.22E-73
C20orf144	ENSG0000(	0.09	234.866	-7.757	3.00E-78
C20orf173	ENSG0000(	0	44.329	-5.502	2.37E-69
C20orf187	ENSG0000(	0	4.06	-2.339	1.78E-41
C20orf195	ENSG0000(	0.39	144.918	-6.714	8.59E-73
C20orf62	ENSG0000(	0	4.93	-2.568	2.57E-49
C20orf78	ENSG0000(	0	7.36	-3.064	5.35E-61
C20orf85	ENSG0000(	0.12	102.778	-6.534	1.54E-66
C20orf96	ENSG0000(	3.16	39.01	-3.266	1.30E-60
C21orf62-/	ENSG0000(	0.32	7.87	-2.748	8.32E-66
C22orf15	ENSG0000(	0.16	7.22	-2.825	1.36E-55
C22orf23	ENSG0000(	0.99	117.677	-5.898	3.09E-71
C22orf31	ENSG0000(	0.03	10.06	-3.425	2.80E-78
C22orf42	ENSG0000(	0.21	21.099	-4.191	4.35E-60
C2orf16	ENSG0000(	0.25	24.55	-4.353	2.41E-69
C2orf27A	ENSG0000(	1.15	9.82	-2.331	4.42E-88
C2orf27AP:	ENSG0000(	0.28	7.56	-2.741	1.24E-68
C2orf40	ENSG0000(	0.44	64.771	-5.513	2.39E-56
C2orf42	ENSG0000(	8.09	55.341	-2.632	2.72E-59
C2orf57	ENSG0000(	0	195.591	-7.619	1.12E-72
C2orf61	ENSG0000(	0.05	17.75	-4.158	1.93E-62
C2orf70	ENSG0000(	0.06	39.681	-5.262	1.33E-69
C2orf73	ENSG0000(	0.06	57.098	-5.776	1.46E-71
C2orf78	ENSG0000(	0	14.58	-3.962	1.54E-84
C2orf81	ENSG0000(	3.05	34.379	-3.127	4.14E-55
C2orf82	ENSG0000(	1.35	14.65	-2.735	1.89E-45
C2orf88	ENSG0000(	1.12	92.937	-5.47	1.54E-76
C3orf18	ENSG0000(	4.93	30.89	-2.427	1.65E-53
C3orf20	ENSG0000(	0.01	7.78	-3.12	2.64E-53
C3orf22	ENSG0000(	0.02	65.662	-6.03	1.11E-69
C3orf30	ENSG0000(	0	93.687	-6.565	1.58E-73
C3orf62	ENSG0000(	4.41	26.319	-2.336	7.11E-59
C3orf67	ENSG0000(	5.81	38.51	-2.536	1.12E-38
C3orf84	ENSG0000(	0	9.38	-3.376	3.02E-59

C4orf17	ENSG0000(	0	18.9	-4.315	7.32E-62
C4orf22	ENSG0000(	0	36.78	-5.24	9.64E-76
C4orf36	ENSG0000(	1.59	19.86	-3.01	3.96E-53
C4orf45	ENSG0000(	0	16.59	-4.137	1.68E-64
C4orf47	ENSG0000(	1.55	21.891	-3.166	1.91E-43
C4orf48	ENSG0000(	16.381	68.75	-2.005	2.56E-28
C4orf51	ENSG0000(	0.47	9.52	-2.839	5.83E-25
C5orf46	ENSG0000(	0.39	22.59	-4.085	2.18E-55
C5orf47	ENSG0000(	0.12	21.97	-4.358	5.02E-88
C5orf49	ENSG0000(	0.23	47.589	-5.304	6.54E-63
C5orf52	ENSG0000(	0	45.48	-5.539	6.76E-74
C5orf58	ENSG0000(	5.32	180.818	-4.846	2.95E-54
C5orf60	ENSG0000(	0.3	52.37	-5.359	1.97E-66
C5orf63	ENSG0000(	1.34	16.35	-2.89	4.82E-104
C6	ENSG0000(	0.16	4.25	-2.178	1.41E-30
C6orf10	ENSG0000(	0.25	42.479	-5.12	2.56E-56
C6orf118	ENSG0000(	0.08	16.64	-4.03	1.63E-54
C6orf141	ENSG0000(	0.47	9.44	-2.828	1.21E-56
C6orf163	ENSG0000(	2.24	14.98	-2.302	3.47E-30
C6orf165	ENSG0000(	0.26	39.711	-5.014	1.14E-62
C6orf201	ENSG0000(	0.13	76.781	-6.105	1.57E-81
C6orf229	ENSG0000(	0	15.22	-4.02	6.22E-56
C6orf52	ENSG0000(	2.69	18.699	-2.416	5.90E-38
C6orf99	ENSG0000(	0.93	153.819	-6.326	1.64E-79
C7orf31	ENSG0000(	8.19	67.349	-2.895	1.52E-32
C7orf34	ENSG0000(	0.05	86.647	-6.383	3.25E-92
C7orf55	ENSG0000(	34.779	201.885	-2.503	7.50E-41
C7orf61	ENSG0000(	0.37	88.923	-6.036	4.04E-87
C7orf62	ENSG0000(	0	27.34	-4.825	2.59E-66
C7orf66	ENSG0000(	0	3.09	-2.032	4.59E-41
C7orf71	ENSG0000(	0.01	4.76	-2.512	1.05E-63
C7orf72	ENSG0000(	0	3.71	-2.236	1.17E-48
C8orf48	ENSG0000(	0.7	7.81	-2.374	1.35E-60
C8orf74	ENSG0000(	0	23.9	-4.638	1.50E-65
C8orf88	ENSG0000(	7.29	101.342	-3.626	2.88E-72
C8orf89	ENSG0000(	0.17	9.85	-3.213	2.21E-61
C9orf116	ENSG0000(	3.39	84.442	-4.283	8.13E-62
C9orf117	ENSG0000(	0.46	32.5	-4.52	1.61E-75
C9orf131	ENSG0000(	0.08	45.49	-5.428	1.82E-67
C9orf135-/	ENSG0000(	0.07	38.22	-5.196	1.34E-67
C9orf153	ENSG0000(	0.04	15.36	-3.976	7.76E-61
C9orf171	ENSG0000(	0.07	26.031	-4.659	4.55E-69
C9orf172	ENSG0000(	1.01	7.5	-2.08	3.82E-60
C9orf173	ENSG0000(	0.22	92.609	-6.262	1.02E-94
C9orf173-/	ENSG0000(	0.18	10.38	-3.27	1.66E-97
C9orf24	ENSG0000(	0.44	142.982	-6.644	4.80E-59
C9orf43	ENSG0000(	0.81	56.539	-4.99	4.25E-88
C9orf50	ENSG0000(	0.08	10.55	-3.419	1.56E-54
C9orf57	ENSG0000(	0	16.65	-4.142	9.08E-62

C9orf84	ENSG00000	0.16	11.79	-3.463	7.96E-112
C9orf9	ENSG00000	2.79	163.482	-5.44	2.74E-85
CA10	ENSG00000	0.09	19.24	-4.215	2.97E-59
CA5B	ENSG00000	1.7	9.87	-2.009	2.80E-58
CA8	ENSG00000	0.94	13.11	-2.863	1.44E-73
CA9	ENSG00000	2.25	35.7	-3.497	6.35E-38
CAB39L	ENSG00000	2.36	25.189	-2.962	4.24E-69
CABLES2	ENSG00000	2.24	39.921	-3.659	1.06E-69
CABS1	ENSG00000	0.02	128.479	-6.988	2.25E-67
CABYR	ENSG00000	17.47	401.316	-4.445	3.94E-41
CACNA1A	ENSG00000	0.77	6.59	-2.1	3.14E-46
CACNA1C	ENSG00000	1.14	16.23	-3.009	3.54E-29
CACNA1C-	ENSG00000	0	5.73	-2.751	3.44E-59
CACNA1H	ENSG00000	6.14	44.659	-2.677	1.17E-58
CACNA2D4	ENSG00000	1.81	15.7	-2.571	2.68E-51
CADM1	ENSG00000	6.91	50.59	-2.705	4.66E-39
CADPS	ENSG00000	0.59	7.75	-2.46	3.11E-19
CAGE1	ENSG00000	0.03	7.57	-3.057	5.88E-60
CAHM	ENSG00000	0.95	13.58	-2.902	1.58E-101
CALB2	ENSG00000	1.24	8.57	-2.095	6.48E-18
CALCOCO1	ENSG00000	17.111	83.678	-2.225	1.50E-54
CALCOCO2	ENSG00000	32.45	168.72	-2.343	2.81E-57
CALR3	ENSG00000	0.15	85.07	-6.226	2.91E-82
CALY	ENSG00000	0.98	11.2	-2.623	1.13E-50
CAMK1	ENSG00000	9.45	45.8	-2.163	4.36E-44
CAMK4	ENSG00000	1.4	53.66	-4.509	5.54E-57
CAMKK1	ENSG00000	2.25	12.48	-2.052	1.83E-58
CAND2	ENSG00000	3.55	21.63	-2.314	4.03E-70
CAPN11	ENSG00000	0.29	44.43	-5.138	6.19E-72
CAPN3	ENSG00000	3.63	78.01	-4.093	1.18E-87
CAPS2	ENSG00000	0.74	8.56	-2.458	6.53E-91
CAPSL	ENSG00000	0	11.04	-3.59	2.55E-49
CAPZA3	ENSG00000	0	226.944	-7.833	2.29E-74
CARF	ENSG00000	0.9	9.1	-2.41	2.91E-69
CARHSP1	ENSG00000	112.891	579.071	-2.349	4.00E-33
CARTPT	ENSG00000	0	16.01	-4.088	2.35E-53
CASC1	ENSG00000	1.19	21.86	-3.384	1.69E-56
CASC16	ENSG00000	0	25.64	-4.736	3.74E-65
CASC2	ENSG00000	0.53	11.41	-3.02	2.82E-80
CASC22	ENSG00000	0	11.71	-3.668	2.11E-59
CASC23	ENSG00000	0	10.68	-3.546	2.73E-59
CASQ1	ENSG00000	0.92	7.93	-2.218	7.00E-30
CAST	ENSG00000	17.75	125.931	-2.759	3.23E-57
CATIP	ENSG00000	0.46	10.72	-3.005	1.33E-59
CATSPER1	ENSG00000	0.29	26.58	-4.418	1.19E-61
CATSPER2	ENSG00000	1.12	51.339	-4.626	2.49E-116
CATSPER2I	ENSG00000	0.94	17.019	-3.215	8.01E-84
CATSPER3	ENSG00000	0.8	38.799	-4.467	2.07E-54
CATSPER4	ENSG00000	0.08	16.731	-4.037	2.95E-64

CATSPERD	ENSG0000(	0.12	28.46	-4.717	1.19E-70
CATSPERG	ENSG0000(	3.58	112.259	-4.628	6.66E-66
CBLN1	ENSG0000(	3.58	39.62	-3.149	7.61E-50
CBY3	ENSG0000(	0.17	19.821	-4.153	1.60E-68
CC2D2B	ENSG0000(	0.14	5.58	-2.529	5.53E-59
CCDC102B	ENSG0000(	2.7	16.33	-2.228	8.00E-36
CCDC103	ENSG0000(	0.53	32.73	-4.462	5.22E-61
CCDC105	ENSG0000(	0	20.959	-4.457	1.89E-62
CCDC108	ENSG0000(	0.33	23.02	-4.175	1.31E-68
CCDC110	ENSG0000(	0.35	67.162	-5.658	6.60E-80
CCDC113	ENSG0000(	1.99	19.53	-2.78	2.72E-55
CCDC114	ENSG0000(	0.68	61.141	-5.209	1.30E-85
CCDC116	ENSG0000(	0.11	38.529	-5.154	5.04E-84
CCDC121	ENSG0000(	2.56	28.249	-3.038	2.16E-55
CCDC126	ENSG0000(	7.91	53.02	-2.6	5.71E-42
CCDC13	ENSG0000(	0.44	12.41	-3.219	1.07E-79
CCDC136	ENSG0000(	9.86	355.596	-5.037	2.03E-65
CCDC144N	ENSG0000(	3.54	18.47	-2.1	2.43E-16
CCDC146	ENSG0000(	1.52	37.281	-3.925	1.13E-80
CCDC148	ENSG0000(	0.52	5.81	-2.164	6.38E-51
CCDC15	ENSG0000(	1.25	15.06	-2.835	1.19E-67
CCDC150	ENSG0000(	7.84	38.18	-2.148	1.22E-33
CCDC151	ENSG0000(	1.81	16.71	-2.656	5.74E-59
CCDC153	ENSG0000(	0.99	10.32	-2.508	7.67E-54
CCDC154	ENSG0000(	0.73	20.78	-3.654	2.52E-87
CCDC155	ENSG0000(	0.88	95.63	-5.684	1.12E-81
CCDC157	ENSG0000(	2.8	49.6	-3.735	4.86E-73
CCDC158	ENSG0000(	0.02	33.21	-5.068	3.26E-89
CCDC159	ENSG0000(	9.85	112.813	-3.391	2.08E-77
CCDC166	ENSG0000(	0	5.33	-2.662	1.93E-56
CCDC168	ENSG0000(	0	9.98	-3.457	3.85E-54
CCDC169	ENSG0000(	1.74	116.629	-5.424	6.30E-66
CCDC170	ENSG0000(	0.25	5	-2.263	8.63E-36
CCDC171	ENSG0000(	2.25	30.099	-3.258	2.41E-52
CCDC172	ENSG0000(	1.3	16.82	-2.954	1.59E-53
CCDC173	ENSG0000(	0.34	71.169	-5.751	8.46E-78
CCDC175	ENSG0000(	0.04	3.34	-2.061	1.37E-68
CCDC176	ENSG0000(	1.84	32.251	-3.549	1.74E-72
CCDC178	ENSG0000(	0.52	13.67	-3.271	1.09E-74
CCDC179	ENSG0000(	0	29.229	-4.918	2.75E-65
CCDC180	ENSG0000(	1.1	50.011	-4.602	1.52E-107
CCDC181	ENSG0000(	2.37	33.669	-3.363	4.97E-52
CCDC182	ENSG0000(	0	36.859	-5.243	2.03E-69
CCDC183	ENSG0000(	0.56	69.42	-5.496	1.65E-98
CCDC183-	ENSG0000(	0.33	6.41	-2.478	1.11E-79
CCDC184	ENSG0000(	0.69	7.92	-2.4	1.15E-49
CCDC185	ENSG0000(	0	45.54	-5.54	1.83E-66
CCDC186	ENSG0000(	2.59	13.97	-2.06	2.64E-62
CCDC24	ENSG0000(	6.9	55.598	-2.841	3.86E-70

CCDC27	ENSG0000(	0.01	24	-4.629	4.77E-66
CCDC28B	ENSG0000(	8.82	44.359	-2.208	1.76E-43
CCDC30	ENSG0000(	0.72	19.67	-3.587	3.30E-79
CCDC33	ENSG0000(	0.07	28.58	-4.789	2.75E-60
CCDC34	ENSG0000(	10.01	53.541	-2.309	3.92E-34
CCDC36	ENSG0000(	0.56	42.109	-4.788	8.02E-93
CCDC37	ENSG0000(	0.14	67.798	-5.915	7.52E-66
CCDC37-A	ENSG0000(	0	101.623	-6.681	2.73E-71
CCDC38	ENSG0000(	0.24	30.17	-4.652	9.07E-59
CCDC39	ENSG0000(	0.43	11.45	-3.122	1.55E-102
CCDC40	ENSG0000(	1	28.879	-3.901	3.82E-69
CCDC42	ENSG0000(	0.24	124.68	-6.663	6.48E-83
CCDC53	ENSG0000(	25.351	161.422	-2.624	3.05E-75
CCDC54	ENSG0000(	0.03	132.973	-7.023	4.74E-91
CCDC60	ENSG0000(	0.24	15.74	-3.755	5.30E-62
CCDC62	ENSG0000(	0.37	41.659	-4.961	1.46E-70
CCDC63	ENSG0000(	0.64	22.811	-3.86	4.85E-54
CCDC64	ENSG0000(	9.87	76.781	-2.839	5.22E-51
CCDC65	ENSG0000(	2.42	39.469	-3.565	2.36E-54
CCDC67	ENSG0000(	0.17	32.271	-4.83	8.26E-79
CCDC68	ENSG0000(	0.26	10.58	-3.2	3.75E-57
CCDC7	ENSG0000(	0.48	68.45	-5.552	1.09E-83
CCDC70	ENSG0000(	0	169.318	-7.412	3.41E-72
CCDC73	ENSG0000(	0.08	4.16	-2.256	5.49E-83
CCDC74A	ENSG0000(	9	108.872	-3.458	2.36E-96
CCDC74B	ENSG0000(	3.85	83.239	-4.118	8.56E-82
CCDC79	ENSG0000(	0.16	14.77	-3.765	1.28E-83
CCDC82	ENSG0000(	8.44	49.7	-2.425	4.62E-64
CCDC83	ENSG0000(	0	27.059	-4.81	8.64E-72
CCDC87	ENSG0000(	0.19	18.01	-3.998	9.48E-76
CCDC88A	ENSG0000(	11.55	49.969	-2.022	2.31E-40
CCDC89	ENSG0000(	0.13	52.621	-5.568	2.10E-80
CCDC91	ENSG0000(	16.75	192.084	-3.443	1.14E-49
CCDC92	ENSG0000(	12.7	96.488	-2.831	2.42E-51
CCDC96	ENSG0000(	0.91	85.041	-5.493	3.07E-74
CCER1	ENSG0000(	0.01	60.249	-5.922	5.40E-84
CCHCR1	ENSG0000(	32.39	186.275	-2.488	9.42E-50
CCIN	ENSG0000(	0.18	92.577	-6.309	1.90E-65
CCNA1	ENSG0000(	1.2	84.459	-5.28	1.70E-47
CCNB3	ENSG0000(	0.33	8.84	-2.887	2.37E-74
CCNH	ENSG0000(	19.83	87.65	-2.089	1.71E-70
CCNJL	ENSG0000(	1.9	16.999	-2.634	2.44E-63
CCNO	ENSG0000(	1.97	21.01	-2.89	1.17E-42
CCNY	ENSG0000(	27.2	285.767	-3.346	2.79E-53
CCNYL2	ENSG0000(	0.05	6.21	-2.78	4.47E-58
CCSER1	ENSG0000(	0.21	19.55	-4.086	2.48E-94
CCSER2	ENSG0000(	7.72	151.828	-4.131	7.50E-65
CCT6B	ENSG0000(	1.49	107.589	-5.447	3.73E-82
CCT8L2	ENSG0000(	0.01	25.5	-4.714	1.28E-62

CD200R1L	ENSG00000	0	3.37	-2.128	3.29E-43
CD300LG	ENSG00000	0.4	27.331	-4.339	1.53E-112
CDADC1	ENSG00000	4.13	32.45	-2.705	4.46E-86
CDC14A	ENSG00000	1.76	31.82	-3.572	1.08E-55
CDC14C	ENSG00000	0.02	3.19	-2.038	1.44E-53
CDC20B	ENSG00000	0.05	3.29	-2.031	1.75E-44
CDH12P2	ENSG00000	0	3.71	-2.236	1.09E-04
CDH18	ENSG00000	0.42	11.94	-3.188	3.58E-58
CDH23	ENSG00000	1.73	15.92	-2.632	3.46E-73
CDHR2	ENSG00000	0.14	36.481	-5.039	6.53E-52
CDHR4	ENSG00000	0.06	10.97	-3.497	3.62E-61
CDIPT-AS1	ENSG00000	0.74	150.936	-6.448	2.46E-77
CDK19	ENSG00000	3.44	22.45	-2.401	1.66E-64
CDK20	ENSG00000	2.93	27.429	-2.855	1.83E-82
CDKL2	ENSG00000	0.21	11.47	-3.365	1.75E-69
CDKL3	ENSG00000	0.95	23.801	-3.669	7.07E-86
CDKN2A	ENSG00000	2.92	26.07	-2.788	4.35E-37
CDKN2B	ENSG00000	1.3	9.73	-2.222	4.48E-44
CDKN2D	ENSG00000	8.97	50.811	-2.378	1.23E-56
CDKN3	ENSG00000	26.08	251.793	-3.223	3.32E-31
CDNF	ENSG00000	1.47	30.49	-3.672	2.61E-79
CDRT1	ENSG00000	0.37	17.33	-3.742	2.80E-78
CDRT15	ENSG00000	0.17	21.201	-4.246	7.03E-63
CDRT15L2	ENSG00000	0	20.599	-4.433	6.03E-75
CDRT15P1	ENSG00000	0.17	11.56	-3.424	1.71E-62
CDRT4	ENSG00000	2.2	41.949	-3.746	6.02E-63
CDRT8	ENSG00000	0	3.59	-2.199	1.32E-45
CDYL2	ENSG00000	1.55	9.93	-2.1	1.66E-54
CEACAM19	ENSG00000	2.26	18.84	-2.605	2.75E-59
CELA3A	ENSG00000	0.03	3.23	-2.038	3.52E-24
CELF2-AS1	ENSG00000	0.05	3.56	-2.119	1.36E-77
CELF3	ENSG00000	0.29	5.66	-2.368	5.63E-27
CELF6	ENSG00000	0.88	16.82	-3.245	4.16E-100
CENPU	ENSG00000	24.34	106.809	-2.089	7.86E-26
CEP112	ENSG00000	4.24	112.993	-4.443	2.54E-70
CEP126	ENSG00000	0.76	23.789	-3.816	2.09E-100
CEP162	ENSG00000	4.34	29.191	-2.499	1.03E-62
CEP164	ENSG00000	8.69	67.878	-2.83	5.25E-91
CEP164P1	ENSG00000	0.04	8.99	-3.264	4.97E-64
CEP170	ENSG00000	22.511	111.77	-2.262	4.07E-31
CEP170P1	ENSG00000	0.91	81.069	-5.425	1.66E-41
CEP295NL	ENSG00000	0.13	57.34	-5.69	1.32E-66
CEP350	ENSG00000	7.46	85.68	-3.357	3.46E-65
CEP41	ENSG00000	7.14	91.201	-3.502	2.57E-50
CEP57L1	ENSG00000	5.66	26.02	-2.02	1.17E-61
CEP70	ENSG00000	7.82	51.62	-2.577	5.20E-50
CEP83-AS1	ENSG00000	0.36	14.33	-3.495	7.76E-76
CEP85L	ENSG00000	3.8	46.16	-3.296	2.51E-51
CERS1	ENSG00000	8.48	37.519	-2.023	1.65E-45

CERS3	ENSG00000	0.2	16.68	-3.881	1.13E-66
CES4A	ENSG00000	1.9	16.151	-2.564	1.10E-80
CETN1	ENSG00000	0.1	166.34	-7.249	1.03E-80
CETN3	ENSG00000	26.009	167.753	-2.643	5.19E-51
CFAP126	ENSG00000	0.08	31.449	-4.909	7.64E-45
CFAP221	ENSG00000	0.87	47.389	-4.694	3.89E-69
CFAP43	ENSG00000	1.02	56.519	-4.832	4.75E-66
CFAP44	ENSG00000	1.82	19.5	-2.862	9.13E-71
CFAP44-AS	ENSG00000	0	6.51	-2.909	3.46E-48
CFAP45	ENSG00000	0.45	47.408	-5.061	4.62E-65
CFAP46	ENSG00000	1.02	25.989	-3.74	1.77E-68
CFAP52	ENSG00000	0.19	17.64	-3.969	1.83E-66
CFAP53	ENSG00000	0.58	97.113	-5.956	3.04E-83
CFAP57	ENSG00000	0.31	19.191	-3.946	3.77E-72
CFAP58	ENSG00000	0.44	17.321	-3.669	6.96E-64
CFAP61	ENSG00000	0.35	21.93	-4.086	5.48E-63
CFAP69	ENSG00000	1.36	11.28	-2.379	4.77E-64
CFAP70	ENSG00000	1.19	44.099	-4.364	1.49E-96
CFAP74	ENSG00000	0.13	18.77	-4.129	5.02E-88
CFAP99	ENSG00000	0.06	19.231	-4.254	3.18E-80
CFD	ENSG00000	10.81	57.221	-2.302	4.73E-21
CH17-140	ENSG00000	0.16	63.319	-5.793	1.52E-66
CH17-264	ENSG00000	0	3.2	-2.07	2.11E-17
CH17-53B	ENSG00000	0	5.53	-2.707	7.87E-71
CH507-21	ENSG00000	0.02	3.18	-2.035	9.85E-35
CH507-39	ENSG00000	0.24	6.97	-2.684	1.39E-27
CH507-42	ENSG00000	0.06	3.93	-2.218	1.24E-66
CHCHD2P	ENSG00000	1.24	8.18	-2.035	1.35E-51
CHCHD6	ENSG00000	18.13	77.6	-2.039	3.84E-45
CHD5	ENSG00000	0.38	73.981	-5.764	4.28E-87
CHDH	ENSG00000	0.96	7.64	-2.14	2.61E-52
CHGA	ENSG00000	4.06	98.632	-4.299	5.04E-52
CHGB	ENSG00000	2.79	16.53	-2.21	2.06E-26
CHKA	ENSG00000	10.17	123.536	-3.479	7.82E-80
CHMP4BP1	ENSG00000	0.73	6.86	-2.184	8.60E-55
CHODL	ENSG00000	3.44	28.719	-2.743	1.04E-43
CHP2	ENSG00000	0.14	5.68	-2.551	5.60E-42
CHRM3-AS	ENSG00000	0.38	26.829	-4.334	2.28E-85
CHRM4	ENSG00000	0.05	6.31	-2.8	3.15E-65
CHST1	ENSG00000	2.48	16.48	-2.329	2.39E-33
CHST13	ENSG00000	0.97	24.07	-3.67	4.61E-44
CHST3	ENSG00000	1.7	22.29	-3.109	2.65E-59
CIB4	ENSG00000	0.05	22.441	-4.481	9.84E-61
CITED1	ENSG00000	1.99	243.216	-6.352	2.26E-71
CITED4	ENSG00000	3.67	31.43	-2.796	2.92E-54
CKLF-CMT	ENSG00000	0.65	11.79	-2.954	6.85E-64
CKM	ENSG00000	0.27	12.73	-3.434	3.28E-64
CKMT2	ENSG00000	1.22	8.21	-2.053	1.60E-53
CLCN2	ENSG00000	3.56	32.52	-2.878	1.08E-74

CLCNKA	ENSG00000	0.35	9.17	-2.913	9.35E-68
CLDN11	ENSG00000	3.31	195.482	-5.511	2.98E-58
CLDND2	ENSG00000	1.14	57.412	-4.771	1.72E-79
CLEC12B	ENSG00000	0.31	20.97	-4.068	8.06E-103
CLEC16A	ENSG00000	8.42	79.621	-3.097	1.51E-90
CLEC18A	ENSG00000	0.51	8.92	-2.716	2.67E-49
CLEC18B	ENSG00000	0.6	25.01	-4.023	1.39E-52
CLEC18C	ENSG00000	0.27	8.75	-2.941	1.09E-45
CLEC3B	ENSG00000	3.06	147.983	-5.198	1.72E-62
CLEC4M	ENSG00000	0.05	4.71	-2.443	1.80E-44
CLGN	ENSG00000	0.35	63.928	-5.588	4.54E-73
CLIC5	ENSG00000	0.46	8.36	-2.681	3.67E-44
CLIP1-AS1	ENSG00000	0	4.77	-2.529	1.24E-53
CLIP4	ENSG00000	2.5	29.51	-3.124	4.77E-68
CLK2P1	ENSG00000	0.04	37.579	-5.213	5.92E-76
CLK4	ENSG00000	5.75	36.511	-2.474	2.27E-93
CLMN	ENSG00000	4.98	99.753	-4.075	9.33E-61
CLPB	ENSG00000	9.94	431.225	-5.304	2.94E-59
CLPS	ENSG00000	0	3.65	-2.217	1.65E-17
CLTCL1	ENSG00000	3.82	67.409	-3.827	1.45E-86
CLU	ENSG00000	159.575	3440.016	-4.422	2.70E-47
CLYBL-AS1	ENSG00000	0	7.61	-3.106	2.24E-57
CMTM1	ENSG00000	0.5	69.261	-5.55	3.41E-73
CMTM2	ENSG00000	0.18	862.212	-9.515	4.92E-88
CNBD1	ENSG00000	0	10.72	-3.551	2.16E-61
CNBD2	ENSG00000	2.01	34.41	-3.556	5.14E-70
CNGA4	ENSG00000	0.12	7.93	-2.995	4.89E-63
CNKSR3	ENSG00000	2.31	13.36	-2.117	1.74E-25
CNN1	ENSG00000	8.67	119.46	-3.639	2.09E-45
CNNM1	ENSG00000	0.65	13.12	-3.097	2.55E-87
CNTD1	ENSG00000	0.77	55.391	-4.994	1.53E-95
CNTFR-AS1	ENSG00000	0	14.54	-3.958	1.27E-63
CNTN1	ENSG00000	0.73	7.44	-2.286	2.70E-31
CNTN2	ENSG00000	0.12	10.99	-3.42	2.62E-42
CNTN4	ENSG00000	1.29	14.41	-2.75	8.35E-36
CNTRL	ENSG00000	6.99	45.079	-2.528	3.12E-60
COIL	ENSG00000	11.73	69.569	-2.471	9.76E-46
COL11A1	ENSG00000	1.24	10.67	-2.381	4.18E-13
COL11A2	ENSG00000	0.64	13.61	-3.155	4.83E-55
COL16A1	ENSG00000	6.96	125.287	-3.988	1.22E-39
COL20A1	ENSG00000	1.49	26.58	-3.469	1.97E-59
COL25A1	ENSG00000	0.21	27.441	-4.555	5.64E-66
COL28A1	ENSG00000	0.71	6.46	-2.125	4.59E-42
COL7A1	ENSG00000	9.61	54.281	-2.381	4.35E-23
COL9A1	ENSG00000	0.41	8.97	-2.822	4.96E-17
COLCA1	ENSG00000	0.33	10.34	-3.092	1.70E-70
COLCA2	ENSG00000	0.52	14.03	-3.306	4.69E-77
COLQ	ENSG00000	1.01	7.69	-2.112	5.11E-57
COMP	ENSG00000	3.17	20.17	-2.344	1.05E-16

COPRS	ENSG00000	45.361	310.402	-2.748	7.30E-60
COPZ2	ENSG00000	4.97	33.339	-2.524	3.67E-21
COX6B2	ENSG00000	0.96	188.719	-6.597	9.50E-79
COX7B2	ENSG00000	0.73	106.668	-5.96	1.16E-66
COX7CP1	ENSG00000	0	4.33	-2.414	9.09E-30
COX8C	ENSG00000	4.36	97.059	-4.193	1.34E-41
CPA1	ENSG00000	0.09	6.29	-2.742	4.90E-41
CPA5	ENSG00000	0.25	48.778	-5.316	1.59E-65
CPB1	ENSG00000	0.03	3.16	-2.014	7.67E-34
CPD	ENSG00000	7.75	49.511	-2.529	1.23E-44
CPE	ENSG00000	12.28	95.722	-2.865	1.43E-24
CPEB2	ENSG00000	1.94	46.5	-4.014	3.29E-91
CPEB2-AS1	ENSG00000	0.01	6.75	-2.94	6.65E-73
CPEB3	ENSG00000	1.03	16.71	-3.125	1.50E-66
CPEB4	ENSG00000	1.79	12.15	-2.237	4.77E-68
CPLX3	ENSG00000	0.11	8.87	-3.152	7.27E-82
CPNE5	ENSG00000	3.91	34.709	-2.863	1.06E-48
CPT1B	ENSG00000	14.07	138.169	-3.207	5.12E-65
CPTP	ENSG00000	11.99	91.868	-2.838	1.19E-63
CPXCR1	ENSG00000	0	5.09	-2.606	1.74E-55
CRADD	ENSG00000	5.93	29.821	-2.153	2.18E-65
CRAT	ENSG00000	8.67	226.096	-4.554	2.08E-85
CRB1	ENSG00000	0.03	3.92	-2.256	5.53E-75
CREB1	ENSG00000	11.62	65.109	-2.389	3.79E-40
CREM	ENSG00000	23.489	360.435	-3.884	1.51E-70
CRIP3	ENSG00000	2.7	54.879	-3.917	3.57E-67
CRIPAK	ENSG00000	1.92	19.53	-2.814	2.15E-96
CRISP2	ENSG00000	0.12	612.259	-9.097	1.57E-79
CRNDE	ENSG00000	9.87	282.282	-4.704	2.14E-59
CRY2	ENSG00000	10.72	50.959	-2.148	6.87E-63
CRYAB	ENSG00000	4.26	28.521	-2.489	2.16E-20
CRYBA2	ENSG00000	0.25	6.04	-2.494	2.01E-47
CRYBG3	ENSG00000	1.97	25.91	-3.18	6.15E-71
CSAD	ENSG00000	9.88	64.592	-2.592	1.35E-56
CSAG3	ENSG00000	0.27	5.1	-2.264	1.60E-24
CSDAP1	ENSG00000	0.06	6.21	-2.766	3.38E-66
CSDC2	ENSG00000	0.49	36.091	-4.638	2.21E-89
CSE1L-AS1	ENSG00000	0.06	16.85	-4.074	6.10E-70
CSMD2-AS	ENSG00000	0	102.757	-6.697	2.72E-73
CSNK1A1L	ENSG00000	0	3.46	-2.157	1.77E-50
CSNK1G1	ENSG00000	3.91	20.91	-2.158	3.33E-44
CSNK1G2	ENSG00000	37.59	242.913	-2.66	2.34E-63
CSNK1G2-	ENSG00000	0.02	14.28	-3.905	2.36E-64
CSNK2A2	ENSG00000	42.22	181.785	-2.08	3.23E-48
CST13P	ENSG00000	0	38.62	-5.308	1.66E-67
CST3	ENSG00000	249.05	1000.149	-2.001	1.94E-35
CST8	ENSG00000	0	27.389	-4.827	6.99E-66
CST9L	ENSG00000	0	80.097	-6.342	2.40E-102
CSTL1	ENSG00000	0.06	11.24	-3.529	2.12E-56

CT45A1	ENSG0000(	0.17	8.44	-3.012	2.17E-23
CT45A10	ENSG0000(	0.13	7.59	-2.926	1.03E-26
CT45A3	ENSG0000(	0.17	12.81	-3.561	1.84E-25
CT45A5	ENSG0000(	0.13	4.08	-2.168	1.10E-16
CT47B1	ENSG0000(	0	8.28	-3.214	1.61E-75
CT55	ENSG0000(	0.16	4.9	-2.347	1.15E-57
CT62	ENSG0000(	0.06	38.149	-5.207	7.41E-69
CT83	ENSG0000(	0.9	73.541	-5.294	9.77E-58
CTA-125H	ENSG0000(	0	18.1	-4.256	2.34E-61
CTA-299D	ENSG0000(	0.02	5.9	-2.758	2.14E-56
CTA-398F1	ENSG0000(	0	4.89	-2.558	2.65E-46
CTA-407F1	ENSG0000(	0	29.871	-4.948	2.77E-74
CTAG1A	ENSG0000(	0.21	15.47	-3.767	1.21E-40
CTAG2	ENSG0000(	1.45	12.98	-2.513	3.02E-19
CTAGE1	ENSG0000(	0	11.94	-3.694	3.81E-76
CTAGE10P	ENSG0000(	0.02	5.68	-2.711	1.06E-64
CTAGE11P	ENSG0000(	0.01	4.59	-2.468	2.82E-55
CTAGE13P	ENSG0000(	0	5.76	-2.757	2.10E-65
CTAGE15	ENSG0000(	0.29	9.25	-2.99	7.41E-40
CTAGE16P	ENSG0000(	0	4.14	-2.362	5.42E-56
CTAGE3P	ENSG0000(	0.34	8.66	-2.85	5.40E-44
CTAGE4	ENSG0000(	2.85	15.15	-2.069	3.95E-21
CTAGE6	ENSG0000(	0	14.55	-3.959	4.38E-63
CTAGE7P	ENSG0000(	0.17	6.59	-2.698	1.01E-77
CTAGE8	ENSG0000(	1.75	10.89	-2.112	4.68E-25
CTAGE9	ENSG0000(	0	9.64	-3.411	2.31E-63
CTB-134H:	ENSG0000(	0.1	10.4	-3.374	1.62E-66
CTB-147C2	ENSG0000(	0	6.79	-2.962	2.45E-51
CTB-164N:	ENSG0000(	0	3.09	-2.032	1.25E-55
CTB-167B5	ENSG0000(	0	4.36	-2.422	1.19E-58
CTB-174D:	ENSG0000(	0	24.45	-4.67	2.49E-67
CTB-181F2	ENSG0000(	0	10.56	-3.531	2.97E-51
CTB-31O2(	ENSG0000(	0.92	34.441	-4.206	8.12E-106
CTB-32O4.	ENSG0000(	0.12	7.41	-2.909	4.74E-90
CTB-33O1	ENSG0000(	0.03	6.05	-2.775	2.40E-64
CTB-35F21	ENSG0000(	0	30.02	-4.955	9.83E-73
CTB-50L17	ENSG0000(	0.3	7.91	-2.777	1.08E-67
CTB-55O6.	ENSG0000(	0.12	10.47	-3.356	2.41E-75
CTB-79E8.:	ENSG0000(	0	8.94	-3.313	3.06E-59
CTB-91J4.1	ENSG0000(	0	6.49	-2.905	1.20E-60
CTC-1337I	ENSG0000(	0.13	5.49	-2.522	1.68E-77
CTC-203F4	ENSG0000(	0.08	12.55	-3.649	7.19E-74
CTC-232P5	ENSG0000(	0	5.23	-2.639	3.59E-49
CTC-270D	ENSG0000(	0.63	11.84	-2.978	1.16E-59
CTC-273B:	ENSG0000(	0.25	25.029	-4.38	4.62E-67
CTC-308K2	ENSG0000(	0.32	6.02	-2.411	6.75E-86
CTC-338M	ENSG0000(	0	11.06	-3.592	9.48E-62
CTC-338M	ENSG0000(	0.94	33.92	-4.17	1.46E-87
CTC-338M	ENSG0000(	0	3.94	-2.305	1.07E-73

CTC-347C:	ENSG0000(	0	3.87	-2.284	7.10E-56
CTC-347C:	ENSG0000(	0	5.25	-2.644	1.96E-55
CTC-360P!	ENSG0000(	0	4.55	-2.473	1.01E-56
CTC-360P!	ENSG0000(	0	6	-2.807	1.33E-54
CTC-360P!	ENSG0000(	0	3.02	-2.007	8.05E-55
CTC-439O	ENSG0000(	0	4.3	-2.406	3.09E-47
CTC-441N	ENSG0000(	0	256.745	-8.01	6.17E-76
CTC-453G:	ENSG0000(	0	8.37	-3.228	1.44E-57
CTC-459F4	ENSG0000(	0	3.09	-2.032	1.85E-34
CTC-459M	ENSG0000(	0	12.08	-3.709	4.57E-61
CTC-471J1	ENSG0000(	1.05	7.88	-2.115	1.23E-68
CTC-479C!	ENSG0000(	0	4.89	-2.558	1.01E-62
CTC-523E:	ENSG0000(	0.14	17.38	-4.011	2.51E-62
CTC-523E:	ENSG0000(	0	4.69	-2.508	1.58E-61
CTC-523E:	ENSG0000(	0.08	4.45	-2.335	1.57E-58
CTC-523E:	ENSG0000(	0.07	5.56	-2.616	6.01E-68
CTC-523E:	ENSG0000(	0.05	11.92	-3.621	2.81E-78
CTC-527H	ENSG0000(	0	13.07	-3.815	2.39E-61
CTC-543D	ENSG0000(	0.7	8.32	-2.455	1.35E-74
CTC-559E!	ENSG0000(	0	4.26	-2.395	7.47E-57
CTC-774J1	ENSG0000(	0	3.34	-2.118	9.81E-55
CTCFL	ENSG0000(	0.2	9.11	-3.075	1.60E-77
CTD-2006(	ENSG0000(	4.33	23.91	-2.225	1.27E-68
CTD-2008I	ENSG0000(	0.13	26.891	-4.625	9.55E-85
CTD-2010I	ENSG0000(	0	6.41	-2.889	6.00E-58
CTD-2012.	ENSG0000(	1.2	15.76	-2.929	8.97E-05
CTD-2014I	ENSG0000(	0.04	10.71	-3.493	3.54E-72
CTD-2014I	ENSG0000(	0	4.74	-2.521	3.86E-52
CTD-2014I	ENSG0000(	0.06	4.01	-2.241	1.32E-59
CTD-2015(	ENSG0000(	0	17.73	-4.227	3.87E-63
CTD-2015I	ENSG0000(	1.94	14.26	-2.376	5.04E-53
CTD-2021.	ENSG0000(	1.2	55.749	-4.689	1.03E-56
CTD-2021.	ENSG0000(	2.24	54.829	-4.107	2.42E-49
CTD-2022I	ENSG0000(	0	4.19	-2.376	5.46E-73
CTD-2022I	ENSG0000(	0.05	5.94	-2.725	6.82E-78
CTD-2024I	ENSG0000(	0	4.9	-2.561	3.03E-37
CTD-2033(	ENSG0000(	0.35	4.94	-2.138	3.51E-81
CTD-2033I	ENSG0000(	0	4.72	-2.516	1.02E-56
CTD-2036I	ENSG0000(	0.26	6.4	-2.554	9.61E-58
CTD-2050I	ENSG0000(	0	3.6	-2.202	1.15E-57
CTD-2054I	ENSG0000(	1.45	18.35	-2.982	6.80E-78
CTD-2071I	ENSG0000(	0	15.61	-4.054	4.75E-53
CTD-2071I	ENSG0000(	0	5.74	-2.753	2.09E-53
CTD-2083I	ENSG0000(	0.09	12.91	-3.674	9.94E-78
CTD-2086(	ENSG0000(	0.03	4.74	-2.478	1.78E-62
CTD-2105I	ENSG0000(	0.04	12.51	-3.699	2.36E-74
CTD-2116I	ENSG0000(	0.12	20.069	-4.234	4.71E-60
CTD-2118I	ENSG0000(	0.05	23.209	-4.527	7.17E-64
CTD-2135I	ENSG0000(	0.02	5.8	-2.737	3.85E-62

CTD-2143	ENSG0000	0	9.16	-3.345	1.81E-58
CTD-2144	ENSG0000	0.62	10.23	-2.793	1.33E-35
CTD-2151.	ENSG0000	0	4.7	-2.511	1.84E-52
CTD-2170	ENSG0000	0	9.67	-3.416	1.52E-57
CTD-2184	ENSG0000	0.18	58.088	-5.646	1.43E-74
CTD-2194	ENSG0000	0	5.05	-2.597	1.86E-66
CTD-2194	ENSG0000	0.02	3.22	-2.049	4.85E-63
CTD-2200	ENSG0000	0.28	11.48	-3.285	9.45E-69
CTD-2201	ENSG0000	0.87	8.78	-2.387	5.13E-66
CTD-2201	ENSG0000	0.03	5.61	-2.682	1.37E-57
CTD-2201	ENSG0000	0.07	3.72	-2.141	3.30E-65
CTD-2210	ENSG0000	0	103.982	-6.714	1.69E-86
CTD-2210	ENSG0000	0	4.46	-2.449	5.53E-51
CTD-2228	ENSG0000	7.41	160.362	-4.262	4.47E-77
CTD-2231	ENSG0000	0	9.3	-3.365	7.78E-53
CTD-2260.	ENSG0000	0.41	4.86	-2.055	1.38E-84
CTD-2267	ENSG0000	0.1	8.72	-3.143	6.00E-73
CTD-2270	ENSG0000	1.24	14.69	-2.808	1.40E-80
CTD-2270	ENSG0000	0.35	4.62	-2.058	1.58E-72
CTD-2277	ENSG0000	0	5.28	-2.651	5.05E-62
CTD-2281	ENSG0000	0	3.08	-2.029	1.08E-44
CTD-2291	ENSG0000	0	5.26	-2.646	9.18E-54
CTD-2296	ENSG0000	0	8.33	-3.222	7.84E-57
CTD-2296	ENSG0000	0	19.44	-4.353	3.92E-55
CTD-2303	ENSG0000	4.11	23.02	-2.233	9.63E-28
CTD-2313	ENSG0000	0	6.98	-2.996	1.58E-47
CTD-2314	ENSG0000	0	8.56	-3.257	2.00E-74
CTD-2329	ENSG0000	0	3.18	-2.064	1.67E-58
CTD-2330	ENSG0000	0	9.2	-3.351	8.81E-61
CTD-2330	ENSG0000	0.06	9.64	-3.327	1.37E-59
CTD-2350.	ENSG0000	0	5.16	-2.623	6.86E-65
CTD-2376	ENSG0000	0.66	8.8	-2.562	1.83E-57
CTD-2501	ENSG0000	0	3.9	-2.293	1.57E-51
CTD-2506	ENSG0000	0.85	10.33	-2.615	2.59E-55
CTD-2514	ENSG0000	0.47	6.1	-2.272	1.42E-30
CTD-2517	ENSG0000	1.12	9.59	-2.321	3.99E-77
CTD-2520	ENSG0000	0	19.39	-4.35	6.73E-63
CTD-2528	ENSG0000	0	5.21	-2.635	4.45E-56
CTD-2530	ENSG0000	1.16	14.49	-2.842	7.39E-48
CTD-2537	ENSG0000	4.43	30.519	-2.537	1.40E-66
CTD-2538	ENSG0000	1.47	68.94	-4.824	4.34E-73
CTD-2538	ENSG0000	0	6.3	-2.868	6.70E-67
CTD-2542	ENSG0000	0	7.57	-3.099	2.01E-60
CTD-2544	ENSG0000	0	6.56	-2.918	3.23E-61
CTD-2553	ENSG0000	2.33	15.41	-2.301	2.29E-67
CTD-2560	ENSG0000	0	9.5	-3.392	1.09E-67
CTD-2568.	ENSG0000	0.07	267.148	-7.969	1.43E-76
CTD-2568	ENSG0000	0	13.44	-3.852	1.96E-66
CTD-2576	ENSG0000	0.23	6.18	-2.545	2.20E-76

CTD-2583I	ENSG0000(	0.03	3.22	-2.035	9.60E-52
CTD-2586I	ENSG0000(	0	7.9	-3.154	1.79E-57
CTD-2588(	ENSG0000(	0.06	6.13	-2.75	6.80E-64
CTD-2623I	ENSG0000(	0.03	3.17	-2.017	1.72E-63
CTD-2623I	ENSG0000(	0	5.04	-2.595	6.86E-66
CTD-2631I	ENSG0000(	0	4.98	-2.58	3.41E-66
CTD-2647I	ENSG0000(	0	10.27	-3.494	9.16E-51
CTD-2649(	ENSG0000(	0.13	11.53	-3.471	2.85E-77
CTD-2650I	ENSG0000(	0.35	6.32	-2.439	3.38E-61
CTD-2655I	ENSG0000(	0.06	17.39	-4.117	3.36E-80
CTD-2666I	ENSG0000(	2.2	15.57	-2.372	2.34E-50
CTD-3028I	ENSG0000(	0	9.98	-3.457	1.62E-62
CTD-3035I	ENSG0000(	0.62	13.66	-3.178	4.43E-75
CTD-3074(	ENSG0000(	0	3.92	-2.299	1.89E-56
CTD-3074(	ENSG0000(	1.78	22.73	-3.094	8.17E-71
CTD-3080I	ENSG0000(	0.09	4.95	-2.449	6.37E-40
CTD-3088(	ENSG0000(	0.7	6.42	-2.126	4.70E-56
CTD-3154I	ENSG0000(	0.5	5.66	-2.151	1.53E-59
CTD-3162I	ENSG0000(	12.69	78.27	-2.534	1.89E-23
CTD-3179I	ENSG0000(	0.05	4.48	-2.384	7.81E-60
CTD-3185I	ENSG0000(	1.44	14.99	-2.712	9.80E-69
CTD-3187I	ENSG0000(	0	22.119	-4.531	3.12E-72
CTD-3194(	ENSG0000(	0	3.56	-2.189	7.39E-51
CTD-3220I	ENSG0000(	8.57	116.969	-3.624	2.07E-40
CTGLF11P	ENSG0000(	0.27	7.67	-2.771	1.67E-55
CTGLF12P	ENSG0000(	1.04	18.04	-3.222	4.40E-65
CTNNA2	ENSG0000(	0.47	24.609	-4.123	3.16E-79
CTNNA3	ENSG0000(	0.47	11.1	-3.041	5.79E-62
CTRB1	ENSG0000(	0	3.38	-2.131	6.92E-24
CTRB2	ENSG0000(	0.04	3.69	-2.173	4.00E-20
CTSF	ENSG0000(	8	184.17	-4.363	1.26E-89
CTSG	ENSG0000(	0.06	4.06	-2.255	4.54E-29
CUL3	ENSG0000(	49.05	271.497	-2.445	5.60E-56
CUL9	ENSG0000(	20.5	92.5	-2.121	3.71E-59
CXADRP2	ENSG0000(	0	28.41	-4.878	1.04E-76
CXADRP3	ENSG0000(	0.06	13.32	-3.756	2.30E-71
CXCL16	ENSG0000(	23.091	203.543	-3.086	8.72E-33
CXorf49	ENSG0000(	0.17	7.41	-2.846	3.63E-29
CXorf49B	ENSG0000(	0.17	4.66	-2.274	5.42E-13
CXorf51B	ENSG0000(	0	164.688	-7.372	8.74E-81
CXorf58	ENSG0000(	0.08	3.37	-2.017	2.16E-68
CXorf65	ENSG0000(	0.97	49.121	-4.669	5.22E-49
CXorf66	ENSG0000(	0	16.979	-4.168	2.39E-70
CYB5D2	ENSG0000(	7.63	40.961	-2.282	3.34E-56
CYB5R2	ENSG0000(	15.83	188.105	-3.49	9.09E-61
CYB5R4	ENSG0000(	6.04	33.109	-2.276	5.39E-37
CYLC1	ENSG0000(	0	36.46	-5.227	2.02E-70
CYLC2	ENSG0000(	0	89.498	-6.5	1.91E-68
CYP11A1	ENSG0000(	2.09	33.27	-3.471	3.14E-47

CYP11B1	ENSG0000(	0	5.28	-2.651	4.88E-68
CYP17A1	ENSG0000(	0.15	15.25	-3.821	5.65E-31
CYP17A1-/	ENSG0000(	0.18	20.669	-4.199	2.27E-79
CYP19A1	ENSG0000(	0.16	21.17	-4.256	9.19E-71
CYP26B1	ENSG0000(	1.04	16.58	-3.107	2.79E-53
CYP2C8	ENSG0000(	1.1	9.23	-2.284	1.32E-36
CYP4F26P	ENSG0000(	0.85	12.7	-2.889	4.06E-33
CYP4F29P	ENSG0000(	1.43	19.8	-3.098	2.71E-32
CYP4F30P	ENSG0000(	0.13	29.821	-4.77	4.87E-75
CYP4F32P	ENSG0000(	0	18.29	-4.27	6.71E-70
CYP4F34P	ENSG0000(	0	11.86	-3.685	4.92E-80
CYP4F35P	ENSG0000(	0.52	20.26	-3.806	1.61E-44
CYP4F62P	ENSG0000(	0.05	20.4	-4.349	4.60E-61
CYP51A1-/	ENSG0000(	0.07	3.66	-2.123	5.46E-73
DAAM2	ENSG0000(	2.28	20.45	-2.709	2.75E-43
DAB1-AS1	ENSG0000(	0	49.59	-5.661	5.48E-70
DACH1	ENSG0000(	0.4	4.94	-2.085	1.32E-30
DACT3	ENSG0000(	1.55	22.841	-3.225	1.39E-34
DACT3-AS	ENSG0000(	0.19	9.4	-3.128	2.76E-62
DAW1	ENSG0000(	0.05	44.029	-5.422	1.71E-73
DAZ1	ENSG0000(	0.12	6.92	-2.822	1.66E-49
DAZL	ENSG0000(	0.62	41.711	-4.721	5.01E-19
DBIL5P	ENSG0000(	0.68	37.32	-4.512	7.83E-73
DCAF10	ENSG0000(	5.96	43.74	-2.684	8.69E-63
DCAF12L1	ENSG0000(	0.61	6.09	-2.139	4.72E-55
DCAF12L2	ENSG0000(	0.33	5.79	-2.352	8.20E-55
DCAF4L1	ENSG0000(	0.35	22.83	-4.142	9.71E-22
DCAF8L2	ENSG0000(	0.19	19.44	-4.102	4.45E-80
DCC	ENSG0000(	0.12	26.13	-4.598	1.64E-61
DCDC1	ENSG0000(	0.11	5.13	-2.465	1.01E-66
DCDC2C	ENSG0000(	0.02	6.28	-2.835	8.17E-61
DCST1	ENSG0000(	0.04	10.39	-3.453	4.15E-66
DCST2	ENSG0000(	1.21	24.69	-3.539	2.51E-62
DCUN1D1	ENSG0000(	10.53	303.488	-4.723	3.05E-58
DDAH1	ENSG0000(	2.28	14.98	-2.285	1.38E-24
DDI1	ENSG0000(	0	30.98	-4.999	1.89E-67
DDX11L1	ENSG0000(	0.27	4.14	-2.017	1.41E-49
DDX11L2	ENSG0000(	1.57	15.401	-2.674	2.55E-56
DDX20	ENSG0000(	10.32	67.991	-2.608	1.83E-46
DDX25	ENSG0000(	3.43	103.371	-4.558	4.21E-63
DDX4	ENSG0000(	2.02	131.607	-5.456	9.96E-59
DDX53	ENSG0000(	0.71	5.91	-2.015	2.08E-40
DEFB119	ENSG0000(	0.08	453.575	-8.717	8.62E-83
DEFB121	ENSG0000(	0	6.17	-2.842	2.51E-54
DEFB123	ENSG0000(	0.83	165.742	-6.51	4.43E-82
DEFB124	ENSG0000(	2.55	15.41	-2.209	3.32E-23
DENND6A	ENSG0000(	0	3.83	-2.272	8.70E-64
DENND6B	ENSG0000(	3.47	22.36	-2.386	3.66E-52
DEPDC7	ENSG0000(	4.89	24.981	-2.141	1.55E-27

DESI1	ENSG00000	15.11	65.871	-2.053	2.92E-53
DFNB31	ENSG00000	4.96	82.458	-3.808	3.27E-112
DFNB59	ENSG00000	1.55	44.12	-4.145	1.71E-103
DGAT2	ENSG00000	3.66	52.388	-3.518	6.43E-40
DGCR5	ENSG00000	0.59	6.78	-2.291	1.60E-41
DGCR6	ENSG00000	12.54	53.511	-2.009	1.39E-30
DGCR9	ENSG00000	0.22	6.45	-2.61	1.28E-49
DHH	ENSG00000	0.1	22.23	-4.4	3.44E-100
DHRS2	ENSG00000	0.33	5.36	-2.258	3.33E-25
DHRS4L1	ENSG00000	0.22	5.99	-2.518	4.94E-67
DHRS9	ENSG00000	1.76	13.42	-2.385	2.39E-23
DIAPH3	ENSG00000	3.25	20.11	-2.312	3.31E-30
DIAPH3-AS	ENSG00000	0	8	-3.17	1.23E-50
DIAPH3-AS	ENSG00000	0	12.43	-3.747	5.21E-59
DICER1-AS	ENSG00000	3.61	23.03	-2.382	1.44E-65
DIO3OS	ENSG00000	0.65	27.05	-4.087	4.02E-46
DISP1	ENSG00000	0.85	8.17	-2.309	1.19E-41
DKFZP434H	ENSG00000	0	4.89	-2.558	1.79E-59
DKFZp434J	ENSG00000	2.31	12.29	-2.005	4.32E-36
DKFZP434K	ENSG00000	0	15.89	-4.078	4.97E-63
DKFZp779M	ENSG00000	0.1	4.21	-2.244	4.78E-57
DKKL1	ENSG00000	4.45	466.133	-6.421	1.34E-60
DLEC1	ENSG00000	0.34	31.089	-4.582	8.27E-101
DLG1-AS1	ENSG00000	0.05	6.79	-2.891	1.11E-68
DLG2	ENSG00000	0.52	5.66	-2.131	3.39E-34
DLG4	ENSG00000	8.03	46.131	-2.384	1.49E-54
DLGAP1	ENSG00000	0.71	6.24	-2.082	3.71E-48
DLGAP2	ENSG00000	0.06	14.64	-3.883	7.82E-64
DLGAP2-A	ENSG00000	0	5.3	-2.655	9.56E-52
DLGAP4-A	ENSG00000	0.88	8.95	-2.404	1.67E-63
DLX6	ENSG00000	0.1	7.73	-2.989	3.49E-86
DMGDH	ENSG00000	0.49	5.64	-2.156	5.84E-101
DMRT1	ENSG00000	3.59	26.44	-2.58	5.82E-23
DMRT3	ENSG00000	0.32	12.29	-3.332	4.80E-73
DMRTB1	ENSG00000	0.85	86.737	-5.568	1.74E-72
DMRTC1B	ENSG00000	0.21	11.42	-3.36	9.69E-57
DMRTC2	ENSG00000	0.07	60.249	-5.839	2.17E-100
DNAAF1	ENSG00000	0.85	132.119	-6.169	4.71E-70
DNAAF3	ENSG00000	2.29	141.807	-5.44	4.57E-64
DNAH1	ENSG00000	2.04	32.71	-3.471	4.19E-94
DNAH10	ENSG00000	1.91	15.83	-2.532	8.03E-25
DNAH12	ENSG00000	0.12	20.269	-4.247	2.04E-81
DNAH14	ENSG00000	5.72	34.961	-2.42	7.23E-45
DNAH17	ENSG00000	1.04	38.799	-4.286	3.12E-77
DNAH2	ENSG00000	0.11	8.65	-3.12	2.48E-64
DNAH3	ENSG00000	0.33	6.84	-2.559	5.39E-66
DNAH6	ENSG00000	0.13	9.81	-3.258	4.45E-62
DNAH7	ENSG00000	0.11	14.65	-3.818	1.77E-78
DNAH8	ENSG00000	0.28	6.37	-2.526	1.36E-58

DNAH9	ENSG00000	0.65	14.36	-3.219	8.08E-58
DNAI1	ENSG00000	0.77	54.24	-4.964	5.01E-71
DNAI2	ENSG00000	0.05	49.99	-5.602	1.28E-78
DNAJA4	ENSG00000	15.51	185.991	-3.502	1.33E-64
DNAJB13	ENSG00000	0.26	25.849	-4.413	1.40E-62
DNAJB3	ENSG00000	0.02	48.15	-5.591	3.73E-80
DNAJB7	ENSG00000	0.13	12.88	-3.619	7.22E-68
DNAJB8	ENSG00000	0	65.89	-6.064	3.04E-71
DNAJB8-A	ENSG00000	0	4.52	-2.465	1.46E-59
DNAJC12	ENSG00000	0.65	8.12	-2.467	4.33E-45
DNAJC18	ENSG00000	6.9	220.341	-4.808	7.28E-69
DNAJC24	ENSG00000	3.72	19.18	-2.096	3.01E-53
DNAJC27	ENSG00000	2.29	15.29	-2.308	1.54E-70
DNAJC27-	ENSG00000	0.77	20.141	-3.578	1.11E-77
DNAJC4	ENSG00000	35.631	407.483	-3.479	1.42E-65
DNAJC5B	ENSG00000	1.16	84.958	-5.315	4.94E-46
DNAJC5G	ENSG00000	0.18	34.071	-4.893	9.59E-137
DNAL1	ENSG00000	5.21	59.1	-3.275	5.02E-59
DNAL4	ENSG00000	16.54	87.851	-2.341	6.93E-54
DNALI1	ENSG00000	5.32	169.447	-4.753	1.67E-62
DND1P1	ENSG00000	0.44	5.3	-2.129	8.52E-30
DNHD1	ENSG00000	3.21	78.422	-4.238	2.83E-95
DNM1P41	ENSG00000	0.15	3.8	-2.061	1.59E-84
DNM1P46	ENSG00000	0.23	18.9	-4.016	1.05E-81
DOC2A	ENSG00000	3.9	83.899	-4.115	1.59E-54
DOCK3	ENSG00000	0.84	7.72	-2.245	2.78E-60
DOCK4-AS	ENSG00000	0.01	3.38	-2.117	4.80E-43
DOCK9-AS	ENSG00000	0.63	15.23	-3.316	5.52E-85
DOK6	ENSG00000	0.1	5.31	-2.52	2.02E-45
DOK7	ENSG00000	0.45	8.78	-2.754	6.01E-65
DOT1L	ENSG00000	18.06	139.072	-2.878	1.57E-54
DPCD	ENSG00000	22.529	181.144	-2.953	6.08E-49
DPEP1	ENSG00000	3.46	78.612	-4.158	3.25E-54
DPEP3	ENSG00000	3.28	61.789	-3.875	1.42E-45
DPF3	ENSG00000	0.53	19.92	-3.773	1.80E-73
DPP10-AS	ENSG00000	2.5	34.059	-3.324	1.76E-42
DPP10-AS	ENSG00000	0	4.93	-2.568	8.86E-46
DPP7	ENSG00000	51.911	259.573	-2.3	5.42E-48
DPY19L1	ENSG00000	8.07	44.28	-2.32	4.84E-50
DPY19L1P1	ENSG00000	0.43	5.04	-2.079	2.08E-94
DPY19L2	ENSG00000	2.7	47.422	-3.71	1.45E-80
DPY19L2P1	ENSG00000	0.75	12.61	-2.959	4.53E-66
DPY19L2P2	ENSG00000	1.61	68.96	-4.744	2.16E-71
DPY19L2P3	ENSG00000	0.57	5.64	-2.08	2.15E-73
DPY19L2P4	ENSG00000	0.08	13.97	-3.793	2.71E-75
DPYSL4	ENSG00000	1.69	14.71	-2.546	4.04E-27
DRC1	ENSG00000	0.23	37.731	-4.977	1.47E-61
DRC7	ENSG00000	0.14	52.691	-5.558	1.19E-72
DRICH1	ENSG00000	0.39	59.9	-5.453	1.90E-75

DSCR9	ENSG00000	0.09	14.72	-3.85	3.06E-71
DSG1-AS1	ENSG00000	0.09	10.41	-3.388	3.47E-49
DTNA	ENSG00000	1	10.24	-2.491	1.85E-31
DUSP1	ENSG00000	12.72	68.888	-2.349	5.98E-29
DUSP13	ENSG00000	0.3	68.669	-5.744	2.13E-74
DUSP15	ENSG00000	1.51	309.865	-6.952	9.14E-89
DUSP18	ENSG00000	3.42	35.879	-3.061	1.43E-47
DUSP21	ENSG00000	0.03	11.79	-3.634	5.01E-66
DUSP26	ENSG00000	0.38	4.79	-2.069	6.98E-28
DUSP28	ENSG00000	3.1	20.44	-2.387	5.16E-76
DUXAP8	ENSG00000	1.42	12	-2.425	1.53E-33
DXO	ENSG00000	12.12	92.539	-2.834	5.74E-85
DYDC1	ENSG00000	0.15	151.775	-7.054	3.30E-80
DYDC2	ENSG00000	0.49	13.03	-3.235	1.45E-43
DYNC2H1	ENSG00000	4.23	27.1	-2.426	3.67E-75
DYNC2LI1	ENSG00000	10.73	47.212	-2.039	2.47E-70
DYNLRB2	ENSG00000	0.76	321.482	-7.517	1.45E-83
DYRK1B	ENSG00000	11.22	115.703	-3.256	5.55E-65
DYRK3	ENSG00000	1.08	44.27	-4.444	7.85E-72
DZIP1	ENSG00000	3.67	77.471	-4.071	1.57E-64
EAF1-AS1	ENSG00000	0.14	6.94	-2.8	1.16E-77
EBLN1	ENSG00000	0.02	3.59	-2.17	2.26E-60
ECHDC2	ENSG00000	31.349	226.096	-2.812	5.50E-77
EDRF1-AS1	ENSG00000	0.36	26.44	-4.335	3.20E-79
EEF1DP2	ENSG00000	0	4.07	-2.342	1.76E-25
EFCAB1	ENSG00000	0.15	47.261	-5.391	6.31E-64
EFCAB10	ENSG00000	2.34	25.74	-3.001	2.88E-44
EFCAB11	ENSG00000	9.1	71.928	-2.852	6.28E-43
EFCAB12	ENSG00000	0.26	12.56	-3.428	6.38E-70
EFCAB13	ENSG00000	1.28	27.441	-3.641	1.14E-99
EFCAB14-A	ENSG00000	0	5.41	-2.68	7.39E-57
EFCAB2	ENSG00000	4.8	32.11	-2.513	3.72E-49
EFCAB3	ENSG00000	0.01	4.65	-2.484	8.84E-51
EFCAB5	ENSG00000	0.08	19.779	-4.266	3.80E-70
EFCAB6	ENSG00000	0.27	71.268	-5.83	1.93E-86
EFCAB8	ENSG00000	0.12	6.27	-2.698	9.45E-58
EFCAB9	ENSG00000	0	12.45	-3.75	9.45E-61
EFEMP2	ENSG00000	13.92	65.562	-2.157	1.18E-15
EFHB	ENSG00000	0.84	17.93	-3.363	3.05E-67
EFHC1	ENSG00000	4.92	36.811	-2.675	7.81E-60
EFHC2	ENSG00000	0.32	9.02	-2.924	3.22E-79
EFTUD1P1	ENSG00000	0.18	3.82	-2.03	4.82E-63
EGFEM1P	ENSG00000	0.14	3.73	-2.053	5.53E-46
EGFLAM-A	ENSG00000	0	8.27	-3.213	2.84E-57
EGLN2	ENSG00000	73.272	332.151	-2.165	8.18E-64
EGR4	ENSG00000	0.06	5.13	-2.532	6.50E-76
EHD1	ENSG00000	21.27	315.609	-3.83	1.12E-60
EID3	ENSG00000	4.61	70.589	-3.674	3.21E-66
EIF3J-AS1	ENSG00000	2.11	11.53	-2.01	3.75E-52

EIF4BP8	ENSG00000	0	4.31	-2.409	6.87E-60
EIF4G3	ENSG00000	18.04	85.65	-2.186	2.39E-51
EIF5A2	ENSG00000	2.52	46.189	-3.745	1.33E-60
EIF5AL1	ENSG00000	0.94	7.71	-2.167	9.72E-36
ELAVL4	ENSG00000	1.02	12.51	-2.742	8.28E-33
ELK2AP	ENSG00000	0.02	8.4	-3.204	7.53E-70
ELK2BP	ENSG00000	0	3.62	-2.208	1.51E-65
ELL	ENSG00000	10.89	53.66	-2.201	1.06E-38
ELL3	ENSG00000	1.41	33.42	-3.836	9.39E-77
ELOVL2	ENSG00000	1.14	7.9	-2.056	4.92E-35
ELOVL2-AS	ENSG00000	0.07	11.14	-3.504	3.04E-86
ELP5	ENSG00000	37.4	212.172	-2.473	5.03E-53
EME2	ENSG00000	2.6	18.93	-2.469	9.31E-92
EMX1	ENSG00000	0.47	6.56	-2.363	4.91E-14
ENKD1	ENSG00000	11.39	77	-2.654	5.48E-76
ENKUR	ENSG00000	0.28	7.7	-2.765	2.56E-45
ENO1-AS1	ENSG00000	0.38	11.18	-3.142	1.40E-64
ENOX1	ENSG00000	0.55	12.26	-3.097	1.69E-62
ENPP5	ENSG00000	2.34	24.281	-2.92	4.28E-65
STPG1	ENSG00000	5.23	40.221	-2.726	1.02E-61
HECW1	ENSG00000	0.31	7.69	-2.73	2.12E-45
ICA1	ENSG00000	6.85	81.12	-3.387	4.50E-54
NDUFAF7	ENSG00000	20.4	94.352	-2.156	3.30E-36
HSPB6	ENSG00000	6.33	66.38	-3.2	4.35E-20
PDK4	ENSG00000	2.47	14.54	-2.163	6.95E-17
SLC22A16	ENSG00000	0.13	26.6	-4.61	6.24E-74
ZMYND10	ENSG00000	0.75	575.39	-8.364	2.49E-76
SKAP2	ENSG00000	4.72	23.14	-2.077	2.90E-33
SPPL2B	ENSG00000	22.45	133.37	-2.519	2.45E-80
YBX2	ENSG00000	5.25	930.91	-7.22	3.78E-73
KRT33A	ENSG00000	0	3.08	-2.029	7.02E-50
SPATA20	ENSG00000	14.14	213.633	-3.825	2.02E-74
FARP2	ENSG00000	8.67	51.56	-2.442	3.12E-61
SCIN	ENSG00000	1.06	10.5	-2.481	1.95E-30
PRSS21	ENSG00000	3.71	136.371	-4.866	9.51E-66
MARK4	ENSG00000	11.82	53.958	-2.1	2.50E-61
PAFAH1B1	ENSG00000	49.459	270.483	-2.428	8.11E-53
ST7L	ENSG00000	10.22	44.68	-2.025	1.43E-66
TKTL1	ENSG00000	2.8	129.84	-5.106	3.75E-21
PIGQ	ENSG00000	14.07	82.773	-2.475	2.31E-68
NFIX	ENSG00000	3.03	15.28	-2.014	1.16E-12
PKD1	ENSG00000	20.43	104.589	-2.301	8.52E-55
MAPK8IP2	ENSG00000	2.27	13.72	-2.17	5.14E-45
POMT2	ENSG00000	21.84	118.882	-2.392	3.50E-69
MLXIPL	ENSG00000	1.15	57.972	-4.778	5.36E-81
HHATL	ENSG00000	0.05	10.76	-3.485	4.12E-91
PHF7	ENSG00000	7.74	1705.406	-7.609	2.76E-84
FUZ	ENSG00000	13.66	132.66	-3.189	3.02E-68
SLC6A13	ENSG00000	0.2	5.93	-2.53	2.52E-37

PRSS3	ENSG00000	0.12	8.68	-3.112	6.48E-33
SCMH1	ENSG00000	9.17	51.421	-2.366	1.84E-60
ZBTB32	ENSG00000	0.93	93.002	-5.606	1.78E-77
SEMA3B	ENSG00000	2.1	26.451	-3.146	5.26E-36
UBR7	ENSG00000	14.05	63.412	-2.098	4.96E-39
MAMLD1	ENSG00000	0.74	28.391	-4.078	1.06E-86
MTMR11	ENSG00000	3.3	23.94	-2.536	5.74E-39
RGPD5	ENSG00000	3.54	22.841	-2.393	1.07E-33
RUFY3	ENSG00000	14.35	72.629	-2.262	1.95E-70
SAMD4A	ENSG00000	2.34	250.557	-6.235	1.45E-69
PLEKHB1	ENSG00000	3.61	47.339	-3.39	2.23E-49
NRXN3	ENSG00000	1.56	10.21	-2.131	2.93E-29
FHL1	ENSG00000	7.48	80.912	-3.272	1.02E-35
GRAMD1B	ENSG00000	1.53	18.95	-2.979	8.29E-68
GLRX2	ENSG00000	4.75	65.739	-3.537	1.54E-59
TOMM34	ENSG00000	31.701	154.161	-2.246	5.17E-56
TBPL1	ENSG00000	31.53	159.001	-2.298	4.26E-52
IFT88	ENSG00000	4.78	68.54	-3.589	4.53E-77
LRRC7	ENSG00000	0.28	5.05	-2.241	3.63E-51
MYOM2	ENSG00000	5.05	35.06	-2.575	3.16E-19
USP2	ENSG00000	0.89	115.703	-5.948	3.71E-91
RAI14	ENSG00000	8.2	42.109	-2.228	3.39E-35
SOX30	ENSG00000	0.26	107.083	-6.423	4.89E-83
RAB27B	ENSG00000	0.08	4.18	-2.262	2.37E-38
FAM65C	ENSG00000	1.22	17.65	-3.071	3.27E-68
SPATA7	ENSG00000	7.74	68.199	-2.985	1.41E-56
ZPBP	ENSG00000	0.1	136.153	-6.962	6.88E-93
TG	ENSG00000	0.5	6.76	-2.371	1.63E-51
ZIC2	ENSG00000	0.65	6.27	-2.14	7.21E-08
MAGEC2	ENSG00000	1.6	22.86	-3.198	9.14E-22
WDR37	ENSG00000	3.97	26.591	-2.473	8.60E-62
SCML1	ENSG00000	1.91	39.159	-3.787	8.26E-89
GUCA1A	ENSG00000	5.4	36.021	-2.532	1.85E-25
EPN3	ENSG00000	0.21	4.1	-2.076	4.23E-33
LAMC3	ENSG00000	10.42	55.211	-2.299	5.82E-26
FAM160A2	ENSG00000	18.491	91.62	-2.249	1.09E-85
MCF2L2	ENSG00000	1.13	8.31	-2.128	2.70E-28
ENTPD2	ENSG00000	0.87	7.83	-2.239	3.27E-29
SPO11	ENSG00000	0	6.91	-2.984	1.78E-86
KCNH2	ENSG00000	8.63	52.76	-2.481	1.31E-45
ITIH4	ENSG00000	3.76	28.769	-2.645	2.51E-39
PHF21B	ENSG00000	1.13	8.47	-2.152	2.33E-53
PIGV	ENSG00000	6.54	46.71	-2.662	3.34E-68
SPAG4	ENSG00000	5.67	59.42	-3.179	2.18E-64
SLC6A16	ENSG00000	1.29	92.378	-5.35	6.57E-87
SPHK2	ENSG00000	4.92	35.809	-2.636	8.56E-51
HAGH	ENSG00000	26.37	148.713	-2.452	1.09E-72
LIMCH1	ENSG00000	1.09	12.48	-2.689	6.93E-36
SPA17	ENSG00000	5.99	296.07	-5.409	3.08E-64

WISP2	ENSG00000	0.5	37.629	-4.687	4.52E-35
SUGP2	ENSG00000	35.15	187.103	-2.379	4.57E-94
SLC12A2	ENSG00000	2.08	13.58	-2.243	7.97E-39
EYA2	ENSG00000	0.86	7.07	-2.117	4.83E-25
SNCAIP	ENSG00000	0.86	9.74	-2.53	9.03E-26
PMS1	ENSG00000	11.22	62.141	-2.369	5.59E-53
IPO5	ENSG00000	54.681	223.572	-2.012	5.49E-43
ROPN1	ENSG00000	0.06	197.416	-7.548	2.27E-83
SNAP91	ENSG00000	0.56	12.31	-3.093	2.48E-54
SEC61A2	ENSG00000	6.07	31.41	-2.197	3.23E-64
TLE2	ENSG00000	7.06	43.209	-2.455	1.80E-26
PDE4A	ENSG00000	5.76	36.501	-2.472	1.01E-35
ZMYND12	ENSG00000	0.96	34.589	-4.183	6.54E-69
STAG3	ENSG00000	12.53	182.353	-3.76	3.04E-74
PFKP	ENSG00000	37.77	188.196	-2.287	3.23E-22
PRKCZ	ENSG00000	15.53	120.684	-2.88	2.84E-47
HDAC4	ENSG00000	3.25	27.401	-2.74	5.84E-45
REEP1	ENSG00000	3.89	48.741	-3.347	1.61E-61
TTC7A	ENSG00000	11.89	121.582	-3.249	3.14E-73
SIRT2	ENSG00000	16.03	95.749	-2.506	4.61E-74
PYGM	ENSG00000	0.9	11.99	-2.773	5.28E-63
PAGE1	ENSG00000	0	21.69	-4.504	2.31E-91
MAST4	ENSG00000	1.92	19.401	-2.805	2.38E-61
SDK2	ENSG00000	2.07	16.05	-2.473	1.84E-41
FSTL3	ENSG00000	7.61	94.202	-3.467	3.43E-73
FRMPD1	ENSG00000	0.46	11.16	-3.058	3.70E-71
ST6GALNA	ENSG00000	2.01	146.615	-5.616	8.85E-68
PTPN21	ENSG00000	4.05	32.869	-2.746	4.88E-68
RHOBTB1	ENSG00000	1.27	34.561	-3.969	3.74E-62
MOV10L1	ENSG00000	0.63	59.73	-5.219	2.05E-93
FNDC8	ENSG00000	0.1	115.559	-6.727	2.57E-68
PPP2R2C	ENSG00000	1.26	9.11	-2.161	1.63E-38
TUBE1	ENSG00000	6.63	30.271	-2.035	8.16E-61
KCNQ2	ENSG00000	4.11	26.119	-2.408	1.05E-26
VPS9D1	ENSG00000	4.89	26.779	-2.238	2.24E-54
WDR62	ENSG00000	18.17	98.489	-2.376	4.23E-26
SEC31B	ENSG00000	2.49	32.869	-3.279	2.76E-109
TUBA3D	ENSG00000	3.56	293.597	-6.014	4.50E-50
MKRN2	ENSG00000	16.12	71.5	-2.082	4.20E-56
MBNL3	ENSG00000	1.77	23.88	-3.167	5.78E-58
SPAG6	ENSG00000	0.74	76.5	-5.477	1.32E-73
LRCH4	ENSG00000	33.639	211.497	-2.617	2.23E-107
FAM76B	ENSG00000	8.07	61.72	-2.79	1.10E-66
NAALAD2	ENSG00000	2.45	21.61	-2.712	5.08E-44
FKBP6	ENSG00000	0.48	81.21	-5.796	1.07E-77
SMC1B	ENSG00000	0.16	30.391	-4.758	3.01E-80
MAP2	ENSG00000	0.98	8.33	-2.236	3.50E-29
PIAS2	ENSG00000	8.32	116.033	-3.65	3.30E-61
LAMP3	ENSG00000	3.5	18.77	-2.135	4.78E-18

NEBL	ENSG0000(	1.33	28.499	-3.662	6.15E-37
ZCWPW1	ENSG0000(	6.96	115.88	-3.876	7.98E-73
PKD2L2	ENSG0000(	0.08	8.49	-3.135	4.53E-80
TP53INP2	ENSG0000(	9.28	50.348	-2.32	5.48E-54
MYH7B	ENSG0000(	0.89	30.53	-4.06	1.12E-60
TOLLIP	ENSG0000(	16.27	102.828	-2.588	1.31E-84
TNS1	ENSG0000(	6.31	30.22	-2.095	1.36E-17
LIPE	ENSG0000(	5.04	96.85	-4.018	5.58E-65
LRRC16A	ENSG0000(	4.32	22.54	-2.146	1.45E-54
EPHA6	ENSG0000(	0.27	24.09	-4.304	6.73E-62
RFX3	ENSG0000(	1.21	23.86	-3.492	2.39E-58
RDH8	ENSG0000(	0.02	4.46	-2.42	3.09E-43
PIH1D3	ENSG0000(	0	13.77	-3.885	1.01E-71
MOK	ENSG0000(	6.24	112.477	-3.97	8.38E-70
SMARCD3	ENSG0000(	8.29	59.309	-2.699	1.01E-34
PGR	ENSG0000(	0.06	3.62	-2.124	2.01E-74
EPB41L3	ENSG0000(	5.66	100.872	-3.935	4.00E-79
TRPM3	ENSG0000(	0.1	6.05	-2.68	2.57E-35
ULK2	ENSG0000(	6.02	37.801	-2.467	6.01E-55
SLC27A5	ENSG0000(	7.53	56.172	-2.745	5.32E-59
GCKR	ENSG0000(	0.32	8.64	-2.869	2.51E-80
MAPRE3	ENSG0000(	6.77	95.18	-3.63	2.61E-90
OVGP1	ENSG0000(	1.42	13.03	-2.535	8.37E-72
ZNF213	ENSG0000(	5.17	38.91	-2.693	7.72E-67
TTC39A	ENSG0000(	5.35	77.788	-3.633	1.48E-55
USP40	ENSG0000(	8.07	35.461	-2.007	6.32E-70
NME8	ENSG0000(	0.68	15.74	-3.317	1.00E-38
EPDR1	ENSG0000(	1.99	11.15	-2.023	2.61E-33
FAT2	ENSG0000(	0.09	16.14	-3.975	2.12E-54
ERO1LB	ENSG0000(	2.51	16.53	-2.32	3.41E-61
PPEF1	ENSG0000(	0.52	17.59	-3.612	2.21E-68
MT3	ENSG0000(	0.54	8.49	-2.623	9.09E-31
GNAO1	ENSG0000(	3.49	24.65	-2.514	1.83E-53
RFX2	ENSG0000(	5.19	88.431	-3.853	3.58E-68
GP6	ENSG0000(	0.15	3.78	-2.055	9.36E-54
GNA11	ENSG0000(	15.35	77.551	-2.264	1.42E-67
KIF9	ENSG0000(	16.26	90.327	-2.404	4.55E-33
PPP1R13B	ENSG0000(	10.61	70.83	-2.629	8.86E-56
TESC	ENSG0000(	5.53	50.209	-2.971	7.41E-49
SIRT4	ENSG0000(	1.22	9.47	-2.238	1.33E-51
OTUB2	ENSG0000(	0.74	17.64	-3.421	2.29E-51
THPO	ENSG0000(	0.29	4.71	-2.146	8.69E-29
TF	ENSG0000(	5.1	35.451	-2.579	5.01E-17
MYO15A	ENSG0000(	1.04	31.491	-3.993	7.77E-88
ZFHX4	ENSG0000(	0.15	5.36	-2.467	8.60E-30
RPGRIP1	ENSG0000(	1.96	53.548	-4.204	1.48E-51
TEKT2	ENSG0000(	0.65	97.531	-5.9	2.51E-58
XXbac-B46	ENSG0000(	3.6	19.38	-2.147	1.12E-51
LRRFIP2	ENSG0000(	18.69	94.208	-2.274	1.30E-69

NXPE1	ENSG00000	0	4.66	-2.501	2.55E-45
TDRD1	ENSG00000	0.54	7.57	-2.476	4.78E-66
MYO3A	ENSG00000	0.97	7.74	-2.149	1.67E-41
PALMD	ENSG00000	2.28	12.26	-2.015	4.67E-34
IZUMO4	ENSG00000	1.42	723.374	-8.226	2.00E-100
P2RX6	ENSG00000	0.5	11.02	-3.002	3.89E-96
SLC7A4	ENSG00000	2.97	19.48	-2.367	7.94E-32
UPB1	ENSG00000	0.23	5.42	-2.384	2.20E-98
YPEL1	ENSG00000	2.01	105.682	-5.147	2.24E-66
SLC35E4	ENSG00000	2.5	28.46	-3.073	2.25E-55
GGA1	ENSG00000	35.37	281.794	-2.959	1.67E-92
GGTLC2	ENSG00000	0	8.19	-3.2	1.34E-35
SOX10	ENSG00000	0.15	3.78	-2.055	4.49E-39
RSPH14	ENSG00000	0.83	32.619	-4.199	6.62E-77
PPP6R2	ENSG00000	23.76	114.332	-2.22	1.64E-86
SBF1	ENSG00000	33.76	182.746	-2.402	9.70E-75
TTLL1	ENSG00000	3.88	19.91	-2.099	1.46E-48
KCTD17	ENSG00000	13.27	77.799	-2.465	5.79E-61
LRRC74A	ENSG00000	0.05	21.4	-4.415	3.64E-72
SAMD15	ENSG00000	0.57	18.76	-3.654	1.29E-67
PPM1A	ENSG00000	21.73	95.239	-2.082	3.04E-49
ZC3H14	ENSG00000	44.87	223.308	-2.29	3.43E-44
NFATC4	ENSG00000	23.95	115.679	-2.225	7.44E-40
NDRG3	ENSG00000	15.27	89.877	-2.482	3.73E-57
MRGBP	ENSG00000	11.86	64.128	-2.34	1.41E-63
TCFL5	ENSG00000	9.35	66.851	-2.713	1.71E-58
SPEF1	ENSG00000	0.3	21.711	-4.127	9.17E-64
SEL1L2	ENSG00000	0.07	12.4	-3.647	3.63E-70
SLC52A3	ENSG00000	4.85	52.559	-3.195	7.52E-62
PDYN	ENSG00000	0.01	4.88	-2.541	5.17E-44
MROH8	ENSG00000	3.32	36.041	-3.1	1.52E-64
EPPIN	ENSG00000	0.17	102.167	-6.462	2.83E-90
MAP1LC3A	ENSG00000	17.14	97.578	-2.442	3.96E-75
ZNF516	ENSG00000	7.04	48.341	-2.617	1.07E-73
MYOM1	ENSG00000	0.99	10.63	-2.547	2.98E-66
LIPG	ENSG00000	1.24	9.68	-2.253	8.53E-19
NOL4	ENSG00000	0.03	26.25	-4.726	3.41E-66
POLI	ENSG00000	7.21	54.041	-2.745	3.30E-94
VSIG1	ENSG00000	0.28	34.391	-4.789	6.79E-89
RHOXF1	ENSG00000	0.91	19.66	-3.435	1.60E-64
PAGE4	ENSG00000	0.1	6.61	-2.79	2.21E-30
MCF2	ENSG00000	0.13	18.9	-4.138	2.43E-128
SMARCA1	ENSG00000	4.52	51.581	-3.252	4.62E-31
PPP1R2P9	ENSG00000	1.36	100.148	-5.422	4.19E-68
PCYT1B	ENSG00000	1.34	16.99	-2.943	1.38E-51
TAF7L	ENSG00000	0.25	39.7	-5.025	1.09E-121
KIAA0226L	ENSG00000	2.89	27.149	-2.855	8.85E-66
KLF5	ENSG00000	8.18	36.061	-2.013	1.53E-19
UGGT2	ENSG00000	3.89	20.79	-2.156	6.25E-57

FLT1	ENSG0000(	2.55	19.03	-2.496	1.30E-37
KATNAL1	ENSG0000(	2.97	20.72	-2.452	3.43E-54
INTS6	ENSG0000(	7.14	53.541	-2.744	6.70E-84
MEDAG	ENSG0000(	0.54	6.04	-2.193	1.90E-46
HSF4	ENSG0000(	3.69	36.87	-3.013	2.79E-51
TSNAXIP1	ENSG0000(	1.59	57.328	-4.493	1.66E-84
PARD6A	ENSG0000(	1.9	54.518	-4.259	1.50E-66
ZNF821	ENSG0000(	5.79	44.721	-2.751	1.91E-56
PRSS54	ENSG0000(	0	55.41	-5.818	2.26E-70
NDRG4	ENSG0000(	6.11	109.599	-3.959	1.45E-74
SMPD3	ENSG0000(	1.36	12.17	-2.48	1.01E-74
NECAB2	ENSG0000(	0.72	13.43	-3.069	4.72E-63
WFDC1	ENSG0000(	1.02	15.5	-3.03	1.02E-32
FAM173A	ENSG0000(	15.76	92.219	-2.476	1.31E-48
RHBDL1	ENSG0000(	0.82	7.84	-2.28	2.37E-73
TMC5	ENSG0000(	0.88	10.34	-2.593	1.29E-36
SLC6A2	ENSG0000(	0.11	5.54	-2.559	1.15E-65
IQCH	ENSG0000(	1.39	11.54	-2.391	3.49E-49
SLC30A4	ENSG0000(	1.01	8.9	-2.3	2.17E-68
MYEF2	ENSG0000(	4.59	61.991	-3.494	2.63E-66
IL7	ENSG0000(	0.32	5.97	-2.401	7.37E-55
TRPS1	ENSG0000(	1.51	10.95	-2.251	7.63E-31
SPAG1	ENSG0000(	2.19	12.28	-2.058	3.45E-31
GML	ENSG0000(	0	3.35	-2.121	1.52E-67
NEFM	ENSG0000(	0.42	23.36	-4.101	3.02E-37
FGL1	ENSG0000(	0.28	6.24	-2.5	1.50E-39
TULP2	ENSG0000(	1.13	50.331	-4.591	9.60E-56
NFKBIB	ENSG0000(	25.81	107.813	-2.021	1.87E-36
LHB	ENSG0000(	0.52	6.26	-2.256	3.47E-52
PEX11G	ENSG0000(	2.3	38.561	-3.584	3.47E-79
LYL1	ENSG0000(	4.05	27.83	-2.513	3.22E-42
RSPH6A	ENSG0000(	0.01	40.26	-5.352	1.17E-71
VRK3	ENSG0000(	26.77	218.183	-2.981	2.19E-59
MED26	ENSG0000(	8.63	48.12	-2.351	2.78E-59
SLC1A6	ENSG0000(	1.37	38.401	-4.055	6.91E-73
OVOL3	ENSG0000(	0.13	4.77	-2.352	4.50E-69
ZFR2	ENSG0000(	6.46	31.219	-2.111	1.55E-29
TJP3	ENSG0000(	14.91	102.707	-2.705	8.70E-20
FZR1	ENSG0000(	21.08	93.131	-2.092	2.07E-66
ICAM5	ENSG0000(	0.25	6.16	-2.518	4.47E-70
ZNRF4	ENSG0000(	0	160.574	-7.336	1.34E-75
SYNGR4	ENSG0000(	0.24	69.329	-5.826	1.21E-88
THEG	ENSG0000(	0.21	57.209	-5.588	3.30E-63
MAST1	ENSG0000(	2.19	13.97	-2.23	5.39E-34
PDE4C	ENSG0000(	1.55	9.54	-2.047	1.62E-43
ISYNA1	ENSG0000(	76.802	497.24	-2.679	1.69E-65
ETV2	ENSG0000(	1	24.33	-3.663	2.50E-60
GAPDHS	ENSG0000(	0.08	387.646	-8.491	1.23E-76
GRIK5	ENSG0000(	1.26	66.768	-4.906	4.46E-77

RNF32	ENSG00000	2.42	87.25	-4.69	3.30E-74
IQCE	ENSG00000	6.97	69.69	-3.149	1.15E-54
SPAM1	ENSG00000	0.1	12.71	-3.64	2.01E-50
FSCN3	ENSG00000	0.15	130.471	-6.837	2.11E-73
MOSPD3	ENSG00000	17.701	88.199	-2.254	8.65E-49
FBXO24	ENSG00000	0.88	120.333	-6.012	7.55E-77
PPP1R17	ENSG00000	0.04	3.44	-2.094	4.88E-28
TBL2	ENSG00000	28.999	259.285	-3.117	2.92E-57
GALNTL5	ENSG00000	0.02	40.19	-5.336	1.83E-66
SPATA6L	ENSG00000	0.42	24.931	-4.191	1.09E-73
OGN	ENSG00000	0.53	5.97	-2.188	4.21E-04
TLE4	ENSG00000	8.67	147.994	-3.946	4.00E-69
TESK1	ENSG00000	23.65	108.337	-2.149	9.83E-66
SH3GL2	ENSG00000	0.7	6.97	-2.229	1.63E-76
PTGDS	ENSG00000	97.032	4550.154	-5.537	3.13E-42
SFXN3	ENSG00000	6.57	30.13	-2.04	2.73E-31
MTPAP	ENSG00000	9.06	83.458	-3.07	1.55E-48
KRT23	ENSG00000	0.37	30.721	-4.533	2.92E-43
GIT1	ENSG00000	30.11	131.461	-2.09	1.25E-57
RUNDC3A	ENSG00000	1.89	38.929	-3.788	2.84E-56
SGCA	ENSG00000	0.53	34.09	-4.519	1.07E-64
RND2	ENSG00000	10.57	49.261	-2.119	2.00E-33
MYH3	ENSG00000	2.16	51.189	-4.046	4.74E-56
KIAA1211	ENSG00000	0.97	35.5	-4.212	1.10E-55
MAPK10	ENSG00000	0.96	39.229	-4.359	4.01E-66
INPP4B	ENSG00000	0.85	42.039	-4.54	2.33E-67
SOD3	ENSG00000	7.13	99.339	-3.625	8.97E-52
HGFAC	ENSG00000	0.09	18.93	-4.193	4.28E-83
LRP2BP	ENSG00000	0.64	11.05	-2.877	1.63E-70
PPARGC1A	ENSG00000	0.18	9.45	-3.147	4.63E-61
ZBTB16	ENSG00000	0.39	10.5	-3.049	7.53E-70
P2RX3	ENSG00000	0.05	10.3	-3.428	4.11E-53
RNF141	ENSG00000	9.44	118.094	-3.512	6.34E-65
GALNT18	ENSG00000	2.12	18.83	-2.668	4.19E-47
MADD	ENSG00000	14.66	64.248	-2.059	3.91E-71
PTPMT1	ENSG00000	25.52	140.808	-2.419	1.85E-49
SLC22A18	ENSG00000	7.19	50.839	-2.662	2.34E-55
SOX6	ENSG00000	0.16	20.97	-4.243	1.98E-74
NUP98	ENSG00000	38.95	170.295	-2.1	1.25E-51
PRPF40B	ENSG00000	13.72	62.15	-2.101	3.37E-67
IL23A	ENSG00000	3	23.03	-2.587	6.10E-26
LIN7A	ENSG00000	0.23	25.06	-4.405	1.46E-65
TNS2	ENSG00000	9.57	56.16	-2.435	1.21E-21
GSG1	ENSG00000	0.33	544.352	-8.68	6.35E-61
HCFC2	ENSG00000	2.86	41.469	-3.46	5.58E-82
RFX4	ENSG00000	0.05	68.341	-6.045	2.94E-76
MAK	ENSG00000	0.15	8.22	-3.003	7.65E-61
OPRM1	ENSG00000	0	5.55	-2.711	1.82E-57
SLC26A8	ENSG00000	0.12	44.12	-5.332	1.27E-79

SAYSD1	ENSG00000	16.05	78.042	-2.213	2.56E-51
ZNF451	ENSG00000	11.71	51.25	-2.039	2.07E-45
FHL5	ENSG00000	0.3	80.147	-5.964	4.82E-106
HDGFL1	ENSG00000	18.03	116.726	-2.629	4.02E-21
WASF1	ENSG00000	8.72	78.367	-3.03	1.22E-53
PHF1	ENSG00000	43.32	308.023	-2.802	1.91E-95
PACRG	ENSG00000	0.39	40.689	-4.907	2.51E-75
PDE10A	ENSG00000	0.26	4.35	-2.086	4.49E-48
SMOC2	ENSG00000	5.61	49.621	-2.937	2.18E-27
FAM120B	ENSG00000	5.85	28.829	-2.123	1.27E-55
WISP3	ENSG00000	0.16	6.01	-2.595	7.80E-61
PCDHB2	ENSG00000	0.78	7.09	-2.184	9.15E-29
NME5	ENSG00000	1.07	63.03	-4.951	4.71E-77
PCDHB5	ENSG00000	0.26	4.08	-2.011	1.40E-16
PCDHB15	ENSG00000	0.74	6.49	-2.106	8.24E-59
SLC27A6	ENSG00000	0.33	7.39	-2.657	4.17E-39
SLC12A7	ENSG00000	7.71	46	-2.432	7.84E-50
IL5	ENSG00000	0.03	5.04	-2.552	2.22E-71
PFKFB4	ENSG00000	8.13	37.46	-2.075	2.21E-51
PRKAR2A	ENSG00000	8.83	105.492	-3.437	1.66E-54
HES1	ENSG00000	3.44	17.45	-2.055	8.87E-09
IFT57	ENSG00000	16.41	167.834	-3.278	4.18E-54
IQCG	ENSG00000	6.27	84.488	-3.556	1.83E-87
MORC1	ENSG00000	0.09	8.1	-3.062	1.82E-78
ROPN1B	ENSG00000	0.22	295.906	-7.927	4.52E-84
KIAA1257	ENSG00000	1.56	28.41	-3.522	1.43E-56
NEK11	ENSG00000	1.44	24.63	-3.393	1.16E-78
HEMK1	ENSG00000	8.06	38.249	-2.115	1.34E-58
GORASP1	ENSG00000	14.34	64.789	-2.101	1.03E-66
PEX5L	ENSG00000	0.08	3.36	-2.013	6.10E-73
PLCH1	ENSG00000	0.4	13.33	-3.356	2.35E-54
ZBTB47	ENSG00000	1.57	11.5	-2.282	8.72E-30
TNNC1	ENSG00000	1.14	13.66	-2.776	4.59E-15
IL1A	ENSG00000	0.14	8.86	-3.113	7.82E-85
KCNIP3	ENSG00000	1.45	15.12	-2.718	8.89E-55
TP53I3	ENSG00000	7.87	53.039	-2.607	1.38E-29
SLC30A3	ENSG00000	1.19	60.241	-4.805	1.08E-119
ITGB6	ENSG00000	1.14	77.541	-5.198	4.27E-62
PDE1A	ENSG00000	1.12	27.68	-3.758	2.56E-51
REEP6	ENSG00000	36.579	394.997	-3.397	9.00E-68
PCSK4	ENSG00000	4.02	198.156	-5.31	8.01E-84
GRB14	ENSG00000	2.18	44.289	-3.832	1.92E-72
GALNT3	ENSG00000	1.94	14.73	-2.42	2.96E-29
SLC9A2	ENSG00000	0.04	4.77	-2.472	1.03E-71
RMDN2	ENSG00000	2.64	26.31	-2.907	2.61E-64
PLEKHA3	ENSG00000	6.09	30.64	-2.158	4.21E-51
PARD3B	ENSG00000	0.74	6.37	-2.083	4.45E-43
PRRX1	ENSG00000	3.74	40.43	-3.128	5.22E-35
MORN1	ENSG00000	2.38	23.19	-2.839	4.15E-77

RALGPS2	ENSG0000	6.59	45.339	-2.61	2.45E-45
KIAA1324	ENSG0000	1.47	31.6	-3.722	3.67E-53
SWT1	ENSG0000	3.12	22.32	-2.501	4.14E-64
TNNI3K	ENSG0000	0.29	6.41	-2.522	2.98E-74
KIF21B	ENSG0000	3.54	17.179	-2.002	2.27E-23
OSCP1	ENSG0000	6.03	105.499	-3.921	6.29E-71
HPCAL4	ENSG0000	0.09	5.13	-2.492	2.47E-65
SSX2IP	ENSG0000	5.67	128.871	-4.283	5.29E-72
KIF17	ENSG0000	5.19	48.529	-3	3.02E-62
TSPAN1	ENSG0000	0.43	46.769	-5.062	4.11E-42
FAAH	ENSG0000	10.78	88.707	-2.929	1.56E-27
NSUN4	ENSG0000	12.47	124.611	-3.221	8.88E-48
F3	ENSG0000	6.15	56.798	-3.015	3.61E-40
MTFR1L	ENSG0000	24.729	115.511	-2.179	2.59E-80
PROX1	ENSG0000	0.11	7.66	-2.964	1.20E-47
2-Mar	ENSG0000	1.9	17.94	-2.707	4.11E-42
STK11	ENSG0000	35.68	158.012	-2.116	1.92E-77
TREH	ENSG0000	0.04	3.47	-2.104	1.35E-30
ZNF541	ENSG0000	0.36	27.441	-4.386	1.19E-99
TNP1	ENSG0000	0.14	8134.285	-12.801	7.53E-75
SPACA1	ENSG0000	0.06	58.27	-5.805	3.22E-75
SGIP1	ENSG0000	0.48	9.89	-2.879	8.96E-61
ZC2HC1B	ENSG0000	0.06	20.559	-4.346	7.69E-59
PMFBP1	ENSG0000	1.03	26.259	-3.747	1.74E-61
SLC16A7	ENSG0000	0.34	13.24	-3.41	2.78E-59
TTLL5	ENSG0000	14.28	119.56	-2.98	6.26E-54
PPP4R4	ENSG0000	0.67	11.55	-2.91	6.47E-70
ZC2HC1C	ENSG0000	2.17	104.567	-5.058	2.88E-69
KCNIP2	ENSG0000	0.91	7.33	-2.125	1.80E-84
EQTN	ENSG0000	0.02	43.281	-5.44	1.58E-71
INSL6	ENSG0000	0.08	20.629	-4.324	7.39E-83
MAGEB4	ENSG0000	0	8.37	-3.228	3.61E-87
SLC25A2	ENSG0000	0.04	8.29	-3.159	8.60E-76
TTLL2	ENSG0000	0.08	25.259	-4.604	5.54E-63
SNX19	ENSG0000	9.06	39.541	-2.011	2.47E-45
TEX11	ENSG0000	1.46	13.4	-2.549	4.75E-39
KIAA1217	ENSG0000	3.4	21.941	-2.382	2.25E-34
SEPT7P9	ENSG0000	0.25	61.699	-5.648	6.01E-71
LYZL1	ENSG0000	0	74.49	-6.238	2.80E-71
IQSEC3	ENSG0000	0.93	8.22	-2.256	9.55E-29
SOHLH2	ENSG0000	1.38	18.47	-3.032	3.54E-44
FAM53C	ENSG0000	16.22	93.428	-2.455	4.84E-65
SIL1	ENSG0000	21.22	108.668	-2.303	2.10E-64
SORBS3	ENSG0000	92.212	377.805	-2.023	1.81E-49
RDH10	ENSG0000	4.63	25.49	-2.234	1.90E-32
TEX14	ENSG0000	0.83	44.73	-4.643	2.48E-70
PAPD5	ENSG0000	5.72	27.17	-2.068	9.73E-44
RP11-408E	ENSG0000	0	15.94	-4.082	1.14E-72
RGSL1	ENSG0000	0	18.939	-4.318	1.79E-67

GJA8	ENSG0000(	0	6.18	-2.844	3.87E-65
MAPK8IP1	ENSG0000(	7.01	38.77	-2.312	1.31E-51
PILRB	ENSG0000(	17.859	164.505	-3.134	8.44E-79
HPCA	ENSG0000(	1.01	13.56	-2.857	1.87E-50
POLK	ENSG0000(	3.42	19.631	-2.223	2.45E-67
OBP2A	ENSG0000(	0.13	4.63	-2.317	4.26E-25
TBX22	ENSG0000(	0.09	28.23	-4.745	1.74E-110
PRM2	ENSG0000(	0.06	15349.46	-13.822	2.20E-78
LDB3	ENSG0000(	0.32	5.79	-2.363	1.01E-57
SPATA1	ENSG0000(	0.64	12.14	-3.002	6.46E-91
LRRC39	ENSG0000(	0.29	7.59	-2.735	1.83E-58
PQLC1	ENSG0000(	33.13	142.606	-2.073	6.14E-45
PHF24	ENSG0000(	2.19	22.161	-2.86	2.79E-46
IFT81	ENSG0000(	6.56	29.531	-2.014	6.84E-45
RASSF8	ENSG0000(	4.9	89.927	-3.946	2.01E-34
OPTN	ENSG0000(	25.801	109.987	-2.05	7.20E-63
IL13RA2	ENSG0000(	2.08	33.19	-3.473	5.11E-38
ESX1	ENSG0000(	0.34	11.85	-3.261	2.01E-59
TNFAIP6	ENSG0000(	1.34	16.12	-2.871	9.78E-36
LPGAT1	ENSG0000(	3.9	20.8	-2.154	7.45E-31
G0S2	ENSG0000(	6.37	36.45	-2.345	7.78E-32
INHA	ENSG0000(	1.06	122.301	-5.903	5.69E-75
OBSL1	ENSG0000(	28.67	423.347	-3.838	1.82E-73
FAM209A	ENSG0000(	0.23	136.749	-6.807	1.58E-79
SLPI	ENSG0000(	0.69	33.451	-4.349	1.03E-29
WFDC3	ENSG0000(	0.97	131.78	-6.075	1.69E-57
GTSF1L	ENSG0000(	0.2	278.879	-7.866	9.77E-72
RNF114	ENSG0000(	72.549	344.7	-2.233	1.87E-42
TP53TG5	ENSG0000(	0.15	21.35	-4.281	5.55E-58
IRGC	ENSG0000(	0.07	197.129	-7.533	1.16E-76
TCP11	ENSG0000(	0.31	526.795	-8.654	2.36E-84
OPN5	ENSG0000(	0	8.19	-3.2	7.40E-71
SSUH2	ENSG0000(	3.09	24.931	-2.664	4.90E-26
LRRC29	ENSG0000(	1.56	9.42	-2.025	8.06E-73
PIWIL1	ENSG0000(	1.4	59.358	-4.652	1.91E-28
GPR18	ENSG0000(	1	13.03	-2.81	5.02E-33
GRK4	ENSG0000(	1.46	81.673	-5.071	3.70E-100
SOX9	ENSG0000(	1.19	33.889	-3.994	1.41E-43
TEKT3	ENSG0000(	0.9	65.348	-5.126	3.54E-75
SLC25A35	ENSG0000(	7.41	41.911	-2.351	6.56E-43
OPRL1	ENSG0000(	1.36	8.81	-2.055	1.10E-48
LINC00029	ENSG0000(	0	7.04	-3.007	4.04E-77
SDCBP2	ENSG0000(	1.11	9.68	-2.34	4.93E-53
FAM182A	ENSG0000(	0.46	13.92	-3.353	1.42E-42
TMX4	ENSG0000(	12.26	66.638	-2.351	3.67E-67
PCSK2	ENSG0000(	0.02	6.12	-2.803	7.24E-50
SIRPD	ENSG0000(	0.21	75.598	-5.984	8.29E-66
ST3GAL3	ENSG0000(	6.07	78.978	-3.5	8.12E-66
TMEM53	ENSG0000(	9.27	44.099	-2.135	1.18E-38

F10	ENSG00000	1.49	13.81	-2.572	1.53E-12
TSKS	ENSG00000	2.87	172.469	-5.486	1.08E-62
SSX1	ENSG00000	0.05	8.49	-3.176	4.67E-74
HSPA2	ENSG00000	6.41	158.023	-4.424	4.44E-64
PZP	ENSG00000	0.08	4.39	-2.319	1.33E-45
PRDM7	ENSG00000	0.14	4.68	-2.317	7.90E-65
WDR60	ENSG00000	6.82	44.73	-2.548	3.14E-65
FAM78A	ENSG00000	5.23	39.76	-2.71	1.09E-50
HRASLS	ENSG00000	3.55	99.76	-4.469	9.64E-68
PLA2G5	ENSG00000	2.17	20.24	-2.744	2.61E-40
FBXL16	ENSG00000	2.73	19	-2.423	6.56E-43
TUBA4A	ENSG00000	12.95	72.881	-2.405	1.99E-30
HIP1	ENSG00000	6.19	57.939	-3.035	8.59E-38
SPINK2	ENSG00000	4.7	510.794	-6.488	2.82E-65
POM121L9	ENSG00000	0.3	70.908	-5.79	3.26E-90
IGLL1	ENSG00000	0	29.13	-4.913	3.02E-67
RIBC2	ENSG00000	1.36	41.489	-4.17	3.03E-53
KRT17	ENSG00000	0.38	5.21	-2.17	2.73E-04
STRIP2	ENSG00000	4.43	26.359	-2.333	1.68E-20
KLHDC10	ENSG00000	2.62	37.25	-3.401	9.86E-64
FEZF1	ENSG00000	0.06	5.49	-2.614	1.33E-48
PDE11A	ENSG00000	0.06	4.08	-2.261	4.97E-35
TTBK2	ENSG00000	2.71	24.299	-2.77	5.48E-58
USP6	ENSG00000	1.96	19.04	-2.759	3.45E-29
SHBG	ENSG00000	0.32	13.94	-3.501	5.91E-54
LRRC6	ENSG00000	6.06	40.419	-2.553	1.40E-23
KLK10	ENSG00000	0.93	8.81	-2.346	7.19E-42
RNASE1	ENSG00000	75.771	1255.111	-4.032	9.50E-54
QRICH2	ENSG00000	4.03	39.379	-3.005	3.39E-50
FOXJ1	ENSG00000	0.2	22.21	-4.274	1.84E-25
VCY	ENSG00000	0.69	129.489	-6.271	2.01E-59
SYT5	ENSG00000	1.06	7.77	-2.09	6.77E-39
GAMT	ENSG00000	14.88	73.791	-2.236	9.95E-30
TSPAN16	ENSG00000	0.07	174.925	-7.361	6.77E-74
GDF1	ENSG00000	0.5	6.5	-2.322	1.67E-63
KIF1A	ENSG00000	1.83	22.11	-3.03	1.98E-31
SLC27A1	ENSG00000	6.22	28.959	-2.053	3.89E-59
RSPH3	ENSG00000	1.3	16.91	-2.961	7.96E-62
STK33	ENSG00000	6.15	58.282	-3.052	1.93E-57
KIAA1683	ENSG00000	1.04	45.781	-4.519	4.93E-73
SAG	ENSG00000	0.08	10.06	-3.356	3.92E-56
TNNT3	ENSG00000	0.87	7.67	-2.213	2.61E-16
PNPLA7	ENSG00000	1.05	8.93	-2.276	2.77E-55
HBZ	ENSG00000	0.06	8.79	-3.207	1.29E-61
GATA5	ENSG00000	0.62	14.53	-3.261	1.12E-40
RBBP8NL	ENSG00000	0.02	3.59	-2.17	2.68E-35
POMT1	ENSG00000	16.829	116.791	-2.724	1.65E-86
PNCK	ENSG00000	4.56	23.02	-2.111	1.19E-30
PKDREJ	ENSG00000	0.06	3.38	-2.047	3.55E-74

HSD17B3	ENSG00000	0.44	44.919	-4.995	3.50E-78
HABP4	ENSG00000	9.66	58.1	-2.471	9.79E-55
RGN	ENSG00000	0.55	5.67	-2.105	9.97E-12
POLN	ENSG00000	0.4	11.76	-3.188	3.79E-114
SYNE1	ENSG00000	7.63	43.961	-2.381	1.38E-50
TTLL9	ENSG00000	0.26	17.21	-3.853	1.19E-75
TEX101	ENSG00000	0.07	184.004	-7.434	7.41E-94
RAB11FIP4	ENSG00000	14.16	85.609	-2.514	4.43E-21
GALNT15	ENSG00000	0.2	6.83	-2.706	1.18E-79
RHOXF2	ENSG00000	0.21	33.77	-4.845	5.98E-52
KHDRBS3	ENSG00000	20.11	195.265	-3.217	2.05E-46
ZSCAN5A	ENSG00000	5.94	34.649	-2.361	1.09E-59
USP29	ENSG00000	0.01	4.37	-2.411	1.58E-75
LRRC9	ENSG00000	0.09	7.57	-2.975	6.07E-54
PODNL1	ENSG00000	0.74	9.2	-2.551	6.43E-39
ZNF20	ENSG00000	2.08	11.4	-2.009	9.88E-48
SPATA6	ENSG00000	5.2	188.784	-4.936	3.03E-62
PRKAA1	ENSG00000	9.59	48.839	-2.235	5.53E-61
SERPINF1	ENSG00000	61.188	405.426	-2.708	2.01E-29
RGS22	ENSG00000	0.42	86.521	-5.946	4.03E-107
MATN2	ENSG00000	7.8	35.189	-2.04	4.44E-16
SCP2D1	ENSG00000	0	70.599	-6.162	5.38E-74
SNAP25	ENSG00000	0.5	6.64	-2.349	1.45E-36
HAPLN2	ENSG00000	0.14	4.36	-2.233	2.43E-99
IGHMBP2	ENSG00000	8.14	40.649	-2.188	5.78E-80
MTL5	ENSG00000	6.08	65.1	-3.223	4.25E-45
VSTM2L	ENSG00000	0.38	5.55	-2.247	9.53E-21
OSER1	ENSG00000	29.421	150.601	-2.317	1.61E-51
PDE6A	ENSG00000	0.03	3.59	-2.156	2.75E-74
TPTE2	ENSG00000	0.62	10.75	-2.859	1.56E-57
RNF17	ENSG00000	4.1	31.139	-2.656	3.13E-21
SPG20	ENSG00000	8.29	39.609	-2.128	2.85E-30
FAM104A	ENSG00000	19.1	193.635	-3.275	1.92E-56
MYH11	ENSG00000	4.68	136.541	-4.598	3.85E-34
PDZD2	ENSG00000	3.02	23.669	-2.617	5.92E-72
MYO18B	ENSG00000	0.77	31.051	-4.179	1.80E-53
FAM83F	ENSG00000	4.64	55.61	-3.327	2.63E-49
SEC14L4	ENSG00000	0.33	6.1	-2.416	8.00E-69
ZDHHC8P1	ENSG00000	1.53	10.05	-2.127	1.70E-51
LRRIQ1	ENSG00000	0.35	15.09	-3.575	1.24E-58
MICALCL	ENSG00000	0.23	19.77	-4.078	3.82E-64
MICAL2	ENSG00000	4.13	65.199	-3.69	2.09E-104
TEX15	ENSG00000	0.16	14.4	-3.731	2.08E-81
PEBP4	ENSG00000	0.18	7.54	-2.855	4.88E-65
MRO	ENSG00000	0.2	26.25	-4.505	2.77E-94
GSTM5	ENSG00000	1.94	14.84	-2.43	1.96E-24
GSTM3	ENSG00000	17.59	989.598	-5.736	3.22E-86
HMGCS2	ENSG00000	0.1	41.08	-5.258	2.40E-22
SPIRE1	ENSG00000	3.54	39.281	-3.149	4.11E-72

LPIN1	ENSG0000(	13.98	115.032	-2.953	1.00E-47
SOX5	ENSG0000(	0.5	40.689	-4.797	1.08E-65
PRH2	ENSG0000(	0.06	3.83	-2.188	4.62E-21
PIWIL4	ENSG0000(	2.17	11.8	-2.014	1.08E-48
SPOCD1	ENSG0000(	0.96	17.3	-3.223	1.08E-80
PHC2	ENSG0000(	18.11	97.951	-2.372	6.59E-35
HOOK1	ENSG0000(	1.99	93.409	-4.981	2.51E-55
RNF138	ENSG0000(	28.41	188.47	-2.688	1.46E-30
TPGS2	ENSG0000(	36.931	226.976	-2.587	2.60E-52
TPP2	ENSG0000(	23.02	102.728	-2.11	2.74E-55
USP30	ENSG0000(	5.28	28.32	-2.223	1.11E-55
FAM71F1	ENSG0000(	2.37	144.547	-5.433	9.12E-42
SRPK2	ENSG0000(	27.2	132.348	-2.241	1.73E-39
PRPH	ENSG0000(	8.41	96.241	-3.369	5.29E-36
FAM186B	ENSG0000(	0.69	72.892	-5.45	9.16E-74
FAIM2	ENSG0000(	3.73	45.1	-3.285	1.22E-36
PAN2	ENSG0000(	27.331	127.053	-2.176	1.15E-64
MAP7	ENSG0000(	5.26	35.321	-2.537	2.05E-46
LACE1	ENSG0000(	3.62	27.729	-2.637	8.19E-42
SMPD2	ENSG0000(	7.38	74.048	-3.163	1.09E-67
RAB11FIP5	ENSG0000(	15.6	182.328	-3.465	7.61E-50
SLC9A5	ENSG0000(	1.05	9.95	-2.417	4.41E-68
GLUL	ENSG0000(	185.155	806.146	-2.116	3.58E-44
TSGA10	ENSG0000(	3.68	71.031	-3.944	3.86E-70
STAB2	ENSG0000(	0.2	4.29	-2.14	7.24E-44
USP44	ENSG0000(	0.59	14.78	-3.311	1.12E-23
LMO7	ENSG0000(	3.8	23.39	-2.345	9.01E-31
SPDYE1	ENSG0000(	0.31	9.01	-2.934	7.73E-61
MMD2	ENSG0000(	0.02	8.34	-3.195	1.71E-70
NKX2-8	ENSG0000(	0.11	10.14	-3.327	9.98E-57
ZFHX2	ENSG0000(	1.09	17.689	-3.161	4.19E-56
MYCBPAP	ENSG0000(	0.28	179.606	-7.141	1.59E-103
ODF2	ENSG0000(	30.5	425.111	-3.758	1.16E-55
RALGPS1	ENSG0000(	4.56	25.591	-2.258	5.39E-54
SLC2A8	ENSG0000(	14.56	103.902	-2.753	8.25E-67
GARNL3	ENSG0000(	1.33	9.41	-2.16	3.46E-44
WDR38	ENSG0000(	0.02	9.31	-3.337	6.42E-52
GABBR2	ENSG0000(	0.29	4.18	-2.006	1.17E-39
HEMGN	ENSG0000(	0.02	50.52	-5.658	4.60E-66
NR5A1	ENSG0000(	2.18	18.35	-2.605	2.26E-58
IL11RA	ENSG0000(	6.27	30.38	-2.11	9.97E-19
RNF38	ENSG0000(	15.6	112.813	-2.777	4.96E-50
SPAG8	ENSG0000(	0.38	51.46	-5.248	3.47E-90
TMEM8B	ENSG0000(	3.74	26.999	-2.562	1.23E-52
FOXP4	ENSG0000(	6.84	37.449	-2.294	1.00E-12
KLC4	ENSG0000(	11.11	50.171	-2.079	2.03E-52
SLC22A7	ENSG0000(	0.16	6.52	-2.697	3.51E-49
SLC22A23	ENSG0000(	1.99	15.1	-2.429	3.53E-23
LRRC1	ENSG0000(	2.1	18.939	-2.685	5.71E-39

FGFBP2	ENSG00000	0.91	10.64	-2.607	6.82E-26
TTC29	ENSG00000	0.05	79.798	-6.266	2.55E-74
MYO7A	ENSG00000	2.41	43.849	-3.717	2.68E-82
MMP20	ENSG00000	0	5.04	-2.595	4.62E-50
TRIM29	ENSG00000	0.26	12.94	-3.468	5.07E-39
PPP2R1B	ENSG00000	11.98	58.44	-2.195	2.21E-42
UNC13C	ENSG00000	0.07	8.91	-3.211	2.05E-43
MAPKBP1	ENSG00000	4.16	66.758	-3.715	5.43E-91
PARP6	ENSG00000	35.849	156.356	-2.094	5.34E-72
LRRC49	ENSG00000	3.81	19.03	-2.058	2.53E-58
ITPKA	ENSG00000	1.7	16.46	-2.693	5.26E-39
PAK6	ENSG00000	0.68	9.27	-2.612	6.38E-63
TTLL7	ENSG00000	1.18	19.45	-3.23	2.98E-83
SLC44A5	ENSG00000	0.18	4.79	-2.295	2.72E-24
TRIM54	ENSG00000	0.72	48.982	-4.861	9.28E-119
LOXL4	ENSG00000	0.47	6.96	-2.437	2.96E-29
STAMBPL1	ENSG00000	2.71	18.63	-2.404	5.43E-53
STAT4	ENSG00000	2.32	31.15	-3.276	2.40E-47
MDH1B	ENSG00000	1.09	33.8	-4.057	1.78E-70
MNS1	ENSG00000	1.55	52.269	-4.385	9.71E-56
FAM13A	ENSG00000	3.7	21.48	-2.258	7.79E-52
MMRN1	ENSG00000	0.4	4.75	-2.038	1.37E-32
PRDM5	ENSG00000	0.49	6.05	-2.242	1.86E-51
LEF1	ENSG00000	5.61	36.671	-2.511	8.51E-31
MTTP	ENSG00000	0.2	5.43	-2.422	3.72E-23
MAPK8IP3	ENSG00000	10.61	74.227	-2.696	6.59E-61
TTLL8	ENSG00000	0	9.27	-3.36	5.03E-65
PLCZ1	ENSG00000	0.04	82.773	-6.332	2.38E-69
PPFIA2	ENSG00000	0.26	11.76	-3.34	6.62E-78
TMEM132I	ENSG00000	0.56	7.15	-2.385	2.07E-50
FAM222A	ENSG00000	4.45	31.31	-2.568	3.04E-55
FOXN4	ENSG00000	0.27	14.41	-3.601	2.46E-72
SMIM2	ENSG00000	0.05	27.599	-4.768	7.90E-62
MORN3	ENSG00000	0.85	94.313	-5.687	5.85E-61
SLAIN1	ENSG00000	3.96	29.709	-2.63	1.88E-70
RNF113B	ENSG00000	0.13	59.01	-5.731	2.56E-81
TTC6	ENSG00000	0.2	8.76	-3.024	9.59E-48
REM2	ENSG00000	0.52	5.89	-2.18	2.27E-51
TSSK4	ENSG00000	0.71	57.531	-5.097	9.79E-77
ESR2	ENSG00000	0.56	6.78	-2.318	2.17E-104
FAM181A	ENSG00000	0	42.051	-5.428	3.10E-73
GCNT3	ENSG00000	0.33	4.9	-2.149	9.99E-37
MAN2C1	ENSG00000	41.961	213.796	-2.322	3.90E-77
GOLGA6D	ENSG00000	0.13	14.13	-3.743	3.51E-55
PCSK6	ENSG00000	2.74	16.03	-2.187	1.74E-44
WDR93	ENSG00000	0.31	13.65	-3.483	2.38E-66
SH3GL3	ENSG00000	2.34	119.353	-5.171	8.59E-57
12-Sep	ENSG00000	0.39	212.187	-7.261	6.15E-76
SLC5A2	ENSG00000	0.28	27.441	-4.474	1.39E-66

MYLK3	ENSG00000	0.21	6.99	-2.723	1.08E-74
KIFC3	ENSG00000	12.55	104.343	-2.959	9.39E-62
ZCCHC14	ENSG00000	5.96	48.05	-2.817	2.49E-70
OSGIN1	ENSG00000	1.24	20.89	-3.289	1.66E-81
RPL3L	ENSG00000	0.59	10.99	-2.915	3.47E-60
GALNS	ENSG00000	8.42	51.549	-2.48	1.51E-81
GAS8	ENSG00000	7.7	38.641	-2.188	1.87E-71
GID4	ENSG00000	8.18	81.109	-3.161	3.73E-54
KIF2B	ENSG00000	0	89.89	-6.506	1.05E-71
SPATA22	ENSG00000	7.4	169.248	-4.341	8.54E-49
SKAP1	ENSG00000	4.71	27.509	-2.32	8.50E-31
LRRC46	ENSG00000	0.84	83.221	-5.516	2.10E-74
SSH2	ENSG00000	6.64	38.88	-2.384	2.87E-59
SPACA3	ENSG00000	0.06	164.871	-7.29	4.89E-76
SLC25A52	ENSG00000	0.16	7.68	-2.904	7.72E-53
GATA6	ENSG00000	1.08	8.25	-2.153	1.65E-12
GREB1L	ENSG00000	0.21	5.56	-2.439	2.18E-55
ZMYND15	ENSG00000	1.31	43.901	-4.281	2.48E-59
RNF157	ENSG00000	4.21	45.05	-3.144	2.47E-54
MBD1	ENSG00000	20.71	132.357	-2.619	5.72E-89
ZCCHC2	ENSG00000	4.42	25.61	-2.296	5.67E-66
FBXO15	ENSG00000	1.03	34.63	-4.134	4.75E-81
TMEM91	ENSG00000	6.61	48.86	-2.712	1.19E-61
SIRT3	ENSG00000	8.93	54.681	-2.487	4.86E-76
EVI5L	ENSG00000	5.06	49.841	-3.069	1.35E-76
ZNF473	ENSG00000	6.37	39.14	-2.445	3.95E-47
FAM71E1	ENSG00000	6.76	179.158	-4.537	1.62E-51
SLC2A5	ENSG00000	2.93	55.372	-3.842	2.54E-61
MMEL1	ENSG00000	1.12	36.88	-4.159	7.27E-47
FHAD1	ENSG00000	0.7	38.609	-4.542	1.54E-64
MYOM3	ENSG00000	0.73	11.64	-2.869	1.79E-67
SYPL2	ENSG00000	0.62	12.18	-3.024	3.90E-78
PROK1	ENSG00000	0.29	68.029	-5.742	2.35E-61
MPC2	ENSG00000	48.691	303.32	-2.615	2.17E-56
MAEL	ENSG00000	3.23	164.369	-5.289	6.49E-53
ILDR2	ENSG00000	0.33	9.21	-2.941	9.31E-78
ISG20L2	ENSG00000	30.969	208.918	-2.715	2.63E-41
FAM163A	ENSG00000	0.06	15.47	-3.958	2.15E-60
RORC	ENSG00000	0.74	15.02	-3.203	5.15E-69
FAM63A	ENSG00000	2.77	30.6	-3.067	3.80E-75
SEMA6C	ENSG00000	11.8	73.5	-2.541	3.52E-60
OAZ3	ENSG00000	7.05	424.581	-5.724	2.05E-53
HORMAD1	ENSG00000	7.79	70.039	-3.015	6.94E-31
GABPB2	ENSG00000	2.37	18.5	-2.533	5.34E-83
HHIPL2	ENSG00000	0.66	13.93	-3.169	3.43E-57
NUP210L	ENSG00000	0.03	21.95	-4.478	1.97E-87
SCCPDH	ENSG00000	11.02	131.652	-3.464	3.72E-61
MLK4	ENSG00000	0.78	8.68	-2.443	2.34E-45
ETNK2	ENSG00000	19.79	177.995	-3.106	3.37E-75

PPFIA4	ENSG00000	1.46	8.87	-2.004	1.05E-52
PLEKHA6	ENSG00000	0.63	7.4	-2.366	7.83E-13
OSR1	ENSG00000	0.66	7.84	-2.413	1.15E-09
REG3G	ENSG00000	0.07	47.629	-5.506	7.26E-53
ST6GAL2	ENSG00000	0.09	4.27	-2.273	5.17E-27
NPHP1	ENSG00000	4.56	34.071	-2.657	6.16E-60
NT5DC4	ENSG00000	0.27	18.699	-3.955	7.60E-33
RABL2A	ENSG00000	4.7	39.36	-2.824	1.40E-65
FAHD2B	ENSG00000	14.5	83.528	-2.447	2.11E-66
LYG1	ENSG00000	0.65	7.14	-2.303	7.19E-71
KIAA1715	ENSG00000	5.58	32.139	-2.332	5.11E-38
ZNF385B	ENSG00000	0.54	14.17	-3.3	1.37E-86
PHOSPHO2	ENSG00000	2.97	19.55	-2.372	7.06E-56
GULP1	ENSG00000	1.22	13.65	-2.722	9.35E-23
UNC80	ENSG00000	0.13	5.85	-2.6	1.62E-95
SPAG16	ENSG00000	7.24	45.959	-2.511	1.24E-57
HES6	ENSG00000	4.58	30.181	-2.482	1.60E-35
STK11IP	ENSG00000	18.96	115.768	-2.548	3.63E-79
OSBPL10	ENSG00000	3.98	73.439	-3.902	7.14E-46
SLC22A14	ENSG00000	0.04	15.08	-3.951	4.09E-71
IGSF11	ENSG00000	0.5	44.929	-4.936	2.65E-98
TMEM108	ENSG00000	3.87	18.67	-2.014	6.00E-29
SPATA16	ENSG00000	0.06	44.059	-5.41	1.59E-68
LPP	ENSG00000	2.59	96.609	-4.765	1.41E-69
NICN1	ENSG00000	5.38	24.58	-2.003	5.02E-63
VPRBP	ENSG00000	13.53	69.642	-2.281	1.06E-46
LYAR	ENSG00000	19.24	105.799	-2.4	7.15E-33
SLC10A6	ENSG00000	0.04	5.34	-2.608	1.26E-52
SCD5	ENSG00000	1.38	28.281	-3.621	4.04E-49
ROPN1L	ENSG00000	0.8	463.587	-8.012	6.03E-79
UGT3A1	ENSG00000	0.21	10.61	-3.262	1.01E-38
PLK2	ENSG00000	7.97	41.231	-2.235	2.80E-24
IQGAP2	ENSG00000	9.62	59.399	-2.508	2.50E-29
SPATA9	ENSG00000	0.25	36.661	-4.913	2.58E-69
TSLP	ENSG00000	0.1	5.58	-2.581	2.86E-34
LECT2	ENSG00000	0.06	11.74	-3.587	1.77E-57
TIMD4	ENSG00000	0.59	184.221	-6.864	1.20E-70
RMND5B	ENSG00000	19.71	121.532	-2.565	6.07E-51
FAM50B	ENSG00000	2.13	18.28	-2.623	1.62E-75
FAM217A	ENSG00000	0.04	26.5	-4.725	2.00E-83
KLHL3	ENSG00000	2.12	13.13	-2.179	1.57E-78
HIST1H2BA	ENSG00000	0.08	6.67	-2.828	1.14E-49
RASGEF1C	ENSG00000	0.18	9.11	-3.099	7.69E-79
TCTE1	ENSG00000	0.51	40.221	-4.771	1.87E-71
RNF217	ENSG00000	0.94	13.25	-2.877	4.56E-71
PNLDC1	ENSG00000	1.59	14.8	-2.609	1.15E-21
SDK1	ENSG00000	1.09	15.23	-2.957	8.96E-40
TRIM50	ENSG00000	0.21	12.8	-3.512	3.26E-104
RAB41	ENSG00000	0.17	5.03	-2.366	9.65E-70

ZNF711	ENSG0000(	2.02	35.859	-3.609	3.39E-55
NXF3	ENSG0000(	0.46	239.187	-7.362	5.54E-99
FBXO25	ENSG0000(	10.36	112.469	-3.32	4.46E-68
FATE1	ENSG0000(	0.73	310.316	-7.491	5.08E-81
MAGEA4	ENSG0000(	0.72	29.219	-4.135	1.28E-21
SLC25A37	ENSG0000(	13.13	56.539	-2.026	6.60E-52
STAR	ENSG0000(	1.94	60.199	-4.38	3.95E-53
SNTG1	ENSG0000(	0	5.98	-2.803	7.35E-38
PXDNL	ENSG0000(	0.93	6.81	-2.017	4.60E-13
TRIM55	ENSG0000(	0.15	18.67	-4.096	4.91E-100
FAM135B	ENSG0000(	0.21	20.45	-4.148	5.33E-60
ZNF462	ENSG0000(	3.18	16.16	-2.037	4.22E-36
WDR31	ENSG0000(	0.62	5.55	-2.015	6.36E-61
PTGES	ENSG0000(	3.31	21.681	-2.396	2.71E-27
HMCN2	ENSG0000(	0.75	11.24	-2.806	1.74E-65
ZEB1	ENSG0000(	2.92	17.73	-2.256	5.16E-34
FAM13C	ENSG0000(	0.73	8.71	-2.489	1.56E-52
LRIT1	ENSG0000(	0.01	7.89	-3.138	2.24E-61
RGR	ENSG0000(	0	4.22	-2.384	1.08E-46
HTR7	ENSG0000(	0.27	7.31	-2.71	2.62E-63
LRRC27	ENSG0000(	4.24	123.596	-4.572	1.66E-84
NKX6-2	ENSG0000(	0.1	3.63	-2.074	1.86E-16
PAOX	ENSG0000(	4.21	95.457	-4.211	5.78E-62
SESN3	ENSG0000(	2.54	63.201	-4.181	1.70E-57
TTC12	ENSG0000(	4.64	58.48	-3.399	6.59E-75
NCAM1	ENSG0000(	1.28	9.65	-2.224	4.08E-18
GLB1L2	ENSG0000(	1.76	11.68	-2.2	3.53E-49
GGTLC1	ENSG0000(	0	21.84	-4.514	3.95E-62
TKFC	ENSG0000(	24.669	112.938	-2.15	1.16E-64
ROM1	ENSG0000(	1.18	7.98	-2.042	2.66E-53
ZP1	ENSG0000(	0.02	9.21	-3.323	2.62E-57
OOSP2	ENSG0000(	0	19.2	-4.336	6.60E-94
FEZ1	ENSG0000(	3.32	36.539	-3.119	5.16E-55
SPATA25	ENSG0000(	0.63	30.33	-4.265	2.95E-79
FAM57B	ENSG0000(	0.23	154.214	-6.979	2.22E-73
HIRIP3	ENSG0000(	12.28	57.472	-2.138	3.58E-54
MPP7	ENSG0000(	0.68	9.41	-2.631	1.77E-50
CCDC7	ENSG0000(	0.08	41.9	-5.312	1.92E-82
FRMPD2B	ENSG0000(	0.01	5.53	-2.693	3.64E-54
LATS2	ENSG0000(	3.43	19.5	-2.21	3.09E-52
KIAA1328	ENSG0000(	1.99	18.539	-2.708	6.74E-93
LYPD6B	ENSG0000(	2.55	48.87	-3.812	5.03E-38
SPATA4	ENSG0000(	0.09	120.4	-6.799	1.35E-81
FSIP1	ENSG0000(	0.28	6.06	-2.464	1.61E-65
PPP1R1C	ENSG0000(	3.74	52.749	-3.503	1.92E-49
PIH1D2	ENSG0000(	0.69	69.271	-5.378	2.73E-78
TEX12	ENSG0000(	0.17	12.71	-3.551	1.82E-87
TKTL2	ENSG0000(	0.07	34.91	-5.069	3.66E-80
LYZL2	ENSG0000(	0	90.628	-6.518	5.95E-73

RAD9B	ENSG00000	0.38	16.27	-3.645	1.49E-89
MAT1A	ENSG00000	0.26	9.89	-3.111	2.65E-98
MAGI1	ENSG00000	4.02	21.07	-2.136	1.56E-31
TEX30	ENSG00000	11.22	136.276	-3.49	5.79E-72
ME3	ENSG00000	1.31	20.53	-3.22	1.50E-59
FER	ENSG00000	4.33	22.32	-2.129	1.35E-48
SLC25A31	ENSG00000	0.09	33.529	-4.985	1.66E-82
TEX9	ENSG00000	2.18	14.37	-2.273	7.82E-61
NR3C2	ENSG00000	0.41	11.82	-3.185	3.79E-71
INPP1	ENSG00000	3.87	78.129	-4.022	1.57E-78
SLC25A4	ENSG00000	7.84	39.579	-2.199	8.58E-46
SERP2	ENSG00000	2.06	62.37	-4.372	4.25E-95
ZNF385D	ENSG00000	0.67	35.429	-4.447	1.78E-71
PABPC3	ENSG00000	0.23	28.479	-4.583	2.49E-73
GLT1D1	ENSG00000	1.83	21.421	-2.986	6.48E-29
RBM46	ENSG00000	1.66	10.63	-2.128	3.39E-27
TUBA3E	ENSG00000	0.38	208.846	-7.249	2.31E-33
TMEM163	ENSG00000	0.93	9.96	-2.506	7.58E-51
HSPB8	ENSG00000	2.53	14.19	-2.105	1.67E-15
GRID2	ENSG00000	0.19	38.991	-5.071	1.13E-71
SH2D6	ENSG00000	0.72	22.07	-3.746	8.22E-54
SPOCK1	ENSG00000	1.8	18.43	-2.795	2.64E-38
SUV39H2	ENSG00000	8.1	46.429	-2.382	2.59E-39
OLAH	ENSG00000	1.76	31.84	-3.573	3.11E-30
ZSCAN1	ENSG00000	1.2	11.2	-2.471	6.00E-49
TRIM36	ENSG00000	2.78	212.85	-5.822	3.80E-61
PLEKHH2	ENSG00000	0.92	6.99	-2.057	3.42E-31
IGSF10	ENSG00000	0.47	10.4	-2.955	2.09E-39
SPEF2	ENSG00000	1.26	11.68	-2.488	8.28E-64
NADK2	ENSG00000	5.12	43.761	-2.871	9.68E-65
SAR1B	ENSG00000	11.9	59.858	-2.238	8.00E-56
WDR78	ENSG00000	0.86	27.31	-3.928	1.97E-76
UTRN	ENSG00000	11.7	66.389	-2.408	2.76E-31
LMNTD1	ENSG00000	0.09	8.06	-3.055	3.80E-27
STK32B	ENSG00000	1.33	12.35	-2.518	4.69E-36
TEKT5	ENSG00000	0.27	43.51	-5.131	3.44E-68
RGPD3	ENSG00000	0.13	8.29	-3.039	2.30E-77
NR4A2	ENSG00000	0.76	7.26	-2.231	1.15E-48
SCN3A	ENSG00000	0.09	4.54	-2.346	1.44E-55
SLC25A27	ENSG00000	2.55	43.721	-3.655	7.94E-80
FRMD1	ENSG00000	0.1	10.17	-3.344	7.40E-87
FAM81B	ENSG00000	0.07	118.528	-6.804	1.77E-66
LINC00467	ENSG00000	17.05	364.127	-4.338	4.66E-37
INO80C	ENSG00000	19.91	111.26	-2.425	3.92E-57
TEX29	ENSG00000	0.11	63.711	-5.865	4.93E-77
SPACA7	ENSG00000	0	109.972	-6.794	3.79E-76
FBXL2	ENSG00000	1.67	22.49	-3.137	1.32E-58
GOLGA8F	ENSG00000	0	27.179	-4.817	2.05E-74
TGIF2LX	ENSG00000	0.14	10.76	-3.367	2.31E-41

ZNF599	ENSG00000	3.46	19.48	-2.199	1.52E-75
SEMA3D	ENSG00000	0.37	5.02	-2.136	1.79E-29
FAM134B	ENSG00000	1.08	76.399	-5.218	8.85E-69
TNIK	ENSG00000	1.6	20.269	-3.032	1.29E-41
FAM167A	ENSG00000	0.5	5.16	-2.038	1.10E-33
FAM27C	ENSG00000	0.74	54.149	-4.986	2.57E-71
SRSF12	ENSG00000	3.01	20.99	-2.455	2.90E-42
PSMA8	ENSG00000	0.13	18.84	-4.134	1.90E-92
NCAM2	ENSG00000	0.5	6.93	-2.402	4.65E-61
L3MBTL4	ENSG00000	0.51	14.34	-3.345	1.65E-135
PDE1C	ENSG00000	0.42	7.73	-2.62	9.03E-50
JAM2	ENSG00000	3.94	34.951	-2.863	2.93E-52
EPHB1	ENSG00000	0.77	7.48	-2.26	8.18E-41
14-Sep	ENSG00000	0.27	14.54	-3.613	4.74E-71
RSPH10B	ENSG00000	1.87	25.95	-3.231	4.29E-35
ODF1	ENSG00000	0	507.828	-8.991	3.90E-79
SLC25A28	ENSG00000	29.28	138.976	-2.209	9.36E-83
USP25	ENSG00000	9.7	47.679	-2.186	1.65E-52
PPM1J	ENSG00000	2.86	92.808	-4.603	6.89E-62
TRIM74	ENSG00000	0.13	7.66	-2.938	1.45E-106
MAGEC1	ENSG00000	0.91	9.31	-2.432	1.06E-28
LRGUK	ENSG00000	0.32	5.37	-2.271	9.81E-73
SETD9	ENSG00000	6.66	48.468	-2.691	6.19E-46
PDZD9	ENSG00000	0.06	19.09	-4.244	5.66E-67
OTOA	ENSG00000	0.86	8.66	-2.377	5.10E-36
SPAG17	ENSG00000	0.61	65.981	-5.379	2.99E-74
SAXO1	ENSG00000	0.03	48.321	-5.581	7.65E-70
TRIM42	ENSG00000	0	20	-4.392	2.32E-57
PXYLP1	ENSG00000	4.11	47.441	-3.245	5.87E-46
PSD3	ENSG00000	2.6	65.839	-4.215	6.70E-63
FAM161B	ENSG00000	2.42	21.45	-2.715	1.67E-40
GPR61	ENSG00000	0.4	4.66	-2.015	7.44E-77
TDRD9	ENSG00000	2.26	57.09	-4.155	1.08E-64
SH3RF2	ENSG00000	0.67	526.065	-8.302	2.13E-84
PPP2R2B	ENSG00000	1.2	24.75	-3.549	1.41E-39
FBXO43	ENSG00000	1.1	7.91	-2.085	1.71E-43
TYSND1	ENSG00000	8.32	43.239	-2.247	1.38E-51
ZFAND3	ENSG00000	39.73	219.214	-2.435	7.74E-45
MAPK13	ENSG00000	6.42	51.789	-2.831	3.13E-59
FBRS	ENSG00000	20.72	208.166	-3.268	2.91E-88
PHKG2	ENSG00000	34.551	242.106	-2.774	3.20E-57
VPS8	ENSG00000	10.75	47.31	-2.04	6.71E-64
PDE6D	ENSG00000	14.73	74.048	-2.254	7.49E-57
FAM92A1P	ENSG00000	0.94	8.37	-2.272	7.59E-46
SHCBP1L	ENSG00000	0.19	141.317	-6.902	9.46E-90
NMNAT2	ENSG00000	1.26	25.3	-3.541	2.37E-73
LYZL4	ENSG00000	0	68.74	-6.124	7.02E-73
SLC6A1	ENSG00000	0.24	6.23	-2.544	6.17E-65
LRP8	ENSG00000	3.5	30.519	-2.808	6.52E-65

SUSD3	ENSG0000(	20	102.508	-2.301	9.83E-28
HYDIN	ENSG0000(	1.14	15.63	-2.958	2.87E-71
ZNF19	ENSG0000(	1.7	17.82	-2.801	5.70E-85
MUM1L1	ENSG0000(	0.24	19.68	-4.06	1.66E-97
LCA5L	ENSG0000(	1.99	105.032	-5.148	8.18E-69
MEGF11	ENSG0000(	0.5	16.3	-3.528	8.30E-53
RADIL	ENSG0000(	3.41	22.161	-2.393	6.15E-46
UBXN11	ENSG0000(	15	84.617	-2.42	4.02E-59
HPD	ENSG0000(	2.7	15.98	-2.198	6.05E-23
LRRC43	ENSG0000(	0.89	12.05	-2.788	1.62E-63
SLC13A3	ENSG0000(	0.81	6.87	-2.12	9.93E-44
RHBDL2	ENSG0000(	0.2	9	-3.059	5.75E-80
RIBC1	ENSG0000(	2.95	49.148	-3.666	8.84E-52
POM121L2	ENSG0000(	0.04	6.44	-2.839	2.95E-68
GDPD5	ENSG0000(	11.84	62.969	-2.317	1.76E-44
PAGE5	ENSG0000(	0.45	32.78	-4.542	7.30E-25
VWA5B1	ENSG0000(	0.02	11.09	-3.567	2.58E-58
RAPGEF6	ENSG0000(	2.97	15.78	-2.08	7.13E-67
FBXW5	ENSG0000(	47.96	433.893	-3.151	1.03E-78
KCNE2	ENSG0000(	0.31	4.99	-2.193	1.26E-54
GOLGA6A	ENSG0000(	0.35	35.49	-4.756	6.81E-58
SCUBE1	ENSG0000(	0.5	9.14	-2.757	5.84E-59
STARD9	ENSG0000(	2.9	31.721	-3.069	5.33E-80
TGM7	ENSG0000(	0.12	8.09	-3.021	3.32E-42
RGL4	ENSG0000(	1.28	10.99	-2.395	6.56E-33
SPRR2G	ENSG0000(	0	3.23	-2.081	6.00E-37
TEPP	ENSG0000(	0.27	25.12	-4.362	6.64E-67
LRRC36	ENSG0000(	0.82	81.12	-5.496	3.21E-84
TPPP3	ENSG0000(	3.96	20.141	-2.092	3.79E-13
ZDHHC1	ENSG0000(	3.51	33.941	-2.954	1.45E-67
ZFYVE28	ENSG0000(	1.59	17.869	-2.865	8.29E-94
NPR2	ENSG0000(	3.65	27.729	-2.627	3.27E-40
TMEM234	ENSG0000(	6.73	41.76	-2.468	3.59E-85
KALRN	ENSG0000(	2.46	22.161	-2.743	4.31E-46
RSPH1	ENSG0000(	1.55	43.931	-4.139	2.14E-50
HSF2BP	ENSG0000(	0.83	31.73	-4.161	1.93E-82
PDXK	ENSG0000(	45.689	629.995	-3.756	5.99E-62
FTCD	ENSG0000(	0.54	64.11	-5.402	2.05E-69
SPATC1L	ENSG0000(	8.56	64.248	-2.771	7.27E-36
GPSM1	ENSG0000(	3.13	36.39	-3.178	4.43E-35
HIPK4	ENSG0000(	0.07	24.01	-4.547	3.31E-70
TMEM190	ENSG0000(	0.21	72.258	-5.92	1.93E-55
PPAPDC3	ENSG0000(	0.86	6.51	-2.014	3.00E-68
SIK3	ENSG0000(	6.73	47.441	-2.648	2.46E-77
LRRC71	ENSG0000(	0.13	51.739	-5.544	3.07E-72
LY6K	ENSG0000(	0.75	50.899	-4.89	3.90E-65
PPP1R16A	ENSG0000(	20.031	112.454	-2.432	4.82E-68
PGLYRP2	ENSG0000(	0.03	6.87	-2.934	2.07E-83
LRWD1	ENSG0000(	38.689	403.94	-3.351	4.32E-46

FBXL13	ENSG0000(	0.69	20.949	-3.699	3.10E-62
NAPEPLD	ENSG0000(	3.53	18.09	-2.075	2.13E-70
LRRC56	ENSG0000(	1.19	29.549	-3.802	9.39E-71
PLXDC1	ENSG0000(	3.84	19.271	-2.066	7.03E-22
GRIN2C	ENSG0000(	0.13	5.88	-2.606	4.48E-111
KLHL10	ENSG0000(	0.36	71.12	-5.729	7.97E-70
IZUMO2	ENSG0000(	0.24	86.228	-6.136	2.14E-69
SYCE2	ENSG0000(	1.22	36.99	-4.097	3.90E-79
ZNF653	ENSG0000(	6.61	43.761	-2.556	8.29E-80
WDR90	ENSG0000(	15.82	182.543	-3.448	4.20E-84
MEIOB	ENSG0000(	5.11	49.292	-3.041	1.45E-34
NEU3	ENSG0000(	2.47	17.27	-2.396	1.05E-84
PPP1R32	ENSG0000(	1.13	131.16	-5.955	1.37E-87
GNG3	ENSG0000(	0.37	14.78	-3.526	7.23E-56
ITIH3	ENSG0000(	0.88	10.56	-2.62	5.28E-61
TSSK3	ENSG0000(	0.62	44.501	-4.812	2.85E-104
UBXN10	ENSG0000(	0.62	13.86	-3.197	2.09E-65
TTLL10	ENSG0000(	0.12	63.751	-5.853	6.55E-67
SCNN1D	ENSG0000(	1.9	47.751	-4.071	2.44E-107
LRRIQ3	ENSG0000(	0.58	17.38	-3.54	2.09E-66
WDR63	ENSG0000(	0.41	22.13	-4.036	7.53E-66
HFM1	ENSG0000(	1.91	14.48	-2.411	1.13E-68
SLC9C2	ENSG0000(	0.09	10.25	-3.368	7.37E-107
KLHDC9	ENSG0000(	3.53	137.825	-4.938	6.72E-75
LRRC52	ENSG0000(	0	21.04	-4.462	1.76E-56
FAM71A	ENSG0000(	0	98.291	-6.634	4.26E-65
TDRD5	ENSG0000(	1.88	21.12	-2.941	2.00E-50
SNED1	ENSG0000(	1	13.68	-2.876	1.62E-46
SPATA17	ENSG0000(	2.52	90.408	-4.699	1.27E-51
WDR64	ENSG0000(	0.04	8.51	-3.193	9.08E-62
KLHDC8A	ENSG0000(	1.01	7.12	-2.014	1.76E-20
TRIM17	ENSG0000(	1.52	54.059	-4.45	8.70E-58
RFTN2	ENSG0000(	1.8	10.87	-2.084	1.42E-28
FAM84A	ENSG0000(	0.23	16.429	-3.825	2.16E-56
ZSWIM2	ENSG0000(	0	5.38	-2.674	7.93E-61
TEKT4	ENSG0000(	0.04	20.89	-4.396	5.59E-61
SPATA18	ENSG0000(	1.05	93.137	-5.521	3.67E-70
NOSTRIN	ENSG0000(	3.59	34.22	-2.94	2.44E-62
INHBB	ENSG0000(	1.3	21.61	-3.297	2.69E-62
PDHA2	ENSG0000(	0	68.071	-6.11	2.91E-81
PACRGL	ENSG0000(	8.04	56.001	-2.657	2.41E-63
SMCP	ENSG0000(	0	1037.502	-10.02	2.72E-81
TDRD10	ENSG0000(	4.22	110.468	-4.416	1.95E-47
PAQR3	ENSG0000(	7.25	39.749	-2.304	1.16E-58
HIPK1	ENSG0000(	10.01	48.12	-2.158	1.22E-54
IGFN1	ENSG0000(	0.16	10.74	-3.339	1.19E-60
PROK2	ENSG0000(	0.32	10.59	-3.134	4.58E-68
LMOD1	ENSG0000(	1.48	21.96	-3.211	3.92E-30
PDCL2	ENSG0000(	2.68	77.842	-4.421	1.01E-45

TSACC	ENSG00000	9.59	1237.917	-6.87	1.97E-63
STK36	ENSG00000	23.99	111.183	-2.166	1.68E-62
NEK10	ENSG00000	0.56	30.77	-4.348	3.35E-68
HDAC11	ENSG00000	4.56	122.03	-4.468	2.77E-67
GLB1L	ENSG00000	5.81	87.65	-3.702	8.58E-78
SPTA1	ENSG00000	0.1	3.99	-2.182	1.42E-54
ICA1L	ENSG00000	2.23	12.19	-2.03	6.88E-49
KIAA1407	ENSG00000	1.61	16.05	-2.708	1.36E-69
ERICH6	ENSG00000	0.07	33.4	-5.007	5.68E-74
TIPARP	ENSG00000	3.67	36.931	-3.022	1.14E-70
FANCD2OS	ENSG00000	0.06	111.121	-6.725	1.25E-73
MTHFD2L	ENSG00000	1.57	11.19	-2.246	2.27E-75
PLSCR2	ENSG00000	0.16	13.65	-3.659	5.99E-38
SPDYA	ENSG00000	0.78	42.94	-4.626	9.44E-90
ZDHHC3	ENSG00000	32.901	156.226	-2.213	9.37E-53
LZTFL1	ENSG00000	8.06	59.35	-2.736	5.39E-52
NMNAT3	ENSG00000	2.16	43.431	-3.814	5.31E-70
IFT122	ENSG00000	15.85	130.553	-2.965	2.57E-74
RPL39L	ENSG00000	59.272	269.603	-2.167	1.55E-28
SFMBT1	ENSG00000	6.03	39.11	-2.512	4.74E-40
UVSSA	ENSG00000	3.05	32.13	-3.032	9.29E-88
ZDHHC19	ENSG00000	1.68	114.039	-5.424	9.50E-81
SLC51A	ENSG00000	3.84	29.57	-2.659	1.34E-61
SGMS2	ENSG00000	0.5	9.53	-2.811	1.22E-47
SLC9B1	ENSG00000	0.99	47.669	-4.612	1.81E-73
HSPA4L	ENSG00000	1.06	92.27	-5.501	2.83E-82
GRM2	ENSG00000	2.44	21.01	-2.678	4.49E-36
SCRG1	ENSG00000	0.07	18.26	-4.17	4.52E-89
TMEM144	ENSG00000	4.08	23.429	-2.266	2.37E-36
ZNF474	ENSG00000	0.03	6.93	-2.945	6.01E-50
PRDM9	ENSG00000	0.05	4.52	-2.394	7.58E-78
SPZ1	ENSG00000	0.99	33.07	-4.098	1.22E-49
FAM170A	ENSG00000	0	21.6	-4.498	6.59E-78
ACSL6	ENSG00000	1.26	49.49	-4.482	3.01E-71
GDF9	ENSG00000	0.33	11.46	-3.228	6.94E-99
STXBP5	ENSG00000	2.89	15.14	-2.053	2.73E-53
HIST1H2A	ENSG00000	0	6.73	-2.95	3.12E-70
PI16	ENSG00000	1.08	14.31	-2.88	4.45E-16
KIAA0895	ENSG00000	3.19	21.22	-2.407	9.55E-46
FAM183B	ENSG00000	0.06	35.911	-5.122	2.36E-79
GPR85	ENSG00000	0.96	10.1	-2.502	6.05E-37
IQUB	ENSG00000	0.27	25.069	-4.359	5.11E-76
SLC13A4	ENSG00000	0.99	11.29	-2.627	4.38E-15
PGAM2	ENSG00000	1.08	254.813	-6.942	1.85E-69
SUN3	ENSG00000	3.89	46.311	-3.274	1.12E-33
SBSPON	ENSG00000	1.07	8.88	-2.255	5.99E-33
OXR1	ENSG00000	4.2	48.04	-3.237	1.98E-61
TMEM184	ENSG00000	0.24	7	-2.69	4.21E-27
GBX1	ENSG00000	0.07	9.06	-3.233	2.39E-83

OSR2	ENSG00000	1.22	41.639	-4.264	1.18E-40
FREM1	ENSG00000	0.6	8.37	-2.55	7.69E-12
TMEM67	ENSG00000	3.96	20.1	-2.089	4.78E-50
LETM2	ENSG00000	1.39	38.711	-4.054	3.12E-67
PRKACG	ENSG00000	0.15	32.119	-4.848	1.11E-69
PRSS37	ENSG00000	0.01	58.12	-5.871	3.79E-70
GKAP1	ENSG00000	11.2	188.562	-3.958	1.72E-51
KIF27	ENSG00000	2.25	26.969	-3.105	3.87E-72
SSMEM1	ENSG00000	0.03	31.48	-4.979	2.72E-68
RP11-213C	ENSG00000	1.4	9.81	-2.171	7.31E-66
TRPV6	ENSG00000	0.55	5.31	-2.025	8.02E-32
RNF183	ENSG00000	0.74	7.99	-2.369	1.91E-46
LRRC18	ENSG00000	0.02	17.01	-4.142	4.31E-60
PHYHIPL	ENSG00000	0.23	31.051	-4.704	3.06E-81
PKNOX2	ENSG00000	0.45	6.08	-2.288	1.46E-32
RPL10L	ENSG00000	6.11	119.361	-4.081	8.56E-40
TMEM63C	ENSG00000	0.97	15.47	-3.064	4.87E-72
NOXRED1	ENSG00000	0.47	11.13	-3.045	4.71E-65
SSX3	ENSG00000	0.09	16.89	-4.037	6.69E-86
VSTM4	ENSG00000	0.85	9.56	-2.513	4.33E-38
SOHLH1	ENSG00000	0.07	21.9	-4.42	2.61E-97
PDZD8	ENSG00000	6.15	58.732	-3.063	2.05E-39
GFI1B	ENSG00000	0.02	5.76	-2.728	6.56E-63
STK32C	ENSG00000	7.51	71.818	-3.097	9.75E-76
PPP1R36	ENSG00000	0.29	55.629	-5.456	2.93E-77
ERICH6B	ENSG00000	0.34	5.37	-2.249	2.09E-86
HSPA12A	ENSG00000	1.16	7.97	-2.054	2.84E-42
FAM35BP	ENSG00000	0.8	10.15	-2.631	3.47E-77
UBTD1	ENSG00000	16.09	76.962	-2.19	1.59E-51
SLC39A13	ENSG00000	13.72	109.873	-2.913	2.48E-76
IFI27L1	ENSG00000	17.67	140.857	-2.926	1.03E-92
PDZRN4	ENSG00000	0.07	3.36	-2.027	1.20E-26
PASD1	ENSG00000	0.3	15.14	-3.634	5.36E-52
TMCO5A	ENSG00000	0	149.582	-7.234	2.00E-75
RP11-299H	ENSG00000	0.09	4.63	-2.369	1.91E-56
SPATA19	ENSG00000	0	171.135	-7.427	8.94E-77
TBATA	ENSG00000	0.02	32.68	-5.045	1.57E-65
FAM227B	ENSG00000	4.1	19.929	-2.037	9.68E-54
POTED	ENSG00000	0.02	4.04	-2.305	1.48E-42
WDR88	ENSG00000	0.16	10.59	-3.321	1.06E-66
PPFIBP2	ENSG00000	12.02	58.06	-2.181	5.74E-41
RIC3	ENSG00000	3.51	20.53	-2.255	5.05E-67
XRRA1	ENSG00000	3.2	56.172	-3.767	2.80E-78
TRIM66	ENSG00000	1.42	34.251	-3.865	3.04E-92
TMEM130	ENSG00000	0.95	45.759	-4.584	7.75E-59
MFAP4	ENSG00000	6.54	32.43	-2.149	4.83E-04
TK2	ENSG00000	8	44.01	-2.322	3.29E-77
IQCD	ENSG00000	1.45	57.209	-4.57	4.01E-65
ZNF667-AS	ENSG00000	18.71	83.008	-2.092	3.47E-56

LDHC	ENSG00000	0.81	335.809	-7.54	1.39E-83
LDHAL6A	ENSG00000	0.24	7.77	-2.822	5.87E-50
PLIN1	ENSG00000	0.11	15.15	-3.863	1.12E-97
GPR182	ENSG00000	0.06	7.06	-2.927	6.04E-57
MIR202HG	ENSG00000	0.22	577.428	-8.889	1.33E-92
NYAP1	ENSG00000	1.68	12.5	-2.333	2.47E-70
MS4A6E	ENSG00000	0	125.078	-6.978	6.02E-75
MS4A14	ENSG00000	0.42	111.63	-6.31	2.51E-81
MS4A5	ENSG00000	0	59.659	-5.923	1.61E-67
EPB42	ENSG00000	0.11	7.21	-2.887	6.88E-85
EVA1C	ENSG00000	2.96	32.41	-3.077	5.13E-61
TCP10L2	ENSG00000	0.05	27.249	-4.75	2.17E-83
NKX3-1	ENSG00000	0.7	18.13	-3.492	1.35E-63
MEI1	ENSG00000	9.59	70.8	-2.761	7.58E-37
TTC16	ENSG00000	0.17	28.381	-4.65	5.59E-78
SUN5	ENSG00000	0	58.72	-5.9	9.77E-72
TBC1D21	ENSG00000	0	63.482	-6.011	1.19E-70
UGT1A6	ENSG00000	0.07	3.85	-2.18	8.39E-47
ISLR2	ENSG00000	0.38	22.7	-4.102	5.01E-47
GOLGA6C	ENSG00000	0.03	9.51	-3.351	1.36E-53
LOXHD1	ENSG00000	0.82	28.58	-4.023	8.76E-56
KATNAL2	ENSG00000	2.36	20.99	-2.71	1.90E-65
ZNF91	ENSG00000	12.19	67.4	-2.375	3.85E-54
RBFOX3	ENSG00000	1.64	10.93	-2.176	3.67E-30
PRRT2	ENSG00000	1.54	16.08	-2.749	2.64E-47
LINC00905	ENSG00000	0	34.999	-5.17	1.82E-89
GPX4	ENSG00000	283.419	1968.252	-2.792	1.70E-51
NOS2P2	ENSG00000	0	9.53	-3.396	3.23E-48
SPATA33	ENSG00000	6.39	190.916	-4.699	4.12E-72
PROCA1	ENSG00000	8.72	348.858	-5.17	3.24E-63
PROSER3	ENSG00000	8.24	41.619	-2.206	1.44E-43
TTYH1	ENSG00000	15.73	94.359	-2.511	1.76E-33
LENG8	ENSG00000	36.859	275.727	-2.87	8.05E-103
PPP1R14A	ENSG00000	20.92	100.141	-2.206	2.40E-40
GPT	ENSG00000	0.18	6.67	-2.7	5.89E-82
KIFC2	ENSG00000	5.97	51.069	-2.901	2.06E-87
SERPINF2	ENSG00000	1.58	14.84	-2.618	1.89E-23
KLK2	ENSG00000	1.22	38.63	-4.158	4.33E-61
KLK5	ENSG00000	0.73	18.52	-3.496	1.14E-31
KLK11	ENSG00000	0.15	28.499	-4.681	3.25E-56
TEKT1	ENSG00000	0.05	37.731	-5.205	2.70E-59
TMEM99	ENSG00000	11.03	139.14	-3.542	2.85E-76
RAB3IL1	ENSG00000	7.68	60.12	-2.816	4.52E-65
HRASLS5	ENSG00000	1.75	84.01	-4.95	4.62E-78
TTC21A	ENSG00000	3.11	141.847	-5.119	8.20E-96
ULK4	ENSG00000	4.1	26.259	-2.418	1.35E-45
HIST3H3	ENSG00000	0	3.48	-2.163	2.38E-58
KIF5C	ENSG00000	5.2	38.499	-2.671	3.73E-61
FAM107A	ENSG00000	0.86	7.05	-2.114	7.62E-14

MOBP	ENSG00000	0.23	16.28	-3.812	7.40E-78
LINC00917	ENSG00000	0.03	138.102	-7.077	8.22E-75
FILIP1L	ENSG00000	4.2	21.54	-2.116	1.55E-28
MFSD2A	ENSG00000	7.79	53.742	-2.639	8.20E-46
HR	ENSG00000	2.11	20.129	-2.764	6.91E-35
TXNDC2	ENSG00000	0.03	149.872	-7.195	7.77E-73
TNXB	ENSG00000	4.15	22.689	-2.202	2.29E-38
SFTPC	ENSG00000	0	5.07	-2.602	1.33E-49
PHYHIP	ENSG00000	0.46	12.63	-3.223	7.95E-67
GFRA2	ENSG00000	1.16	12.22	-2.614	3.39E-32
VWA3B	ENSG00000	0.36	85.342	-5.988	2.22E-66
LDLRAD4	ENSG00000	1.55	17.57	-2.864	7.70E-50
KCTD19	ENSG00000	0.52	79.522	-5.727	6.45E-85
TSPY2	ENSG00000	0.86	20.559	-3.535	6.04E-12
SHOX2	ENSG00000	0.45	5.58	-2.182	7.38E-24
PPIP5K1	ENSG00000	4.31	27.389	-2.419	8.56E-71
LRRC28	ENSG00000	10.1	50.059	-2.202	1.18E-47
SLC35G2	ENSG00000	1.09	18.79	-3.243	1.54E-94
FEM1B	ENSG00000	11.89	91.448	-2.842	5.03E-54
MFSD7	ENSG00000	4.34	28.34	-2.458	3.09E-34
VCX3A	ENSG00000	1.15	88.781	-5.384	7.18E-55
ZBBX	ENSG00000	0.8	51.239	-4.859	1.50E-62
GOT1L1	ENSG00000	0	18.539	-4.288	1.92E-58
IL13	ENSG00000	0.02	57.531	-5.843	5.15E-76
GCSAML	ENSG00000	0.06	3.76	-2.167	4.65E-83
THBS3	ENSG00000	8.33	101.567	-3.459	2.17E-68
SH3TC2	ENSG00000	0.67	11.52	-2.906	3.02E-85
SHE	ENSG00000	5.06	28.57	-2.287	1.58E-34
NR0B1	ENSG00000	0.6	23.6	-3.943	1.51E-84
PDILT	ENSG00000	0	22.59	-4.56	1.15E-65
RSPH10B2	ENSG00000	0.92	21.48	-3.549	2.24E-36
SCN9A	ENSG00000	0.14	4.44	-2.255	1.54E-77
GJB1	ENSG00000	0.33	13.67	-3.463	1.93E-26
RGPD8	ENSG00000	2.05	17.17	-2.575	1.35E-41
NRG4	ENSG00000	0.82	15.42	-3.173	1.65E-57
NLGN1	ENSG00000	0.41	6.28	-2.368	9.61E-46
TAPT1	ENSG00000	4.96	38.289	-2.721	1.66E-84
ONECUT1	ENSG00000	0.07	4.66	-2.403	1.03E-54
OTUD7A	ENSG00000	0.57	5.85	-2.125	2.49E-76
ZFPM2	ENSG00000	0.67	6.16	-2.1	1.58E-35
HSFY2	ENSG00000	0.01	15.65	-4.043	5.10E-52
FAM153A	ENSG00000	0.56	139.913	-6.497	8.23E-124
SIMC1	ENSG00000	3.15	37.919	-3.229	9.87E-71
HMP19	ENSG00000	0.08	19.231	-4.227	6.91E-65
USP50	ENSG00000	0.78	14.68	-3.139	1.39E-51
FAM161A	ENSG00000	3.94	19.08	-2.023	3.82E-40
FAXDC2	ENSG00000	3.32	18.78	-2.195	5.17E-39
STX8	ENSG00000	29.48	122.326	-2.017	3.60E-41
FRMPD2	ENSG00000	0.06	12.66	-3.688	1.10E-58

KRT86	ENSG0000(	0.09	5.18	-2.503	5.44E-54
SPATA24	ENSG0000(	4.95	188.535	-4.993	3.42E-66
NPAS2	ENSG0000(	2.34	23.659	-2.884	2.18E-58
KRT72	ENSG0000(	0.06	31.21	-4.925	4.62E-66
KISS1	ENSG0000(	0.49	5.82	-2.194	3.01E-21
FAM71B	ENSG0000(	0	39.62	-5.344	4.57E-64
TTLL6	ENSG0000(	0.99	37.229	-4.264	4.14E-66
RBMXL2	ENSG0000(	6.81	46.96	-2.618	5.19E-31
GPR37	ENSG0000(	1.73	13.66	-2.425	2.90E-38
TPD52L3	ENSG0000(	0	143.349	-7.173	3.68E-85
USP32	ENSG0000(	27.889	115.607	-2.013	1.76E-38
RNF139	ENSG0000(	16.04	81.701	-2.279	1.05E-55
PLA2G1B	ENSG0000(	0.1	5.22	-2.499	1.32E-59
TPT1-AS1	ENSG0000(	10.54	131.379	-3.52	2.22E-73
TANC2	ENSG0000(	3.6	22.389	-2.346	8.87E-53
NCBP2L	ENSG0000(	0	3.09	-2.032	5.09E-49
MBD3L1	ENSG0000(	0.03	35.009	-5.128	4.17E-63
PGK2	ENSG0000(	0	181.697	-7.513	1.90E-78
ZNF415	ENSG0000(	4.42	29.97	-2.514	3.36E-70
PRKCDBP	ENSG0000(	10.51	191.114	-4.061	2.06E-59
XKR6	ENSG0000(	1.49	9.54	-2.082	6.30E-65
OBP2B	ENSG0000(	0.03	13.62	-3.827	7.31E-73
FUT3	ENSG0000(	0.25	4.59	-2.161	2.01E-37
PRKCE	ENSG0000(	0.96	22.98	-3.613	2.55E-67
KRT15	ENSG0000(	0.41	25.221	-4.217	1.83E-24
LURAP1	ENSG0000(	1.5	10.98	-2.261	1.32E-58
KRT13	ENSG0000(	0.12	14.84	-3.822	6.11E-23
HOPX	ENSG0000(	6.11	59.149	-3.081	4.13E-50
SPACA5B	ENSG0000(	0	32.969	-5.086	8.98E-64
SPACA5	ENSG0000(	0.05	13.33	-3.771	1.92E-66
MROH2B	ENSG0000(	0.01	37.509	-5.253	5.29E-75
LPAR3	ENSG0000(	1.29	9.21	-2.157	1.77E-59
MAP6	ENSG0000(	2.12	23.9	-2.997	8.81E-53
LKAAEAR1	ENSG0000(	0.15	43.561	-5.276	2.34E-63
TCEA2	ENSG0000(	29.991	157.084	-2.351	8.17E-57
LRRC34	ENSG0000(	2.03	18.59	-2.693	1.89E-45
PAH	ENSG0000(	0.46	13.37	-3.299	4.95E-48
GATM	ENSG0000(	5.44	59.8	-3.239	2.34E-66
SYCE1	ENSG0000(	3.12	131.99	-5.013	3.30E-68
SLFNL1	ENSG0000(	0.29	97.937	-6.261	1.43E-77
WDR87	ENSG0000(	0.15	13.3	-3.636	1.98E-65
PWWP2B	ENSG0000(	3.56	22.299	-2.353	2.81E-58
FBXL14	ENSG0000(	12.7	55.441	-2.043	2.13E-30
TRAPPC12	ENSG0000(	53.809	248.395	-2.186	4.12E-51
PRND	ENSG0000(	0.69	74.899	-5.489	2.03E-67
KLF17	ENSG0000(	0.31	15.81	-3.682	5.38E-24
FBXW10	ENSG0000(	0.04	12.77	-3.727	7.77E-73
FOXB1	ENSG0000(	0.08	3.62	-2.097	7.64E-33
LRRC48	ENSG0000(	1.26	32.11	-3.873	1.03E-77

LDHAL6B	ENSG00000	0.03	36.111	-5.171	5.75E-89
TEX37	ENSG00000	0.6	106.498	-6.07	1.95E-52
SLC9C1	ENSG00000	0.02	4.21	-2.353	8.51E-59
FRMD3	ENSG00000	0.69	13.76	-3.127	7.45E-67
TPSAB1	ENSG00000	0.23	8.43	-2.939	7.31E-20
SERHL	ENSG00000	0.38	15.82	-3.607	1.18E-83
MACROD2	ENSG00000	1.04	7.61	-2.077	1.31E-66
SPTLC3	ENSG00000	0.36	4.55	-2.029	1.19E-32
FAM195A	ENSG00000	23.691	103.737	-2.085	2.49E-50
RSPH9	ENSG00000	1.01	20.29	-3.405	8.58E-59
PRSS30P	ENSG00000	0.57	19.87	-3.733	4.52E-87
TCEAL1	ENSG00000	5.07	30.279	-2.365	9.93E-36
HSFY1	ENSG00000	0	9.77	-3.429	3.22E-30
FAM170B	ENSG00000	0	7.01	-3.002	4.18E-48
SNTG2	ENSG00000	0.38	10.75	-3.09	3.48E-72
ZFAND4	ENSG00000	3.44	74.847	-4.094	6.52E-73
FAM71D	ENSG00000	0.1	41.12	-5.259	8.13E-68
LRRC20	ENSG00000	2.99	15.47	-2.045	2.64E-37
TMEM217	ENSG00000	0.74	22.63	-3.763	2.12E-83
SNX32	ENSG00000	0.54	10.07	-2.846	5.38E-85
OVOL1	ENSG00000	0.7	47.019	-4.82	1.19E-43
NBEA	ENSG00000	0.54	5.56	-2.091	4.19E-44
XKR3	ENSG00000	0.01	7.85	-3.131	1.44E-49
SH3RF3	ENSG00000	0.61	5.5	-2.013	6.87E-41
LRRN3	ENSG00000	0.51	8.93	-2.717	2.43E-38
PLAC8L1	ENSG00000	0.31	21.941	-4.13	2.43E-58
SLC2A14	ENSG00000	9.31	60.442	-2.575	1.50E-17
GPR137	ENSG00000	30.681	335.227	-3.408	1.15E-83
NDNF	ENSG00000	0.06	11.63	-3.575	5.13E-49
IQCF1	ENSG00000	0.49	147.604	-6.64	1.99E-59
GLIPR1L1	ENSG00000	0.03	37.431	-5.222	6.02E-60
THAP2	ENSG00000	1.94	18.65	-2.741	2.21E-62
RNASE11	ENSG00000	0	68.251	-6.114	4.34E-73
MST1	ENSG00000	11.9	115.239	-3.172	1.98E-76
PC	ENSG00000	5.14	41.55	-2.793	5.94E-66
LRFN4	ENSG00000	4.38	33.901	-2.698	6.93E-59
HSPB7	ENSG00000	0.58	28.779	-4.236	1.33E-63
TAS1R1	ENSG00000	0.21	5.3	-2.38	2.32E-68
SPATA3	ENSG00000	0	252.09	-7.984	1.71E-73
TIGD3	ENSG00000	0.27	6.3	-2.523	1.06E-80
10-Mar	ENSG00000	0.2	216.781	-7.504	7.61E-87
PHOSPHO1	ENSG00000	0.81	119.51	-6.057	4.16E-59
SPTBN2	ENSG00000	6.27	138.533	-4.263	2.58E-52
3-Mar	ENSG00000	2.39	34.589	-3.392	1.62E-80
LRRC63	ENSG00000	0.29	4.71	-2.146	6.76E-49
SPERT	ENSG00000	0.06	122.742	-6.867	1.86E-72
FAM174A	ENSG00000	6.98	36.259	-2.223	3.25E-84
FAM53A	ENSG00000	2.59	26.9	-2.958	1.06E-54
GSTA3	ENSG00000	0.86	9.25	-2.462	7.27E-33

SNX31	ENSG0000(	0.19	4.25	-2.141	5.85E-19
ZNHIT2	ENSG0000(	9.72	86.138	-3.023	6.24E-60
EVX2	ENSG0000(	0.01	3.8	-2.249	1.57E-44
ZHX3	ENSG0000(	4.5	40.481	-2.915	8.29E-54
PODN	ENSG0000(	3.26	33.581	-3.021	5.57E-29
STAG3L3	ENSG0000(	10.82	78.809	-2.755	1.73E-64
RP11-313F	ENSG0000(	6.41	31.97	-2.154	1.38E-70
GTF2IRD2B	ENSG0000(	4.1	26.07	-2.408	3.54E-41
STARD6	ENSG0000(	0.06	19.74	-4.29	1.05E-72
GOLGA6L2	ENSG0000(	0	52.45	-5.74	1.20E-66
KLK15	ENSG0000(	0.09	4.73	-2.394	1.25E-79
IL20RB	ENSG0000(	1.18	10.44	-2.392	2.89E-30
IQCK	ENSG0000(	4.06	42.101	-3.091	6.97E-64
PCP2	ENSG0000(	0.62	169.377	-6.717	7.61E-75
PTDSS2	ENSG0000(	18.23	107.418	-2.495	4.78E-62
KCTD13	ENSG0000(	12.22	104.43	-2.995	9.75E-99
TEX36	ENSG0000(	0	29.03	-4.908	1.47E-60
ZDHHC14	ENSG0000(	3.04	16.8	-2.14	1.62E-53
FAM182B	ENSG0000(	1.47	35.221	-3.874	7.05E-47
PPM1E	ENSG0000(	0.58	7.39	-2.409	1.37E-53
HIGD2B	ENSG0000(	0.04	4.76	-2.47	9.88E-64
NPPA	ENSG0000(	0.36	4.7	-2.067	1.67E-34
GOLGA8A	ENSG0000(	26.08	118.668	-2.144	2.17E-30
VWA3A	ENSG0000(	0.06	12.46	-3.666	2.53E-57
PHYKPL	ENSG0000(	13.28	96.857	-2.777	1.48E-88
NRIP3	ENSG0000(	3.92	47.751	-3.309	2.21E-52
RFESD	ENSG0000(	2.74	17.259	-2.288	7.21E-50
TSGA10IP	ENSG0000(	1.29	15.47	-2.846	2.51E-35
UBQLNL	ENSG0000(	0.23	37.959	-4.985	1.95E-72
UBQLN3	ENSG0000(	0.02	205.101	-7.659	1.53E-74
UCP3	ENSG0000(	0.65	7.19	-2.311	1.96E-83
PRM1	ENSG0000(	0.1	17993.65	-13.998	7.84E-77
TOM1L2	ENSG0000(	7.41	56.199	-2.766	5.42E-82
TEX26	ENSG0000(	0	42.541	-5.444	1.39E-72
GOLGA8EP	ENSG0000(	0	11.35	-3.626	3.61E-64
LINC00521	ENSG0000(	0	28.879	-4.901	8.05E-70
TTLL11	ENSG0000(	1.44	18.27	-2.981	9.25E-73
RP11-121M	ENSG0000(	0.28	13.9	-3.541	7.54E-58
ZNF645	ENSG0000(	0	10.71	-3.55	8.33E-62
SWI5	ENSG0000(	23.33	203.459	-3.071	3.40E-62
WBSCR28	ENSG0000(	0	126.956	-7	1.67E-72
HOXD8	ENSG0000(	1.3	23.95	-3.439	5.81E-95
ZNF683	ENSG0000(	1.27	105.441	-5.551	1.02E-54
HSF5	ENSG0000(	0.15	31.139	-4.805	6.55E-81
FOXG1	ENSG0000(	0.2	5.06	-2.336	3.50E-58
ENTHD1	ENSG0000(	0.08	8.4	-3.122	5.93E-93
HMGB4	ENSG0000(	0.02	1033.913	-9.987	1.46E-82
SLCO3A1	ENSG0000(	6.85	62.141	-3.008	6.85E-78
PLA2G16	ENSG0000(	21.08	174.332	-2.989	3.65E-46

GNG7	ENSG0000(	1.46	11.18	-2.308	3.40E-62
MAP3K19	ENSG0000(	0.3	14.28	-3.555	2.35E-35
MEX3C	ENSG0000(	17.94	145.431	-2.951	2.96E-49
HORMAD2	ENSG0000(	0.09	24.591	-4.553	5.02E-88
TGIF2LY	ENSG0000(	0	4.92	-2.566	1.30E-40
PFN4	ENSG0000(	0.33	133.796	-6.663	8.38E-88
MAGEB6	ENSG0000(	0.24	4.12	-2.046	7.33E-45
LRRC37A3	ENSG0000(	3.55	36.77	-3.053	3.34E-75
MIR7-3HG	ENSG0000(	0	18.03	-4.25	1.97E-63
MAMSTR	ENSG0000(	1.61	11.66	-2.278	7.82E-44
MUC20	ENSG0000(	1.45	13.75	-2.59	9.21E-21
FMR1NB	ENSG0000(	0.1	76.818	-6.145	4.31E-85
TMEM80	ENSG0000(	6.9	47.871	-2.629	4.03E-90
MRVI1-AS:	ENSG0000(	0.13	216.405	-7.588	3.61E-87
LINC00982	ENSG0000(	0.55	42.579	-4.813	9.39E-70
FAM9B	ENSG0000(	0.36	8.08	-2.739	2.33E-44
SPACA4	ENSG0000(	0.06	161.064	-7.256	8.97E-77
FBXO39	ENSG0000(	0.22	39.24	-5.044	2.05E-66
ZBTB38	ENSG0000(	5.02	25.43	-2.134	1.98E-44
OVOS2	ENSG0000(	19.73	134.969	-2.713	3.28E-22
FLJ40194	ENSG0000(	0.01	52.928	-5.739	5.01E-66
UBE2U	ENSG0000(	0.15	45.591	-5.34	3.08E-65
RBM44	ENSG0000(	0.16	13.96	-3.689	2.88E-89
VCX2	ENSG0000(	0.05	24.35	-4.594	3.20E-62
FAM187B	ENSG0000(	0.02	87.869	-6.445	8.20E-72
PVRL3	ENSG0000(	1.59	36.491	-3.856	3.65E-69
SPATA31C:	ENSG0000(	0.11	16.47	-3.976	1.56E-63
MAMDC4	ENSG0000(	3.39	17.09	-2.043	2.57E-41
ODF3	ENSG0000(	0.01	11.84	-3.668	6.00E-56
SPATA31E:	ENSG0000(	0.09	9.27	-3.236	3.50E-47
TSPYL6	ENSG0000(	0.38	20.53	-3.964	2.79E-51
MLF1	ENSG0000(	16.979	527.928	-4.879	5.48E-67
NDUFAF3	ENSG0000(	52.508	487.513	-3.191	3.96E-66
GRAMD1C	ENSG0000(	1.79	20.01	-2.913	3.42E-62
TSSK6	ENSG0000(	1.01	267.333	-7.061	7.66E-74
GOLGA8Q	ENSG0000(	0.14	8.91	-3.12	7.57E-65
PPP1R42	ENSG0000(	0.08	32.981	-4.976	3.78E-77
FAR2P2	ENSG0000(	8.01	62.9	-2.826	9.03E-55
RNF212	ENSG0000(	1.18	23.19	-3.472	1.98E-66
PRM3	ENSG0000(	0	101.948	-6.686	1.70E-72
TNP2	ENSG0000(	0	51.041	-5.702	5.14E-57
TMPRSS9	ENSG0000(	0.2	7.75	-2.866	4.18E-80
FAM220A	ENSG0000(	17.85	95.862	-2.361	1.19E-40
MCMDC2	ENSG0000(	0.42	9.92	-2.943	4.11E-76
P4HTM	ENSG0000(	5.18	41.969	-2.798	1.34E-61
KLHL11	ENSG0000(	1.76	12.14	-2.251	2.51E-30
PSAPL1	ENSG0000(	0.02	5.93	-2.764	4.07E-53
PARP10	ENSG0000(	16.3	69.632	-2.03	2.15E-42
THBD	ENSG0000(	1.64	9.75	-2.026	6.36E-25

FAM132B	ENSG00000	1.46	22.899	-3.28	3.18E-47
GDPD4	ENSG00000	1.28	24.25	-3.469	8.16E-32
RIIAD1	ENSG00000	0.7	22.83	-3.809	4.41E-56
H1FOO	ENSG00000	0.04	9.26	-3.302	1.59E-55
TRIM73	ENSG00000	0.81	23.831	-3.778	9.05E-119
OPLAH	ENSG00000	2.88	93.428	-4.605	2.02E-73
TMEM52	ENSG00000	2.7	42.929	-3.57	1.09E-71
ERICH3	ENSG00000	0.04	46.391	-5.51	2.25E-73
EXD1	ENSG00000	0.16	29.941	-4.737	5.85E-88
PER1	ENSG00000	12.42	72.71	-2.457	1.36E-63
GGN	ENSG00000	0.68	153.958	-6.527	5.84E-75
SIGLECL1	ENSG00000	0.07	26.79	-4.699	6.58E-69
NSUN7	ENSG00000	1.59	14.3	-2.562	3.11E-46
WSCD1	ENSG00000	2.42	25.01	-2.927	1.75E-36
TMEM31	ENSG00000	1.07	136.361	-6.052	2.07E-59
GPC5	ENSG00000	0.07	3.33	-2.017	1.75E-67
LINC00174	ENSG00000	5.93	32.251	-2.262	1.20E-39
SHARPIN	ENSG00000	54.36	266.039	-2.27	2.07E-60
LSMEM2	ENSG00000	0.3	4.62	-2.112	3.84E-58
RNF151	ENSG00000	0.1	260.293	-7.892	1.70E-76
PLD6	ENSG00000	2.98	27.851	-2.858	7.09E-71
GRM8	ENSG00000	0.22	8.74	-2.997	1.08E-46
TPPP2	ENSG00000	0.08	440.225	-8.674	5.53E-76
LINC00305	ENSG00000	0.05	6.4	-2.817	8.57E-49
KIAA1875	ENSG00000	0.71	31.169	-4.234	9.45E-80
SYCN	ENSG00000	0	35.36	-5.184	4.59E-88
LRRC3B	ENSG00000	0.07	6.49	-2.807	7.47E-44
PCBP1-AS1	ENSG00000	12.39	56.551	-2.104	6.59E-35
PDXDC1	ENSG00000	29.761	152.249	-2.317	1.26E-49
NRXN1	ENSG00000	0.38	8.53	-2.788	5.19E-43
GOLGA8J	ENSG00000	0.04	6.3	-2.811	3.24E-67
RP11-1319	ENSG00000	0.55	9.91	-2.815	2.27E-63
FAM71E2	ENSG00000	0	45.241	-5.531	8.87E-68
WFDC11	ENSG00000	0	4.73	-2.518	2.96E-54
EXOC3	ENSG00000	15.64	85.241	-2.374	2.13E-79
TDRD6	ENSG00000	1.46	40.07	-4.061	5.89E-84
FAR2P1	ENSG00000	0.74	91.448	-5.732	6.46E-48
FAM71C	ENSG00000	0	35.25	-5.18	3.74E-69
PRNT	ENSG00000	0	9.73	-3.424	8.34E-42
MGC45922	ENSG00000	0.3	11.79	-3.298	1.42E-85
HARBI1	ENSG00000	2.79	14.18	-2.002	2.12E-44
GLIPR1L2	ENSG00000	0.16	30.67	-4.771	9.89E-87
TSPYL5	ENSG00000	7.43	46.639	-2.499	3.68E-49
PIPSL	ENSG00000	0.25	14.84	-3.664	1.69E-61
LSMEM1	ENSG00000	0.56	7.52	-2.449	1.78E-79
MAPK15	ENSG00000	1.55	71.619	-4.832	3.00E-69
RP11-429J	ENSG00000	0.41	6.89	-2.484	3.02E-74
HLA-V	ENSG00000	0.07	5.02	-2.492	8.61E-62
PJA1	ENSG00000	7.04	33.079	-2.084	2.64E-18

PENK	ENSG0000(	1.23	175.095	-6.303	8.36E-72
SLC25A41	ENSG0000(	0.21	14.44	-3.674	8.45E-71
NME9	ENSG0000(	0.44	7.91	-2.629	2.17E-67
SPEM1	ENSG0000(	0.19	90.49	-6.265	1.96E-69
SAGE1	ENSG0000(	0.01	4.29	-2.389	5.65E-82
MEX3D	ENSG0000(	16.64	107.187	-2.617	1.01E-38
GPR135	ENSG0000(	0.16	28.929	-4.689	6.27E-84
ZBTB20	ENSG0000(	0.22	21.5	-4.205	4.74E-79
ODF3L2	ENSG0000(	0.2	66.712	-5.818	8.24E-66
FTMT	ENSG0000(	0.23	71.491	-5.881	2.11E-67
GOLGA8CP	ENSG0000(	0	18.67	-4.298	2.01E-74
LINC00301	ENSG0000(	0	112.29	-6.824	6.85E-81
MGAT4C	ENSG0000(	0.1	7.26	-2.909	5.91E-64
RP11-69E1	ENSG0000(	5.12	26.13	-2.148	1.07E-45
FAM153B	ENSG0000(	0.69	215.209	-6.999	4.86E-146
GABRG3	ENSG0000(	0.1	9.89	-3.307	7.69E-58
IZUMO1	ENSG0000(	0.14	44.941	-5.333	1.11E-74
SPACA6P	ENSG0000(	5.75	54.409	-3.037	6.05E-86
YBEY	ENSG0000(	20.54	105.419	-2.305	5.42E-36
TEX19	ENSG0000(	0.33	36.839	-4.83	3.99E-77
VCX	ENSG0000(	1.11	59.56	-4.843	9.46E-45
PPP1R27	ENSG0000(	0.1	6.27	-2.724	9.00E-55
RGS6	ENSG0000(	0.24	15.38	-3.724	9.33E-60
PAQR7	ENSG0000(	5.73	61.75	-3.221	8.26E-94
MAFA	ENSG0000(	0.41	4.93	-2.072	1.36E-22
GRID1	ENSG0000(	0.45	5.78	-2.225	9.44E-75
TMEM198I	ENSG0000(	10.33	64.498	-2.531	5.25E-91
FUNDC2P2	ENSG0000(	0	60.93	-5.953	1.93E-66
RP11-181(	ENSG0000(	1.7	11.11	-2.165	4.86E-60
GLUD2	ENSG0000(	1.71	19.821	-2.942	1.08E-53
TMEM95	ENSG0000(	0	13.21	-3.829	8.69E-65
WFDC10B	ENSG0000(	0.06	3.5	-2.086	4.08E-48
ODF3L1	ENSG0000(	0.09	30.149	-4.837	6.87E-61
OTOP2	ENSG0000(	0.02	5.61	-2.696	9.74E-54
SLC25A10	ENSG0000(	11.3	51.172	-2.085	5.98E-39
RGPD6	ENSG0000(	10.08	51.52	-2.245	1.35E-61
WBP2NL	ENSG0000(	0.46	110.913	-6.26	1.31E-84
GAS6	ENSG0000(	23.3	136.153	-2.497	1.61E-54
POM121L1	ENSG0000(	0	8.33	-3.222	8.12E-55
POTEC	ENSG0000(	0.03	7.44	-3.035	3.37E-64
WT1-AS	ENSG0000(	0.21	6.86	-2.7	5.98E-82
RIMBP3C	ENSG0000(	0.09	14.21	-3.803	1.84E-47
TISP43	ENSG0000(	0	13.22	-3.83	5.28E-63
FAM9A	ENSG0000(	0.05	5.51	-2.632	1.01E-79
EPHA10	ENSG0000(	1.34	41.619	-4.187	2.20E-55
SPDYE4	ENSG0000(	0	10.1	-3.472	3.61E-71
REC114	ENSG0000(	0.6	51.749	-5.043	2.13E-66
JRKL	ENSG0000(	2.4	15.48	-2.277	8.89E-55
TMEM89	ENSG0000(	0.1	30.92	-4.859	1.69E-64

NPIPA1	ENSG0000(	54.511	251.863	-2.188	7.39E-79
TFDP3	ENSG0000(	0.16	5.33	-2.448	3.08E-28
RP11-958N	ENSG0000(	3.39	20.29	-2.278	1.40E-37
MEX3B	ENSG0000(	4.43	61.991	-3.536	3.15E-57
FAM46C	ENSG0000(	6.77	52.109	-2.773	3.66E-23
PCBP3	ENSG0000(	2.86	27.57	-2.888	1.22E-64
GOLGA8G	ENSG0000(	0	37.509	-5.267	5.02E-78
TP53TG3	ENSG0000(	0.6	8.97	-2.64	1.91E-46
11-Mar	ENSG0000(	0.47	130.119	-6.479	3.00E-87
OR4N4	ENSG0000(	0	7.71	-3.123	1.21E-45
TUBA8	ENSG0000(	0.98	67.578	-5.114	4.34E-73
NPIPA5	ENSG0000(	14.1	62.882	-2.081	1.55E-26
MAATS1	ENSG0000(	0.91	52.921	-4.819	4.24E-91
PNMA3	ENSG0000(	1.08	22.279	-3.484	6.40E-67
FAM3B	ENSG0000(	1.07	15.8	-3.021	1.14E-29
NPIPA5	ENSG0000(	74.682	308.686	-2.033	1.53E-51
PP2D1	ENSG0000(	0.1	16.97	-4.03	5.60E-59
TRIML1	ENSG0000(	0	16.979	-4.168	2.87E-58
RP13-1039	ENSG0000(	0.05	20.129	-4.331	2.29E-60
RPL7AP28	ENSG0000(	0.14	9.02	-3.136	1.23E-52
SPRR4	ENSG0000(	0	7.54	-3.094	1.73E-38
LRTOMT	ENSG0000(	4.52	171.586	-4.967	6.97E-82
TSPYL2	ENSG0000(	20.8	256.532	-3.562	7.00E-110
GOLGA6L4	ENSG0000(	1.66	16.32	-2.703	5.52E-91
PGP	ENSG0000(	14.72	68.688	-2.148	4.57E-52
OLIG1	ENSG0000(	0.21	4.01	-2.05	2.41E-41
PRKD1	ENSG0000(	0.9	17.04	-3.247	7.24E-57
MROH7	ENSG0000(	0.44	126.027	-6.463	6.66E-100
IQCF2	ENSG0000(	0	113.59	-6.84	2.02E-71
SPATA32	ENSG0000(	0.06	65.471	-5.971	3.55E-74
PLA2G6	ENSG0000(	11.95	84.021	-2.715	2.13E-62
NUTM1	ENSG0000(	0.39	25.77	-4.267	6.61E-68
FAM19A3	ENSG0000(	0.52	10.2	-2.881	3.12E-54
PRSS55	ENSG0000(	0	39.281	-5.332	2.07E-66
ODF4	ENSG0000(	0	18.5	-4.285	4.48E-71
SERINC4	ENSG0000(	0.12	4.73	-2.355	3.89E-96
FAM110C	ENSG0000(	0.26	4.06	-2.006	3.83E-55
SMIM10	ENSG0000(	1.28	29.619	-3.747	9.56E-70
TCTE3	ENSG0000(	1.4	26.67	-3.527	5.24E-83
OSBP2	ENSG0000(	2.11	41.81	-3.783	5.14E-47
ZBTB7C	ENSG0000(	0.63	6.56	-2.214	9.83E-62
TCEAL2	ENSG0000(	1.69	52.98	-4.327	2.44E-59
LCN12	ENSG0000(	0.33	26.829	-4.387	2.44E-95
WT1	ENSG0000(	1	32.76	-4.077	1.24E-90
FAM227A	ENSG0000(	0.9	16.381	-3.193	1.36E-63
NT5C1B	ENSG0000(	0.16	51.371	-5.497	1.91E-69
MROH2A	ENSG0000(	0.06	6.38	-2.8	2.96E-77
FLRT2	ENSG0000(	2.64	14.28	-2.07	1.86E-07
MFSD6L	ENSG0000(	0.36	33.599	-4.669	1.98E-53

GOLGA8DF	ENSG00000	0	11.63	-3.659	3.16E-64
LINC00313	ENSG00000	0.25	4.95	-2.251	2.64E-87
NRBP2	ENSG00000	7.02	34.84	-2.16	7.07E-48
MAGEA11	ENSG00000	0.25	6.38	-2.562	1.75E-61
PPIL6	ENSG00000	1.14	30.721	-3.89	1.15E-68
TEX33	ENSG00000	0	123.956	-6.965	4.92E-72
NUPR1L	ENSG00000	1.24	443.779	-7.633	2.70E-69
SPPL2C	ENSG00000	0	48.65	-5.634	2.47E-68
RGPD2	ENSG00000	6.36	29.09	-2.031	7.65E-32
PARK2	ENSG00000	0.55	5.73	-2.118	1.70E-76
LINC00158	ENSG00000	0.08	6.1	-2.717	1.85E-54
MUC1	ENSG00000	2.57	72.821	-4.37	2.32E-57
LMNTD2	ENSG00000	1.26	42.611	-4.27	8.67E-77
SPATA45	ENSG00000	0.14	12.88	-3.606	6.22E-57
SPATA8	ENSG00000	0.08	497.136	-8.849	7.32E-91
LMLN	ENSG00000	2.01	14.67	-2.38	2.34E-79
SHC4	ENSG00000	0.23	9.06	-3.032	8.14E-90
SMIM23	ENSG00000	0	35.75	-5.2	1.93E-62
MORN5	ENSG00000	0.16	111.538	-6.6	7.61E-72
EP400NL	ENSG00000	6.48	69.859	-3.244	6.03E-92
WDR53	ENSG00000	20.559	91.689	-2.104	2.21E-30
PCYT2	ENSG00000	16.33	358.218	-4.374	3.88E-63
NAT8L	ENSG00000	1.13	19.92	-3.296	3.36E-73
TMEM210	ENSG00000	0.06	111.46	-6.729	2.24E-76
NPIPB4	ENSG00000	29.29	124.154	-2.047	2.64E-31
TRIM69	ENSG00000	9.67	46.471	-2.153	5.75E-36
PRSS38	ENSG00000	0	5.01	-2.587	4.67E-68
FAM186A	ENSG00000	0.14	22.05	-4.338	1.37E-58
PLK5	ENSG00000	0.58	9.16	-2.685	3.06E-71
LEMD1	ENSG00000	1.52	111.136	-5.476	4.22E-69
ZPBP2	ENSG00000	0.04	103.73	-6.654	2.10E-93
PIP5K1C	ENSG00000	14.52	82.681	-2.431	4.59E-63
TEX38	ENSG00000	0.21	522.142	-8.756	9.72E-81
PRR30	ENSG00000	0	197.566	-7.633	1.14E-75
UBL4B	ENSG00000	0.07	137.443	-7.016	5.79E-75
SLC51B	ENSG00000	0.09	12.45	-3.625	9.81E-67
KDM4D	ENSG00000	1.37	32.999	-3.843	9.32E-62
GPAT2	ENSG00000	1.89	22.011	-2.993	9.94E-50
MST1P2	ENSG00000	0.82	22.99	-3.72	2.19E-77
SLC36A3	ENSG00000	0	7.5	-3.087	2.01E-54
LINC00643	ENSG00000	0.08	41.489	-5.298	3.35E-67
GOLGA8R	ENSG00000	0.42	9.56	-2.895	1.24E-70
TRDN	ENSG00000	0.24	5.8	-2.455	1.32E-41
SPATA12	ENSG00000	0.13	25.24	-4.537	9.04E-63
TMPRSS12	ENSG00000	0	22.011	-4.524	2.76E-72
FAM228A	ENSG00000	0.4	55.291	-5.329	4.15E-91
GNG2	ENSG00000	11.04	153.553	-3.682	1.00E-56
MYT1L	ENSG00000	0.16	6.63	-2.718	1.25E-63
ZNF396	ENSG00000	0.92	6.94	-2.048	9.31E-71

SPATC1	ENSG00000	0.15	50.73	-5.491	1.55E-70
KIF24	ENSG00000	1.65	12.23	-2.32	3.52E-52
ZFP91	ENSG00000	11.01	71.051	-2.585	1.36E-64
MST1L	ENSG00000	1.9	26.07	-3.223	1.28E-51
MPPED1	ENSG00000	0.09	7.21	-2.913	6.84E-52
SPATA31D	ENSG00000	0	10.08	-3.47	3.32E-66
HYAL3	ENSG00000	2.62	17.771	-2.374	7.54E-46
SELV	ENSG00000	1.59	22.36	-3.173	2.90E-42
TRABD2A	ENSG00000	2.3	59.301	-4.192	2.36E-47
MAPT	ENSG00000	0.71	41.639	-4.64	2.19E-47
TMEM232	ENSG00000	0.59	18.41	-3.61	7.25E-76
LINC01551	ENSG00000	0	8.77	-3.288	2.84E-61
FAM183A	ENSG00000	0.94	17.321	-3.239	1.26E-35
ESPN	ENSG00000	4.87	198.335	-5.086	7.64E-73
TMPRSS6	ENSG00000	0.25	9.9	-3.124	4.63E-51
TMEM262	ENSG00000	0.4	28.789	-4.411	1.40E-82
PLCD1	ENSG00000	10.02	76.85	-2.821	3.01E-50
HEATR4	ENSG00000	0.31	15.41	-3.647	2.20E-74
LYPD6	ENSG00000	0.68	7.58	-2.353	1.69E-24
SPATA21	ENSG00000	0.05	12.41	-3.675	4.86E-66
H1FNT	ENSG00000	0.06	284.818	-8.075	9.89E-86
RP11-195F	ENSG00000	4.96	332.819	-5.808	8.50E-79
ZNF546	ENSG00000	1.13	17.06	-3.084	1.49E-86
HYPM	ENSG00000	0.08	53.16	-5.648	2.11E-66
IFT140	ENSG00000	4.73	43.081	-2.944	6.45E-69
POTEG	ENSG00000	0.01	3.13	-2.032	1.36E-57
ZNF385C	ENSG00000	1.81	22.139	-3.042	1.39E-64
TMEM247	ENSG00000	0.57	65.748	-5.41	1.15E-62
RGPD1	ENSG00000	10.69	66.879	-2.538	2.85E-31
KRT18P59	ENSG00000	0.03	4.84	-2.503	1.00E-70
RP11-723C	ENSG00000	0.18	5.92	-2.552	2.06E-53
FAM69C	ENSG00000	0.16	7.13	-2.809	1.62E-44
TMEM72	ENSG00000	0.03	3.76	-2.208	4.72E-38
FAM205C	ENSG00000	0	34.539	-5.151	3.97E-66
TMEM202	ENSG00000	0	25.06	-4.704	2.50E-59
RP11-24M	ENSG00000	0	6.06	-2.82	4.67E-54
TMEM220	ENSG00000	5.56	32.86	-2.368	9.18E-55
LRRC74B	ENSG00000	0.08	14.95	-3.884	8.53E-78
LCN10	ENSG00000	0.96	10.63	-2.569	8.91E-44
ZCCHC13	ENSG00000	0.04	28.201	-4.811	2.03E-60
TPRG1	ENSG00000	1.06	9.82	-2.393	1.90E-82
RP11-43F1	ENSG00000	2.63	19.32	-2.485	5.43E-61
MORN2	ENSG00000	15.46	223.417	-3.769	4.71E-59
RNF133	ENSG00000	0.02	55.572	-5.793	3.01E-72
TMEM215	ENSG00000	0.12	4.56	-2.312	8.24E-45
FAM166A	ENSG00000	0.18	84.858	-6.185	3.61E-87
LINC00265	ENSG00000	5.19	50.181	-3.048	1.07E-56
FAM230A	ENSG00000	0	9.05	-3.329	6.34E-59
LRRIQ4	ENSG00000	0.04	3.43	-2.091	3.58E-45

FAM92A1	ENSG00000	29.769	157.204	-2.362	2.76E-47
PRR19	ENSG00000	5.14	29.171	-2.297	6.60E-38
H3F3C	ENSG00000	0.2	26.999	-4.544	1.32E-68
PPP3R2	ENSG00000	0.01	47.029	-5.571	1.04E-75
GOLGA6L3	ENSG00000	0.08	11.82	-3.569	1.61E-81
NANOS2	ENSG00000	0.06	4.57	-2.394	4.54E-68
SERPINA5	ENSG00000	7.31	416.363	-5.65	1.18E-72
KRTDAP	ENSG00000	0.07	14.27	-3.835	1.72E-36
HBA2	ENSG00000	11.21	67.102	-2.48	8.60E-04
PAQR9	ENSG00000	0.14	26.09	-4.571	6.32E-58
GOLGA8M	ENSG00000	0.2	8.09	-2.921	1.21E-71
SAXO2	ENSG00000	1.76	32.54	-3.603	1.22E-65
HILS1	ENSG00000	0.09	25.479	-4.602	2.39E-68
TEKT4P2	ENSG00000	2.33	12.59	-2.029	2.24E-29
SLC4A5	ENSG00000	1.18	14.61	-2.84	2.46E-66
FSIP2	ENSG00000	0.32	25.871	-4.347	9.80E-71
TBC1D3P2	ENSG00000	0.02	14.35	-3.912	1.64E-69
TMCO2	ENSG00000	0	158.034	-7.313	1.92E-76
ZDHHC11	ENSG00000	3.22	28.041	-2.783	6.70E-56
ZNF563	ENSG00000	1.98	15.26	-2.448	2.93E-70
FBF1	ENSG00000	6.77	60.572	-2.986	3.06E-85
USP32P1	ENSG00000	15.95	132.357	-2.976	8.72E-34
UTS2B	ENSG00000	0.24	5.28	-2.34	9.29E-41
MSANTD1	ENSG00000	0.27	4.71	-2.169	2.47E-71
RNFT1	ENSG00000	6.84	58.048	-2.913	1.30E-68
GAGE2A	ENSG00000	0.61	62.53	-5.302	1.19E-59
TMEM120A	ENSG00000	35.231	252.387	-2.806	7.79E-89
PLAC9	ENSG00000	3.35	19.16	-2.212	1.92E-09
FAM47B	ENSG00000	0	11.95	-3.695	4.68E-66
NKAPL	ENSG00000	0.49	39.01	-4.747	1.00E-94
UBE2Q2P1	ENSG00000	1.44	10.79	-2.273	6.91E-50
FSCB	ENSG00000	0.01	39.871	-5.339	6.98E-63
FAM47E	ENSG00000	0.84	12.56	-2.882	5.04E-73
LINC00943	ENSG00000	0.03	84.87	-6.381	2.60E-73
SPANXN3	ENSG00000	0	55.291	-5.815	3.75E-79
FAM150B	ENSG00000	1.3	40.571	-4.176	9.87E-96
SPANXN4	ENSG00000	0	28.22	-4.869	7.05E-67
RP11-113D	ENSG00000	0	19.66	-4.369	1.16E-70
FAM179A	ENSG00000	1.03	7.55	-2.074	1.76E-34
SPATA31D	ENSG00000	0	6.71	-2.947	1.16E-51
KIAA0408	ENSG00000	0.37	5.22	-2.183	1.78E-43
TBC1D28	ENSG00000	0	32.701	-5.075	2.95E-71
OTUD6A	ENSG00000	0.21	5.13	-2.341	3.15E-16
MMP23B	ENSG00000	3.7	20.53	-2.196	2.76E-21
SPATA41	ENSG00000	0.25	6.96	-2.671	3.96E-87
USP32P3	ENSG00000	4.65	60.501	-3.444	2.20E-31
SYCP2	ENSG00000	1.75	40.461	-3.914	1.53E-58
MYBPC1	ENSG00000	0.07	6.81	-2.868	2.71E-58
KIAA0895L	ENSG00000	11.3	65.689	-2.439	3.44E-78

VN1R2	ENSG0000(	0.02	4.06	-2.311	8.78E-44
PLEKHG4	ENSG0000(	12.63	97.423	-2.852	7.88E-43
STK40	ENSG0000(	16.45	73.449	-2.093	7.42E-77
OR10J1	ENSG0000(	0	8.5	-3.248	3.89E-47
SRGAP3	ENSG0000(	1.14	10.56	-2.433	4.89E-57
RP11-497H	ENSG0000(	1.07	10.64	-2.491	9.95E-27
ZBTB44	ENSG0000(	8.83	44.359	-2.206	4.74E-57
STK31	ENSG0000(	2.75	60.77	-4.042	8.96E-55
NLGN3	ENSG0000(	0.3	5.41	-2.302	1.46E-39
ZNF781	ENSG0000(	2.75	15.51	-2.138	2.40E-33
INCA1	ENSG0000(	1.58	65.181	-4.681	2.75E-91
SPANXD	ENSG0000(	0.04	26.8	-4.74	2.95E-62
NOXO1	ENSG0000(	0.48	7.55	-2.53	1.84E-67
NBPF4	ENSG0000(	0.02	4.01	-2.296	1.11E-62
NPIPB15	ENSG0000(	3.74	74.227	-3.988	2.76E-54
GK2	ENSG0000(	0.01	75.583	-6.245	9.85E-80
TCEAL3	ENSG0000(	7.17	43.099	-2.432	2.26E-30
LINC00238	ENSG0000(	0	107.761	-6.765	1.02E-70
PFN3	ENSG0000(	0.08	30.93	-4.886	3.17E-61
ZNF846	ENSG0000(	3.34	46.649	-3.457	1.52E-96
PDXDC2P	ENSG0000(	5.49	33.62	-2.415	8.21E-60
TOMM20L	ENSG0000(	0.06	17.8	-4.149	1.18E-69
RGPD4	ENSG0000(	0.01	4.42	-2.424	3.09E-68
KIAA1211L	ENSG0000(	1.12	10.18	-2.399	1.42E-63
TEX43	ENSG0000(	0	27.249	-4.82	2.47E-66
KPNA5	ENSG0000(	2.35	43.09	-3.718	1.84E-72
SCOC-AS1	ENSG0000(	1.47	14.03	-2.605	5.24E-63
SMIM10L2	ENSG0000(	0.49	7.01	-2.426	1.52E-55
NPIPB9	ENSG0000(	0.93	19.92	-3.438	1.18E-79
RP11-573D	ENSG0000(	0.58	7.62	-2.448	1.36E-28
LRRC8B	ENSG0000(	1.23	11.41	-2.476	2.18E-49
NEK5	ENSG0000(	0.92	10.7	-2.607	5.72E-40
MAGEA6	ENSG0000(	0.09	9.65	-3.288	6.32E-38
PIWIL2	ENSG0000(	4.21	21.021	-2.079	1.11E-19
NOL4L	ENSG0000(	14.77	71.13	-2.193	5.03E-42
KB-1592A4	ENSG0000(	0.02	91.088	-6.496	2.01E-83
TPSB2	ENSG0000(	0.25	8.83	-2.975	7.14E-22
ZNF165	ENSG0000(	2.2	21.299	-2.801	2.25E-44
VEPH1	ENSG0000(	0.17	9.61	-3.181	2.42E-34
FABP12	ENSG0000(	0	6.67	-2.939	2.11E-45
GGT3P	ENSG0000(	0.12	3.85	-2.114	5.14E-57
ZNF628	ENSG0000(	5.45	31.71	-2.342	7.35E-45
LINC00477	ENSG0000(	0	3.13	-2.046	2.66E-64
TTC30A	ENSG0000(	0.72	8.9	-2.525	2.63E-64
SSPO	ENSG0000(	0.12	4.55	-2.309	3.11E-64
ZNF433	ENSG0000(	2.61	32.919	-3.232	1.77E-75
TXNRD3	ENSG0000(	4.66	39.029	-2.822	1.64E-73
KLHDC1	ENSG0000(	0.34	4.76	-2.104	2.05E-72
RP1-253P7	ENSG0000(	0.13	5.34	-2.488	2.48E-79

FAM212B	ENSG00000	1.34	10.17	-2.255	6.77E-57
ZNF790	ENSG00000	2.14	13.38	-2.195	1.64E-51
MEIG1	ENSG00000	0.62	19.35	-3.651	7.31E-71
LEKR1	ENSG00000	0.16	13.32	-3.626	1.73E-84
KEL	ENSG00000	1.46	76.468	-4.977	1.01E-72
SPANXA1	ENSG00000	1.29	22.15	-3.338	1.13E-27
TUBA3C	ENSG00000	0.13	532.411	-8.883	5.45E-83
NUP62CL	ENSG00000	0.83	11.63	-2.787	2.23E-50
MB	ENSG00000	0.47	4.95	-2.017	9.73E-25
NPIPB6	ENSG00000	0.87	53.101	-4.855	4.40E-78
HMGN5	ENSG00000	8.52	103.852	-3.461	8.18E-60
SVIP	ENSG00000	7.82	75.829	-3.123	1.49E-76
FAM47C	ENSG00000	0.03	3.8	-2.22	1.05E-55
RPS6KL1	ENSG00000	4.89	26.521	-2.224	2.66E-59
TUBB3	ENSG00000	0.09	19.721	-4.249	6.63E-75
RP11-98J2	ENSG00000	1.51	17.99	-2.919	5.17E-56
TMEM116	ENSG00000	7.27	33.139	-2.045	8.79E-57
NUP210P1	ENSG00000	0	7.16	-3.029	5.26E-51
TMEM239	ENSG00000	0	137.319	-7.112	1.80E-74
MYL4	ENSG00000	0.91	19.849	-3.448	2.49E-27
PLN	ENSG00000	0.34	5.31	-2.235	8.34E-31
RP11-598D	ENSG00000	0.2	9.38	-3.113	2.34E-69
SPANXC	ENSG00000	0	25.41	-4.723	1.07E-47
FAM19A2	ENSG00000	1.18	18.45	-3.157	8.86E-71
MAGEA1	ENSG00000	0.06	5.78	-2.677	1.53E-47
SMOC1	ENSG00000	3.31	44.749	-3.408	2.59E-56
LRRTM3	ENSG00000	0.02	15.2	-3.989	5.10E-78
PLXNB3	ENSG00000	0.39	27.651	-4.365	7.93E-58
OXCT2	ENSG00000	0.47	93.72	-6.01	5.85E-76
EPS8L3	ENSG00000	0.09	9.1	-3.212	1.16E-35
SYCP1	ENSG00000	0.13	44.479	-5.331	1.63E-93
MAGEB3	ENSG00000	0.17	5.28	-2.424	1.10E-69
STKLD1	ENSG00000	1.36	25.77	-3.504	1.25E-68
PNMA5	ENSG00000	1.42	11.12	-2.324	1.88E-12
ITPRIPL1	ENSG00000	2.47	35.631	-3.4	9.91E-43
MT-ND1	ENSG00000	2644.904	15412.36	-2.542	2.10E-43
SHISA4	ENSG00000	4.14	23.92	-2.277	2.91E-37
KIAA0753	ENSG00000	5.14	29.37	-2.306	4.88E-49
MFAP3L	ENSG00000	2.28	71.218	-4.461	6.58E-64
RNU5B-1	ENSG00000	0	14.32	-3.937	8.38E-14
SNORD118	ENSG00000	0	5.62	-2.727	1.63E-07
RNU4-1	ENSG00000	2.93	14.95	-2.021	6.49E-12
SNORA73	ENSG00000	0	3.25	-2.087	6.32E-39
RN7SK	ENSG00000	14.29	71.002	-2.235	7.87E-29
RN7SKP23(	ENSG00000	0	3.43	-2.147	5.75E-49
RP11-98D1	ENSG00000	0.78	6.89	-2.148	1.24E-43
FAM83H-A	ENSG00000	1.63	11.57	-2.257	7.66E-36
IBA57-AS1	ENSG00000	0.18	25.801	-4.505	4.02E-85
TCP10	ENSG00000	0	28.14	-4.865	2.53E-81

MSTO2P	ENSG0000(	1.95	15.519	-2.485	7.46E-56
FAM229B	ENSG0000(	15.55	586.789	-5.15	2.23E-71
FANK1	ENSG0000(	2.08	104.133	-5.093	5.74E-79
PRR9	ENSG0000(	0.02	3.43	-2.119	5.36E-24
LELP1	ENSG0000(	0.06	1406.408	-10.375	1.80E-79
FAM24A	ENSG0000(	0	21.18	-4.471	1.33E-62
PPAPDC1A	ENSG0000(	1.45	10.93	-2.284	6.54E-39
HIST2H2A/	ENSG0000(	33.831	198.775	-2.52	2.71E-42
SPATA42	ENSG0000(	0	353.654	-8.47	9.40E-75
SPANXA2	ENSG0000(	0.24	15.3	-3.717	3.53E-36
SAMD13	ENSG0000(	1.4	12.15	-2.454	2.52E-65
RP11-188(	ENSG0000(	0.04	8.54	-3.197	1.60E-58
RHOXF2B	ENSG0000(	0.08	16.89	-4.05	1.11E-23
LIPJ	ENSG0000(	0.02	5.53	-2.678	1.49E-55
TRPC5OS	ENSG0000(	0.06	15.72	-3.979	1.44E-96
LRRC73	ENSG0000(	0.29	60.522	-5.576	1.74E-82
TCEAL5	ENSG0000(	1.26	73.231	-5.038	1.37E-61
TCEAL6	ENSG0000(	0.04	33.831	-5.066	2.16E-94
TDRG1	ENSG0000(	0.21	112.938	-6.557	4.74E-91
TRAF3IP1	ENSG0000(	4.09	43.549	-3.13	2.51E-66
GIGYF2	ENSG0000(	16.72	72.7	-2.056	6.39E-52
PTPN20	ENSG0000(	0.73	12.21	-2.933	6.20E-87
GGNBP1	ENSG0000(	0.1	5.99	-2.668	1.04E-57
TXNDC8	ENSG0000(	0	26.64	-4.789	1.54E-68
PAGE3	ENSG0000(	0	9.81	-3.434	1.03E-74
TMEM225	ENSG0000(	0	15.49	-4.044	5.11E-63
ERICH2	ENSG0000(	2.17	222.443	-6.139	1.18E-63
SPANXN5	ENSG0000(	0	17.64	-4.22	1.17E-64
XAGE1A	ENSG0000(	7.16	39.349	-2.306	4.62E-06
XAGE1B	ENSG0000(	3.49	53.731	-3.608	1.02E-13
HSPA1L	ENSG0000(	1.5	82.968	-5.07	1.34E-66
MSH5	ENSG0000(	8.48	59.991	-2.686	8.41E-72
LY6G5C	ENSG0000(	2.46	26.8	-3.006	6.55E-65
PSORS1C1	ENSG0000(	1.46	136.692	-5.807	5.78E-95
LINC01552	ENSG0000(	0.05	13.42	-3.78	2.47E-79
TRIM31	ENSG0000(	0.39	5.6	-2.247	1.38E-47
ZNRD1-AS	ENSG0000(	1.85	32.199	-3.542	2.85E-72
RPS26P8	ENSG0000(	0.28	14	-3.551	7.74E-58
FAM153C	ENSG0000(	0.53	154.075	-6.663	1.74E-138
OR2H1	ENSG0000(	0	19.149	-4.333	1.21E-69
MAMDC2-	ENSG0000(	0.11	6.06	-2.669	1.06E-101
RANBP17	ENSG0000(	2.68	21.9	-2.638	1.73E-70
TTC25	ENSG0000(	2.22	58.282	-4.202	7.49E-56
SPATA31A:	ENSG0000(	0.15	10.76	-3.354	1.24E-59
FAM216A	ENSG0000(	11.27	57.181	-2.245	3.33E-50
FAM201A	ENSG0000(	0.14	3.92	-2.11	4.32E-73
IGFL2	ENSG0000(	0.83	9.44	-2.512	1.15E-40
RP6-99M1	ENSG0000(	0	5.21	-2.635	2.95E-52
FAM221B	ENSG0000(	0.02	19.231	-4.31	2.78E-69

LRRC10B	ENSG0000(	0.71	6.87	-2.202	2.93E-40
TRIM13	ENSG0000(	21.04	93.87	-2.106	7.47E-40
MS4A13	ENSG0000(	0	4.4	-2.433	1.93E-48
PRSS1	ENSG0000(	0.05	5.54	-2.639	1.67E-13
SYCE1L	ENSG0000(	1.69	31.13	-3.578	2.35E-61
FAM71F2	ENSG0000(	0.66	35.68	-4.466	2.52E-65
FAM205A	ENSG0000(	0	30.01	-4.955	2.95E-68
LINC00654	ENSG0000(	0.57	17.321	-3.545	1.64E-77
KRT17P7	ENSG0000(	0.04	20.841	-4.392	2.25E-79
TTLL10-AS	ENSG0000(	0.04	4.52	-2.408	7.25E-50
SPDYE2	ENSG0000(	1.12	12.85	-2.708	1.72E-49
KRT17P5	ENSG0000(	0.04	28.949	-4.848	1.88E-89
MGAT4D	ENSG0000(	0	19	-4.322	5.74E-70
KRT17P4	ENSG0000(	0.14	21.66	-4.313	1.46E-76
SLCO6A1	ENSG0000(	0.05	39.829	-5.281	1.05E-80
LINC00661	ENSG0000(	0.06	66.661	-5.996	9.25E-84
IZUMO3	ENSG0000(	0	16.79	-4.153	2.78E-59
RP11-812E	ENSG0000(	0	4.61	-2.488	5.39E-69
TP53TG3D	ENSG0000(	0.92	19.929	-3.446	4.26E-71
TP53TG3C	ENSG0000(	0.22	7.56	-2.811	3.19E-53
SERF1B	ENSG0000(	42.2	221.367	-2.364	4.87E-51
STAG3L1	ENSG0000(	2.37	27.88	-3.099	5.05E-92
MUC19	ENSG0000(	0.49	67.822	-5.529	6.15E-73
VCX3B	ENSG0000(	0.8	82.738	-5.54	5.14E-54
FAM183DF	ENSG0000(	0	5.77	-2.759	9.15E-57
RP11-1212	ENSG0000(	10.87	57.871	-2.31	1.11E-34
GAGE1	ENSG0000(	0.45	53.4	-5.229	1.53E-46
LINC01531	ENSG0000(	0	6.18	-2.844	1.40E-48
TTC23L	ENSG0000(	0.21	40.31	-5.093	1.91E-86
RFPL3S	ENSG0000(	3.17	109.068	-4.722	6.57E-59
LRRC72	ENSG0000(	0	6.06	-2.82	5.88E-53
SRRM2-AS	ENSG0000(	0.53	25.91	-4.137	8.90E-83
RP11-404K	ENSG0000(	0	83.8	-6.406	7.00E-74
SMIM21	ENSG0000(	0	3.13	-2.046	4.56E-66
ZDHHC11E	ENSG0000(	0.6	7.9	-2.476	3.89E-57
RP11-503N	ENSG0000(	0.11	19.34	-4.196	7.40E-82
GOLGA8O	ENSG0000(	0.05	8.41	-3.164	9.24E-68
TMEM191(	ENSG0000(	2.95	151.628	-5.272	3.35E-65
KB-1183D5	ENSG0000(	0	27.49	-4.832	3.93E-70
HBA1	ENSG0000(	6.76	39.21	-2.373	1.46E-06
TCEB3B	ENSG0000(	0	5.19	-2.63	2.33E-58
TSSK2	ENSG0000(	0	169.353	-7.412	5.70E-74
RP11-93O1	ENSG0000(	0	4.76	-2.526	8.68E-55
RP11-553A	ENSG0000(	0.42	6.6	-2.42	1.04E-65
LNP1	ENSG0000(	2.12	23.131	-2.951	2.22E-48
PRSS50	ENSG0000(	1.56	71.908	-4.832	3.98E-71
KRBOX1-AS	ENSG0000(	0	3.17	-2.06	2.05E-65
ZCWPW2	ENSG0000(	0.63	10.06	-2.762	4.79E-65
RNU1-28P	ENSG0000(	4.86	44.2	-2.947	1.13E-33

RNU1-27P	ENSG00000	4.86	44.301	-2.951	1.70E-36
RNU1-1	ENSG00000	4.86	44.2	-2.947	1.13E-33
RNVU1-18	ENSG00000	4.86	44.2	-2.947	1.13E-33
RNU1-2	ENSG00000	4.86	44.301	-2.951	1.70E-36
RNU1-4	ENSG00000	4.86	44.301	-2.951	1.70E-36
RNU1-3	ENSG00000	4.86	44.2	-2.947	1.13E-33
MT-RNR2	ENSG00000	1962.53	9678.044	-2.301	1.70E-34
KB-1572G7	ENSG00000	0.13	10.67	-3.368	9.46E-64
TRBV7-1	ENSG00000	0	9.55	-3.399	1.06E-53
TSSK1B	ENSG00000	0.02	68.659	-6.094	1.12E-69
PRR22	ENSG00000	7.16	66.32	-3.044	1.97E-44
SNORA57	ENSG00000	0	17.49	-4.209	1.74E-24
EWSAT1	ENSG00000	0.16	19.02	-4.109	1.60E-67
RPL29P7	ENSG00000	0	12.15	-3.717	1.76E-56
FGFR1OP	ENSG00000	12.3	69.772	-2.412	9.72E-54
RP11-760D	ENSG00000	0.03	3.5	-2.127	6.96E-52
RPL36AP48	ENSG00000	0	5.42	-2.683	2.65E-62
FAM24B	ENSG00000	2.41	13.1	-2.048	3.63E-66
C6orf165	ENSG00000	0.24	20.35	-4.106	1.12E-59
PPP1R2P10	ENSG00000	0.55	5.42	-2.05	6.15E-45
TSGA13	ENSG00000	0	17.95	-4.244	2.97E-66
GSTM2	ENSG00000	18.39	138.418	-2.846	3.89E-40
MAGEA12	ENSG00000	0.06	3.66	-2.136	2.26E-27
RPLP1P6	ENSG00000	1	14.3	-2.935	9.13E-83
YBX2P1	ENSG00000	0	6.17	-2.842	7.26E-71
TTLL13P	ENSG00000	0.15	18.75	-4.102	9.20E-73
RP13-39P1	ENSG00000	0.38	8.8	-2.828	3.33E-62
VN1R64P	ENSG00000	0	3.45	-2.154	3.83E-59
SLC25A1P3	ENSG00000	0.04	13.88	-3.839	3.36E-69
FAM209B	ENSG00000	0.41	147.298	-6.717	1.14E-77
TTLL3	ENSG00000	11.16	134.13	-3.474	4.17E-76
UCA1	ENSG00000	0.05	3.84	-2.205	9.18E-25
ST7-OT4	ENSG00000	0.06	21.98	-4.438	4.68E-88
TTC41P	ENSG00000	0.62	7.68	-2.422	3.33E-64
LCNL1	ENSG00000	0.24	58.141	-5.576	1.53E-115
LRRC37A4F	ENSG00000	3.62	42.209	-3.225	2.16E-23
FAM187A	ENSG00000	0.62	20.68	-3.742	1.27E-58
PLIN5	ENSG00000	0.38	4.54	-2.005	6.31E-50
SEC14L6	ENSG00000	0.38	6.68	-2.476	1.23E-66
STARD10	ENSG00000	27.42	387.216	-3.772	5.59E-52
LRRC37A11	ENSG00000	0.1	4.55	-2.335	2.49E-55
RP11-74E2	ENSG00000	0.02	7.3	-3.025	1.29E-67
RP11-989E	ENSG00000	0	8.19	-3.2	5.21E-75
SLC6A10P	ENSG00000	1.07	66.8	-5.034	1.94E-70
RP11-83B2	ENSG00000	0.03	4.24	-2.347	3.52E-66
ZSWIM8	ENSG00000	35.05	205.044	-2.515	3.38E-75
IQCF5	ENSG00000	0.26	135.889	-6.763	3.83E-62
IQCF6	ENSG00000	0	24.401	-4.667	8.63E-62
RP1-139D8	ENSG00000	0	155.19	-7.287	2.07E-73

RP11-429J	ENSG0000(	0.23	9.99	-3.159	8.38E-79
HNRNPA1F	ENSG0000(	0.03	40.461	-5.331	2.26E-66
LINC01347	ENSG0000(	1.86	22.969	-3.067	3.09E-46
RAD51AP2	ENSG0000(	0.09	7.69	-2.995	6.78E-75
U82695.5	ENSG0000(	0.04	16.34	-4.059	2.39E-88
SPATA31D	ENSG0000(	0.03	14.84	-3.943	2.99E-79
NPIPA8	ENSG0000(	1.95	17.95	-2.683	2.20E-19
TBC1D26	ENSG0000(	0.09	12.15	-3.593	3.34E-77
FAM74A1	ENSG0000(	0	4.84	-2.546	4.44E-36
LINC00588	ENSG0000(	0	3.52	-2.176	7.36E-58
RP11-1023	ENSG0000(	0.03	25.189	-4.668	8.88E-70
RP11-1023	ENSG0000(	1.68	10.71	-2.127	3.06E-52
GOLGA6B	ENSG0000(	0.03	12.11	-3.67	5.05E-56
GOLGA8B	ENSG0000(	18.75	106.92	-2.45	2.10E-33
KCNU1	ENSG0000(	0	6.29	-2.866	9.98E-56
HOMEZ	ENSG0000(	3.62	47.389	-3.389	7.01E-62
RNF212B	ENSG0000(	0.49	11.22	-3.036	1.30E-57
RP11-613H	ENSG0000(	0	3.45	-2.154	2.76E-53
TMCO5B	ENSG0000(	0	75.94	-6.266	8.14E-74
SLC25A5P1	ENSG0000(	0.21	13.17	-3.55	1.03E-79
NPEPL1	ENSG0000(	13.02	60.329	-2.129	3.89E-59
FAM230B	ENSG0000(	0.01	33.42	-5.091	9.36E-71
GAB4	ENSG0000(	0.02	4.47	-2.423	1.79E-76
GRK6P1	ENSG0000(	0.07	3.86	-2.183	1.08E-53
GUSBP9	ENSG0000(	1.7	25.989	-3.321	1.60E-69
RP13-104F	ENSG0000(	1.92	15.43	-2.492	7.75E-72
ZC3H11B	ENSG0000(	0.06	6.03	-2.729	7.37E-64
TTC34	ENSG0000(	0.16	11.8	-3.464	5.49E-83
HIST1H2AI	ENSG0000(	0	22.721	-4.568	7.10E-72
RP11-244K	ENSG0000(	0	9.32	-3.367	7.95E-67
GAPDHP72	ENSG0000(	0.03	9.52	-3.352	5.57E-68
POM121L4	ENSG0000(	0	4.81	-2.539	5.49E-52
RPS7P5	ENSG0000(	0	10.64	-3.541	1.78E-50
PSMC1P11	ENSG0000(	0	5.13	-2.616	2.21E-57
ISCA1P1	ENSG0000(	3.47	32.03	-2.885	2.25E-37
SYCE3	ENSG0000(	0.57	190.506	-6.93	5.82E-88
KRT19P1	ENSG0000(	0	23.779	-4.631	3.53E-69
RP11-889L	ENSG0000(	2.3	74.718	-4.52	1.10E-127
PAPOLB	ENSG0000(	0.05	16.45	-4.055	4.89E-73
TEX40	ENSG0000(	0	12.66	-3.772	1.31E-70
FAM228B	ENSG0000(	5.11	48.341	-3.014	3.14E-66
RPL23AP47	ENSG0000(	0	6.31	-2.87	2.04E-12
RP11-394A	ENSG0000(	0.25	12.13	-3.393	2.78E-42
FLJ35934	ENSG0000(	0.79	37.131	-4.413	2.59E-75
VAMP2	ENSG0000(	17.73	86.258	-2.22	7.20E-51
SLC25A51F	ENSG0000(	0	6.93	-2.987	1.00E-58
RP3-406A7	ENSG0000(	0	9.01	-3.323	9.16E-60
MTMR9LP	ENSG0000(	1.38	9.26	-2.108	1.25E-24
MAGEA3	ENSG0000(	0.13	10.39	-3.333	3.40E-35

POM121L1	ENSG0000(	0	16.8	-4.154	4.77E-69
EXOC3-AS	ENSG0000(	1.08	10.21	-2.43	5.49E-73
RNU2-2P	ENSG0000(	0.13	10.04	-3.288	8.26E-31
RN7SKP7	ENSG0000(	0	4.46	-2.449	1.37E-37
RP1-171K1	ENSG0000(	0.09	3.79	-2.136	6.37E-22
TH2LCRR	ENSG0000(	0	19.48	-4.356	1.54E-70
RP11-274J	ENSG0000(	0	5.03	-2.592	1.70E-50
RP11-542F	ENSG0000(	0	3.05	-2.018	2.90E-47
RP11-223F	ENSG0000(	0.02	7.5	-3.059	4.51E-56
SMPD4P1	ENSG0000(	0	3.13	-2.046	6.39E-76
RP11-393K	ENSG0000(	0	3.99	-2.319	6.55E-55
LINC01120	ENSG0000(	0.04	17.64	-4.164	3.53E-86
RP4-613A2	ENSG0000(	0.12	16.19	-3.94	1.53E-75
LINC01208	ENSG0000(	0.04	3.51	-2.117	7.16E-44
RP11-644K	ENSG0000(	0.06	11.51	-3.561	2.32E-68
RP4-717I2	ENSG0000(	7.06	40.731	-2.372	3.88E-50
MIR503HG	ENSG0000(	2.21	51.789	-4.04	5.58E-53
FAM95B1	ENSG0000(	2.39	208.831	-5.952	3.83E-65
RP11-73B2	ENSG0000(	0	6.01	-2.809	8.64E-55
RP11-430C	ENSG0000(	0	5.71	-2.746	4.80E-56
RP11-252F	ENSG0000(	0	4.08	-2.345	3.84E-49
ROR1-AS1	ENSG0000(	0.06	4.36	-2.338	9.81E-50
SNRPD2P2	ENSG0000(	0	14.55	-3.959	1.20E-59
RP4-622L5	ENSG0000(	0.14	4.05	-2.147	1.47E-63
LINC00633	ENSG0000(	0	14.44	-3.949	1.59E-60
POM121L1	ENSG0000(	0	12.18	-3.72	4.13E-56
RP11-433C	ENSG0000(	0.03	3.59	-2.156	3.57E-45
HAGLR	ENSG0000(	2.23	69.879	-4.456	1.40E-53
LINC00466	ENSG0000(	0	4.13	-2.359	3.54E-75
RP11-336K	ENSG0000(	0	3.14	-2.05	3.25E-52
MSL3P1	ENSG0000(	4.72	23.02	-2.07	3.16E-29
RP11-462C	ENSG0000(	0.11	11.44	-3.486	2.49E-66
LINC00703	ENSG0000(	0	4.94	-2.57	1.13E-58
PRR29	ENSG0000(	0.8	19.28	-3.494	6.07E-61
RP11-48F1	ENSG0000(	0	6.63	-2.932	1.58E-44
PRKAR2A-A	ENSG0000(	0.42	23.76	-4.124	1.61E-70
RP11-61J1	ENSG0000(	0	4.66	-2.501	3.83E-29
RP11-385M	ENSG0000(	0.05	4.09	-2.277	1.14E-58
LINC01015	ENSG0000(	0	20.06	-4.396	3.90E-75
GPX5	ENSG0000(	0.02	4.03	-2.302	2.18E-53
ZMIZ1-AS1	ENSG0000(	0.35	5.64	-2.298	5.04E-58
HSD52	ENSG0000(	0	11.45	-3.638	5.07E-64
PDSS1P2	ENSG0000(	0	9.26	-3.359	1.49E-63
LAMTOR5-	ENSG0000(	2.5	43.721	-3.676	1.10E-84
RP11-697E	ENSG0000(	0	3.73	-2.242	3.61E-47
TEX26-AS1	ENSG0000(	0.05	23.309	-4.533	2.00E-76
RP11-54O1	ENSG0000(	0	6.32	-2.872	1.72E-55
IQCF4	ENSG0000(	0	275.192	-8.11	1.39E-73
LINC00240	ENSG0000(	1.32	9.39	-2.163	6.42E-59

RP1-90J20	ENSG0000(	0.26	12.86	-3.459	2.12E-44
XX-FW810(	ENSG0000(	0	15.75	-4.066	1.38E-58
LARGE-AS1	ENSG0000(	0	31.18	-5.008	3.11E-64
RP11-160H	ENSG0000(	0	7	-3	6.96E-66
RP11-228E	ENSG0000(	0.51	6.36	-2.285	2.76E-74
RP11-349E	ENSG0000(	0	20.391	-4.419	1.67E-58
HMGB3P22	ENSG0000(	0.5	21.29	-3.893	7.05E-65
RP11-383C	ENSG0000(	0	5.32	-2.66	3.08E-46
RP11-456H	ENSG0000(	0	12.61	-3.767	1.73E-56
RP1-127D3	ENSG0000(	0	4.87	-2.553	6.07E-52
LA16c-83F	ENSG0000(	0.09	8.46	-3.117	3.60E-75
RP5-937E2	ENSG0000(	0.05	24.18	-4.584	5.24E-70
PCDH9-AS	ENSG0000(	0	3.58	-2.195	5.64E-48
TAF1A-AS1	ENSG0000(	1.26	10.01	-2.284	1.72E-53
RP11-415J	ENSG0000(	0.58	9	-2.662	6.99E-83
RP11-449J	ENSG0000(	0	4.69	-2.508	7.74E-45
SNX18P3	ENSG0000(	0.17	15.39	-3.808	1.22E-93
RP11-69A2	ENSG0000(	0	3.65	-2.217	4.89E-60
RP11-3B7.1	ENSG0000(	0.23	16.09	-3.796	2.53E-91
RP1-257A1	ENSG0000(	0	12.74	-3.78	2.92E-63
RAB6C-AS1	ENSG0000(	0.13	5.77	-2.583	1.25E-51
PLCB1-IT1	ENSG0000(	0	3.28	-2.098	1.52E-44
RP11-760D	ENSG0000(	0	3.9	-2.293	9.19E-66
RP11-390F	ENSG0000(	0.7	26.999	-4.042	9.77E-74
KCNMA1-A	ENSG0000(	0	4.39	-2.43	1.80E-44
LINC01492	ENSG0000(	0	4.49	-2.457	6.78E-53
RP11-550H	ENSG0000(	0	8.07	-3.181	1.76E-55
RP11-70C1	ENSG0000(	0.07	3.72	-2.141	4.99E-60
RP5-1022F	ENSG0000(	0	6.98	-2.996	3.04E-58
FAM225B	ENSG0000(	0.16	19.34	-4.132	4.10E-76
NRG3-AS1	ENSG0000(	0	9.33	-3.369	3.36E-55
RP5-850O1	ENSG0000(	0	3.77	-2.254	1.15E-48
PROSER2-A	ENSG0000(	0.2	6.71	-2.684	1.14E-67
FAM229A	ENSG0000(	1.84	174.525	-5.95	2.60E-96
RP11-452K	ENSG0000(	1.14	32.491	-3.968	1.31E-57
LINC00694	ENSG0000(	0.08	49.47	-5.546	1.72E-79
RP3-495K2	ENSG0000(	0	13.53	-3.861	7.59E-61
RP3-476K8	ENSG0000(	0	3.34	-2.118	2.36E-57
RP11-384K	ENSG0000(	8.28	43.691	-2.268	2.41E-61
ODF2-AS1	ENSG0000(	0.31	7.87	-2.759	3.59E-68
MTND1P23	ENSG0000(	3.32	17.08	-2.065	7.33E-06
OR7E19P	ENSG0000(	0.03	6.58	-2.88	3.15E-58
UBXN10-A	ENSG0000(	0.6	6.79	-2.284	1.85E-69
KCNIP2-AS	ENSG0000(	0.11	42.851	-5.304	7.35E-79
RP11-180C	ENSG0000(	0.18	10.28	-3.257	1.34E-61
RP4-712E4	ENSG0000(	0	7.12	-3.021	5.85E-54
TEX22	ENSG0000(	0.34	13.69	-3.455	6.39E-65
RP11-419C	ENSG0000(	3.82	28.31	-2.604	2.34E-59
LEMD1-AS	ENSG0000(	0.09	4.07	-2.218	2.76E-69

RP11-307F	ENSG0000(	0	5.06	-2.599	5.54E-36
GRM7-AS3	ENSG0000(	0	16.05	-4.092	1.37E-59
RP11-61N2	ENSG0000(	10.06	58.63	-2.431	2.55E-30
TMEM191/	ENSG0000(	11.29	150.33	-3.622	2.95E-45
RP11-435M	ENSG0000(	0	6.87	-2.976	1.71E-59
ZNF192P1	ENSG0000(	0.61	6.6	-2.239	9.24E-89
ST7-AS2	ENSG0000(	0	3.7	-2.233	3.34E-51
SORCS3-A	ENSG0000(	0	39.221	-5.33	6.44E-74
RP11-358D	ENSG0000(	0	6.37	-2.882	6.61E-57
RP11-31F1	ENSG0000(	2.72	24.02	-2.75	1.29E-54
OR8T1P	ENSG0000(	0.09	3.6	-2.077	2.20E-64
RP11-393J	ENSG0000(	0	3.49	-2.167	2.65E-49
RP3-462C1	ENSG0000(	0	22.8	-4.573	3.72E-31
RP13-401N	ENSG0000(	6.94	34.951	-2.179	2.50E-21
RBM22P3	ENSG0000(	0	7.65	-3.113	1.35E-71
LINC00390	ENSG0000(	0	3.7	-2.233	6.97E-57
LINC00606	ENSG0000(	0	7.07	-3.013	8.41E-55
RP11-340I(	ENSG0000(	0.19	7.79	-2.885	7.12E-61
LINC00343	ENSG0000(	0	37.741	-5.276	6.49E-74
RP11-128N	ENSG0000(	0.47	5.03	-2.036	8.82E-48
KIF4B	ENSG0000(	0.01	3.11	-2.025	9.32E-64
PSMD10P2	ENSG0000(	0.06	4.36	-2.338	4.99E-49
RP11-526D	ENSG0000(	0.05	21.29	-4.408	3.76E-71
TEX41	ENSG0000(	1.96	10.88	-2.005	4.84E-62
LENG8-AS1	ENSG0000(	1.69	14.05	-2.484	2.14E-92
LINC00424	ENSG0000(	0	4.7	-2.511	8.65E-55
PSMD5-AS	ENSG0000(	7.2	38.751	-2.277	1.99E-48
RP5-1024C	ENSG0000(	0.95	34.151	-4.172	1.16E-70
SRRM5	ENSG0000(	0.4	57.452	-5.384	3.48E-72
RP11-203E	ENSG0000(	0.96	17.97	-3.275	3.35E-98
MROH5	ENSG0000(	0.1	11.07	-3.456	6.21E-55
RP11-148E	ENSG0000(	0.06	6.01	-2.725	9.61E-55
RP11-402F	ENSG0000(	0.77	13.43	-3.027	2.14E-23
RP11-575L	ENSG0000(	0.18	7.96	-2.925	2.21E-72
LINC01359	ENSG0000(	0.05	4.3	-2.336	4.00E-89
LINC00354	ENSG0000(	0	9.74	-3.425	3.05E-65
LINC00454	ENSG0000(	0	19.7	-4.372	8.99E-86
RBMY1J	ENSG0000(	0	5.72	-2.748	2.46E-68
LINC00658	ENSG0000(	0	77.128	-6.288	6.11E-76
RP5-956O1	ENSG0000(	0.03	8.68	-3.232	5.66E-62
RP11-168F	ENSG0000(	0	12.35	-3.739	4.11E-61
LINC00511	ENSG0000(	1.26	13.42	-2.674	1.09E-40
RP11-286N	ENSG0000(	0	16.31	-4.114	2.07E-66
RBMX2P5	ENSG0000(	0.03	7.31	-3.012	7.26E-64
RP11-433J	ENSG0000(	0	10.92	-3.575	3.17E-61
LBX1-AS1	ENSG0000(	0.28	12.48	-3.397	3.09E-55
RP11-545A	ENSG0000(	0.15	6.89	-2.778	1.06E-71
RP11-196D	ENSG0000(	0.32	12.39	-3.343	3.30E-47
RP11-214D	ENSG0000(	0	10.73	-3.552	9.95E-55

RP11-317F	ENSG0000(	0	10.24	-3.491	3.95E-47
MIR663AH	ENSG0000(	0.1	45.069	-5.388	1.95E-74
RP11-121A	ENSG0000(	0	4.45	-2.446	3.57E-49
XXbac-BCX	ENSG0000(	0	4.51	-2.462	1.89E-50
RP11-367J	ENSG0000(	0	5.83	-2.772	5.65E-42
RP11-203J	ENSG0000(	0.23	10.07	-3.17	1.40E-73
SPANXB1	ENSG0000(	0.24	92.161	-6.231	7.16E-66
SMIM2-AS	ENSG0000(	0.84	12.09	-2.831	2.24E-56
LINC01364	ENSG0000(	0	11.19	-3.608	7.74E-53
RP4-669P1	ENSG0000(	0.52	13.57	-3.261	2.60E-79
STK4-AS1	ENSG0000(	0.24	4.49	-2.146	4.28E-62
RP11-152N	ENSG0000(	3.91	23.74	-2.333	1.02E-70
SPAG5-AS	ENSG0000(	1.49	45.01	-4.208	3.13E-94
RP5-905H7	ENSG0000(	0	17.81	-4.233	1.07E-61
RP1-28O1(	ENSG0000(	0.5	7.07	-2.428	1.29E-78
LINC01068	ENSG0000(	0	4.58	-2.48	2.80E-58
RP11-73O(	ENSG0000(	0.4	8.64	-2.784	1.48E-46
RP11-578F	ENSG0000(	0	19.46	-4.355	7.37E-64
PSPC1P1	ENSG0000(	0	5.46	-2.692	4.38E-59
RP11-102(	ENSG0000(	0	21.96	-4.521	1.35E-60
RP11-73B2	ENSG0000(	0.55	6.15	-2.206	4.48E-50
RP11-141N	ENSG0000(	0	3.74	-2.245	3.03E-49
RP11-143N	ENSG0000(	0.06	18.47	-4.199	1.70E-61
RP11-191N	ENSG0000(	0	21.64	-4.501	5.52E-68
RP11-372N	ENSG0000(	0	4.35	-2.42	3.92E-42
LINC01341	ENSG0000(	1.16	12.95	-2.691	6.90E-47
RP11-383(	ENSG0000(	0.05	10.88	-3.5	4.90E-64
RP11-69E1	ENSG0000(	0	12.48	-3.753	4.63E-67
MFI2-AS1	ENSG0000(	3.95	68.379	-3.809	9.20E-71
RP11-165F	ENSG0000(	0.13	5.48	-2.52	5.73E-60
RP3-467K1	ENSG0000(	0.02	4.77	-2.5	6.79E-69
RP1-317E2	ENSG0000(	0.67	11.24	-2.874	9.16E-79
RP11-244N	ENSG0000(	0	4.25	-2.392	9.09E-39
RP13-60M	ENSG0000(	0	8.21	-3.203	4.56E-55
MT-ATP8	ENSG0000(	6584.233	28407.81	-2.109	2.00E-30
RP11-1379	ENSG0000(	0	7.77	-3.133	2.32E-63
RALY-AS1	ENSG0000(	1.39	18.379	-3.019	1.78E-66
PCAT6	ENSG0000(	4.29	119.684	-4.512	7.97E-67
RP11-132N	ENSG0000(	0	78.858	-6.319	2.60E-72
LINC01350	ENSG0000(	0	79.798	-6.336	3.16E-69
RP11-235(	ENSG0000(	0.04	10.93	-3.52	2.91E-69
RP11-206L	ENSG0000(	2.76	14.94	-2.084	2.02E-29
RPL17P43	ENSG0000(	0.3	13.96	-3.525	4.49E-52
RP5-845O2	ENSG0000(	0	10.54	-3.529	3.03E-53
MIR646HG	ENSG0000(	0.6	17.179	-3.506	1.26E-56
RP11-365F	ENSG0000(	0.2	7.69	-2.856	1.40E-59
RP11-85L2	ENSG0000(	0.06	27.971	-4.772	9.96E-57
GAS2L1P2	ENSG0000(	0.22	8.1	-2.899	1.93E-54
LINC00687	ENSG0000(	0	15.8	-4.07	1.84E-63

RP5-864K1	ENSG0000(	0.52	20.05	-3.792	3.09E-69
LINC01125	ENSG0000(	5.18	46.09	-2.93	5.77E-49
LINC01013	ENSG0000(	0.16	6.27	-2.648	5.70E-52
RP11-347D	ENSG0000(	0	8.49	-3.246	1.04E-61
RP4-586O1	ENSG0000(	0	81.007	-6.358	1.24E-68
RP11-108M	ENSG0000(	0.24	11.37	-3.318	6.89E-58
HOTAIR	ENSG0000(	0.09	6.77	-2.834	1.02E-54
RP5-1051H	ENSG0000(	0.35	99.898	-6.224	2.24E-61
LINC00448	ENSG0000(	0	7.9	-3.154	5.39E-55
RP4-676J1	ENSG0000(	0	3.06	-2.021	1.26E-50
RP11-336A	ENSG0000(	0	3.6	-2.202	1.13E-38
RP3-335N1	ENSG0000(	0	8.6	-3.263	1.71E-60
TNKS2-AS1	ENSG0000(	1.12	13.43	-2.767	1.26E-58
RP11-284C	ENSG0000(	0	4.19	-2.376	6.04E-56
GS1-18A18	ENSG0000(	0	5.2	-2.632	9.06E-58
GABRG3-A	ENSG0000(	0	41.659	-5.415	9.24E-69
RP13-39P1	ENSG0000(	8.45	63.109	-2.762	2.17E-41
RP11-242F	ENSG0000(	0.01	6.08	-2.809	1.34E-60
RP4-784A1	ENSG0000(	0	3.34	-2.118	2.31E-44
PIK3IP1-AS	ENSG0000(	0.26	8.7	-2.945	4.28E-99
TSPY3	ENSG0000(	0.32	7.09	-2.616	1.16E-05
HOMER2P1	ENSG0000(	0.21	5.08	-2.329	2.43E-09
RP11-265D	ENSG0000(	0.19	4.82	-2.29	7.91E-06
LINC01277	ENSG0000(	0.02	5.41	-2.652	2.06E-54
HSFY1P1	ENSG0000(	0	3.01	-2.004	4.47E-50
SPRR2C	ENSG0000(	0	47.669	-5.605	3.11E-67
RP11-20P5	ENSG0000(	0	3.38	-2.131	4.94E-53
LINC01548	ENSG0000(	0	14.08	-3.915	7.46E-61
RP11-250H	ENSG0000(	0.12	6.61	-2.764	7.87E-46
PGA4	ENSG0000(	0.05	4.19	-2.305	5.17E-32
LINC00710	ENSG0000(	0	63.848	-6.019	1.18E-69
HNRNPA1F	ENSG0000(	0.14	4.29	-2.214	2.20E-52
RP11-552D	ENSG0000(	0	14.85	-3.986	3.18E-73
POM121L8	ENSG0000(	0	22.38	-4.547	7.21E-67
MCHR2-AS	ENSG0000(	0	99.442	-6.65	4.43E-78
KRT8P12	ENSG0000(	6.25	32.599	-2.212	7.09E-46
LINC00452	ENSG0000(	0.03	5.24	-2.599	1.16E-69
RP11-12M	ENSG0000(	0	9.52	-3.395	5.37E-62
RP11-494C	ENSG0000(	0	8.18	-3.199	1.03E-27
RP11-202I1	ENSG0000(	0	5.55	-2.711	4.22E-51
RP11-202D	ENSG0000(	0	6.99	-2.998	8.52E-55
LINC01523	ENSG0000(	0	6.37	-2.882	5.64E-59
RP11-119E	ENSG0000(	0.69	5.95	-2.04	3.42E-56
UBE2DNL	ENSG0000(	0.52	34.951	-4.564	2.48E-59
RP11-423C	ENSG0000(	0.05	4.33	-2.344	1.83E-58
RP5-827L5	ENSG0000(	0	12.29	-3.732	4.10E-58
RP11-390F	ENSG0000(	0.1	4.48	-2.317	2.22E-44
LINC01501	ENSG0000(	0.04	3.66	-2.164	1.75E-54
ISPD-AS1	ENSG0000(	0.11	10.64	-3.39	6.70E-57

RP11-374N	ENSG00000	0	64.829	-6.041	5.36E-74
XX-CR54.1	ENSG00000	0.09	14.32	-3.813	4.57E-88
RP5-971N1	ENSG00000	0.02	8.06	-3.151	1.34E-75
RP11-298H	ENSG00000	0	3.49	-2.167	6.75E-45
UBE2Q1-A	ENSG00000	0.1	7.11	-2.882	6.75E-72
SNRPFP2	ENSG00000	0	3.27	-2.094	2.40E-44
ZNF688	ENSG00000	5.43	41.821	-2.735	4.18E-59
PGA3	ENSG00000	0.1	10.78	-3.421	2.35E-59
RP11-363N	ENSG00000	0	3.75	-2.248	7.91E-66
RP11-668C	ENSG00000	0.02	15.95	-4.055	1.03E-62
HSPD1P21	ENSG00000	0	17.81	-4.233	2.39E-77
RHEBP1	ENSG00000	1.78	10.5	-2.049	7.68E-35
PRPS1L1	ENSG00000	0.05	37.379	-5.192	2.95E-82
ZRANB2-A	ENSG00000	0.45	5.41	-2.144	5.75E-88
RP11-366F	ENSG00000	0.03	4.53	-2.425	5.58E-44
IQCF3	ENSG00000	0.03	177.293	-7.435	6.42E-71
TOB1-AS1	ENSG00000	0.59	10.2	-2.816	1.04E-69
RP5-905H7	ENSG00000	0.09	42.851	-5.33	1.68E-61
RP11-217E	ENSG00000	0.07	43.449	-5.376	2.85E-73
RP11-80B9	ENSG00000	0	30.33	-4.969	5.23E-61
LINC00364	ENSG00000	0	4.15	-2.365	1.46E-42
RP11-402C	ENSG00000	0	77.541	-6.295	2.53E-71
RP11-407E	ENSG00000	0.07	12.69	-3.677	3.78E-54
RP11-232D	ENSG00000	1.91	24.429	-3.127	2.43E-22
FAM92A1P	ENSG00000	0.01	13.6	-3.854	8.50E-63
RP4-697K1	ENSG00000	0	3.09	-2.032	1.02E-45
SPATA31C1	ENSG00000	0.1	17.45	-4.068	9.15E-68
RP11-90C4	ENSG00000	0	3.37	-2.128	1.74E-50
RP11-334J	ENSG00000	0.5	9.36	-2.788	7.69E-67
NAALADL2	ENSG00000	0	4.65	-2.498	1.93E-43
RP11-213G	ENSG00000	0	28.019	-4.859	8.01E-84
TPI1P2	ENSG00000	0.46	4.91	-2.017	5.09E-43
VN1R32P	ENSG00000	0.05	14.04	-3.84	4.37E-76
KARSP2	ENSG00000	0.04	6.57	-2.864	3.10E-74
GOLGA6L5I	ENSG00000	2.33	43.09	-3.727	3.17E-94
RP4-737E2	ENSG00000	0.01	12.48	-3.738	4.99E-69
LINC01066	ENSG00000	0	4.75	-2.524	2.08E-49
RP5-902P8	ENSG00000	0.81	23.53	-3.76	5.11E-99
RP11-14O1	ENSG00000	0	6.77	-2.958	4.77E-45
RP11-390E	ENSG00000	0	7.05	-3.009	1.03E-51
LRRC37A6F	ENSG00000	0.2	5.11	-2.348	5.67E-63
U73166.2	ENSG00000	0.16	26.87	-4.587	1.10E-83
PROX1-AS1	ENSG00000	0	6.63	-2.932	3.50E-60
NOS2P3	ENSG00000	0.06	13.25	-3.749	5.27E-77
RP11-95M	ENSG00000	0	4.5	-2.459	1.23E-54
RP11-297A	ENSG00000	0.06	8.93	-3.228	5.69E-81
RP11-74C1	ENSG00000	0.04	7.63	-3.053	4.28E-65
LINC01183	ENSG00000	0.21	17.579	-3.941	4.67E-72
RP5-828H9	ENSG00000	0.03	16.62	-4.096	1.11E-63

ZNF32-AS2	ENSG00000	0.44	5.03	-2.066	1.24E-77
GPAA1P2	ENSG00000	0.82	6.75	-2.09	1.18E-50
RP11-402(	ENSG00000	0	30.26	-4.966	4.82E-67
RP5-1198(	ENSG00000	1.29	11.1	-2.402	7.89E-46
RP11-286H	ENSG00000	0.14	6.39	-2.697	8.23E-66
RP3-406P2	ENSG00000	0	4.84	-2.546	1.28E-41
RGPD4-AS	ENSG00000	0	32.581	-5.07	1.30E-82
SAP18P3	ENSG00000	0	3.35	-2.121	9.19E-49
LINC01001	ENSG00000	36.849	169.177	-2.169	7.59E-29
RP4-738P1	ENSG00000	0.01	5.39	-2.662	1.69E-58
ZNF341-AS	ENSG00000	0	4.84	-2.546	8.64E-60
RP11-251F	ENSG00000	0	52.091	-5.73	1.09E-67
FOXD3-AS	ENSG00000	0.17	13.55	-3.636	3.52E-05
RP5-968J1	ENSG00000	0	5.53	-2.707	4.10E-44
RP11-383M	ENSG00000	0	3.33	-2.114	7.21E-55
RP11-195E	ENSG00000	0.12	3.95	-2.144	4.66E-11
RP11-379(	ENSG00000	0	3.77	-2.254	1.53E-51
STMND1	ENSG00000	0.05	7	-2.93	1.73E-61
LINC00692	ENSG00000	0	5.44	-2.687	3.42E-59
RP11-314(	ENSG00000	0.24	4.09	-2.037	5.29E-68
RP4-631H1	ENSG00000	0	5.3	-2.655	4.38E-27
LINC00326	ENSG00000	0	13.32	-3.84	9.99E-67
LINC00271	ENSG00000	0.14	14.52	-3.767	2.54E-81
RP1-140A9	ENSG00000	0.13	4.47	-2.275	2.10E-64
RP11-263K	ENSG00000	1.22	15.56	-2.899	6.61E-57
RP11-514F	ENSG00000	0	3.27	-2.094	4.29E-38
TSSK1A	ENSG00000	0.18	6.78	-2.721	3.40E-77
RP5-1073(	ENSG00000	0	9.09	-3.335	1.09E-73
HAR1B	ENSG00000	0.05	6.24	-2.786	9.88E-57
RP1-207H1	ENSG00000	0.03	5.03	-2.549	1.24E-49
RP11-34D1	ENSG00000	0	8.35	-3.225	6.63E-61
RP11-495F	ENSG00000	0.37	7.88	-2.696	6.05E-43
PCED1CP	ENSG00000	0	4.71	-2.513	6.52E-69
RP11-723(	ENSG00000	0.7	9.52	-2.63	2.64E-70
RP1-225E1	ENSG00000	0.09	7.44	-2.953	2.82E-101
RP11-148E	ENSG00000	0	3.28	-2.098	1.58E-34
RP11-413(	ENSG00000	0	7.8	-3.138	2.00E-54
FAR2P4	ENSG00000	0.11	21.769	-4.358	6.32E-64
LINC00441	ENSG00000	0.07	7.83	-3.045	1.28E-68
FAM225A	ENSG00000	0.77	36.969	-4.423	1.83E-77
RP11-410(	ENSG00000	0	4.11	-2.353	2.19E-56
LINC01249	ENSG00000	0.21	9.07	-3.057	4.34E-27
RP11-162K	ENSG00000	0	6.59	-2.924	2.44E-56
FAHD2CP	ENSG00000	6.83	47.52	-2.631	7.33E-64
RP11-575L	ENSG00000	0.05	10.14	-3.407	1.05E-74
RPS6KA2-A	ENSG00000	0	13.94	-3.901	2.62E-62
RP11-404(	ENSG00000	0.07	8.07	-3.083	1.58E-65
LINC-PINT	ENSG00000	1.78	13.86	-2.418	5.63E-61
TSPAN19	ENSG00000	0.06	5.98	-2.719	3.96E-58

RP11-227H	ENSG0000(	0.21	4.35	-2.144	1.25E-22
RP11-402F	ENSG0000(	0	9.05	-3.329	6.24E-27
PCAT7	ENSG0000(	0.19	4.12	-2.105	3.97E-39
LINC01388	ENSG0000(	0	3.05	-2.018	1.88E-56
LINC01198	ENSG0000(	3.38	38.62	-3.177	4.94E-27
PRH1	ENSG0000(	1.18	80.738	-5.229	2.32E-63
RP11-29B9	ENSG0000(	0	6.47	-2.901	4.02E-52
RP11-557H	ENSG0000(	0	4.57	-2.478	1.42E-51
LINC00202	ENSG0000(	0.71	213.027	-6.968	1.96E-76
PPP1R2P3	ENSG0000(	0.06	50.611	-5.606	2.42E-76
RP11-111F	ENSG0000(	0.35	8.33	-2.789	1.26E-45
FLJ27354	ENSG0000(	0.71	39.021	-4.549	1.08E-99
LEF1-AS1	ENSG0000(	0.21	49.6	-5.386	3.22E-82
ZBED9	ENSG0000(	1.26	12.72	-2.602	7.96E-69
XXbac-BPG	ENSG0000(	0	29.9	-4.95	1.05E-65
RFX3-AS1	ENSG0000(	0.26	5.79	-2.43	6.83E-75
RP11-360D	ENSG0000(	0	32.479	-5.065	3.05E-74
RP3-461P1	ENSG0000(	0	14.9	-3.991	4.13E-65
RP11-302L	ENSG0000(	0	8.28	-3.214	5.62E-67
LINC00202	ENSG0000(	1.25	91.842	-5.367	4.76E-54
LINC00865	ENSG0000(	0.36	21.421	-4.043	4.68E-73
RP11-4C2(	ENSG0000(	0	3.11	-2.039	1.46E-52
XXyac-YX1	ENSG0000(	2.51	88.223	-4.668	9.55E-48
RP11-240L	ENSG0000(	0	3.3	-2.104	1.01E-72
GNG12-AS	ENSG0000(	0.04	3.68	-2.17	2.66E-56
RP4-715N1	ENSG0000(	0	4.84	-2.546	5.85E-71
RP11-154D	ENSG0000(	0.06	20.09	-4.314	1.21E-71
RP11-46O2	ENSG0000(	0	4.14	-2.362	3.35E-41
VIPR1-AS1	ENSG0000(	0.04	4.43	-2.384	5.62E-43
RP3-399L1	ENSG0000(	0	3.17	-2.06	2.75E-45
RP5-1022J	ENSG0000(	0	7.81	-3.139	1.97E-52
OSBPL10-A	ENSG0000(	0	14.74	-3.976	3.52E-67
RP11-403N	ENSG0000(	0	4.15	-2.365	1.42E-53
XXbac-BPG	ENSG0000(	0	7.96	-3.163	5.89E-65
RP11-385N	ENSG0000(	0.1	8.31	-3.081	3.12E-52
RSU1P2	ENSG0000(	0	7.87	-3.149	9.72E-57
LINC01517	ENSG0000(	0.07	7.1	-2.92	9.36E-65
LINC00385	ENSG0000(	0	7.27	-3.048	9.41E-49
LINC01431	ENSG0000(	0.7	16.939	-3.4	3.51E-70
GOLGA8N	ENSG0000(	2.11	28.019	-3.222	2.27E-71
RP4-718D2	ENSG0000(	0	4.43	-2.441	3.94E-41
LINC00442	ENSG0000(	0	20.33	-4.415	1.45E-65
MARK2P9	ENSG0000(	0.05	7.07	-2.942	5.36E-73
KIZ-AS1	ENSG0000(	0.01	3.3	-2.09	3.10E-53
LINC01022	ENSG0000(	0	4.66	-2.501	1.81E-61
YWHAEP1	ENSG0000(	0.05	10.8	-3.49	1.96E-72
RP11-382F	ENSG0000(	0.31	6.47	-2.512	2.52E-29
RP11-498E	ENSG0000(	0.18	44.56	-5.271	1.76E-61
LMLN-AS1	ENSG0000(	0.05	4.01	-2.254	2.30E-54

SNX18P2	ENSG0000(	0	3.01	-2.004	8.01E-50
SMG7-AS1	ENSG0000(	0.27	23.669	-4.28	3.46E-80
RP4-665N4	ENSG0000(	0.03	10.54	-3.486	1.80E-69
SEC1P	ENSG0000(	0.08	6.59	-2.813	3.98E-70
RP5-1115A	ENSG0000(	0.11	4.79	-2.383	2.11E-54
PLCE1-AS2	ENSG0000(	0	22.11	-4.53	4.99E-69
LINC00342	ENSG0000(	3.27	93.118	-4.462	6.76E-106
VPS13A-AS	ENSG0000(	0	24.55	-4.675	2.02E-64
RP11-475C	ENSG0000(	0.03	5.88	-2.74	1.12E-71
RP5-908M	ENSG0000(	0	20.21	-4.407	6.45E-65
RP11-1212	ENSG0000(	64.382	306.384	-2.233	5.30E-48
RP11-24P1	ENSG0000(	0	6.17	-2.842	6.78E-49
RP5-930J4	ENSG0000(	0	3.08	-2.029	3.39E-38
RP11-90C4	ENSG0000(	0	3.06	-2.021	5.45E-42
RP11-440C	ENSG0000(	0.1	4.06	-2.202	3.57E-50
RP11-375C	ENSG0000(	0.01	5.37	-2.657	1.60E-61
HOXB-AS3	ENSG0000(	0.48	9.03	-2.761	1.41E-46
VN1R20P	ENSG0000(	0.1	3.73	-2.104	2.96E-83
RP11-364E	ENSG0000(	0	16.17	-4.102	1.12E-48
UBE2E2-AS	ENSG0000(	0	12.21	-3.724	3.54E-75
RP11-863K	ENSG0000(	1.36	8.62	-2.027	3.39E-49
RP11-536F	ENSG0000(	0	3.32	-2.111	3.74E-45
RP3-468B3	ENSG0000(	0.03	24.01	-4.602	3.54E-73
RP11-67L3	ENSG0000(	0.43	23.28	-4.086	4.59E-62
LINC00642	ENSG0000(	0.01	7.45	-3.065	1.68E-61
LINC00167	ENSG0000(	0.04	3.49	-2.11	6.54E-65
NPIPB7	ENSG0000(	0.4	33.77	-4.634	1.88E-75
LINC00472	ENSG0000(	0.11	4.03	-2.18	3.88E-73
RP11-415J	ENSG0000(	0	15.82	-4.072	1.97E-68
LINC00264	ENSG0000(	0.09	50.422	-5.56	5.85E-76
RP5-1030M	ENSG0000(	0	5.73	-2.751	1.29E-54
RP5-875O1	ENSG0000(	0.19	17.43	-3.953	2.27E-64
STXBP5-AS	ENSG0000(	0.14	11.43	-3.447	7.52E-74
RP11-295C	ENSG0000(	3.33	55.001	-3.693	1.78E-68
LINC01518	ENSG0000(	0	8.34	-3.223	1.98E-79
RP3-486I3.	ENSG0000(	0.93	10.2	-2.537	2.84E-70
LINC00690	ENSG0000(	0	7.44	-3.077	1.41E-62
RP4-665J2	ENSG0000(	1.31	12.94	-2.593	6.68E-58
RP11-22M	ENSG0000(	0	8.51	-3.249	6.58E-54
RP11-123C	ENSG0000(	0	4.12	-2.356	1.88E-46
LINC01251	ENSG0000(	0.05	11.15	-3.532	1.12E-81
RP11-295C	ENSG0000(	0	7.98	-3.167	7.78E-61
TSPY4	ENSG0000(	1.53	36.131	-3.875	8.57E-08
RP4-650F1	ENSG0000(	0	17.759	-4.23	2.25E-73
LINC01360	ENSG0000(	0	5.63	-2.729	1.73E-36
RP11-823F	ENSG0000(	0.02	15.12	-3.982	3.94E-63
PPP1R2P2	ENSG0000(	0.07	8.55	-3.158	9.49E-64
PAGE2	ENSG0000(	0.89	32.45	-4.146	8.19E-37
RP11-1217	ENSG0000(	0	22.59	-4.56	1.73E-67

RP11-168(	ENSG0000(	0	15.76	-4.067	3.45E-54
RP11-395L	ENSG0000(	0	9.1	-3.336	9.96E-65
RP11-446F	ENSG0000(	0	3.1	-2.036	7.04E-44
LINC01191	ENSG0000(	0.19	172.792	-7.19	3.12E-72
RP5-942I1(	ENSG0000(	0.11	54.738	-5.65	2.37E-79
RP11-315I:	ENSG0000(	0.51	17.55	-3.619	1.25E-74
TMEM229/	ENSG0000(	0.21	5.42	-2.408	2.45E-54
RP11-353N	ENSG0000(	0.09	10.58	-3.409	1.91E-64
RP3-325F2	ENSG0000(	0	34.859	-5.164	1.31E-62
RP11-157E	ENSG0000(	0	36.511	-5.229	2.36E-74
TULP3P1	ENSG0000(	0	3.63	-2.211	1.24E-68
RPL36P20	ENSG0000(	0	9.85	-3.44	8.29E-69
RP13-16H:	ENSG0000(	0	18.371	-4.276	2.77E-69
LINC00229	ENSG0000(	0	12.92	-3.799	6.39E-59
RP11-52J3	ENSG0000(	0	5.43	-2.685	1.30E-54
RP11-340I(	ENSG0000(	0.09	13.82	-3.765	1.34E-72
RP11-561(	ENSG0000(	0.08	34.391	-5.034	3.17E-74
RBMY1A1	ENSG0000(	0.07	11.47	-3.543	7.04E-62
FGF14-AS1	ENSG0000(	0	14.23	-3.929	4.88E-61
SLC16A12-	ENSG0000(	0	8.73	-3.282	4.99E-52
ERICH3-AS	ENSG0000(	0	3.93	-2.302	4.01E-41
RP11-78H:	ENSG0000(	0	5.53	-2.707	1.12E-45
LINC01309	ENSG0000(	0	5.92	-2.791	1.26E-43
RPSAP9	ENSG0000(	1.01	7.49	-2.079	3.36E-69
RP1-149A1	ENSG0000(	0	3.43	-2.147	2.02E-47
WDR82P1	ENSG0000(	0.1	3.43	-2.01	1.70E-65
PCCA-AS1	ENSG0000(	0	8.31	-3.219	4.67E-55
RP11-262H	ENSG0000(	0.22	8.61	-2.978	6.85E-37
RP1-92O14	ENSG0000(	0.32	5.3	-2.255	7.88E-70
RP11-166E	ENSG0000(	3.37	37.049	-3.122	4.83E-70
FAM170B-	ENSG0000(	0	6.89	-2.98	2.01E-58
RP11-272F	ENSG0000(	0.12	3.52	-2.013	2.63E-53
LINC01052	ENSG0000(	0	7.15	-3.027	1.83E-55
RFTN1P1	ENSG0000(	0	5.42	-2.683	1.08E-54
RP11-526/	ENSG0000(	0.01	8.59	-3.247	7.23E-57
SLIT1-AS1	ENSG0000(	0	41.781	-5.419	2.21E-65
RP11-547(	ENSG0000(	0	13.72	-3.88	8.04E-60
SOX9-AS1	ENSG0000(	0.11	9.06	-3.18	3.45E-79
RP11-85O:	ENSG0000(	0	8.05	-3.178	2.30E-55
GRID1-AS1	ENSG0000(	0	12.79	-3.786	2.17E-66
GTF3C2-AS	ENSG0000(	0.57	42.311	-4.786	4.38E-83
RP11-328K	ENSG0000(	0	4.84	-2.546	4.02E-63
LINC00700	ENSG0000(	0	6.54	-2.915	1.53E-50
RP11-347D	ENSG0000(	0	11.57	-3.652	7.42E-60
RP11-272J	ENSG0000(	0	8.98	-3.319	5.26E-57
RP11-61I1:	ENSG0000(	0.42	5.33	-2.156	3.38E-98
GAPDHP52	ENSG0000(	0.03	5.27	-2.606	7.59E-55
ID2-AS1	ENSG0000(	0.12	4.1	-2.187	4.43E-43
SETP9	ENSG0000(	0.06	3.31	-2.024	5.13E-50

ZSCAN31	ENSG0000(	2.18	34.58	-3.484	2.30E-59
RP3-417L2	ENSG0000(	0	24.271	-4.659	1.09E-69
RP11-374F	ENSG0000(	0.05	10.33	-3.432	1.08E-56
TMEM30C	ENSG0000(	0.07	3.59	-2.101	9.17E-45
LINC01440	ENSG0000(	0	6.14	-2.836	1.75E-48
SMIM1	ENSG0000(	3.66	54.198	-3.566	1.88E-72
PPP1R3E	ENSG0000(	2.94	44.891	-3.542	3.93E-96
LINC01525	ENSG0000(	0	5.2	-2.632	1.62E-61
RP11-121A	ENSG0000(	0	3.99	-2.319	1.74E-40
FAM83C-A	ENSG0000(	0.13	5.08	-2.428	1.15E-49
SMIM2-IT1	ENSG0000(	0	7.03	-3.005	4.37E-61
RP11-399E	ENSG0000(	0	58.858	-5.903	1.34E-80
LINC00961	ENSG0000(	0.3	9.21	-2.973	1.46E-88
RP11-397(	ENSG0000(	0	3.62	-2.208	3.52E-41
TOPORS-A	ENSG0000(	10.22	46.061	-2.068	1.17E-52
RP5-928E2	ENSG0000(	0	4.34	-2.417	2.45E-57
NEURL1-AS	ENSG0000(	0	6	-2.807	9.21E-57
UBE2R2-AS	ENSG0000(	0.04	4.4	-2.376	2.17E-57
JARID2-AS	ENSG0000(	0.18	62.331	-5.746	4.85E-75
RP4-639F2	ENSG0000(	1.41	20.281	-3.142	9.52E-66
HIPK1-AS1	ENSG0000(	0.2	7.65	-2.85	4.66E-79
LINC00533	ENSG0000(	0	3.39	-2.134	8.71E-59
LINC00494	ENSG0000(	0.14	13.28	-3.647	3.68E-55
RNF148	ENSG0000(	0.04	60.141	-5.877	4.02E-70
MIR670HG	ENSG0000(	0	10.43	-3.515	1.48E-76
LINC00993	ENSG0000(	0	5.65	-2.733	3.21E-76
LINC00894	ENSG0000(	1.01	13.7	-2.871	2.64E-78
RP11-342M	ENSG0000(	0	17.09	-4.177	3.07E-56
RP11-436I	ENSG0000(	0	17.28	-4.192	2.94E-67
RP11-634E	ENSG0000(	0.04	3.45	-2.097	2.80E-62
RP11-73M	ENSG0000(	0.11	8.13	-3.04	3.99E-94
LINC00837	ENSG0000(	0.41	16.959	-3.671	2.05E-52
RP11-338(	ENSG0000(	0	6.56	-2.918	4.09E-54
TSPEAR-AS	ENSG0000(	0.25	4.22	-2.062	9.61E-50
TM4SF19-A	ENSG0000(	0.3	9.25	-2.979	6.52E-80
MACROD2	ENSG0000(	0	28.489	-4.882	9.10E-68
LINC01553	ENSG0000(	0	9.51	-3.394	3.02E-57
UBOX5-AS	ENSG0000(	0.03	4.01	-2.282	3.24E-58
MORC2-AS	ENSG0000(	0.32	56.23	-5.438	9.41E-76
PRRX2-AS1	ENSG0000(	0	35.041	-5.172	2.16E-65
LINC00445	ENSG0000(	0	11.36	-3.628	3.14E-58
LL22NC03-	ENSG0000(	0	6.78	-2.96	1.57E-47
LINC01069	ENSG0000(	0	4.88	-2.556	4.16E-50
MIR7515H(	ENSG0000(	0	68.18	-6.112	6.64E-70
SLC25A3P1	ENSG0000(	0.27	16.37	-3.774	1.74E-63
Z82214.3	ENSG0000(	0	13.44	-3.852	2.44E-95
RP5-1099E	ENSG0000(	0	3.78	-2.257	1.56E-45
RP11-340I	ENSG0000(	0	6.15	-2.838	1.25E-61
MRGPRG-A	ENSG0000(	0	11.37	-3.629	3.77E-61

LINC01241	ENSG0000(	0	5	-2.585	3.33E-45
RP3-430A1	ENSG0000(	0	12.5	-3.755	5.45E-63
LINC00854	ENSG0000(	1.74	148.137	-5.766	2.02E-68
RP11-307(	ENSG0000(	0	4.62	-2.491	2.59E-51
LINC01134	ENSG0000(	0.24	7.31	-2.745	5.02E-69
TSPY10	ENSG0000(	0.57	19.49	-3.706	1.15E-06
RP11-79M	ENSG0000(	0.07	3.34	-2.02	2.11E-50
RP11-107N	ENSG0000(	0.31	4.64	-2.106	1.02E-49
UBE2L5P	ENSG0000(	0.33	45.402	-5.125	4.31E-81
LINC00608	ENSG0000(	0	45.521	-5.54	4.61E-71
LINC00427	ENSG0000(	0	5.51	-2.703	5.69E-60
KCNMA1-/	ENSG0000(	0.01	3.63	-2.197	4.47E-54
RP11-66A2	ENSG0000(	0	5.95	-2.797	5.66E-58
KLF2P4	ENSG0000(	0.18	4.93	-2.329	7.78E-51
LINC01424	ENSG0000(	0.33	9.06	-2.919	2.62E-70
RP1-125I3.	ENSG0000(	0	15.5	-4.044	2.80E-29
RP13-254E	ENSG0000(	0.37	15.93	-3.627	2.98E-53
STARD13-.	ENSG0000(	0.08	35.879	-5.094	4.44E-74
LINC00347	ENSG0000(	0	32.96	-5.086	8.89E-68
RPL23AP24	ENSG0000(	0	5.52	-2.705	1.88E-21
LINC01010	ENSG0000(	0.11	10.69	-3.397	7.09E-39
RP11-478H	ENSG0000(	0	5.84	-2.774	1.55E-47
GAGE12B	ENSG0000(	0	183.915	-7.531	4.06E-28
RP11-168F	ENSG0000(	0	5.21	-2.635	8.40E-47
INTS6-AS1	ENSG0000(	1.45	23.321	-3.311	1.10E-115
RP11-383(	ENSG0000(	0	5.13	-2.616	2.29E-45
RP11-978I.	ENSG0000(	0	4.29	-2.403	2.55E-48
RP11-535(	ENSG0000(	0	3.34	-2.118	9.39E-55
LINC00614	ENSG0000(	0.05	15.06	-3.935	6.20E-63
RP11-544A	ENSG0000(	0	12.31	-3.734	2.09E-58
ZEB1-AS1	ENSG0000(	2.11	17.111	-2.542	1.85E-67
NDUFA6-A	ENSG0000(	4.27	51.399	-3.314	2.60E-69
PRMT5-AS	ENSG0000(	0.59	16.36	-3.449	1.18E-81
RP4-669L1	ENSG0000(	21.12	143.419	-2.707	9.68E-35
RP11-415D	ENSG0000(	0.08	8.56	-3.146	5.54E-49
NR2F1-AS1	ENSG0000(	1.36	20.381	-3.179	2.28E-51
ZNF295-AS	ENSG0000(	0.1	34.82	-5.025	4.92E-49
RP11-402F	ENSG0000(	0.11	8.75	-3.135	4.79E-59
LINC00851	ENSG0000(	0	28.509	-4.883	2.95E-68
GS1-124K5	ENSG0000(	3.63	59.821	-3.715	2.04E-69
SYP-AS1	ENSG0000(	0	3.56	-2.189	1.17E-46
RP11-473M	ENSG0000(	0.4	7.3	-2.568	1.04E-21
LINC01304	ENSG0000(	0	40.29	-5.368	1.16E-67
PRSS56	ENSG0000(	0.01	3.25	-2.073	1.84E-42
LINC01522	ENSG0000(	0	5.14	-2.618	1.32E-56
RP11-147(	ENSG0000(	0	32.59	-5.07	2.39E-65
RP11-67L3	ENSG0000(	0	8.57	-3.259	5.44E-57
LINC01351	ENSG0000(	0.24	5.48	-2.386	5.24E-33
RP11-280(	ENSG0000(	0.04	10.02	-3.406	5.14E-60

RP11-206L	ENSG0000(	1.63	29.13	-3.518	2.36E-85
LINC01514	ENSG0000(	0	4.39	-2.43	3.11E-51
RP11-112L	ENSG0000(	0.21	9	-3.047	3.21E-64
RP11-343H	ENSG0000(	0.29	22.441	-4.184	1.14E-71
OXCT2P1	ENSG0000(	0.46	22.99	-4.038	5.52E-82
RP11-406A	ENSG0000(	0	3.53	-2.18	1.21E-46
RP11-690I	ENSG0000(	0.36	8.97	-2.874	2.78E-81
ERICH1-AS	ENSG0000(	0.05	15.87	-4.006	5.79E-75
HCG4P3	ENSG0000(	0.69	7.17	-2.273	6.19E-47
TEX36-AS1	ENSG0000(	0	42.229	-5.434	6.54E-66
RP3-400B1	ENSG0000(	0	3.82	-2.269	4.37E-47
RP3-453P2	ENSG0000(	0	9.04	-3.328	2.23E-58
RP11-483E	ENSG0000(	0	24.939	-4.697	3.61E-75
NFIA-AS1	ENSG0000(	0	60.249	-5.937	8.77E-67
RP1-63G5.	ENSG0000(	0.02	33.941	-5.098	6.30E-68
LINC01430	ENSG0000(	0	13.02	-3.809	2.87E-62
RP11-181E	ENSG0000(	0	7.26	-3.046	6.48E-50
KCNQ1DN	ENSG0000(	0	5.05	-2.597	2.24E-54
RP11-443E	ENSG0000(	0.22	6.51	-2.622	6.64E-61
RP11-38C1	ENSG0000(	0	10.29	-3.497	2.36E-50
PAGR1	ENSG0000(	5.04	54.089	-3.189	1.24E-105
SPATA3-AS	ENSG0000(	0.13	75.792	-6.087	2.42E-68
TSPY6P	ENSG0000(	0.9	27.839	-3.924	1.65E-08
GOLGA2P5	ENSG0000(	1.79	31.979	-3.563	3.93E-66
LINC01589	ENSG0000(	0.06	14.09	-3.831	1.39E-52
RP11-420L	ENSG0000(	0	9.5	-3.392	2.21E-58
RP11-252N	ENSG0000(	0	12.14	-3.716	2.19E-56
PAXBP1-AS	ENSG0000(	0.39	5.07	-2.127	3.13E-88
MPC1L	ENSG0000(	1.11	18.68	-3.221	7.90E-39
RP13-210D	ENSG0000(	0	21.591	-4.498	1.07E-64
MYO5BP2	ENSG0000(	0	8.71	-3.279	1.29E-56
PAGE2B	ENSG0000(	1.06	70.18	-5.111	5.22E-63
HOMER2P2	ENSG0000(	0.1	7.25	-2.907	1.17E-38
TBC1D3P1	ENSG0000(	0	12.15	-3.717	8.02E-71
SNORD97	ENSG0000(	0	3.58	-2.195	1.76E-03
RP11-379K	ENSG0000(	0.1	4.36	-2.285	2.17E-62
RP11-400L	ENSG0000(	0.06	16.62	-4.055	3.98E-70
RPS17P15	ENSG0000(	0	7.57	-3.099	9.83E-56
RP4-584D1	ENSG0000(	0.1	32.751	-4.939	2.72E-62
RP11-634E	ENSG0000(	0	3.24	-2.084	6.67E-49
POM121L7	ENSG0000(	0.01	42.291	-5.422	2.08E-79
LINC01210	ENSG0000(	0	32.05	-5.047	1.86E-72
MYLK-AS1	ENSG0000(	0.9	11.31	-2.696	1.45E-82
LINC00879	ENSG0000(	0.25	4.94	-2.249	8.46E-40
RP11-88I2	ENSG0000(	1.12	7.67	-2.032	1.75E-27
MEIKIN	ENSG0000(	0.22	12.15	-3.43	3.09E-84
MYADML	ENSG0000(	0	17.759	-4.23	1.54E-71
RP11-295F	ENSG0000(	6.24	45.679	-2.689	2.99E-70
RP11-65N1	ENSG0000(	0	4.86	-2.551	4.64E-71

RP11-789F	ENSG0000(	0	4.91	-2.563	7.60E-50
RP11-446H	ENSG0000(	0.11	5.38	-2.523	5.69E-121
IGSF11-AS	ENSG0000(	0	30.17	-4.962	3.05E-65
LINC01471	ENSG0000(	0	6.24	-2.856	3.85E-67
RP11-169N	ENSG0000(	0	7.63	-3.109	4.56E-60
RP11-293A	ENSG0000(	0.68	70.35	-5.408	5.27E-75
TEX35	ENSG0000(	0.05	152.165	-7.189	9.05E-72
RP1-93H18	ENSG0000(	0.06	8.7	-3.194	1.95E-71
SMKR1	ENSG0000(	3.88	307.639	-5.983	5.20E-58
RP11-379F	ENSG0000(	0.47	14.4	-3.389	3.72E-66
RP11-758F	ENSG0000(	0	85.662	-6.437	1.83E-77
RP11-430C	ENSG0000(	0.45	4.87	-2.017	1.58E-68
RP11-146D	ENSG0000(	0.33	20.21	-3.995	2.60E-100
FAR2P3	ENSG0000(	2.28	47.11	-3.875	7.95E-59
TCAM1P	ENSG0000(	0.16	30.721	-4.773	2.49E-86
LA16c-306	ENSG0000(	0.04	10.59	-3.478	2.28E-76
RP11-206L	ENSG0000(	0.34	4.4	-2.011	1.31E-11
SPATA31D	ENSG0000(	0	11.37	-3.629	1.98E-61
RN7SL860F	ENSG0000(	0	9.18	-3.348	2.39E-35
LRRD1	ENSG0000(	0.04	11.37	-3.572	1.58E-60
KRBOX1	ENSG0000(	1.48	271.949	-6.782	1.72E-93
PLCXD2-AS	ENSG0000(	0	4.71	-2.513	6.76E-53
RP5-1002N	ENSG0000(	0	6.16	-2.84	1.94E-47
NSUN6	ENSG0000(	5.66	29.691	-2.204	3.86E-81
RP11-639E	ENSG0000(	0	32.43	-5.063	2.63E-59
RP11-147I	ENSG0000(	0.92	9.6	-2.465	4.21E-50
IQCJ-SCHI	ENSG0000(	0	6.44	-2.895	4.78E-50
RP11-768C	ENSG0000(	0	15.48	-4.043	1.47E-65
RP11-342N	ENSG0000(	0	7.98	-3.167	2.19E-63
LINC01487	ENSG0000(	0	11.31	-3.622	6.62E-58
LINC01192	ENSG0000(	0	12.72	-3.778	3.45E-57
RP11-545C	ENSG0000(	0.45	189.216	-7.035	3.18E-51
LINC00635	ENSG0000(	0.01	28	-4.844	1.65E-65
PTPRG-AS1	ENSG0000(	2.54	15.55	-2.225	3.14E-62
SSX2	ENSG0000(	0.09	27.131	-4.69	2.21E-51
IDS	ENSG0000(	0.94	11.15	-2.647	8.42E-47
RP11-6F2.5	ENSG0000(	0	6.93	-2.987	2.40E-56
ZBTB20-AS	ENSG0000(	0.11	60.012	-5.78	2.14E-70
PAQR9-AS	ENSG0000(	0.11	4.8	-2.385	1.37E-39
PRICKLE2-A	ENSG0000(	0	4.88	-2.556	9.92E-51
RN7SL484F	ENSG0000(	0	3.8	-2.263	8.20E-23
INMT	ENSG0000(	2.25	17.37	-2.499	3.20E-27
TMEM239	ENSG0000(	0.02	17.13	-4.152	4.29E-65
RP11-420J	ENSG0000(	0	7.63	-3.109	1.21E-56
SUMO1P1	ENSG0000(	2.15	15	-2.345	1.60E-28
FABP5P3	ENSG0000(	0.23	6.08	-2.525	5.22E-53
LINC00893	ENSG0000(	1.05	26.921	-3.768	7.94E-90
LA16c-3G1	ENSG0000(	0.04	4.34	-2.36	2.76E-68
RP11-690C	ENSG0000(	0	6.35	-2.878	1.64E-51

RP11-520D	ENSG00000	0	11.04	-3.59	4.75E-53
TCP10L	ENSG00000	0.32	257.244	-7.612	2.32E-87
PVRL3-AS1	ENSG00000	0.23	13.76	-3.585	2.94E-80
STAG3L5P	ENSG00000	10.78	95.121	-3.029	2.32E-66
NPPA-AS1	ENSG00000	2.66	42.329	-3.565	2.34E-69
LINC00901	ENSG00000	0	9.69	-3.418	3.14E-58
RBMY1E	ENSG00000	0	3.6	-2.202	7.65E-55
GTF2A1L	ENSG00000	0.13	48.88	-5.464	1.50E-84
RP3-477O	ENSG00000	0	7.89	-3.152	2.38E-58
LINC01206	ENSG00000	0.01	18.301	-4.256	1.06E-79
RGAG4	ENSG00000	1.87	10.51	-2.004	2.79E-27
LINC00882	ENSG00000	0.56	30.471	-4.334	3.84E-96
GLYCTK-AS	ENSG00000	0.26	8.32	-2.887	1.06E-53
SOX2-OT	ENSG00000	0.49	6.44	-2.32	1.52E-48
ZNF709	ENSG00000	2.48	20.05	-2.597	4.23E-49
STRC	ENSG00000	0.19	18.29	-4.019	1.04E-109
RP11-118N	ENSG00000	0.13	6.4	-2.711	2.25E-69
PLCH1-AS	ENSG00000	0	3.52	-2.176	1.82E-50
RP11-446F	ENSG00000	0	18.47	-4.283	7.41E-72
RP11-556N	ENSG00000	0.65	9.19	-2.627	2.61E-71
RPS11P6	ENSG00000	0.16	28.889	-4.687	2.53E-77
FTH1P18	ENSG00000	0	5.58	-2.718	3.38E-62
RP11-12M	ENSG00000	0	27.41	-4.828	7.96E-69
RP11-538F	ENSG00000	0	12.82	-3.789	4.55E-73
RP11-550I	ENSG00000	0.32	4.96	-2.175	1.61E-72
TUSC7	ENSG00000	0	15.82	-4.072	3.46E-64
LINC00359	ENSG00000	0	3.15	-2.053	1.77E-65
FGF14-IT1	ENSG00000	0.02	6.98	-2.968	1.60E-64
PTPRVP	ENSG00000	0.15	9.4	-3.177	1.07E-94
RP11-445N	ENSG00000	0	3.76	-2.251	2.08E-54
NAT6	ENSG00000	9.03	86.617	-3.127	1.45E-67
WFDC6	ENSG00000	0.38	48.411	-5.162	1.39E-80
LINC01214	ENSG00000	0	7.02	-3.004	4.22E-52
UPK3B	ENSG00000	0.32	4.94	-2.17	4.27E-24
RP11-264K	ENSG00000	0	4.71	-2.513	1.04E-45
RP11-164C	ENSG00000	0	6.96	-2.993	4.06E-73
ZNF487	ENSG00000	2.48	32.981	-3.288	4.01E-63
RP11-307F	ENSG00000	0	42.379	-5.439	2.02E-71
TIPARP-AS	ENSG00000	1.5	59.85	-4.605	4.74E-72
GSTA1	ENSG00000	3.53	175.593	-5.285	6.96E-44
RGAG1	ENSG00000	0.3	9	-2.943	1.69E-87
RP11-368N	ENSG00000	0.63	13.48	-3.151	8.86E-36
RP1-300I2.	ENSG00000	0	5.36	-2.669	1.16E-77
IL12A-AS1	ENSG00000	0.02	3.43	-2.119	1.54E-47
RN7SL417F	ENSG00000	0	3.07	-2.025	5.96E-37
MARK2P17	ENSG00000	0	3.66	-2.22	7.29E-69
GSTA2	ENSG00000	2.2	36.59	-3.554	1.30E-33
FLNB-AS1	ENSG00000	0.34	5.42	-2.26	2.94E-64
FOXP1-AS	ENSG00000	0	6.61	-2.928	2.92E-53

ZFAND6P1	ENSG00000	0	6.5	-2.907	4.24E-64
GS1-259H	ENSG00000	1.11	195.563	-6.542	4.71E-87
LINC01330	ENSG00000	0	10.86	-3.568	1.26E-47
GMCL1P1	ENSG00000	0	7.56	-3.098	8.38E-70
PKD1P1	ENSG00000	1.38	12.87	-2.543	3.43E-22
PEX5L-AS2	ENSG00000	0	28.47	-4.881	5.10E-59
RP11-734K	ENSG00000	0	6.64	-2.934	7.27E-59
HDAC11-A	ENSG00000	0.08	5.34	-2.553	8.58E-56
RP4-800G7	ENSG00000	1.94	34.329	-3.587	5.30E-105
RP11-14D2	ENSG00000	0	3.57	-2.192	1.39E-49
RAD21L1	ENSG00000	0.22	8.71	-2.993	2.69E-58
HBB	ENSG00000	4.5	51.399	-3.252	2.70E-06
LIFR-AS1	ENSG00000	0.39	4.81	-2.063	6.44E-63
RP11-181C	ENSG00000	0.57	5.65	-2.083	3.03E-56
RP11-320N	ENSG00000	0.01	8.2	-3.187	2.64E-53
LINC01024	ENSG00000	1.52	14.95	-2.662	9.86E-55
RNF139-AS	ENSG00000	1.14	10.56	-2.433	5.49E-105
RP11-10K1	ENSG00000	0.36	6.22	-2.408	7.30E-82
USP2-AS1	ENSG00000	0.88	26.31	-3.861	3.33E-60
RP11-15B1	ENSG00000	0.09	5.15	-2.496	1.84E-75
RP11-367J	ENSG00000	0.13	9.75	-3.25	9.66E-73
NEAT1	ENSG00000	78.302	381.171	-2.269	7.47E-30
RORA-AS1	ENSG00000	0.47	8.62	-2.71	6.31E-52
RP11-712E	ENSG00000	0.73	30.33	-4.179	1.26E-99
GS1-24F4.2	ENSG00000	0.16	15.5	-3.83	9.22E-60
FLJ21408	ENSG00000	0.39	9.42	-2.906	8.24E-71
RP11-796E	ENSG00000	0.54	8.39	-2.608	2.01E-70
RP11-33B1	ENSG00000	9.69	51.71	-2.302	1.96E-62
LINC01513	ENSG00000	0	4.43	-2.441	1.73E-55
RAB30-AS1	ENSG00000	5.38	34.36	-2.47	7.01E-66
RP11-696N	ENSG00000	1.11	10.14	-2.4	1.26E-44
LINC01096	ENSG00000	0.13	38.529	-5.129	6.17E-66
RP11-260E	ENSG00000	0.03	3.29	-2.058	2.41E-59
PRR7-AS1	ENSG00000	0.3	10.41	-3.134	8.38E-64
EXTL3-AS1	ENSG00000	0.39	10.66	-3.068	2.59E-93
LACTB2-AS	ENSG00000	0.33	5.69	-2.331	3.92E-54
RP11-461C	ENSG00000	0.2	17.08	-3.913	5.61E-80
RP11-386I	ENSG00000	0.07	4.74	-2.423	1.91E-65
RP11-1277	ENSG00000	0.54	11.14	-2.979	3.04E-83
LINC00535	ENSG00000	0.2	7.5	-2.824	7.56E-68
RASSF8-AS	ENSG00000	2.15	16.04	-2.436	4.17E-26
H2AFJ	ENSG00000	57.11	744.639	-3.682	3.71E-47
RGMB-AS1	ENSG00000	1.41	90.202	-5.242	7.21E-66
RP11-325N	ENSG00000	0.03	5.42	-2.64	3.42E-78
RP1-179N1	ENSG00000	0.52	20.72	-3.837	2.15E-104
RP11-588C	ENSG00000	0	9.43	-3.383	7.65E-60
RP11-727A	ENSG00000	2.51	17.34	-2.385	1.03E-95
RP11-637A	ENSG00000	0.71	9.74	-2.651	3.63E-56
SPTY2D1-A	ENSG00000	0.51	481.135	-8.319	1.65E-83

RP11-1277	ENSG0000(	0.58	13.5	-3.198	9.62E-107
RP11-404k	ENSG0000(	0	19.22	-4.338	1.07E-52
RP11-103J	ENSG0000(	0	6.68	-2.941	1.95E-60
FOXD1-AS	ENSG0000(	0.54	8.37	-2.605	9.14E-34
NRAV	ENSG0000(	2.58	60.459	-4.102	2.39E-67
INSL3	ENSG0000(	1.26	203.487	-6.5	1.11E-38
SLIT2-IT1	ENSG0000(	0	35.941	-5.207	2.27E-67
LINC00616	ENSG0000(	0	11.65	-3.661	2.13E-60
MEF2C-AS	ENSG0000(	0.57	6.84	-2.32	3.31E-77
RP11-366H	ENSG0000(	0	4.04	-2.333	4.46E-81
RP11-313E	ENSG0000(	0.17	13.53	-3.634	1.05E-47
RP11-503N	ENSG0000(	0	21.47	-4.49	4.82E-67
KB-1269D1	ENSG0000(	0.1	5.44	-2.55	2.52E-60
SEMA6A-A	ENSG0000(	1.87	11.99	-2.178	5.87E-67
RP11-517E	ENSG0000(	0.13	14	-3.731	3.30E-69
POU5F2	ENSG0000(	0.05	17.179	-4.114	6.19E-73
MTATP6P1	ENSG0000(	600.782	2412.31	-2.004	3.94E-28
RP11-2O17	ENSG0000(	0	7.06	-3.011	6.07E-56
LINC01339	ENSG0000(	0	7.68	-3.118	1.92E-50
RP11-844F	ENSG0000(	0.33	113.354	-6.426	9.60E-142
RP11-158I1	ENSG0000(	0	8.74	-3.284	3.46E-52
RP11-557J	ENSG0000(	0	9.56	-3.401	5.10E-60
RP11-495K	ENSG0000(	0.05	8.58	-3.19	3.92E-60
RP11-292D	ENSG0000(	0	3.46	-2.157	2.95E-53
WI2-2373I	ENSG0000(	0	30.83	-4.992	7.17E-71
RP11-974F	ENSG0000(	0.16	37.229	-5.042	3.01E-71
LINC01095	ENSG0000(	0	24.35	-4.664	1.38E-69
RP11-412F	ENSG0000(	0.3	6.67	-2.561	2.22E-28
FLJ46284	ENSG0000(	0.05	6.79	-2.891	3.65E-64
RP11-344C	ENSG0000(	0	3.45	-2.154	1.35E-49
RP11-58B2	ENSG0000(	0	3.37	-2.128	5.05E-64
PCNAP1	ENSG0000(	0	6.13	-2.834	8.63E-60
ZNF436-AS	ENSG0000(	1.59	16.39	-2.747	8.14E-84
EPPIN-WFI	ENSG0000(	0.08	23.789	-4.521	7.57E-76
RP11-423H	ENSG0000(	0	8.08	-3.183	8.18E-60
RP11-669N	ENSG0000(	0.16	6.74	-2.738	7.40E-60
RP11-428L	ENSG0000(	0	5.44	-2.687	3.16E-44
LINC01088	ENSG0000(	0.43	8.7	-2.762	2.54E-62
LINC01333	ENSG0000(	0	9.63	-3.41	4.79E-48
LINC01016	ENSG0000(	0.13	138.131	-6.944	1.75E-80
RP11-434D	ENSG0000(	0.03	5.26	-2.603	6.46E-83
RP1-27K12	ENSG0000(	0.06	10.9	-3.489	9.26E-63
RP11-340A	ENSG0000(	0	22.389	-4.548	2.70E-61
RP11-492C	ENSG0000(	0	26.489	-4.781	3.94E-64
RP11-171N	ENSG0000(	0	7.82	-3.141	4.93E-46
SPATS1	ENSG0000(	0.23	25.251	-4.416	7.00E-58
RP11-496H	ENSG0000(	0.84	11.48	-2.762	4.83E-64
RP11-281F	ENSG0000(	0	16.62	-4.139	3.55E-52
RP11-565A	ENSG0000(	0	3.24	-2.084	7.21E-56

MIR143HG	ENSG0000(	0.25	4.27	-2.076	9.12E-25
RP11-279(	ENSG0000(	0.44	6.7	-2.419	1.12E-69
RP11-423H	ENSG0000(	1	14.39	-2.944	2.20E-71
THEGL	ENSG0000(	0.07	5.92	-2.693	3.00E-57
SRD5A3-A	ENSG0000(	1.41	27.851	-3.582	1.87E-61
FER1L5	ENSG0000(	0.03	14.59	-3.92	4.51E-73
RP11-79C(	ENSG0000(	0	10.88	-3.57	2.20E-61
RP11-346J	ENSG0000(	0	4.29	-2.403	2.14E-54
LINC01377	ENSG0000(	0	15.27	-4.024	4.28E-59
LINC00964	ENSG0000(	0.13	39.311	-5.157	2.89E-69
RP11-73G1	ENSG0000(	0	20.941	-4.456	6.19E-55
RP11-420(	ENSG0000(	0	3.95	-2.307	4.06E-60
RP11-983(	ENSG0000(	0	10.57	-3.532	9.53E-62
RP11-665I	ENSG0000(	0	3.63	-2.211	7.37E-49
RP11-495K	ENSG0000(	0.02	6.09	-2.797	1.19E-46
GOLGA8K	ENSG0000(	0.2	10.03	-3.2	2.74E-83
RP11-563E	ENSG0000(	0.1	6.24	-2.719	1.69E-42
SLC7A11-A	ENSG0000(	0.1	10.79	-3.422	3.41E-67
RP11-180(	ENSG0000(	0.09	4.4	-2.309	7.02E-42
RP11-778J	ENSG0000(	0	10.89	-3.572	1.86E-56
YJEFN3	ENSG0000(	4.96	63.249	-3.43	1.11E-66
RP11-577(	ENSG0000(	0	5.16	-2.623	3.09E-52
RP13-401N	ENSG0000(	0.89	16.7	-3.227	1.13E-54
RP11-417F	ENSG0000(	0.03	5.91	-2.746	7.05E-62
RP4-622L5	ENSG0000(	0	16.89	-4.161	3.59E-54
RP11-848(	ENSG0000(	1.01	14.43	-2.94	4.32E-58
RP11-700N	ENSG0000(	0.05	10.32	-3.43	7.64E-83
RP11-480(	ENSG0000(	0	5.76	-2.757	1.20E-54
KIAA1210	ENSG0000(	0.03	25.84	-4.704	2.17E-104
RP11-834(	ENSG0000(	0.14	34.76	-4.971	2.56E-70
WBP1LP2	ENSG0000(	0.24	4.27	-2.087	1.15E-70
RP4-669L1	ENSG0000(	10.16	78.08	-2.825	1.29E-35
SMAD1-AS	ENSG0000(	0	20.32	-4.414	2.91E-60
LINC01511	ENSG0000(	0.06	7.69	-3.035	3.68E-61
RP11-565A	ENSG0000(	0.29	5.35	-2.299	4.32E-43
ROPN1L-A	ENSG0000(	0	11.5	-3.644	4.83E-55
RP11-597D	ENSG0000(	0	189.059	-7.57	2.97E-77
RP11-756F	ENSG0000(	0.07	6.43	-2.796	4.60E-67
XXbac-BPC	ENSG0000(	0	3.8	-2.263	6.53E-49
LINC01596	ENSG0000(	0	16.829	-4.156	7.75E-60
RP11-486L	ENSG0000(	0.07	9.06	-3.233	2.79E-78
RP11-161D	ENSG0000(	0	6.61	-2.928	1.39E-46
RP11-322N	ENSG0000(	0	174.078	-7.452	8.10E-75
NT5C1B-RI	ENSG0000(	0	18.97	-4.32	2.93E-65
RP11-834(	ENSG0000(	1.68	26.7	-3.37	2.91E-27
RP11-386E	ENSG0000(	0	6.58	-2.922	6.78E-55
LINC01365	ENSG0000(	0	3.72	-2.239	5.55E-50
RP11-91H1	ENSG0000(	0	10	-3.459	2.05E-57
RP11-11NS	ENSG0000(	0	3.15	-2.053	4.00E-59

RP11-663F	ENSG0000(	0	4.11	-2.353	1.58E-58
RP11-1338	ENSG0000(	0	8.95	-3.315	8.05E-55
LNX1-AS1	ENSG0000(	0	12.68	-3.774	2.21E-55
RP11-392E	ENSG0000(	0.11	15.51	-3.895	2.21E-76
HMMR-AS	ENSG0000(	0	3.93	-2.302	7.27E-48
RP11-535(	ENSG0000(	0	9.37	-3.374	6.49E-58
RP11-100N	ENSG0000(	0	4.13	-2.359	6.50E-45
RP11-115D	ENSG0000(	0.38	5.21	-2.17	4.17E-39
RP11-419(	ENSG0000(	0	4.24	-2.39	2.93E-65
SEPT14P4	ENSG0000(	0	3.28	-2.098	9.28E-22
RP11-98J2	ENSG0000(	0.07	26.53	-4.685	1.33E-66
F11-AS1	ENSG0000(	0.14	25.04	-4.514	7.55E-60
RP11-45L9	ENSG0000(	0	10.83	-3.564	5.37E-62
LINC01337	ENSG0000(	0.1	8.05	-3.04	5.77E-77
ZNF345	ENSG0000(	3.22	33.219	-3.02	6.43E-69
RP11-73G1	ENSG0000(	0	32.529	-5.067	7.95E-67
RP11-171N	ENSG0000(	0	5.01	-2.587	3.23E-46
RP11-506H	ENSG0000(	0.08	3.59	-2.087	5.59E-51
RP11-506N	ENSG0000(	0	3.59	-2.199	1.72E-48
RP11-328N	ENSG0000(	0	24.729	-4.685	5.24E-75
RP11-420A	ENSG0000(	0.45	5.25	-2.108	7.39E-94
RP11-483A	ENSG0000(	0	4.28	-2.4	3.11E-33
FOXD1	ENSG0000(	0.68	6.33	-2.125	1.24E-34
RP11-197N	ENSG0000(	0.01	5.23	-2.625	4.25E-55
RP11-572(	ENSG0000(	0.06	5.34	-2.58	3.08E-59
RP11-798K	ENSG0000(	0	11.68	-3.664	6.90E-59
MALAT1	ENSG0000(	21.14	160.574	-2.867	5.47E-52
RP11-724(	ENSG0000(	0.12	8.14	-3.029	7.04E-57
RP11-6N13	ENSG0000(	0.04	14.64	-3.911	6.22E-62
RP11-774D	ENSG0000(	0.08	3.8	-2.152	2.94E-47
RP11-128(	ENSG0000(	0.23	4.75	-2.225	3.07E-54
RP11-600L	ENSG0000(	0	4.98	-2.58	4.47E-50
RP11-318I	ENSG0000(	0	8.14	-3.192	8.28E-54
RP11-614F	ENSG0000(	0.9	40.74	-4.457	4.86E-53
RNA5SP21(	ENSG0000(	0	5.6	-2.722	3.11E-04
SCARNA13	ENSG0000(	1.07	8.17	-2.147	1.34E-60
RP11-219J	ENSG0000(	0	25.27	-4.715	1.17E-67
RP11-17A4	ENSG0000(	0	6.65	-2.935	3.54E-58
RP11-369E	ENSG0000(	0	13.71	-3.879	1.28E-61
RP11-863K	ENSG0000(	0	16.44	-4.124	1.07E-62
RP11-421F	ENSG0000(	0	8.69	-3.276	1.09E-59
RP11-809(	ENSG0000(	1.05	7.52	-2.055	3.39E-49
RP11-716D	ENSG0000(	0	5.67	-2.738	2.29E-55
RP11-779(	ENSG0000(	0	13.17	-3.825	3.60E-66
LINC01606	ENSG0000(	0.12	88.818	-6.325	2.61E-90
LINC00293	ENSG0000(	0	10.49	-3.522	1.29E-54
RP11-261(	ENSG0000(	1.95	32.599	-3.51	8.22E-46
RP11-738(	ENSG0000(	0	12.6	-3.766	1.27E-47
EVX1-AS	ENSG0000(	0.79	7.21	-2.197	5.16E-17

RP11-231D	ENSG00000	0.07	79.39	-6.231	7.90E-80
RP11-619L	ENSG00000	0	26.17	-4.764	9.63E-63
RP11-770E	ENSG00000	0	14.18	-3.924	6.25E-70
RP11-697E	ENSG00000	0	16.79	-4.153	6.80E-69
LINC01603	ENSG00000	0	7.12	-3.021	8.21E-56
RP11-114C	ENSG00000	0	3.1	-2.036	4.70E-29
NACA2	ENSG00000	1.43	76.622	-4.997	8.66E-65
SEPT10P1	ENSG00000	0.05	8.75	-3.215	2.92E-64
RP11-26M	ENSG00000	0	4.33	-2.414	2.63E-54
HOXA-AS2	ENSG00000	0.57	11.74	-3.021	3.04E-34
RP11-108C	ENSG00000	0	30.41	-4.973	6.51E-67
RP11-100L	ENSG00000	0	4.83	-2.543	5.50E-54
RP11-536N	ENSG00000	0	16.781	-4.152	1.49E-63
RP11-587H	ENSG00000	0	4.04	-2.333	6.71E-61
LINCR-000	ENSG00000	0.17	4.66	-2.274	1.22E-44
RP11-317N	ENSG00000	0.07	6.01	-2.712	5.63E-72
RP11-561E	ENSG00000	0	37.439	-5.264	1.10E-67
PRSS51	ENSG00000	0.14	4.67	-2.314	8.18E-29
RP11-981G	ENSG00000	0	29.331	-4.923	3.27E-64
KB-1410C5	ENSG00000	0	17.421	-4.203	1.05E-76
RP11-177H	ENSG00000	0.1	11.11	-3.461	5.78E-70
RP11-787D	ENSG00000	0	7.77	-3.133	1.08E-54
KB-1083B1	ENSG00000	0	18.23	-4.265	1.78E-62
RP11-404L	ENSG00000	0	25.56	-4.731	2.44E-65
LINC01030	ENSG00000	0.03	4.26	-2.352	7.89E-61
RP11-1415	ENSG00000	5.98	64.239	-3.224	3.04E-58
RP11-705C	ENSG00000	0	3.15	-2.053	1.57E-48
RP11-386G	ENSG00000	0	5.89	-2.784	5.16E-55
FER1L6-AS	ENSG00000	0.02	3.7	-2.204	5.11E-67
RP11-347C	ENSG00000	0.71	6.43	-2.119	2.40E-74
RP11-10A1	ENSG00000	0	4.4	-2.433	1.55E-52
IGHVII-44-	ENSG00000	0	7.74	-3.128	1.27E-33
RP11-410L	ENSG00000	1.45	18.46	-2.99	6.11E-73
RP11-4K16	ENSG00000	0	11.48	-3.642	1.06E-63
NRG1-IT1	ENSG00000	0.21	29.889	-4.674	2.44E-66
RP11-363E	ENSG00000	0.32	68.171	-5.712	9.72E-72
RP11-369E	ENSG00000	0	13.52	-3.86	2.35E-62
RP11-150C	ENSG00000	0.04	7.48	-3.027	1.69E-78
RP11-705C	ENSG00000	0	54.871	-5.804	9.56E-70
RP11-99H2	ENSG00000	0	4.31	-2.409	1.27E-47
RP11-787D	ENSG00000	0	5.37	-2.671	3.55E-55
KB-1043D8	ENSG00000	0	5.66	-2.736	1.91E-54
RP11-115J	ENSG00000	0.04	10.54	-3.472	5.06E-72
RP11-108C	ENSG00000	0	6.33	-2.874	4.22E-50
RP11-17A4	ENSG00000	0.01	5.07	-2.587	7.96E-66
RP11-58O3	ENSG00000	0	10.34	-3.503	1.24E-49
RP11-24P4	ENSG00000	0	5.03	-2.592	1.02E-60
RP11-10A1	ENSG00000	0.36	28.58	-4.443	6.94E-52
RP11-628E	ENSG00000	0	9.59	-3.405	5.31E-61

RP11-343F	ENSG0000(	0	3.12	-2.043	2.45E-50
RP11-677I	ENSG0000(	0.1	8.72	-3.143	1.27E-72
RP11-560(	ENSG0000(	0	12.89	-3.796	2.14E-60
RP11-755F	ENSG0000(	0.55	29.97	-4.321	2.36E-87
RP11-380(	ENSG0000(	0.53	7.48	-2.47	4.40E-63
FBXO3-AS:	ENSG0000(	0	13.35	-3.843	5.27E-69
RP11-958J	ENSG0000(	0	14.97	-3.997	2.28E-48
LINC01493	ENSG0000(	0	3.2	-2.07	5.48E-48
RP1-17K7.:	ENSG0000(	0	30.93	-4.997	3.78E-71
RP11-395(	ENSG0000(	0.87	6.76	-2.053	1.27E-40
RP11-354A	ENSG0000(	0.09	5.97	-2.677	6.97E-80
RP11-1415	ENSG0000(	0.89	13.88	-2.977	3.75E-48
RP11-680E	ENSG0000(	0.02	3.14	-2.021	4.00E-43
RP11-496I	ENSG0000(	1.37	37.17	-4.009	1.15E-76
RP11-109E	ENSG0000(	0	30.06	-4.957	1.36E-65
RP1-17K7.:	ENSG0000(	0	12.44	-3.748	1.54E-60
RP11-574N	ENSG0000(	0.92	14.63	-3.025	2.79E-44
NPIPA2	ENSG0000(	0.22	4.15	-2.078	1.13E-31
RP11-867(	ENSG0000(	0.14	23.18	-4.407	3.51E-93
RP11-831A	ENSG0000(	0	18.301	-4.271	4.44E-63
SCARNA9	ENSG0000(	0.58	6.57	-2.26	4.25E-68
RP11-677N	ENSG0000(	0.46	4.85	-2.002	3.18E-64
KIRREL3-A:	ENSG0000(	0	26.711	-4.792	5.58E-70
KRTAP5-9	ENSG0000(	0.09	4.2	-2.254	5.36E-66
RP11-23J9	ENSG0000(	0.3	9.17	-2.968	1.12E-102
RP11-347H	ENSG0000(	3.66	58.989	-3.686	3.55E-47
RP11-712L	ENSG0000(	0.54	8.62	-2.643	2.15E-45
ZFP91-CN	ENSG0000(	0	3.61	-2.205	1.38E-46
RP11-495(	ENSG0000(	0	3.82	-2.269	1.79E-43
ZNF286A	ENSG0000(	0.11	19.91	-4.236	1.06E-77
RP5-1027(	ENSG0000(	0	4.76	-2.526	4.02E-49
OVOL1-AS	ENSG0000(	0	9.53	-3.396	2.51E-59
RP11-646J	ENSG0000(	0	3.49	-2.167	1.03E-52
STX17-AS1	ENSG0000(	0.76	6.17	-2.026	3.73E-41
TOLLIP-AS	ENSG0000(	0.5	7.27	-2.463	8.92E-113
RP11-318(	ENSG0000(	0	5.24	-2.642	3.90E-50
RP11-670N	ENSG0000(	0	5.14	-2.618	3.63E-51
RP11-166D	ENSG0000(	1.5	14.78	-2.658	1.60E-19
TCEB2P2	ENSG0000(	1.36	26.359	-3.535	2.81E-54
RP11-702F	ENSG0000(	0	7.66	-3.114	4.96E-65
RP11-375D	ENSG0000(	0	7.16	-3.029	4.82E-53
WTAPP1	ENSG0000(	0.1	4.04	-2.196	1.46E-43
RP11-1082	ENSG0000(	0	3.69	-2.23	1.71E-54
LINC01495	ENSG0000(	0	3.43	-2.147	4.71E-46
RP11-201N	ENSG0000(	0	10.53	-3.527	1.86E-60
RP11-358H	ENSG0000(	0	21.13	-4.468	8.29E-59
RP11-469N	ENSG0000(	0	3.36	-2.124	9.39E-59
NOX5	ENSG0000(	0.08	6.83	-2.858	5.41E-96
RP11-539(	ENSG0000(	0.69	39.401	-4.579	2.05E-93

SEPT14P8	ENSG0000(	0.14	5.36	-2.48	2.84E-03
RP11-535A	ENSG0000(	0.11	38.759	-5.163	4.62E-75
NPIPB8	ENSG0000(	0.31	18.6	-3.903	5.61E-82
NEDD8-M	ENSG0000(	1.19	11.61	-2.526	8.59E-42
RP11-168K	ENSG0000(	0	4.27	-2.398	1.25E-47
RP11-810F	ENSG0000(	0	3.81	-2.266	6.47E-52
RP11-6B19	ENSG0000(	0	3.3	-2.104	2.87E-38
LINC00925	ENSG0000(	0.85	39.601	-4.456	3.28E-61
RP11-273E	ENSG0000(	0.4	6.61	-2.442	2.39E-39
RP11-188C	ENSG0000(	0.19	6.98	-2.745	1.90E-87
RP11-502N	ENSG0000(	0	62.361	-5.986	3.75E-74
FAM222A-	ENSG0000(	0	13.38	-3.846	2.74E-66
RP11-867C	ENSG0000(	0	5.31	-2.658	1.91E-34
RP11-711K	ENSG0000(	0	7.06	-3.011	2.09E-56
RP11-407A	ENSG0000(	0	7.69	-3.119	6.86E-63
RP11-728C	ENSG0000(	0.26	5.43	-2.351	6.71E-39
RP11-526F	ENSG0000(	0	11.85	-3.684	5.02E-55
RP13-942N	ENSG0000(	2.55	21.23	-2.647	4.61E-57
RP11-319E	ENSG0000(	0.06	19.05	-4.241	1.41E-62
RP11-778H	ENSG0000(	0	21.22	-4.474	3.98E-61
LINC00944	ENSG0000(	0.12	31.36	-4.853	9.26E-70
RP11-504C	ENSG0000(	0.19	13.44	-3.601	1.23E-72
RP3-446N1	ENSG0000(	0	4.82	-2.541	1.92E-56
RP11-106C	ENSG0000(	0.14	8.14	-3.003	2.11E-35
RP11-705C	ENSG0000(	0.1	56.539	-5.709	2.37E-67
RP13-653N	ENSG0000(	0	10.05	-3.466	4.91E-62
LINC01486	ENSG0000(	0	14.43	-3.948	7.44E-66
RP11-522N	ENSG0000(	0.04	3.4	-2.081	4.45E-51
RP11-49K4	ENSG0000(	0	3.03	-2.011	1.78E-36
RP11-727F	ENSG0000(	1.33	9.46	-2.166	2.17E-56
RP11-669N	ENSG0000(	0	3.69	-2.23	6.75E-47
RP11-407A	ENSG0000(	0	31.919	-5.041	4.76E-67
RP13-941N	ENSG0000(	0.52	16.35	-3.513	4.69E-58
RP11-3L23	ENSG0000(	0	23.079	-4.59	2.61E-64
RP11-7M8	ENSG0000(	0	4	-2.322	1.92E-52
RP11-664D	ENSG0000(	0.07	20.68	-4.341	6.83E-56
RP13-81N3	ENSG0000(	0	11.91	-3.69	1.43E-57
RP11-474D	ENSG0000(	0	6.57	-2.92	5.89E-62
RP11-783K	ENSG0000(	1.46	40.229	-4.067	1.80E-58
RP11-881M	ENSG0000(	0	19.401	-4.351	1.01E-67
RP11-268F	ENSG0000(	0.22	6.57	-2.633	2.06E-47
RP11-783K	ENSG0000(	0.37	750.183	-9.099	1.09E-81
RP11-123C	ENSG0000(	0	5	-2.585	1.79E-48
RP11-783K	ENSG0000(	1.15	25.47	-3.622	5.31E-88
RP11-554D	ENSG0000(	0	4.05	-2.336	5.30E-54
RP11-320N	ENSG0000(	0.48	5.52	-2.139	3.64E-60
TMPO-AS1	ENSG0000(	3.01	21.55	-2.492	3.33E-37
FAM205BP	ENSG0000(	0	10.83	-3.564	4.58E-61
RP11-669E	ENSG0000(	0	3.45	-2.154	2.52E-62

RP11-983F	ENSG0000(	2.44	15.07	-2.224	1.60E-43
RP11-762I	ENSG0000(	1.77	15.44	-2.569	1.68E-46
RP11-310I:	ENSG0000(	0	3.19	-2.067	1.28E-44
RP11-121(	ENSG0000(	0	6.87	-2.976	1.09E-53
RP11-175F	ENSG0000(	0.36	4.88	-2.112	6.08E-61
RP11-1016	ENSG0000(	0.01	5.72	-2.734	2.52E-92
RP11-486A	ENSG0000(	0.81	8.12	-2.333	1.31E-35
NPIPB1P	ENSG0000(	0.03	17.859	-4.195	2.31E-64
RP11-755(	ENSG0000(	0	11.81	-3.679	1.90E-59
OR7E47P	ENSG0000(	1.02	10.25	-2.477	2.97E-47
OVCH1-AS	ENSG0000(	0.75	16.49	-3.321	3.07E-39
RP11-100F	ENSG0000(	0	9.2	-3.351	7.12E-53
RP11-384J	ENSG0000(	0.04	7.87	-3.092	4.82E-52
LINC01481	ENSG0000(	0.37	9.28	-2.908	2.59E-66
RP11-175F	ENSG0000(	0	4.34	-2.417	7.83E-53
RP11-620J	ENSG0000(	14.78	296.584	-4.237	5.91E-76
RP11-482D	ENSG0000(	0.16	20.629	-4.221	1.17E-76
RP11-396F	ENSG0000(	0.65	9.06	-2.608	6.18E-39
LINC01490	ENSG0000(	0	5.82	-2.77	1.86E-52
MGAM2	ENSG0000(	0.05	3.26	-2.02	4.77E-32
RP11-966I	ENSG0000(	0	5.03	-2.592	2.51E-52
RP11-474L	ENSG0000(	0.05	9.06	-3.26	3.86E-63
RP11-70F1	ENSG0000(	0	22.841	-4.575	1.88E-62
RP11-630(	ENSG0000(	0	45.559	-5.541	3.28E-63
RP11-552I:	ENSG0000(	0	11.71	-3.668	1.50E-58
RP1-90J4.1	ENSG0000(	0	14.39	-3.944	6.93E-81
RP11-167N	ENSG0000(	0	5.88	-2.782	6.22E-51
LINC00935	ENSG0000(	0.83	31.471	-4.149	3.67E-41
RP11-100F	ENSG0000(	0	8.18	-3.199	9.24E-52
RP11-543H	ENSG0000(	0	4.84	-2.546	1.34E-44
RP11-781A	ENSG0000(	0.1	11.69	-3.528	2.05E-58
RP11-328(	ENSG0000(	0	4.61	-2.488	5.62E-49
RP11-474F	ENSG0000(	0.04	5.93	-2.736	1.38E-63
RP11-89K2	ENSG0000(	0	5.17	-2.625	3.53E-51
RP11-347(	ENSG0000(	0.84	57.071	-4.98	3.97E-63
RP11-70F1	ENSG0000(	0	10.95	-3.579	1.21E-57
RP11-864J	ENSG0000(	0.13	4.28	-2.224	3.21E-57
RP11-345J	ENSG0000(	0.42	4.96	-2.069	1.65E-31
RP11-977(	ENSG0000(	1.42	9.86	-2.166	2.13E-57
RP11-38F2	ENSG0000(	0	6.96	-2.993	1.88E-58
PRSS58	ENSG0000(	0	30.67	-4.985	5.88E-61
RP11-162N	ENSG0000(	0	7.07	-3.013	8.59E-39
RP11-219E	ENSG0000(	0.56	11.41	-2.992	5.01E-37
LINC00592	ENSG0000(	0	4.49	-2.457	2.27E-56
TESC-AS1	ENSG0000(	0	7.76	-3.131	7.82E-62
RP11-536(	ENSG0000(	0.07	5.35	-2.569	3.83E-51
RP11-107(	ENSG0000(	0	5.52	-2.705	1.03E-75
RP11-300J	ENSG0000(	0	3.27	-2.094	6.84E-43
RP11-665(	ENSG0000(	0	17.34	-4.197	2.41E-50

RP11-486(	ENSG0000(	0	5.39	-2.676	7.40E-52
RP11-452I	ENSG0000(	0	3.41	-2.141	1.80E-43
RP11-164J	ENSG0000(	1.07	12.25	-2.678	3.15E-94
RP11-388E	ENSG0000(	0	3.67	-2.223	1.67E-46
RP11-76E1	ENSG0000(	0.03	6.84	-2.928	6.42E-63
SPESP1	ENSG0000(	6.13	56.219	-3.005	2.56E-44
RP11-99L1	ENSG0000(	0	3.51	-2.173	9.26E-47
RP11-783L	ENSG0000(	0	4.54	-2.47	3.26E-47
RP11-185F	ENSG0000(	0	3.67	-2.223	2.12E-51
RP11-829H	ENSG0000(	0.15	7.55	-2.894	5.13E-44
RP11-111A	ENSG0000(	0.12	10.6	-3.373	1.37E-59
RP11-753I	ENSG0000(	0	4.22	-2.384	6.40E-45
RP11-14J7	ENSG0000(	0.01	6.47	-2.887	4.12E-54
LINC01500	ENSG0000(	0	10.87	-3.569	1.17E-62
FAM181A-	ENSG0000(	0	16.27	-4.11	1.62E-60
NBEAP1	ENSG0000(	0.12	4.67	-2.34	8.06E-38
RP11-219E	ENSG0000(	0	18.52	-4.287	3.30E-52
LINC00930	ENSG0000(	0.05	84.401	-6.346	7.20E-82
RP11-509A	ENSG0000(	0.21	5.05	-2.322	1.16E-53
RP11-386M	ENSG0000(	0.02	6.11	-2.801	8.92E-67
LINC01193	ENSG0000(	0	17.81	-4.233	3.34E-67
RP11-661(	ENSG0000(	0	5.35	-2.667	9.46E-54
PRC1-AS1	ENSG0000(	0.15	33.219	-4.895	1.04E-67
RP11-547I	ENSG0000(	0.06	10.52	-3.442	5.94E-58
RP11-603E	ENSG0000(	2.27	28.13	-3.155	1.47E-48
RP11-112J	ENSG0000(	0.17	4.56	-2.249	3.66E-66
RP11-73E1	ENSG0000(	0.46	9.9	-2.9	9.99E-60
RP11-862(	ENSG0000(	0	11.17	-3.605	7.87E-63
RP11-163M	ENSG0000(	0	10.19	-3.484	2.82E-55
LINC01579	ENSG0000(	0.77	10.07	-2.645	4.80E-46
LINC01568	ENSG0000(	0	4.78	-2.531	1.48E-42
LINC01580	ENSG0000(	0.11	3.52	-2.026	8.25E-46
RP11-561E	ENSG0000(	0.33	5.99	-2.394	1.63E-70
RNASE4	ENSG0000(	1.8	12.63	-2.283	5.68E-19
RP11-300J	ENSG0000(	0	3.39	-2.134	9.64E-52
RP11-123K	ENSG0000(	0.17	4.43	-2.214	6.21E-52
MC1R	ENSG0000(	3.61	37.519	-3.063	1.33E-80
RP11-286(	ENSG0000(	0	33.18	-5.095	2.15E-60
RP11-232(	ENSG0000(	0	26.729	-4.793	2.70E-50
RP5-1021I	ENSG0000(	0.01	9.27	-3.346	5.16E-60
RP11-164(	ENSG0000(	0	3.41	-2.141	6.46E-48
RP11-463J	ENSG0000(	0	8.21	-3.203	2.64E-66
RP11-857E	ENSG0000(	0.11	14.31	-3.786	5.69E-80
RP11-363J	ENSG0000(	0.2	9.46	-3.124	6.21E-70
RP11-1017	ENSG0000(	0.53	7.85	-2.532	3.48E-55
LINC01467	ENSG0000(	0.02	7	-2.971	5.08E-55
TSPY1	ENSG0000(	0.4	9.92	-2.963	5.07E-06
RP11-2C7.	ENSG0000(	0	4.6	-2.485	1.48E-53
RNASE11	ENSG0000(	0	12.84	-3.791	1.58E-59

RP11-371E	ENSG0000(	0	4.45	-2.446	7.72E-26
RP11-168L	ENSG0000(	0	29.539	-4.933	1.90E-65
RP11-603E	ENSG0000(	0.47	5.08	-2.048	3.15E-47
LINC00911	ENSG0000(	0	6.74	-2.952	2.72E-59
RP11-973N	ENSG0000(	0.05	8.32	-3.15	4.10E-74
RP11-840I:	ENSG0000(	0.08	7.18	-2.921	3.39E-66
SMIM6	ENSG0000(	0.12	33.681	-4.953	1.78E-54
RP1-261D:	ENSG0000(	0.53	8.71	-2.666	8.51E-65
LINC00929	ENSG0000(	0	27.729	-4.844	2.49E-55
RP11-1078	ENSG0000(	0.02	10.48	-3.492	1.17E-55
RP11-763K	ENSG0000(	0	8.1	-3.186	2.30E-62
RP11-69H:	ENSG0000(	0	61.341	-5.962	3.30E-72
RP11-810K	ENSG0000(	0	39.45	-5.338	3.22E-79
SLC35G6	ENSG0000(	0.02	6.2	-2.819	1.96E-62
RP11-182J	ENSG0000(	1.32	9.46	-2.173	8.13E-40
RP11-684E	ENSG0000(	0	22.27	-4.54	1.39E-70
USP3-AS1	ENSG0000(	1.07	26.199	-3.716	2.03E-72
RP11-643N	ENSG0000(	0.05	4.34	-2.346	5.71E-75
RP11-624L	ENSG0000(	0	7.61	-3.106	3.22E-48
LINGO1-AS	ENSG0000(	0	12.59	-3.764	5.53E-64
SPATA8-AS	ENSG0000(	0	7.53	-3.093	8.51E-59
PAK6	ENSG0000(	1.64	13.73	-2.48	1.31E-48
RP11-687N	ENSG0000(	0	3.93	-2.302	4.47E-45
RP11-183E	ENSG0000(	0	3.06	-2.021	4.67E-46
OR11K1P	ENSG0000(	0	6.72	-2.949	1.83E-58
RP11-798K	ENSG0000(	0.14	3.7	-2.044	9.21E-39
RP11-15E1	ENSG0000(	0.31	4.51	-2.072	2.29E-46
LINC00927	ENSG0000(	0	56.308	-5.841	5.60E-73
RP11-605F	ENSG0000(	0.06	4.15	-2.281	9.88E-71
RP11-27M	ENSG0000(	0	5.17	-2.625	3.76E-47
RP11-566K	ENSG0000(	0.11	54.071	-5.633	4.92E-72
THTPA	ENSG0000(	4.58	29.311	-2.441	1.29E-54
RP11-462F	ENSG0000(	0	10.83	-3.564	7.71E-57
RORA-AS2	ENSG0000(	0	7.66	-3.114	1.07E-56
UBE2Q2L	ENSG0000(	0.18	10.32	-3.262	9.63E-64
RP11-526I:	ENSG0000(	0.13	5.65	-2.557	3.17E-59
LINC01491	ENSG0000(	0	7.34	-3.06	1.14E-58
RP11-66B2	ENSG0000(	0.74	7.47	-2.283	3.87E-74
RBM17P4	ENSG0000(	0	3.63	-2.211	2.99E-61
RP11-463I:	ENSG0000(	0.08	4.38	-2.317	7.48E-54
RP11-554D	ENSG0000(	0	13.17	-3.825	3.53E-67
LINC01582	ENSG0000(	0	4.92	-2.566	3.82E-79
RP11-643C	ENSG0000(	0.09	3.58	-2.071	2.78E-65
RP11-89K1	ENSG0000(	3.35	32.159	-2.93	1.21E-59
LINGO1-AS	ENSG0000(	0.02	16.59	-4.108	4.89E-65
RP11-299H	ENSG0000(	0	6.03	-2.814	1.98E-67
RP11-485C	ENSG0000(	0	18.83	-4.31	1.50E-63
RP11-762H	ENSG0000(	0	6.83	-2.969	4.19E-59
LINC00677	ENSG0000(	0	7.64	-3.111	2.58E-57

RP11-348E	ENSG0000(	0	3.33	-2.114	4.25E-56
LINC00933	ENSG0000(	0.18	9.42	-3.143	2.92E-90
ZNF444P1	ENSG0000(	0.36	8.42	-2.792	3.37E-66
RP11-1006	ENSG0000(	0.2	30.849	-4.73	2.59E-77
RP11-53L2	ENSG0000(	0	3.77	-2.254	2.63E-48
LINC00567	ENSG0000(	0.01	8.34	-3.209	7.90E-82
PWRN1	ENSG0000(	1.33	85.36	-5.212	5.28E-69
RP11-932(	ENSG0000(	0	4.96	-2.575	1.32E-56
RP6-91H8.	ENSG0000(	0.04	53.861	-5.721	3.10E-71
RP11-218N	ENSG0000(	0.18	13.62	-3.631	6.43E-53
RP11-553L	ENSG0000(	2.28	16.91	-2.449	1.42E-48
RP11-480I.	ENSG0000(	0	11.5	-3.644	5.90E-72
LA16c-390	ENSG0000(	0.59	5.59	-2.051	6.51E-67
GOLGA2P1	ENSG0000(	0	19.01	-4.323	7.93E-84
RP11-2C24	ENSG0000(	0.04	6.23	-2.797	1.79E-60
RP11-33B1	ENSG0000(	2.47	21.97	-2.727	4.23E-48
RP11-568N	ENSG0000(	0.36	8.05	-2.734	4.66E-60
LA16c-312	ENSG0000(	0	7.12	-3.021	1.86E-54
RP11-109F	ENSG0000(	0	3.04	-2.014	1.68E-59
RP11-83N9	ENSG0000(	0.99	8.9	-2.315	2.94E-51
RP11-441F	ENSG0000(	0.07	3.35	-2.023	8.50E-57
RP3-333B1	ENSG0000(	0	6.03	-2.814	1.16E-62
RP11-303E	ENSG0000(	0.15	15.401	-3.834	2.82E-69
WI2-2610K	ENSG0000(	0.04	46.549	-5.515	3.17E-62
RP11-368I	ENSG0000(	0.14	8.91	-3.12	1.35E-65
LINC00919	ENSG0000(	0	58.94	-5.905	5.05E-70
RP11-101E	ENSG0000(	0.28	15.44	-3.683	7.09E-62
ITFG1-AS1	ENSG0000(	0.29	84.278	-6.047	1.57E-78
TBC1D3G	ENSG0000(	0	28.019	-4.859	6.17E-58
RP11-24M	ENSG0000(	0.26	16.66	-3.809	4.64E-65
RP11-505K	ENSG0000(	1.03	19.59	-3.342	4.16E-70
NTRK3-AS:	ENSG0000(	0	7.92	-3.157	3.15E-54
HSPB9	ENSG0000(	0.26	17.01	-3.837	9.09E-66
LINC01570	ENSG0000(	0	27.63	-4.839	1.69E-70
RP11-1035	ENSG0000(	0.32	15.58	-3.651	3.50E-74
LINC01043	ENSG0000(	0	3.02	-2.007	2.14E-43
RP11-56L1	ENSG0000(	0	4.41	-2.436	3.30E-58
RP1-155D2	ENSG0000(	0	5.47	-2.694	8.20E-62
RP13-735L	ENSG0000(	0.35	5.95	-2.364	1.84E-50
RP11-202D	ENSG0000(	0	12.81	-3.788	2.98E-63
RP11-96D1	ENSG0000(	0	12.01	-3.702	8.37E-75
LCMT1-AS	ENSG0000(	0.66	7.07	-2.281	6.18E-78
RP4-561L2	ENSG0000(	0.17	4.65	-2.272	5.43E-98
RP11-85A1	ENSG0000(	0.13	7.09	-2.84	6.42E-91
LINC01584	ENSG0000(	0	6.82	-2.967	1.39E-58
RP11-109E	ENSG0000(	0	8.71	-3.279	6.40E-64
RP11-510N	ENSG0000(	0	7.79	-3.136	2.34E-58
PWRN2	ENSG0000(	0	19.03	-4.324	6.13E-68
RP11-24N1	ENSG0000(	17.67	97.18	-2.395	9.69E-16

RP11-491F	ENSG0000(	0.43	5.07	-2.086	2.68E-67
RP11-327F	ENSG0000(	0	5.44	-2.687	1.54E-50
RP11-24D:	ENSG0000(	0	16.59	-4.137	7.43E-57
RP11-452L	ENSG0000(	0.25	5.07	-2.28	4.35E-79
RP11-303E	ENSG0000(	0.74	8.38	-2.43	3.24E-63
RP11-114(	ENSG0000(	0.24	8.77	-2.978	8.36E-41
RP11-439E	ENSG0000(	0.12	4.85	-2.385	1.08E-55
RP11-165N	ENSG0000(	0	3.37	-2.128	4.00E-43
LINC01227	ENSG0000(	0	26.221	-4.767	2.23E-62
RP11-482N	ENSG0000(	0.07	3.38	-2.033	1.48E-62
RP11-114H	ENSG0000(	0.13	12.88	-3.619	9.13E-77
RP11-872J	ENSG0000(	1.12	7.5	-2.003	2.28E-51
RP11-1437	ENSG0000(	0	5.77	-2.759	1.02E-56
RP11-524(	ENSG0000(	0.28	15.23	-3.664	3.17E-71
RP11-80F2	ENSG0000(	0.18	36.141	-4.976	7.25E-78
LINC01229	ENSG0000(	0.2	8.1	-2.923	2.57E-76
TAT-AS1	ENSG0000(	0.24	18.47	-3.973	1.13E-65
RP11-380D	ENSG0000(	0	5.07	-2.602	2.70E-66
RP11-483F	ENSG0000(	0	7.32	-3.057	1.80E-62
RP11-548N	ENSG0000(	0	5.49	-2.698	3.34E-47
RP11-30L1	ENSG0000(	0.4	16.95	-3.68	5.98E-60
RP11-297L	ENSG0000(	0	7	-3	6.02E-48
RP11-190D	ENSG0000(	0	4.13	-2.359	7.70E-52
RP11-854K	ENSG0000(	0.35	6.35	-2.445	5.97E-39
RP11-546E	ENSG0000(	0	7.88	-3.151	4.41E-62
RP11-96C2	ENSG0000(	0.24	4.05	-2.026	1.27E-70
RP11-370F	ENSG0000(	0	5.63	-2.729	6.01E-52
GS1-279B7	ENSG0000(	0	4.53	-2.467	8.29E-50
RP1-149C7	ENSG0000(	0.05	3.96	-2.24	6.84E-66
RP11-481J	ENSG0000(	0	3.23	-2.081	8.37E-38
RP1-166H4	ENSG0000(	0	5.32	-2.66	1.25E-58
LMO7-AS1	ENSG0000(	0.34	4.61	-2.066	2.25E-29
RP11-496D	ENSG0000(	0.06	4.83	-2.459	9.84E-77
RP11-17M	ENSG0000(	1.9	37.639	-3.736	1.92E-28
RP4-798A1	ENSG0000(	0.81	7.68	-2.262	6.53E-50
RP11-697E	ENSG0000(	0.14	11.19	-3.419	1.43E-76
RP11-215E	ENSG0000(	0.03	11.51	-3.602	1.51E-49
RP11-723(	ENSG0000(	0.77	9.08	-2.51	3.74E-74
RP11-68I1:	ENSG0000(	0.31	6	-2.418	4.51E-76
RP11-1136	ENSG0000(	0	32.03	-5.046	8.92E-66
RP11-2L4.1	ENSG0000(	0	3.67	-2.223	5.06E-46
RNF126P1	ENSG0000(	0.63	17.57	-3.51	4.75E-50
RP3-496C2	ENSG0000(	0.07	11.04	-3.492	1.09E-71
LA16c-361	ENSG0000(	0.14	5.33	-2.473	1.78E-86
RP11-304L	ENSG0000(	0.22	11.22	-3.324	1.48E-63
GOLGA8T	ENSG0000(	0.11	8.32	-3.07	1.75E-70
RP3-388M	ENSG0000(	0.2	4.42	-2.175	2.14E-72
RP11-760D	ENSG0000(	0.01	22.86	-4.562	2.75E-83
ULK4P1	ENSG0000(	0.7	6.81	-2.2	2.69E-44

RP11-299H	ENSG0000(	0.01	6.97	-2.98	8.91E-66
RP11-343H	ENSG0000(	0.07	4.32	-2.314	7.91E-83
RP11-160C	ENSG0000(	0	13.68	-3.876	6.12E-83
RP11-653J	ENSG0000(	0	5.29	-2.653	7.39E-63
RP11-279C	ENSG0000(	0.73	7.56	-2.307	1.17E-32
PDCD6IPP2	ENSG0000(	2.44	25.76	-2.96	1.89E-51
RP11-60L3	ENSG0000(	0	3.09	-2.032	2.69E-46
RP11-165N	ENSG0000(	0	41.85	-5.421	5.07E-65
RP11-244E	ENSG0000(	0	5.13	-2.616	9.79E-59
RP11-114H	ENSG0000(	0	8.2	-3.202	5.29E-62
RP11-326A	ENSG0000(	0	38.539	-5.305	1.21E-74
RP11-1007	ENSG0000(	0.39	7.13	-2.548	1.75E-71
RP11-217E	ENSG0000(	0.24	14.53	-3.647	4.63E-67
RP11-589C	ENSG0000(	0	3.11	-2.039	1.63E-62
RP11-626C	ENSG0000(	0.66	12.97	-3.073	1.73E-57
RP11-152C	ENSG0000(	0	12.06	-3.707	1.89E-66
PAN3-AS1	ENSG0000(	0.6	8.13	-2.513	5.52E-95
TP53TG3B	ENSG0000(	0.42	12.01	-3.196	2.12E-52
RP11-527L	ENSG0000(	0.38	9.52	-2.93	1.68E-46
RP11-665J	ENSG0000(	0	4.79	-2.534	1.32E-51
RP11-457N	ENSG0000(	0.52	13.61	-3.265	2.98E-92
TMEM249	ENSG0000(	0.45	38.81	-4.779	6.12E-73
TPBGL	ENSG0000(	0.63	5.71	-2.041	6.21E-55
RP11-426L	ENSG0000(	0	5.99	-2.805	6.83E-66
PRSS46	ENSG0000(	0	9.64	-3.411	6.43E-64
RP11-414J	ENSG0000(	0.03	4.19	-2.333	1.52E-82
RP11-580I	ENSG0000(	0.03	4.39	-2.388	2.65E-60
RP11-883C	ENSG0000(	0	10.51	-3.525	1.78E-50
RP11-700H	ENSG0000(	0	3.98	-2.316	3.68E-52
GOLGA6L7I	ENSG0000(	0.05	44.78	-5.446	6.16E-75
RP11-178L	ENSG0000(	0.01	11.13	-3.586	5.27E-73
RP11-327F	ENSG0000(	0	3.21	-2.074	3.73E-42
RP4-612B1	ENSG0000(	0.59	6.1	-2.159	3.59E-85
MIR3976H	ENSG0000(	0	15.55	-4.049	2.55E-63
GOLGA8S	ENSG0000(	0.12	9.44	-3.221	3.14E-77
RP11-152C	ENSG0000(	0	7.38	-3.067	7.61E-60
RP11-1223	ENSG0000(	0.71	130.381	-6.264	4.21E-78
GOLGA8H	ENSG0000(	0.2	7.88	-2.888	1.71E-66
RP11-744I	ENSG0000(	0	4	-2.322	3.33E-46
RP11-244E	ENSG0000(	0.45	6.15	-2.302	1.01E-41
RP11-434E	ENSG0000(	0	12.79	-3.786	1.77E-60
LINC00662	ENSG0000(	3.39	27.92	-2.72	3.91E-61
RP11-146F	ENSG0000(	2.08	49.942	-4.048	1.58E-70
RP11-893F	ENSG0000(	0	4.23	-2.387	6.20E-51
RP11-63A1	ENSG0000(	0	8.89	-3.306	8.86E-56
RP11-23E1	ENSG0000(	0	46.52	-5.57	5.12E-32
RP13-638C	ENSG0000(	0.37	16.67	-3.689	5.83E-49
RP11-266L	ENSG0000(	0.13	6.92	-2.809	5.53E-102
RP11-353N	ENSG0000(	0.01	3.51	-2.159	9.31E-71

RP11-160E	ENSG0000(	0.33	9.35	-2.96	3.94E-44
RP11-960E	ENSG0000(	0	26.02	-4.756	4.94E-59
RP11-515E	ENSG0000(	0	7.01	-3.002	2.54E-58
RP11-388(	ENSG0000(	1.06	8.22	-2.162	8.54E-79
RP11-65J2	ENSG0000(	0.61	58.161	-5.2	3.58E-69
LA16c-349	ENSG0000(	0.19	4.07	-2.091	5.85E-76
RP11-266L	ENSG0000(	0.02	6.98	-2.968	1.41E-68
RP11-196(	ENSG0000(	0.43	8.43	-2.721	5.10E-93
RP11-353N	ENSG0000(	0.04	6.21	-2.793	1.08E-67
RP11-46I8.	ENSG0000(	0.46	6.51	-2.363	1.86E-30
RP11-1055	ENSG0000(	1.31	11.12	-2.391	6.95E-45
RP11-156F	ENSG0000(	4.17	54.628	-3.428	3.52E-74
TMPOP2	ENSG0000(	0.19	7.27	-2.797	1.96E-69
ROCK1P1	ENSG0000(	1.46	49.559	-4.361	2.86E-73
RP11-388(	ENSG0000(	0.11	4.12	-2.206	5.65E-61
RP11-291L	ENSG0000(	4.53	37.65	-2.805	7.08E-43
RP11-166F	ENSG0000(	0.08	9.44	-3.273	1.94E-59
LRRC37A17	ENSG0000(	0.93	9.14	-2.393	5.02E-60
RP11-849I.	ENSG0000(	0.69	10.39	-2.753	5.42E-34
RP11-333E	ENSG0000(	0.47	15.519	-3.49	2.29E-83
RP11-109N	ENSG0000(	0	7.72	-3.124	1.98E-62
THCAT158	ENSG0000(	0.1	11.45	-3.501	4.37E-70
RP1-4G17.	ENSG0000(	0	6.31	-2.87	2.69E-49
RP1-59D14	ENSG0000(	1.07	7.64	-2.061	2.81E-72
RP11-202D	ENSG0000(	0	9.88	-3.444	1.40E-57
RP11-707(	ENSG0000(	0	8.59	-3.262	5.58E-64
UBBP4	ENSG0000(	1.16	114.3	-5.738	3.78E-88
RP11-527H	ENSG0000(	0	3.35	-2.121	1.02E-50
PPP4R1-AS	ENSG0000(	0	7.2	-3.036	9.89E-67
RP11-628(	ENSG0000(	0	15.75	-4.066	1.59E-62
RP11-746E	ENSG0000(	0	12.52	-3.757	1.89E-58
RP11-580I.	ENSG0000(	2.7	17.23	-2.301	3.26E-48
LINC00908	ENSG0000(	0.17	4.84	-2.319	4.44E-65
RP11-573D	ENSG0000(	0.36	13.09	-3.373	2.65E-62
RP11-53I6.	ENSG0000(	0	4.46	-2.449	2.62E-43
SNORD3A	ENSG0000(	76.119	544.843	-2.823	9.73E-10
RP11-434D	ENSG0000(	0.2	7.75	-2.866	9.17E-64
RP11-192H	ENSG0000(	0.08	7.81	-3.028	3.34E-66
RP11-583F	ENSG0000(	0.26	11.1	-3.263	7.17E-77
RP11-739N	ENSG0000(	0.03	11.48	-3.599	4.00E-57
RP1-37N7.	ENSG0000(	0.01	3.79	-2.246	1.59E-57
RP11-757(	ENSG0000(	0.05	5.45	-2.619	4.90E-66
RP13-270F	ENSG0000(	0.35	35.52	-4.758	3.20E-83
RP11-271K	ENSG0000(	0.05	16.02	-4.019	2.37E-60
RP11-14P2	ENSG0000(	0.06	3.95	-2.223	9.59E-11
NOTCH2NI	ENSG0000(	6.28	45.679	-2.681	1.11E-80
MAPT-AS1	ENSG0000(	0	18.76	-4.305	6.90E-65
RP11-381F	ENSG0000(	0.11	3.55	-2.035	2.37E-29
RP11-92B1	ENSG0000(	0	4.88	-2.556	1.34E-55

RP11-26N1	ENSG0000	0	3.43	-2.147	6.65E-45
RP11-343K	ENSG0000	0	3.53	-2.18	8.27E-43
RP11-6N17	ENSG0000	6.9	37.851	-2.298	5.71E-44
RP11-214C	ENSG0000	0.03	3.92	-2.256	2.32E-58
RP11-1109	ENSG0000	0.07	4.09	-2.25	6.42E-52
RP11-387H	ENSG0000	0.11	10.87	-3.419	6.30E-81
RN7SL664F	ENSG0000	0	3.09	-2.032	6.27E-47
RP11-285N	ENSG0000	0	3.44	-2.151	1.80E-45
SNORD3B-	ENSG0000	1.32	9.2	-2.136	1.09E-05
KRT18P55	ENSG0000	0.02	15.15	-3.985	8.42E-79
MIR1539	ENSG0000	0	3.85	-2.278	1.86E-62
RP11-434D	ENSG0000	0	4.38	-2.428	4.41E-53
RP13-516N	ENSG0000	0.11	3.54	-2.032	2.67E-62
RP11-156L	ENSG0000	0.04	5.05	-2.54	6.37E-55
RP11-849F	ENSG0000	0.98	13.25	-2.847	5.24E-42
RP11-17M	ENSG0000	0.59	11.67	-2.994	3.02E-74
RP11-19P2	ENSG0000	0	9.75	-3.426	5.49E-58
RP11-20B2	ENSG0000	0	13.37	-3.845	2.11E-65
LINC00668	ENSG0000	1.21	63.399	-4.865	1.88E-71
GATA6-AS	ENSG0000	0.26	5.6	-2.389	2.95E-22
NDUFV2-A	ENSG0000	1.08	56.18	-4.781	1.38E-85
RHOT1P1	ENSG0000	0.18	10.17	-3.243	9.08E-58
RP11-715C	ENSG0000	0	3.1	-2.036	1.76E-67
RP11-111H	ENSG0000	0	5.05	-2.597	5.75E-51
TBC1D3P5	ENSG0000	0	3.73	-2.242	1.86E-47
RP11-149I	ENSG0000	0	3.73	-2.242	1.30E-55
RP11-401F	ENSG0000	0.26	6.21	-2.517	3.37E-76
GDF10	ENSG0000	0.12	9.04	-3.164	6.47E-38
RP11-94B1	ENSG0000	0	4	-2.322	2.97E-49
RP5-1148A	ENSG0000	0.78	6.17	-2.01	2.80E-33
MYO15B	ENSG0000	20.46	91.982	-2.115	1.62E-35
TBC1D29	ENSG0000	0.12	13.71	-3.715	1.18E-58
RP11-862L	ENSG0000	0.11	16.58	-3.985	6.38E-46
RP5-1022F	ENSG0000	0	9.29	-3.363	7.91E-61
ZNF793-AS	ENSG0000	6.17	62.292	-3.142	2.75E-46
FXYD1	ENSG0000	2.53	14.5	-2.135	5.84E-11
RP11-749H	ENSG0000	0	16.5	-4.129	4.91E-59
LRRC37A16	ENSG0000	0.34	5.12	-2.191	2.41E-56
RP11-92C4	ENSG0000	0.07	21.39	-4.387	2.19E-71
RP11-456C	ENSG0000	0.15	11.41	-3.432	1.59E-49
SLC14A2-A	ENSG0000	0	16.54	-4.133	1.28E-66
RP11-846C	ENSG0000	0	9.03	-3.326	4.47E-56
RP11-449J	ENSG0000	0.25	21.251	-4.154	8.47E-64
RP11-677C	ENSG0000	0	7.92	-3.157	1.25E-22
RP11-411E	ENSG0000	0.23	8.88	-3.006	1.66E-57
RP11-527L	ENSG0000	0	4.53	-2.467	3.14E-53
LCN6	ENSG0000	0.78	9.83	-2.605	1.49E-38
WDR7-OT1	ENSG0000	0	4.03	-2.331	2.64E-47
LINC01255	ENSG0000	0	34.329	-5.143	3.62E-67

MAP3K14-	ENSG0000(	1.76	75.258	-4.788	3.51E-72
RP11-8H2.	ENSG0000(	0.05	3.71	-2.165	2.43E-76
SNHG22	ENSG0000(	1.04	8.05	-2.149	3.14E-76
LINC01478	ENSG0000(	0	3.24	-2.084	4.09E-50
LINC00906	ENSG0000(	0	19.67	-4.369	2.27E-70
RP11-749H	ENSG0000(	0	3.04	-2.014	1.96E-41
RP11-527L	ENSG0000(	0	3.7	-2.233	4.14E-52
RP11-552F	ENSG0000(	0.62	6.89	-2.284	2.54E-82
RP1-37N7.	ENSG0000(	0.17	4.09	-2.121	1.93E-36
ZNF571-AS	ENSG0000(	3.56	35.911	-3.017	8.38E-65
MIR497HG	ENSG0000(	0.86	7.89	-2.257	7.67E-48
RP11-666A	ENSG0000(	0.52	6.12	-2.228	6.86E-95
RP11-383M	ENSG0000(	0	5.39	-2.676	1.48E-74
RP11-128F	ENSG0000(	0	7.14	-3.025	1.83E-55
RP11-126C	ENSG0000(	0	64.44	-6.032	1.19E-67
RAD51L3-F	ENSG0000(	0	5.37	-2.671	9.77E-49
RP11-905K	ENSG0000(	0.05	3.52	-2.106	2.63E-55
RP11-64C1	ENSG0000(	0	4.04	-2.333	8.08E-65
RP11-95O2	ENSG0000(	0.56	10.43	-2.873	3.99E-77
LINC01539	ENSG0000(	0.03	23.501	-4.572	4.49E-63
RP11-64C1	ENSG0000(	0.37	28.509	-4.429	1.67E-72
RP11-718I	ENSG0000(	0	7.37	-3.065	1.39E-50
RP11-379L	ENSG0000(	0	3.07	-2.025	1.22E-43
LINC01533	ENSG0000(	0	12.94	-3.801	6.90E-61
SMIM22	ENSG0000(	1.57	54.628	-4.436	4.26E-46
RP5-1023E	ENSG0000(	0	368.468	-8.529	2.82E-83
RP11-157E	ENSG0000(	0	5.64	-2.731	8.89E-52
RP4-569M	ENSG0000(	0.11	7.43	-2.925	1.32E-54
MAGEA9B	ENSG0000(	0.39	12.46	-3.275	5.37E-32
RP11-805I	ENSG0000(	0	4.96	-2.575	4.82E-60
RP11-388K	ENSG0000(	0	5.82	-2.77	1.24E-52
ZNF649-AS	ENSG0000(	0.05	53.319	-5.693	9.56E-76
RP11-678C	ENSG0000(	0.02	10.64	-3.512	6.23E-61
SSX2B	ENSG0000(	0.22	7.54	-2.807	2.93E-22
RP11-805I	ENSG0000(	0.01	5.91	-2.774	7.39E-44
RP11-464F	ENSG0000(	0.37	7.83	-2.688	1.37E-77
RP11-316C	ENSG0000(	0.33	4.36	-2.011	5.76E-48
MAGEA2	ENSG0000(	0.14	5.14	-2.429	8.22E-28
TEX13A	ENSG0000(	0.07	13.97	-3.806	7.28E-71
MIMT1	ENSG0000(	0.21	7.62	-2.833	3.18E-50
HSFX2	ENSG0000(	0.3	15.04	-3.625	9.43E-59
SCGB1B2P	ENSG0000(	1.08	10.67	-2.488	2.08E-50
RP11-514D	ENSG0000(	0	4.23	-2.387	1.40E-48
RP11-1137	ENSG0000(	0	8.63	-3.268	3.61E-70
ESPNP	ENSG0000(	0.17	4.12	-2.13	2.89E-48
SPANXN2	ENSG0000(	0	3.93	-2.302	1.19E-49
RP1-172N1	ENSG0000(	0	19.28	-4.342	3.89E-68
RP11-256I	ENSG0000(	1.14	13.02	-2.712	4.09E-71
LINC01082	ENSG0000(	0	18.07	-4.253	1.25E-36

ZNF350-A[illegible]	ENSG0000[illegible]	1.51	22.139	-3.205	2.36E-51
GOLGA2P9	ENSG0000[illegible]	0.08	91.493	-6.42	1.70E-81
LINC01233	ENSG0000[illegible]	0.13	20.609	-4.257	3.87E-65
NXF2	ENSG0000[illegible]	0.83	12.34	-2.866	2.74E-32
NXF2B	ENSG0000[illegible]	0.56	14.64	-3.326	2.25E-52
ZIM2	ENSG0000[illegible]	0.54	6.97	-2.372	5.03E-59
RP11-113[illegible]	ENSG0000[illegible]	0.06	39.229	-5.246	3.10E-87
RP11-816[illegible]	ENSG0000[illegible]	0.12	3.6	-2.038	6.13E-64
RP4-740C[illegible]	ENSG0000[illegible]	1.11	8.75	-2.208	5.16E-42
RP11-932[illegible]	ENSG0000[illegible]	2.17	25.849	-3.082	2.22E-61
RP5-1069[illegible]	ENSG0000[illegible]	0	12.85	-3.792	2.78E-67
RP11-394[illegible]	ENSG0000[illegible]	1.18	19.211	-3.213	2.88E-38
RP11-245[illegible]	ENSG0000[illegible]	0	4.93	-2.568	3.19E-60
RNU11	ENSG0000[illegible]	0	5.84	-2.774	1.02E-05
RP11-568[illegible]	ENSG0000[illegible]	0	10.5	-3.524	1.16E-60
RP11-793[illegible]	ENSG0000[illegible]	0.8	6.99	-2.15	2.29E-56
RP11-572[illegible]	ENSG0000[illegible]	1.65	9.73	-2.018	4.41E-72
RP11-123[illegible]	ENSG0000[illegible]	0	12.61	-3.767	1.36E-71
HEATR9	ENSG0000[illegible]	0.07	15.5	-3.947	2.01E-60
RP11-359[illegible]	ENSG0000[illegible]	0.07	3.48	-2.066	4.62E-58
LOC40191[illegible]	ENSG0000[illegible]	0.06	4.51	-2.378	1.91E-53
RP11-373[illegible]	ENSG0000[illegible]	0	3.23	-2.081	2.07E-27
U1	ENSG0000[illegible]	0	4.34	-2.417	8.61E-16
RP11-103[illegible]	ENSG0000[illegible]	0	5.9	-2.787	1.11E-56
LINC00221	ENSG0000[illegible]	1.94	11.73	-2.114	7.17E-17
RP11-355[illegible]	ENSG0000[illegible]	3.59	17.78	-2.033	3.29E-54
RP11-607[illegible]	ENSG0000[illegible]	0.13	22.5	-4.378	1.12E-74
NAMA	ENSG0000[illegible]	0.01	4.96	-2.561	6.48E-71
RP11-416[illegible]	ENSG0000[illegible]	0	6.11	-2.83	5.50E-55
RP11-75N[illegible]	ENSG0000[illegible]	0	5.92	-2.791	6.03E-57
RP4-799G[illegible]	ENSG0000[illegible]	0	6.14	-2.836	4.09E-62
MMP28	ENSG0000[illegible]	0.93	51.388	-4.763	1.33E-89
RP11-89C[illegible]	ENSG0000[illegible]	0.65	8.58	-2.538	5.91E-60
RP11-335[illegible]	ENSG0000[illegible]	0	8.13	-3.191	5.33E-76
LINC00904	ENSG0000[illegible]	0	3.37	-2.128	1.22E-58
RP11-548[illegible]	ENSG0000[illegible]	0.81	16.84	-3.301	1.28E-25
GS1-393G[illegible]	ENSG0000[illegible]	0.32	6.99	-2.598	2.72E-78
MROH7-T[illegible]	ENSG0000[illegible]	0.08	11.99	-3.588	2.72E-85
RP1-202O[illegible]	ENSG0000[illegible]	0	44.929	-5.521	2.51E-71
RP11-52L5	ENSG0000[illegible]	0	7.26	-3.046	1.65E-66
RP11-426[illegible]	ENSG0000[illegible]	0.23	17.689	-3.925	1.01E-72
RP11-493[illegible]	ENSG0000[illegible]	0.26	119.817	-6.583	3.61E-72
RP4-635E1	ENSG0000[illegible]	6.52	31.899	-2.129	8.61E-62
RP11-354[illegible]	ENSG0000[illegible]	0	3.57	-2.192	7.00E-66
RP11-572[illegible]	ENSG0000[illegible]	0.24	5.57	-2.406	6.72E-77
RP4-736L2	ENSG0000[illegible]	0.12	7.96	-3	2.01E-63
RP11-79O[illegible]	ENSG0000[illegible]	0.16	4.63	-2.279	1.66E-67
RP1-313I6[illegible]	ENSG0000[illegible]	1.39	13.79	-2.63	1.00E-57
RP11-421[illegible]	ENSG0000[illegible]	0.24	7.2	-2.725	1.56E-63

RP11-107(	ENSG0000(	0	4.4	-2.433	2.70E-50
RP11-428J	ENSG0000(	1.34	13.61	-2.642	6.24E-42
FGF14-AS2	ENSG0000(	1.77	11.03	-2.119	6.87E-81
NBPF25P	ENSG0000(	0.84	7.78	-2.255	2.35E-58
RP11-156E	ENSG0000(	0.15	6.43	-2.692	1.00E-94
HIST2H2A/	ENSG0000(	3.77	85.591	-4.182	4.11E-58
XXbac-BPG	ENSG0000(	0.49	7.26	-2.471	6.42E-84
RP5-855D2	ENSG0000(	1.39	9.7	-2.163	1.01E-49
XXbac-BPG	ENSG0000(	0.81	12.26	-2.873	1.11E-47
TPTE2P2	ENSG0000(	1.67	9.93	-2.033	1.07E-30
RP11-140K	ENSG0000(	4.37	98.92	-4.218	3.77E-83
RP11-65L1	ENSG0000(	0	6.66	-2.937	2.53E-65
RP13-539J	ENSG0000(	0.06	7.18	-2.948	4.14E-59
RP11-359I	ENSG0000(	0.25	15.47	-3.72	2.24E-71
RP11-25O1	ENSG0000(	0	3.81	-2.266	1.17E-59
RP11-363E	ENSG0000(	0.09	4.14	-2.237	2.04E-52
RP1-225E1	ENSG0000(	0	6.37	-2.882	2.59E-55
RP11-532N	ENSG0000(	0.2	6.19	-2.583	2.59E-56
RP11-713N	ENSG0000(	0.33	18.24	-3.855	8.93E-49
RP11-105N	ENSG0000(	0	7.49	-3.086	1.98E-32
XXbac-BPG	ENSG0000(	0.22	10.67	-3.258	1.89E-80
RP11-638I	ENSG0000(	18.45	105.601	-2.454	9.31E-36
ZSWIM8-A	ENSG0000(	1.59	19.46	-2.982	6.15E-76
RP11-504F	ENSG0000(	10.52	57.221	-2.337	8.07E-55
RP11-191L	ENSG0000(	0.47	6.04	-2.26	2.32E-73
RP11-190A	ENSG0000(	0.39	6.08	-2.349	1.69E-35
RP11-803E	ENSG0000(	0.97	12.59	-2.786	2.51E-50
RP1-63G5.	ENSG0000(	0	22.461	-4.552	9.48E-68
MESTIT1	ENSG0000(	0.07	3.56	-2.091	1.08E-53
RP11-44N2	ENSG0000(	0.26	11.08	-3.261	6.98E-72
STAG3L5P-	ENSG0000(	29.49	198.858	-2.713	4.28E-83
JMJD1C-A	ENSG0000(	0.36	9.85	-2.996	1.88E-57
RP11-335L	ENSG0000(	0.1	3.67	-2.086	1.87E-66
RP11-464F	ENSG0000(	0.48	4.98	-2.015	1.87E-77
RP11-602N	ENSG0000(	0.12	5.35	-2.503	4.21E-80
RP11-219A	ENSG0000(	0	4.21	-2.381	2.30E-52
RP11-295N	ENSG0000(	0.1	3.74	-2.107	1.87E-54
RP11-398(	ENSG0000(	0.91	7.18	-2.099	1.54E-69
LL22NC03-	ENSG0000(	1.28	13.58	-2.677	6.00E-40
RP5-1159(	ENSG0000(	0.84	12.93	-2.92	4.22E-82
RP11-74E2	ENSG0000(	0.18	4.63	-2.254	9.08E-50
RP11-392E	ENSG0000(	0.1	12.4	-3.607	5.86E-82
RP11-118K	ENSG0000(	0.69	6.44	-2.138	3.25E-32
FAM95C	ENSG0000(	0.9	89.958	-5.581	1.66E-100
RP11-428K	ENSG0000(	0.1	3.72	-2.101	2.39E-62
RP11-216L	ENSG0000(	0.84	19.569	-3.483	9.74E-88
RP11-559N	ENSG0000(	0.04	3.79	-2.203	1.26E-62
LYPD4	ENSG0000(	0.02	137.129	-7.081	2.52E-94
RP11-458F	ENSG0000(	10.58	63.079	-2.468	8.24E-36

RP11-449F	ENSG0000(	0.68	6.39	-2.137	7.07E-71
RP11-392E	ENSG0000(	0.15	23.501	-4.413	9.68E-101
RP11-11N1	ENSG0000(	0.07	7.59	-3.005	3.07E-61
FAM25BP	ENSG0000(	0	9.7	-3.42	9.29E-56
RP3-402G1	ENSG0000(	0.51	8.52	-2.656	2.87E-82
RP11-136C	ENSG0000(	0.13	7.57	-2.923	7.88E-131
RP11-496H	ENSG0000(	1	235.714	-6.887	3.96E-72
RBAKDN	ENSG0000(	1.3	672.457	-8.194	6.49E-73
RP11-672L	ENSG0000(	0.95	9.73	-2.46	1.03E-72
RP11-634H	ENSG0000(	1.95	18.621	-2.734	5.82E-60
RP11-255E	ENSG0000(	0.3	6.18	-2.465	2.88E-67
RP11-87C7	ENSG0000(	0	9.08	-3.333	2.44E-58
ST7-OT4_2	ENSG0000(	0	3.28	-2.098	3.50E-14
ST7-OT4_1	ENSG0000(	0	4.79	-2.534	1.15E-28
RP5-908M	ENSG0000(	3.68	36.39	-2.998	2.10E-67
RP11-758H	ENSG0000(	2.01	17.22	-2.598	9.70E-75
RP11-613E	ENSG0000(	0.09	14.44	-3.824	7.94E-60
U1	ENSG0000(	4.86	44.301	-2.951	1.70E-36
RP11-227D	ENSG0000(	0	3.43	-2.147	3.38E-55
GOLGA6L1	ENSG0000(	0.12	34.64	-4.992	6.75E-54
FAM27E3	ENSG0000(	0.8	22.161	-3.686	1.67E-69
uc_338	ENSG0000(	0	3.69	-2.23	4.83E-26
OR7E156P	ENSG0000(	1.41	15.6	-2.784	1.04E-14
H2AFB1	ENSG0000(	0.1	10.71	-3.412	9.44E-17
SOCS7	ENSG0000(	8.78	99.373	-3.359	2.13E-52
RP11-757C	ENSG0000(	0.1	17.31	-4.057	3.77E-72
TBC1D3H	ENSG0000(	0	10	-3.459	4.95E-38
RP11-566K	ENSG0000(	1.13	20.76	-3.353	8.32E-60
RP11-44M	ENSG0000(	3.02	28.709	-2.886	8.95E-95
GAGE13	ENSG0000(	0.28	25.259	-4.359	1.26E-42
RP11-347I	ENSG0000(	0.65	6.46	-2.177	4.92E-83
RP11-486C	ENSG0000(	0.08	20.281	-4.3	2.53E-64
RP5-994D1	ENSG0000(	0.11	4.46	-2.298	6.10E-46
TPTE	ENSG0000(	3.96	81.769	-4.061	7.43E-57
LLNLR-304	ENSG0000(	0.08	5.3	-2.544	2.94E-51
RP11-403E	ENSG0000(	0.05	24.501	-4.602	2.09E-74
RP11-285E	ENSG0000(	0	5.67	-2.738	1.77E-63
SPDYE10P	ENSG0000(	0.43	14.32	-3.421	1.43E-73
RIMBP3B	ENSG0000(	0.04	15.49	-3.987	1.24E-64
FAM95B1	ENSG0000(	3.32	90.039	-4.397	2.36E-38
RP11-2C24	ENSG0000(	0.09	13.99	-3.782	3.06E-71
TBC1D3B	ENSG0000(	2.01	19.79	-2.788	6.90E-50
RP1-292L2	ENSG0000(	1.39	14.74	-2.719	8.89E-94
LINC01297	ENSG0000(	0.08	6.38	-2.773	3.98E-71
RP11-12A2	ENSG0000(	0.25	7.19	-2.712	3.88E-69
TBC1D3I	ENSG0000(	0.06	8.21	-3.119	1.32E-47
RP5-864K1	ENSG0000(	0.46	8.48	-2.699	2.55E-34
RP11-382E	ENSG0000(	0	6.28	-2.864	1.56E-64
ZNF280B	ENSG0000(	2.48	16.19	-2.304	1.12E-59

RP1-101D8	ENSG00000	0.01	4.66	-2.486	4.77E-62
RP11-187C	ENSG00000	0.3	5.46	-2.313	1.44E-53
RP4-737E2	ENSG00000	0	5.88	-2.782	2.28E-51
RP3-446N1	ENSG00000	0	6.69	-2.943	3.28E-55
RP11-157L	ENSG00000	0	23.569	-4.619	4.03E-64
RP11-15J2	ENSG00000	0	4.92	-2.566	1.67E-58
RP5-908M	ENSG00000	0.36	4.81	-2.095	1.21E-81
RP11-666C	ENSG00000	1.01	8.84	-2.291	3.48E-62
RP11-266L	ENSG00000	1.93	81.019	-4.807	1.94E-78
LINC00684	ENSG00000	0.09	322.085	-8.211	1.69E-78
KB-1572G7	ENSG00000	0	4.64	-2.496	1.65E-45
RP4-547N1	ENSG00000	0.01	4.54	-2.456	3.38E-89
LYZL6	ENSG00000	0	62.708	-5.993	1.26E-72
RP11-100C	ENSG00000	0.03	7.74	-3.085	6.52E-65
RP11-54C4	ENSG00000	0.71	14.87	-3.214	2.25E-93
RIMBP3	ENSG00000	0.15	33.381	-4.902	1.12E-68
RP11-457N	ENSG00000	0.02	19.04	-4.296	1.74E-69
RP11-291L	ENSG00000	2.95	27.53	-2.853	4.01E-46
LINC00162	ENSG00000	0	7.19	-3.034	3.48E-31
RP11-613E	ENSG00000	1.01	29.28	-3.913	1.10E-56
PRSS2	ENSG00000	0.08	7.22	-2.928	3.18E-13
TBC1D3F	ENSG00000	0	7.31	-3.055	1.20E-37
RP11-61K9	ENSG00000	2.05	18.72	-2.693	2.62E-75
SPATA31A3	ENSG00000	0.07	5.57	-2.618	3.63E-51
SPDYE11	ENSG00000	0.17	4.01	-2.098	1.06E-67
SPATA31A7	ENSG00000	0.09	5.04	-2.47	8.47E-42
RP5-1017F	ENSG00000	0	17.4	-4.202	7.55E-71
FAM27E2	ENSG00000	0	3.44	-2.151	2.91E-44
TTC28-AS1	ENSG00000	0	3.41	-2.141	5.94E-14
LINC00226	ENSG00000	0.07	18.85	-4.213	4.42E-60
FLJ36000	ENSG00000	0.34	9.18	-2.925	6.57E-54
RP11-195L	ENSG00000	0.09	13.78	-3.761	8.35E-66
NPPA-AS1	ENSG00000	0.92	10.97	-2.64	9.54E-22
RP11-417C	ENSG00000	0	10	-3.459	3.51E-57
RP11-291L	ENSG00000	7	55.001	-2.807	6.26E-48
RP11-8P11	ENSG00000	0.01	3.11	-2.025	2.99E-68
RP11-478K	ENSG00000	0	9.45	-3.385	2.00E-61
RP11-467L	ENSG00000	0.71	9.04	-2.554	1.07E-67
GSTTP1	ENSG00000	0	36.481	-5.228	1.62E-58
RP11-476H	ENSG00000	0	9.94	-3.452	4.71E-77
RP11-108H	ENSG00000	0	3.12	-2.043	6.24E-48
STAG3L2	ENSG00000	5.33	34.881	-2.503	2.57E-62
RP11-520E	ENSG00000	0.26	7.55	-2.763	4.51E-57
RP4-550H1	ENSG00000	0.13	6.15	-2.662	6.45E-66
GOLGA6L6	ENSG00000	0.01	7.02	-2.989	7.46E-57
RP11-325L	ENSG00000	0.4	5.26	-2.161	3.06E-65
RP11-380N	ENSG00000	0.29	4.28	-2.033	5.22E-61
RP11-486I	ENSG00000	0.71	38.851	-4.543	2.20E-82
RP13-347D	ENSG00000	0	3.21	-2.074	2.39E-65

GOLGA8IP	ENSG0000(	0.07	8.79	-3.194	3.19E-67
RP11-961A	ENSG0000(	0.45	7.57	-2.563	2.69E-65
RP11-374N	ENSG0000(	0	4.28	-2.4	1.08E-30
XXbac-BPG	ENSG0000(	0	6.21	-2.85	1.33E-69
RP11-734K	ENSG0000(	2.45	102.721	-4.91	4.53E-63
RP11-693J	ENSG0000(	0.63	8.28	-2.509	5.41E-51
GOLGA6L1	ENSG0000(	0.02	7.67	-3.087	6.09E-76
H2AFB3	ENSG0000(	0.07	38.719	-5.214	4.26E-59
LLNLF-136	ENSG0000(	0	3.13	-2.046	5.07E-59
RP11-269C	ENSG0000(	0.45	7.74	-2.592	3.73E-78
RP11-714N	ENSG0000(	0	3.2	-2.07	2.04E-76
GOLGA6L2	ENSG0000(	0.01	12.33	-3.722	1.25E-63
FAM230A	ENSG0000(	0	11.42	-3.635	9.45E-56
RP11-403F	ENSG0000(	2.15	14.9	-2.336	9.63E-64
RP11-356N	ENSG0000(	0	3.38	-2.131	8.47E-25
RP11-426C	ENSG0000(	0.26	5.11	-2.278	9.86E-63
RDM1	ENSG0000(	2.28	16.959	-2.453	3.48E-49
RP11-34H1	ENSG0000(	0	7.41	-3.072	2.17E-36
TEX28	ENSG0000(	0	11.3	-3.621	2.93E-66
RP11-736N	ENSG0000(	0.08	4.28	-2.289	8.62E-65
RP11-87C1	ENSG0000(	0	4.26	-2.395	2.96E-17
RP11-440J	ENSG0000(	0	8.55	-3.256	3.14E-65
RP11-12A2	ENSG0000(	0.25	10.09	-3.149	1.24E-86
PIK3R3	ENSG0000(	0	22.25	-4.539	4.83E-54
TSC22D1-A	ENSG0000(	0.25	5.6	-2.401	3.61E-113
SSTR3	ENSG0000(	0.24	9.65	-3.102	1.15E-72
RP11-245D	ENSG0000(	2.92	33.611	-3.142	1.92E-51
TBC1D3C	ENSG0000(	0	6.66	-2.937	8.44E-22
OR7E104P	ENSG0000(	0.1	5.47	-2.556	5.95E-21
RP11-89H1	ENSG0000(	0.07	3.78	-2.159	1.43E-66
RP11-96A1	ENSG0000(	0	8.7	-3.278	1.10E-67
RP11-457D	ENSG0000(	0	8.17	-3.197	1.93E-63
POTEB3	ENSG0000(	0	5.26	-2.646	3.14E-64
TMEM191E	ENSG0000(	2.89	172.278	-5.477	4.00E-57
GOLGA6L1(	ENSG0000(	0.88	24.19	-3.744	3.08E-65
IQCA1L	ENSG0000(	0.03	14.69	-3.929	2.04E-61
MCM8-AS	ENSG0000(	0	5.99	-2.805	2.70E-61
RP11-12A2	ENSG0000(	0	30.279	-4.967	1.26E-63
FAM27B	ENSG0000(	0.45	20.72	-3.905	2.30E-49
RP11-23E1	ENSG0000(	0.47	5.8	-2.21	2.01E-60
RP11-327I	ENSG0000(	0	11.66	-3.662	8.06E-90
NPPA-AS1	ENSG0000(	0.07	11.26	-3.518	5.24E-14
RP11-612J	ENSG0000(	0	4.92	-2.566	5.33E-44
RP11-511H	ENSG0000(	0.25	4.24	-2.068	3.57E-91
RP13-580E	ENSG0000(	0.59	23.039	-3.918	1.29E-43
RP11-667F	ENSG0000(	0.07	9.36	-3.275	5.47E-59
RP11-76C1	ENSG0000(	0	6.18	-2.844	7.49E-57
RP1-149M	ENSG0000(	0	16.6	-4.138	6.08E-63
RP11-203H	ENSG0000(	0.11	5.91	-2.638	2.05E-57

RP11-36D1	ENSG00000	0	7.48	-3.084	1.29E-51
RP11-218N	ENSG00000	0	4.63	-2.493	1.50E-61
RP11-810F	ENSG00000	0.15	3.96	-2.109	2.10E-81
RP11-461C	ENSG00000	0	15.519	-4.046	2.72E-63
RP11-83B2	ENSG00000	0	3.89	-2.29	5.45E-48
RP11-417N	ENSG00000	4.75	22.55	-2.034	6.70E-35
RP11-661A	ENSG00000	0.82	6.51	-2.045	7.22E-72
RP11-574K	ENSG00000	0.96	6.96	-2.022	2.44E-49
RP11-495K	ENSG00000	0.03	4.53	-2.425	4.35E-56
RP11-752C	ENSG00000	0.17	3.74	-2.018	5.48E-85
LINC00552	ENSG00000	0.05	17.72	-4.156	8.99E-86
RP11-499E	ENSG00000	0.05	3.92	-2.228	5.52E-67
RP11-886F	ENSG00000	0.06	34.099	-5.049	3.45E-72
RP11-570J	ENSG00000	0	4.93	-2.568	4.61E-39
RP11-69M	ENSG00000	0.61	5.79	-2.076	8.33E-72
RP11-447N	ENSG00000	0	3.89	-2.29	6.56E-58
RP11-1221	ENSG00000	0	12.45	-3.75	1.30E-57
RP11-76C1	ENSG00000	0.09	3.69	-2.105	1.84E-46
U51561.1	ENSG00000	0	3.66	-2.22	5.52E-53
MGC39584	ENSG00000	0.07	24.411	-4.57	5.04E-68
RP3-523C2	ENSG00000	0	5.82	-2.77	8.74E-60
RP11-384N	ENSG00000	0.3	5.45	-2.311	2.47E-86
LA16c-380	ENSG00000	1.01	11.75	-2.665	2.31E-69
MIR4697H	ENSG00000	1.58	12.04	-2.338	2.81E-63
RP11-542F	ENSG00000	0	4.74	-2.521	1.04E-58
RP11-1228	ENSG00000	5.75	29.999	-2.199	2.73E-27
RP11-5O17	ENSG00000	0.53	9.08	-2.72	4.13E-56
RP11-837J	ENSG00000	0.08	4.63	-2.382	4.53E-59
LINC01374	ENSG00000	0	4.48	-2.454	2.22E-60
SCAANT1	ENSG00000	0.46	5.32	-2.114	1.53E-47
RP11-983C	ENSG00000	0	8.96	-3.316	4.74E-60
LINC00251	ENSG00000	0.02	3.2	-2.042	1.46E-42
LINC01232	ENSG00000	0.82	9.41	-2.516	3.90E-98
RP4-777O2	ENSG00000	0.08	3.47	-2.049	5.61E-57
LINC00254	ENSG00000	0	3.93	-2.302	3.54E-50
RP5-991B1	ENSG00000	0	13.7	-3.878	7.37E-62
RP5-991B1	ENSG00000	0	56.04	-5.834	1.18E-72
SEPSECS-A	ENSG00000	4.22	23.98	-2.259	1.98E-28
RP4-669P1	ENSG00000	0.7	15.95	-3.318	5.87E-67
LINC00895	ENSG00000	0	3.5	-2.17	7.99E-73
GS1-114I9	ENSG00000	0.83	6.72	-2.077	2.04E-29
RP11-384K	ENSG00000	0.06	8.62	-3.182	1.46E-80
FBF1	ENSG00000	0.46	5.78	-2.215	5.11E-83
TMBIM4	ENSG00000	1.04	24.289	-3.632	4.24E-41
RP11-506F	ENSG00000	0.39	8.39	-2.756	8.29E-85
RP11-527L	ENSG00000	0.24	6.07	-2.511	1.79E-50
RP11-173A	ENSG00000	0.06	8.07	-3.097	1.06E-65
LINC01002	ENSG00000	60.241	406.919	-2.736	2.93E-27
RP11-389C	ENSG00000	0	35.299	-5.182	3.66E-72

C11orf71	ENSG0000(	2.18	222.675	-6.136	1.07E-76

Table S3

GOID	Pathway	Count	PValue
1	intracell	500	########
2	cell part	527	########
3	cell	527	########
4	intracell	491	########
5	organelle	458	########
6	binding	472	2.67E-96
7	cellular	475	4.58E-90
8	intracell	425	1.07E-88
9	single-or	435	3.99E-83
10	cytoplasm	394	3.04E-82
11	single-or	400	8.41E-74
12	single or	135	3.55E-68
13	membrane-	394	1.62E-66
14	reproduct	138	1.02E-65
15	reproduct	138	1.13E-65
16	sexual re	109	5.66E-64
17	multi-org	114	1.14E-60
18	protein b	362	3.63E-60
19	intracell	362	1.29E-59
20	spermatog	88	1.44E-58
21	male game	88	1.60E-58
22	gamete ge	94	2.37E-57
23	multicell	103	6.57E-57
24	multicell	101	1.70E-55
25	developme	249	1.34E-53
26	anatomica	240	1.35E-53
27	single-or	243	2.73E-51
28	cellular	198	1.34E-50
29	multi-org	153	4.92E-49
30	multicell	264	3.18E-48
31	biologica	345	4.53E-46
32	metabolic	333	2.74E-44
33	organic s	326	3.69E-44
34	cellular	233	2.30E-42
35	cell diff	176	2.83E-42
36	cellular	234	3.83E-41
37	organelle	278	1.65E-40
38	cytoplasm	272	3.56E-40
39	regulatic	322	3.96E-40
40	primary m	305	4.53E-38
41	regulatic	308	5.67E-38
42	nucleus	243	4.67E-37
43	intracell	265	5.66E-36
44	single-mu	219	1.22E-35

45	cilium	64	1.26E-35
46	multicell	194	4.62E-35
47	cellular	298	1.07E-34
48	non-membr	169	5.25E-34
49	intracell	169	5.25E-34
50	cell proj	111	4.61E-33

Table S4

GOID	Pathway	Count	PValue
1	Metabolis	69	1.61E-08
2	Assembly	18	2.05E-07
3	Metabolis	32	3.59E-06
4	Organelle	19	5.93E-05
5	Intraflag	8	8.53E-05
6	Fertiliza	6	0.000119
7	Reproduct	6	0.000119
8	Signaling	12	0.000122
9	Anchoring	9	0.00051
10	Peptide h	8	0.000918
11	@@Disease	22	0.102254
12	@@Pyruvat	3	0.051806
13	Synthesis	4	0.001437
14	Signal Tr	62	0.001521
15	Meiosis	9	0.001765
16	PIWI-inte	5	0.001959
17	MHC class	9	0.001964
18	Meiotic s	7	0.003386
19	Glutathio	5	0.004194
20	Biologica	11	0.004215

Table S5

GOID	Pathway	Count	PValue
1	CCKR sigr	10	0.005916
2	Huntingtc	8	0.010263
3	p53 pathw	4	0.042298

Table S6

GOID	Pathway	Count	PValue
1	Adherens	9	9.29E-05
2	Metabolic	41	0.000138
3	Pyruvate	6	0.000948
4	Carbon me	9	0.001436
5	Glycolysi	7	0.001736
6	Glutathio	6	0.002923
7	Glucagon	8	0.003265
8	Longevity	6	0.006937
9	Leukocyte	8	0.007047
10	Synthesis	3	0.008589
11	Endocytos	12	0.009503
12	Ovarian s	5	0.012946
13	Epstein-B	10	0.014369
14	Bacterial	6	0.014947
15	Influenza	9	0.01733
16	Salivary	6	0.024212
17	Huntingto	9	0.027937
18	Fc gamma	6	0.028852
19	Longevity	6	0.030039
20	Protein p	8	0.035188

Table S7

OS gene	All DEGs	Venn gene
DPCD	PTPN6	GSTA1
AC018816.3	GSTO1	LAPTM5
RP11-757O6.1	TNFRSF1B	PRM1
RP11-30P6.6	LYZ	SYNGR4
PTCHD1	GIMAP4	FNDC8
FADS2	DSP	VAMP2
RP1-142L7.9	CXCR4	FSCN3
APOC3	EFEMP1	MAMLD1
SPDEF	COL4A1	GLB1L
RP11-857B24.1	CRIP1	
IL5	LAPTM5	
CUTC	MMP9	
AWAT2	BASP1	
GRIK5	NFE2L3	
SAMD14	CORO1A	
INSRR	CCL19	
CAV3	HLA-DRB1	
APOA4	TYROBP	
RP5-963E22.4	C1QA	
LINC01484	ISG15	
RPL23AP25	CD9	
RP11-242C19.2	KRT8	
ECHS1	CDH1	
IGHV3-66	C1QB	
SLIT1	CD74	
CFC1	TGFBI	
MBD3L1	ARHGDIB	
UPF3AP1	LUM	
TEX35	HLA-DRA	
SSSCA1	FZD5	
AMHR2	CD24	
RP11-434E6.4	CCL2	
C5orf66-AS1	GBP1	
RP11-210M15.2	RAB25	
TTC41P	MMP12	
PDE9A	ADAMDEC1	
OMA1	SPP1	
NGFRAP1	LIN28A	
ISYNA1	KRT19	
CATSPER4	SPACA1	
ZNF705G	LINC00588	
METTL12	RNFT1	
RP11-619A14.3	RIBC2	
RP11-799D4.1	KPNA5	

CISD3	ZNF280B
DNPH1	DNAI2
IHH	NACA2
RP11-555K12.1	MORC1
RP11-177F15.1	GPR137
RP11-334L9.1	ADAM18
SNAP47-AS1	ADGB
RP3-337H4.6	ADCY10
RP11-89H19.1	CCDC70
RP6-65G23.5	SPO11
BEX1	RNF32
CTC-820M8.1	C11orf63
C1QTNF9	ADAM2
AC007326.10	PAPOLB
CTB-1144G6.6	LINC00837
LRRC75A-AS1	BTG4
CCDC162P	DPY19L1P1
MS4A12	LDHAL6B
CRX	SCIN
RP11-588H23.3	GLB1L
HSD17B2	CEP112
RP11-368M16.3	ATXN3L
IGKV1-17	RPGRIP1
FENDRR	SYNGR4
ZNF114	SPATA1
DNAAF2	SOX30
CYP2A13	TEX12
ADAMTS7P4	CCDC88A
TMEM257	CYLC1
RNF208	UBQLN3
RP5-1057I20.5	GRK4
SFRP5	CCDC186
RP11-88H12.2	CYLC2
IQCC	ERO1LB
RP11-51F16.1	GAPDHS
FAM90A20P	AURKC
DMRTC1	GPC5
RP11-1057B6.1	CARF
AC006273.5	LINC00342
ERVWE2	SPATA31C2
C11orf86	C20orf195
DDRGK1	C6orf10
SIGLEC12	IQCK
RP11-752G15.10	CAMK4
CTD-2616J11.11	ART3
BAAT	RFPL3S

MDP1	AKAP4
ARPC3P1	CCHCR1
RP11-193H5.2	IRGC
AC007879.2	TSSK2
LCN15	GK2
SLC25A22	NME8
RP11-757G1.6	CBLN1
RP11-138I1.4	CCIN
KCNJ13	USP2
ADCK5	PPP1R2P9
RP11-445F12.1	ADD3-AS1
FAM229B	PRKAA1
MGAT4A	ALLC
AVPR1B	C16orf95
ZNF780A	TEX28
CDK5R2	MYT1L
GPX8	COIL
TMLHE-AS1	CT62
SFTPD-AS1	RFX2
CDH7	MROH7
RP11-299H22.3	PRPS1L1
TRPC4	TNP2
RP11-264I13.2	C9orf9
RP11-543B16.2	PSORS1C1
CENPB	GPR135
RP11-295D4.4	HSPA1L
SMAD6	TTLL7
BIRC5	PMFBP1
AP000783.1	ZBTB32
DMP1	LRRC36
AMIGO3	FBXO24
CGREF1	POM121L9P
CH507-216K13.1	AQP5
RP1-241P17.4	ZC2HC1C
AC005618.9	KDM4D
SH3PXD2A-AS1	ZNRF4
MTHFS	ACRV1
RP11-367N14.2	ZSCAN5A
BMPER	CTAGE1
RP13-270P17.3	NOL4
PHPT1	LRRC48
PNMA6A	TSGA10
RP11-274B21.3	RDH8
RP11-383B4.4	CATSPERG
AC023347.1	APH1B
MLIP	GRAMD1C

RP11-725P16.2	SLCO3A1
PTRF	SNX19
SMTNL2	MNS1
NPM3	SYCP2
ZNF7	VPRBP
RP11-55K22.2	LRIT1
RP11-458F8.4	HIRIP3
AQP10	PACRG
PNOC	ZCCHC14
PORCN	IZUMO4
FCRL5	TEX15
RP5-1028K7.3	TULP2
TIMM8AP1	C16orf71
DLX3	EFCAB6
ATP2C2	HCFC2
HNF1A	PGK2
KIF20B	ADAM5
SLN	WDR78
RPS20P4	PRKACG
IGKV1OR2-108	TDRD1
RP11-295M18.6	PDHA2
FKSG54	CLPB
RN7SL208P	ANKMY1
DNAJC17	DDX25
F2RL3	ADAM30
C1orf105	ACTL7B
AP000349.2	TAF7L
RP4-570O12.3	SSX3
CELF6	CETN1
NPLOC4	RAB27B
IGSF8	ZCWPW1
C1orf116	LRP2BP
RP3-467L1.6	SLC25A31
SCARNA6	LECT2
RP11-864I4.4	ARHGAP35
CTB-151G24.2	TCP11
BX255923.2	AHI1
C20orf144	ACR
HPCAL4	OXCT2
RPS6KB2	CAPN11
GCSAML-AS1	TRAPPC12
RP1-80N2.2	CST8
ABC14-1080714F1·	CCDC7
GOLGA2P9	MCF2
XXbac-BPG308K3.	CLIP4
AC012066.1	DOC2A

SND1	HHIPL2
SDHAF1	ST7L
ASGR1	CCDC15
IGLV4-60	GDF9
ASS1P2	SLC1A6
RP11-482G13.1	NAT6
RP11-552F3.4	PIWIL1
RP11-481J2.2	CDADC1
PSMB5	LYZL6
IRX3	SSX1
FRY-AS1	SOCS7
CTC-558O2.1	SPATA6
RP11-324H9.1	HSPA4L
RP11-496H1.2	HOOK1
ST6GALNAC1	ZNF821
AC010900.2	SPATA7
ARL3	FNDC8
ADAD1	DKKL1
RPS9	ACE
RP11-776H12.1	MS4A5
ZC3HAV1L	TSKS
RN7SL263P	SYCP1
CRHR1-IT1	YPEL1
CD151	MMP28
RP11-442G21.2	ROPN1B
LINC00475	CHD5
ATP6V1B1-AS1	TEX40
RPL39L	LDHC
AKAP4	TTC12
TERF1P2	DNAH17
RP11-341D18.2	CTNNA3
RP11-338N10.3	ZBBX
MRPS14	TRIM66
H2AFX	SHCBP1L
CXorf49	CRISP2
SMN2	FSCN3
CCDC86	UTRN
RP11-102K13.5	PIGV
KCNJ4	C1orf105
ATRIP	PRSS21
EP300-AS1	SPTBN2
GINM1	SLC25A28
TRIP6	ACSBG2
RP5-1096J16.1	ZMYND10
CRACR2B	ACTL7A
IGLV7-46	IL13

CNIH1	IFT88
FLJ33360	RAB11FIP5
PCDHB5	TCP10
PXT1	HSF2BP
ATP10A	AGBL2
SNHG8	ZNF473
UTS2B	MAPRE3
RP11-172E9.2	DYRK3
EEF1E1P1	MAK
SLC25A47	FER
DIO2	CREM
GLTSCR2-AS1	ELL3
RP11-420N3.3	SFMBT1
AKR1D1	ZPBP
PITX2	USP32
SLC2A7	SMG7-AS1
THTPA	BBIP1
RP11-71G12.1	RBMXL2
CHD4	ODF1
CENPCP1	SOX5
SLC25A23	CHGA
BCL2L14	HYPM
HMSD	DRICH1
C14orf80	ANKS1B
ATP11B	ABCB9
MT-ND5	MAPKBP1
ENTHD1	CHKA
MISP	CCT6B
RP11-193H5.1	TEX30
FAM149A	PCSK2
RP11-439M11.1	ALDH1A2
AFP	ANKRD7
ANKRD20A5P	CLTCL1
TUBB8P1	SMPD2
HRAS	CCNO
ZNF579	INTS6
CYP2C18	SPANXB1
BTBD16	TEX14
CEP250	MAGEC1
OAT	BAP1
AC093642.3	FGFR1OP
TCEB2	CDKL3
DCTN3	SAMD4A
RP11-486B10.4	CASC1
MUC5B	NR3C2
RBM4	CCNA1

RP11-398H6.1	PHF1
WFDC6	PRM2
RP11-799B12.1	TTBK2
MRPL43	ARHGAP28
CNTN6	MTL5
SCRN2	TSSK1B
FBXL15	HRASLS
RP11-270C12.3	PRM1
BEX5	SLC6A16
TLX1	ODF2
C11orf16	STAG3
AGPAT2	SPA17
HTR4	C2orf42
ANP32AP1	FKBP6
TUBAP2	GSG1
ZWINT	SAYSD1
CTC-499B15.4	FRMPD1
RP11-430H10.2	GSTA3
RP11-732A19.5	SLC2A5
ADAMTS4	TEX13A
RPL27A	SMCP
MAFG-AS1	C10orf88
U3	LY6G5C
EXOC3L2	TTC30A
AP000265.1	NUP62CL
RP11-299L17.3	C1QTNF9B-AS1
CTD-2595P9.4	PHF7
FOXA2	RPL10L
TRBV12-5	LRP8
RP11-579D7.4	ZNHIT2
LALBA	NDUFAF3
RP11-473M20.9	PCYT2
AFTPH	SPAG1
FOXA1	SPEF1
PCGF6	CRAT
RP4-610C12.3	INHA
EIF2S2P3	CUL3
SPA17	RNF17
RP11-77H9.6	DUSP13
EIF3FP3	CLGN
RP11-2H3.7	OAZ3
CTD-2288F12.1	TNP1
UNC80	LIN7A
ACTL6B	YBX2
RP11-1280N14.3	DDX4
PGR	ULK2

RCN3	ZNF165
CALY	DNAI1
DTNA	HMGN5
IGKV3-7	CEP350
RP11-29H23.7	VCX2
VPS13A	BAG1
POLA2	IGLL1
ITIH2	TSNAXIP1
USP30	IQCE
PPP1R1A	DXO
ARHGAP19-SLIT1	ADAM3A
RP11-326C3.13	DNAH7
GRIN2C	SPAG6
LINC01485	USP29
CDH15	CDKL2
PCAT18	VRK3
HDGFRP2	SH3TC2
RHOD	DFNB31
CTC-459F4.1	RALGPS1
GUSBP6	PHKG2
FMO2	NRIP3
PSMA7	CYB5R2
CASC8	BRDT
KITLG	TOLLIP
NUTM2E	IFT140
CCDC144A	RSPH6A
GOT1	TBL2
RN7SKP30	KIAA0895
SNORA76C	H2AFJ
C1orf198	VAMP2
PPIAP31	GID4
NPC1L1	ADORA1
WDR18	B9D1
FOXC2	CCDC181
MRPL14	SPINK2
AF131215.9	TLE4
RPL29P19	CNNM1
RP11-697K23.3	C9orf116
PKD1L2	CNTRL
IGKV1-9	CTNNA2
GATA6	SLC30A4
PASK	SRPK2
AP000936.1	CDK20
SH3BP5-AS1	FAAH
NAPRT	PIP5K1C
RNA5-8S5	TP53TG5

RNA5-8S5	BRAP
RNA5-8S5	CCNH
RNA5-8S5	NME5
TLR8-AS1	ATF7IP2
ACAD11	CABYR
HNRNPA1P6	RUFY3
RP11-158H5.2	ZDHHC14
CREBRF	DPEP3
RP1-43E13.2	ZNF415
PUS3	SLC30A3
RP11-438L7.1	NR0B1
IL12B	FAM53C
ACTN4P2	PLSCR2
MEIS3	CSNK2A2
WDR82P1	KLHL11
AF127936.3	DZIP1
RP11-404F10.2	PIK3R3
EPPIN-WFDC6	SIK3
SPOCK3	PAOX
RP11-214O1.3	KCTD13
TUBA8	HAGH
RP11-396C23.2	TTLL1
OSBP2	PGAM2
IL9RP3	GRAMD1B
IQCF5	FHL5
PNLIPRP2	LRRC49
PTGER1	FUZ
OCIAD2	SHARPIN
LL0XNC01-240C2.	PROX1
RP11-492M23.2	CCT8L2
TNNC1	DYNC2H1
COMT	DNALI1
AMH	ASB9
HOXA9	TRIM13
AC111200.7	EIF4G3
OR7E10P	NUP98
AC006042.6	OXR1
NDUFB8	CDK19
C11orf91	FAM216A
ERVMER61-1	OR7E156P
E4F1	SKAP2
RP5-907C10.3	PGR
HMGCS2	CCDC176
AP000593.7	CTSF
CTB-151G24.1	ARL4A
RP11-452K12.3	SPRR2C

AP001631.9	PIWIL2
PFN3	WDR60
SUV39H2	PDXK
PF4	DOCK3
ADI1	ABCA5
CCIN	TFDP3
RP11-44N11.1	GDF10
AC000036.4	PPEF1
RP11-331F9.3	STAG3L3
RP11-554L12.1	NPIPB15
UPK1B	ETNK2
PAMR1	MARK4
MUCL1	AGBL5
RP11-161I6.2	SLC2A8
SMIM24	ELOVL2
TRIP10	TBPL1
RPL35P1	SSX2IP
PNMT	TEKT2
DUS1L	SKAP1
SLC9A5	TMBIM4
MT-ND4L	OPLAH
RP11-84A14.4	TCFL5
RP11-28G8.1	CTAG2
CECR6	ATP8A2
IGSF5	PRPH
RAB3IP	CCSER2
RP11-30J20.1	PAK6
ASF1A	SLC35G2
NCAPH	CHODL
JMJD7-PLA2G4B	AKAP3
KB-1507C5.4	KHDRBS3
LURAP1L	MYLK3
SPDYE16	HGFAC
OR7E91P	POLI
RP11-718O11.1	CRYBG3
IL17RB	TPGS2
SNX29	MRGBP
CHGA	FAM46C
SLCO1B1	SNAP91
CLDN9	MBNL3
ZNF44	INPP4B
SZT2	APPBP2
RFC2	IPO5
PACSIN3	GRB14
RP11-196G11.1	TPTE
SLC17A4	SPAG4

IKBKGP1	ELP5
GPR37	TPP2
GPHN	IQCG
CTC-459F4.9	RNF141
CBR1	MAP7
RP11-493L12.6	INPP1
GFRA2	GPX4
CTD-2161F6.2	TESC
SNORA59B	DPY19L2P2
HIST1H4A	PVRL3
RP11-299H22.6	POMT1
RP11-108L7.15	STAT4
ANOS2P	WT1
CRYBA2	TOMM34
WSCD2	KCNH2
TMEM259	CCDC53
ACTBP8	FBXL2
ALG9	ACE2
VSIG1	CLEC16A
FNDC7	GUCA1A
NTN4	CCNJL
TMEM72	CETN3
ROCK1	IFT81
SV2A	PDE1A
CTC-260F20.3	SCRG1
LINC00632	ENOX1
IGLV3-27	PDZD2
ANPEP	CALB2
C17orf99	WASF1
RCAN1	MLF1
OR7E157P	KLHDC10
PCDHA3	LPIN1
PON3	GLUD2
MSX1	TEX11
CYP4F35P	CUL9
TCEAL8	MAPK13
ARHGAP6	SCCPDH
IP6K3	MAMLD1
ITGAD	SLC12A2
RP11-350G8.3	PRND
HLA-DQA1	SCML1
RBM14	FOXG1
	CDKN3
	EFHC1
	TCEAL2
	PAQR3

CITED1
NAALAD2
ST6GALNAC2
FOXD1
SPHK2
LRRC16A
HSPA2
PMS1
GOLGA8N
MAGEA1
WDR62
CYB5R4
BCL2L14
CYP26B1
USP25
LRRC6
CCDC92
ME3
TTYH1
SLC6A1
NDRG4
AZGP1
PENK
TCEA2
IFT57
CLK4
GPR18
BAZ2B
INHBB
RNF139
MAGEA11
BAZ1A
GSTM3
CAND2
MPC2
GLRX2
MYOM2
SPAG16
GULP1
DMRT1
ZCCHC2
KLK11
ISYNA1
DAZL
RPL39L
CYP17A1

SAR1B
PPARGC1A
ZNF516
EPB41L3
SEC61A2
TMX4
NXF3
ACAT1
SPATA20
MICAL2
HSD17B3
DAAM2
SH3GL2
KLK5
IL13RA2
P4HTM
STX8
OPTN
DACH1
LZTFL1
LRRN3
TKTL1
PRKCZ
C16orf45
REEP1
GATM
SERPINA5
SLC27A6
PPFIBP2
GSTA1
BBX
CENPU
ARNT2
ELAVL4
PRKCDBP
JAM2
OSR2
MAGEA4
KRT23
ZBTB38
ASAP2
TUBA4A
ALDH1A1
CEP70
TNFAIP6
PTGDS

INSL3
CNN1
NEFM
CPE
SMARCA1
PLK2
CLU
PLA2G16
TIPARP
TSPYL5
SOX9
KIF5C
MAGEA12
HOPX
ZBTB20
HMGCS2
CKM
STAR
ABCA8
FHL1
SPOCK1
LIPG
MAGEA6
CADM1
MOK
PFKP
CYP11A1
CST3
DDAH1
SERPINF1
APOA1
BEX1

Table S8

OS gene	Top 40 gene ranked by degree	Venn gene
DPCD	TYROBP	LAPTM5
AC018816.3	HLA-DRA	
RP11-757O6.1	C1QA	
RP11-30P6.6	C1QB	
PTCHD1	CD74	
FADS2	MMP9	
RP1-142L7.9	PRM2	
APOC3	CCL2	
SPDEF	CXCR4	
RP11-857B24.1	SYCP1	
IL5	TNP2	
CUTC	SPP1	
AWAT2	SPO11	
GRIK5	IL13	
SAMD14	HLA-DRB1	
INSRR	FKBP6	
CAV3	LAPTM5	
APOA4	SYCP2	
RP5-963E22.4	TEX12	
LINC01484	STAG3	
RPL23AP25	MMP12	
RP11-242C19.2	AKAP4	
ECHS1	LDHC	
IGHV3-66	PGK2	
SLIT1	CDH1	
CFC1	ACR	
MBD3L1	PRM1	
UPF3AP1	ODF1	
TEX35	CRISP2	
SSSCA1	GAPDHS	
AMHR2	PAPOLB	
RP11-434E6.4	CREM	
C5orf66-AS1	KRT19	
RP11-210M15.2	CAMK4	
TTC41P	DKKL1	
PDE9A	SPA17	
OMA1	CCL19	
NGFRAP1	PIWIL1	
ISYNA1	SPACA1	
CATSPER4	DDX25	
ZNF705G		
METTL12		
RP11-619A14.3		
RP11-799D4.1		

CISD3
DNPH1
IHH
RP11-555K12.1
RP11-177F15.1
RP11-334L9.1
SNAP47-AS1
RP3-337H4.6
RP11-89H19.1
RP6-65G23.5
BEX1
CTC-820M8.1
C1QTNF9
AC007326.10
CTB-1144G6.6
LRRC75A-AS1
CCDC162P
MS4A12
CRX
RP11-588H23.3
HSD17B2
RP11-368M16.3
IGKV1-17
FENDRR
ZNF114
DNAAF2
CYP2A13
ADAMTS7P4
TMEM257
RNF208
RP5-1057I20.5
SFRP5
RP11-88H12.2
IQCC
RP11-51F16.1
FAM90A20P
DMRTC1
RP11-1057B6.1
AC006273.5
ERVWE2
C11orf86
DDRGK1
SIGLEC12
RP11-752G15.10
CTD-2616J11.11
BAAT

MDP1
ARPC3P1
RP11-193H5.2
AC007879.2
LCN15
SLC25A22
RP11-757G1.6
RP11-138I1.4
KCNJ13
ADCK5
RP11-445F12.1
FAM229B
MGAT4A
AVPR1B
ZNF780A
CDK5R2
GPX8
TMLHE-AS1
SFTPD-AS1
CDH7
RP11-299H22.3
TRPC4
RP11-264I13.2
RP11-543B16.2
CENPB
RP11-295D4.4
SMAD6
BIRC5
AP000783.1
DMP1
AMIGO3
CGREF1
CH507-216K13.1
RP1-241P17.4
AC005618.9
SH3PXD2A-AS1
MTHFS
RP11-367N14.2
BMPER
RP13-270P17.3
PHPT1
PNMA6A
RP11-274B21.3
RP11-383B4.4
AC023347.1
MLIP

RP11-725P16.2
PTRF
SMTNL2
NPM3
ZNF7
RP11-55K22.2
RP11-458F8.4
AQP10
PNOC
PORCN
FCRL5
RP5-1028K7.3
TIMM8AP1
DLX3
ATP2C2
HNF1A
KIF20B
SLN
RPS20P4
IGKV1OR2-108
RP11-295M18.6
FKSG54
RN7SL208P
DNAJC17
F2RL3
C1orf105
AP000349.2
RP4-570O12.3
CELF6
NPLOC4
IGSF8
C1orf116
RP3-467L1.6
SCARNA6
RP11-864I4.4
CTB-151G24.2
BX255923.2
C20orf144
HPCAL4
RPS6KB2
GCSAML-AS1
RP1-80N2.2
ABC14-1080714F14.1
GOLGA2P9
XXbac-BPG308K3.5
AC012066.1

SND1
SDHAF1
ASGR1
IGLV4-60
ASS1P2
RP11-482G13.1
RP11-552F3.4
RP11-481J2.2
PSMB5
IRX3
FRY-AS1
CTC-558O2.1
RP11-324H9.1
RP11-496H1.2
ST6GALNAC1
AC010900.2
ARL3
ADAD1
RPS9
RP11-776H12.1
ZC3HAV1L
RN7SL263P
CRHR1-IT1
CD151
RP11-442G21.2
LINC00475
ATP6V1B1-AS1
RPL39L
AKAP4
TERF1P2
RP11-341D18.2
RP11-338N10.3
MRPS14
H2AFX
CXorf49
SMN2
CCDC86
RP11-102K13.5
KCNJ4
ATRIP
EP300-AS1
GINM1
TRIP6
RP5-1096J16.1
CRACR2B
IGLV7-46

CNIH1
FLJ33360
PCDHB5
PXT1
ATP10A
SNHG8
UTS2B
RP11-172E9.2
EEF1E1P1
SLC25A47
DIO2
GLTSCR2-AS1
RP11-420N3.3
AKR1D1
PITX2
SLC2A7
THTPA
RP11-71G12.1
CHD4
CENPCP1
SLC25A23
BCL2L14
HMSD
C14orf80
ATP11B
MT-ND5
ENTHD1
MISP
RP11-193H5.1
FAM149A
RP11-439M11.1
AFP
ANKRD20A5P
TUBB8P1
HRAS
ZNF579
CYP2C18
BTBD16
CEP250
OAT
AC093642.3
TCEB2
DCTN3
RP11-486B10.4
MUC5B
RBM4

RP11-398H6.1
WFDC6
RP11-799B12.1
MRPL43
CNTN6
SCRN2
FBXL15
RP11-270C12.3
BEX5
TLX1
C11orf16
AGPAT2
HTR4
ANP32AP1
TUBAP2
ZWINT
CTC-499B15.4
RP11-430H10.2
RP11-732A19.5
ADAMTS4
RPL27A
MAFG-AS1
U3
EXOC3L2
AP000265.1
RP11-299L17.3
CTD-2595P9.4
FOXA2
TRBV12-5
RP11-579D7.4
LALBA
RP11-473M20.9
AFTPH
FOXA1
PCGF6
RP4-610C12.3
EIF2S2P3
SPA17
RP11-77H9.6
EIF3FP3
RP11-2H3.7
CTD-2288F12.1
UNC80
ACTL6B
RP11-1280N14.3
PGR

RCN3
CALY
DTNA
IGKV3-7
RP11-29H23.7
VPS13A
POLA2
ITIH2
USP30
PPP1R1A
ARHGAP19-SLIT1
RP11-326C3.13
GRIN2C
LINC01485
CDH15
PCAT18
HDGFRP2
RHOD
CTC-459F4.1
GUSBP6
FMO2
PSMA7
CASC8
KITLG
NUTM2E
CCDC144A
GOT1
RN7SKP30
SNORA76C
C1orf198
PPIAP31
NPC1L1
WDR18
FOXC2
MRPL14
AF131215.9
RPL29P19
RP11-697K23.3
PKD1L2
IGKV1-9
GATA6
PASK
AP000936.1
SH3BP5-AS1
NAPRT
RNA5-8S5

RNA5-8S5
RNA5-8S5
RNA5-8S5
TLR8-AS1
ACAD11
HNRNPA1P6
RP11-158H5.2
CREBRF
RP1-43E13.2
PUS3
RP11-438L7.1
IL12B
ACTN4P2
MEIS3
WDR82P1
AF127936.3
RP11-404F10.2
EPPIN-WFDC6
SPOCK3
RP11-214O1.3
TUBA8
RP11-396C23.2
OSBP2
IL9RP3
IQCF5
PNLIPRP2
PTGER1
OCIAD2
LL0XNC01-240C2.1
RP11-492M23.2
TNNC1
COMT
AMH
HOXA9
AC111200.7
OR7E10P
AC006042.6
NDUFB8
C11orf91
ERVMER61-1
E4F1
RP5-907C10.3
HMGCS2
AP000593.7
CTB-151G24.1
RP11-452K12.3

AP001631.9
PFN3
SUV39H2
PF4
ADI1
CCIN
RP11-44N11.1
AC000036.4
RP11-331F9.3
RP11-554L12.1
UPK1B
PAMR1
MUCL1
RP11-161I6.2
SMIM24
TRIP10
RPL35P1
PNMT
DUS1L
SLC9A5
MT-ND4L
RP11-84A14.4
RP11-28G8.1
CECR6
IGSF5
RAB3IP
RP11-30J20.1
ASF1A
NCAPH
JMJD7-PLA2G4B
KB-1507C5.4
LURAP1L
SPDYE16
OR7E91P
RP11-718O11.1
IL17RB
SNX29
CHGA
SLCO1B1
CLDN9
ZNF44
SZT2
RFC2
PACSIN3
RP11-196G11.1
SLC17A4

IKBKGP1
GPR37
GPHN
CTC-459F4.9
CBR1
RP11-493L12.6
GFRA2
CTD-2161F6.2
SNORA59B
HIST1H4A
RP11-299H22.6
RP11-108L7.15
ANOS2P
CRYBA2
WSCD2
TMEM259
ACTBP8
ALG9
VSIG1
FNDC7
NTN4
TMEM72
ROCK1
SV2A
CTC-260F20.3
LINC00632
IGLV3-27
ANPEP
C17orf99
RCAN1
OR7E157P
PCDHA3
PON3
MSX1
CYP4F35P
TCEAL8
ARHGAP6
IP6K3
ITGAD
RP11-350G8.3
HLA-DQA1
RBM14
